# Meerwein‐type Bromoarylation with Arylthianthrenium Salts

**DOI:** 10.1002/anie.202209882

**Published:** 2022-10-20

**Authors:** Yuan Cai, Tobias Ritter

**Affiliations:** ^1^ Max-Planck-Institut für Kohlenforschung Kaiser-Wilhelm-Platz 1 45470 Mülheim an der Ruhr Germany

**Keywords:** Arylthianthrenium Salts, Bromoarylation of Alkenes, Late-Stage Functionalization, Meerwein Arylation

## Abstract

Herein, we report a photocatalyzed Meerwein‐type bromoarylation of alkenes with stable arylthianthrenium salts, formed by site‐selective C−H thianthrenation. This protocol can be applied to late‐stage functionalization of a variety of biomolecules that are difficult to access by other aryl coupling reagents. Halogen introduction allows for a variety of follow‐up transformations, affording numerous biologically active skeletons.

## Graphical abstract

Despite more than 80 years of research, Meerwein haloarylation with complex small molecules has not been possible because the required aryldiazonium salts are often not readily accessible from complex structures. We report the first late stage Meerwein‐type bromoarylation of alkenes with stable arylthianthrenium salts, which provides the opportunity to generate complexity quickly.

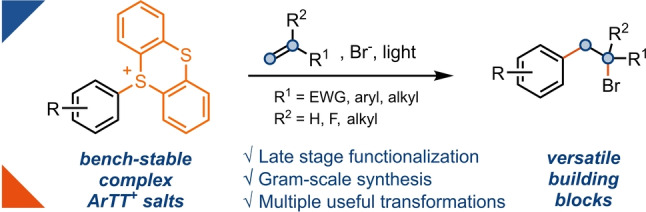

Since the first report by Hans Meerwein in 1939,^[1]^ radical arylation of alkenes has provided multiple useful C−C bond‐forming reactions.^[2]^ However, Meerwein arylations require thermally unstable or even explosive^[3]^ aryldiazonium salts.^[4]^ Moreover, multiple side reactions, such as the Sandmeyer reaction and formation of azo compounds typically result in low yield of the desired product in traditional Meerwein arylation, and late‐stage functionalizations have not been reported at all. Meerwein arylations can therefore currently not be used to functionalize complex small molecules in synthetically useful yields.^[5]^ Herein, we present a photocatalyzed Meerwein‐type bromoarylation of alkenes with stable arylthianthrenium salts that can furnish synthetically valuable alpha‐bromo‐substituted carboxylic acid and related derivatives, such as sulfones and phosphates, from various Michael acceptors and styrenes. The transformation can be applied to late stage functionalization of complex small molecules. The C−Br bond can be transformed to C−N, C−O, C−S, C−P, and C−Se bonds smoothly, to afford a variety of privileged drug scaffolds, such as phenylalanines and thiazolidinediones, which can be introduced to pharmaceutically relevant molecules.

Chemists make synthetic use of aryl radicals because they provide a complementary strategy for arylation reactions in addition to transition‐metal‐mediated cross‐coupling reactions of aryl halides.^[2]^ Meerwein arylations can currently be accomplished by generation of aryl radicals from aryldiazonium^[4]^ and ‐iodonium salts.^[6]^ Gaunt and co‐workers have described a Cu‐mediated Meerwein azido‐arylation and bromoarylation using diaryliodonium salts.^[6b]^ Late‐stage Meerwein arylation provides the opportunity to generate complexity quickly due to the modularity of the reaction; each of the three reaction components, that is, the aryl radical source, the olefin, and the trapping reagent can be modified as part of the multicomponent reaction.^[4]^ Conventional copper‐catalyzed Meerwein haloarylation reactions are complicated by side reactions.[^4e^, ^4f^] For example, copper also induces the Sandmeyer reaction,^[7]^ which results in halogenated byproducts because the rate of halogen atom transfer from CuX_2_ to aryl radical is often faster (*k*≈10^8^ M^−1^ s^−1^)^[8]^ than the rate of addition of aryl radical to activated (*k*≈10^8^ M^−1^ s^−1^)^[9]^ and unactivated olefins (*k*≈10^7^ M^−1^ s^−1^).^[10]^ Stable aryl halides are generally either too difficult to reduce to radicals^[11]^ or too slow to fragment^[12]^ due to their high negative reduction potential, which limits their applications for Meerwein arylations. Jui and co‐workers reported several radical hydroarylation and hydroheteroarylation reactions of olefins.^[13]^ Chen and co‐workers reported an atom‐economical cross‐coupling of halopyridines and unactivated alkenes under photoredox catalysis.^[14a]^ Wang and co‐workers reported a photocatalyzed hydroarylation of azine‐substituted enamides with the in situ‐generated aryl thianthrenium salts.^[14b]^ Nevertheless, Meerwein haloarylation with complex small molecules have not been accomplished so far.[^6b^, ^13^, ^14^, ^15^]

Our group reported a site‐selective thianthrenation methodology of structurally complex arenes, to afford stable arylthianthrenium salts,^[16a]^ which can be used as aryl radical precursors for late‐stage formation of C−CF_3_,^[16b]^ C−N,^[16c]^ C−O,^[16d]^ and C−F^12b^ bonds under copper photoredox catalysis. Because arylthianthrenium salts are more readily accessible than aryldiazonium and ‐iodonium salts and do not share the same limitations as halides for reduction owing to their positive charge, we evaluated their potential for Meerwein arylation reactions (Figure 1). Combination of arylthianthrenium salts under photoredox conditions with copper sources for attempted bromoarylation of methyl acrylate resulted in serious Sandmeyer side reaction to afford aryl bromides (see Table S1). Yet, we found that the reaction proceeded productively in the presence of photocatalysts without copper, albeit at a low efficiency. *N*‐phenyl‐benzo[*b*]phenothiazine (**PTH**) photocatalysts^[17]^ performed best when compared to metal (Ru, Ir) based photocatalysts (see Table S1). In the absence of copper, the undesired Sandmeyer bromination product was negligible (<5 %). Reaction optimization revealed that irradiation of a mixture of arylthianthrenium salt **TT‐1**, 3.0 equiv methyl acrylate, and 2.0 equiv tetrabutylammonium bromide (TBAB) with white light‐emitting diodes (LEDs) in acetonitrile at −20 °C for 2 h afforded the desired bromoarylation product **1** in 68 % isolated yield (Figure 2). The reaction could be readily accomplished similarly effectively on a gram‐scale (63 % yield) with 93 % recovery of thianthrene.

**Figure 1:**
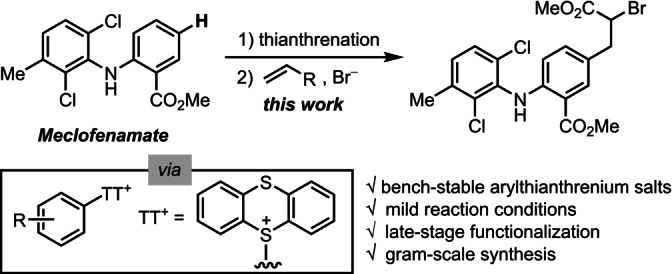
Radical arylations of alkenes through Meerwein‐type haloarylation.

**Figure 2:**
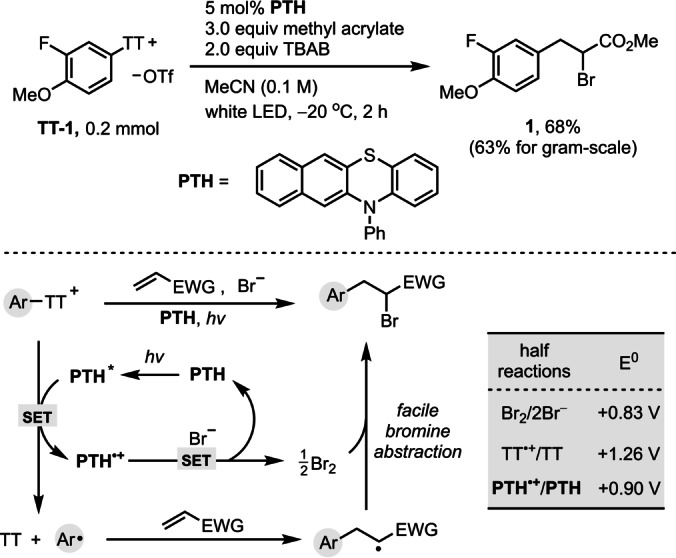
Optimized reaction conditions and proposed mechanism of arylthianthrenium salts mediated Meerwein‐type bromoarylation of alkene.

Based on the experimental observations and Stern–Volmer quenching experiments, we proposed the mechanism shown in Figure 2, with oxidative quenching of the highly reducing excited **PTH** (−1.92 V vs SCE in DMA)^[17b]^ by the arylthianthrenium salt^[15f]^ **TT‐1** to generate, upon mesolytic cleavage, aryl radicals and **PTH**⋅^**+**^. An electron donor acceptor complex between arylthianthrenium salt **TT‐1** and **PTH** was not observed (see Figure S1). Bromide anion would be oxidized by **PTH**⋅^**+**^ in agreement with the respective oxidation potentials (Figure 2).^[18]^ This assumption is supported by the independent reaction between **PTH**⋅^**+**^ and TBAB (see Figure S7). Conducting the experiment under standard conditions in the presence of 1,3,5‐trimethoxybenzene resulted in a slightly lower yield of **1**, and 2‐bromo‐1,3,5‐trimethoxybenzene was detected in 5 % yield, further supporting the intermediacy of free bromine (see Figure S8). Subsequent facile bromine abstraction by the arylethyl radical afford the final bromoarylation product.

In combination with late‐stage thianthrenation of arenes,^[16a]^ our protocol can quickly access substituted complex small molecule that are not readily accessible by current methods. No other constitutional isomers were observed for any of the compounds shown in Table 1, enabling straightforward purification. Electron‐deficient (**17)**, ‐neutral (**12**, **23)** and ‐rich (**2)** arenes all afforded comparable yields, whereas electron‐donating groups on aryldiazonium salts generally are problematic in conventional Meerwein arylations, possibly owing to faster halogen abstraction from CuX_2_.^[10]^ Another distinguishing feature of the transformation disclosed herein is the ability to access di‐ and even trisubstituted (**14**, **18**, **26**) arylthianthrenium salts from arenes, while most aryldiazonium salts used in conventional Meerwein bromoarylation reactions are limited to mono‐ or polyhalosubstituted anilines.^[4]^ The average yield of the products shown in Table 1 is 56 % with oligomer and aryl‐H being the two major side products observed. While both side products reduce the yield, it is important to note that other methods are currently not able to afford the products in a single step. The reaction is tolerant towards a variety of functional groups including cyclopropyl, alkyl chlorides, aryl halides, aryl pseudohalides, ethers, aldehyde, ketones, esters, amides, sulfonamides, nitriles, nitro groups, and heteroaromatics, such as pyridine (**7**), benzofuran (**21**) and indol (**26**), as well as protic groups, such as secondary amines and amides. Benzyloxy groups (**22**, **25**) and *ortho*‐alkoxy‐substituted arenes (**16**, **18**, **26**) decreased the product yields and form larger quantities of hydrodefunctionalized side products. Several Michael acceptors are tolerated: benzyl (**27**) and tertiary butyl acrylates (**28**), acrylonitrile (**29**), methyl vinyl ketone (**30**), vinyl sulfone (**31**), vinyl phosphate (**32**), *α*‐methylene lactone (**33**), fluoroacrylate (**34**), methacrolein (**35**) and protected (**36**) or unprotected acrylamide (**37**). Monosubstituted styrenes (**38**, **39**) are also compatible, and aryl‐alkene coupling products were not observed.^[4a]^ Generally, reactions with non‐activated alkenes are more difficult to conduct, since the slower addition step facilitates undesired side reactions of the aryl radical, such as Sandmeyer halogenation and hydrogen abstraction.^[19]^ In our protocol, the non‐activated 4‐bromo‐1‐butene (**40**) was also reactive productively albeit requiring higher equivalents (Table 1).

**Table 1:**
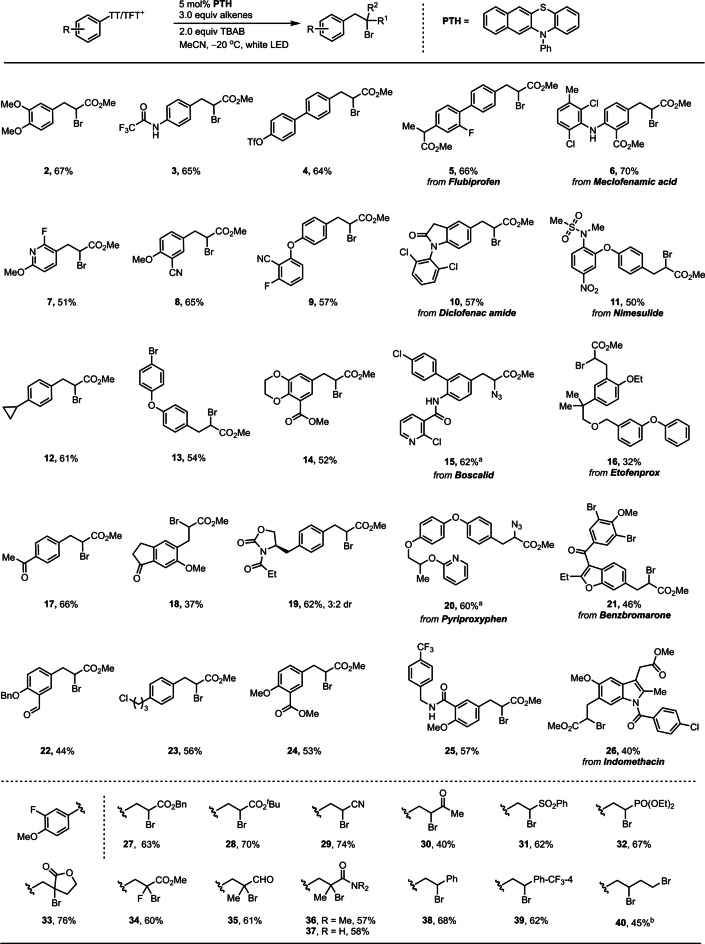
Substrate scope of Meerwein bromoarylation with arylthianthrenium Salts.

[a] After the reaction, NaN_3_ was added and stirred at room temperature overnight. [b] 10.0 equiv of alkenes were added.

Meerwein haloarylation forms a new C(sp^2^)−C(sp^3^) bond and also introduces a halide functionality for subsequent further transformations. To demonstrate the synthetic utility of bromoarylation products in our protocol, several follow‐up transformations were successfully conducted with nitrogen‐, sulfur‐, oxygen‐, phosphine‐ or selenium‐based nucleophiles (Figure 3). Numerous valuable functional groups, heterocyclic compounds, and privileged drug scaffolds, such as phenylalanine (**15**, **20**, **41**) and thiazolidinedione (**49**), were obtained.

**Figure 3:**
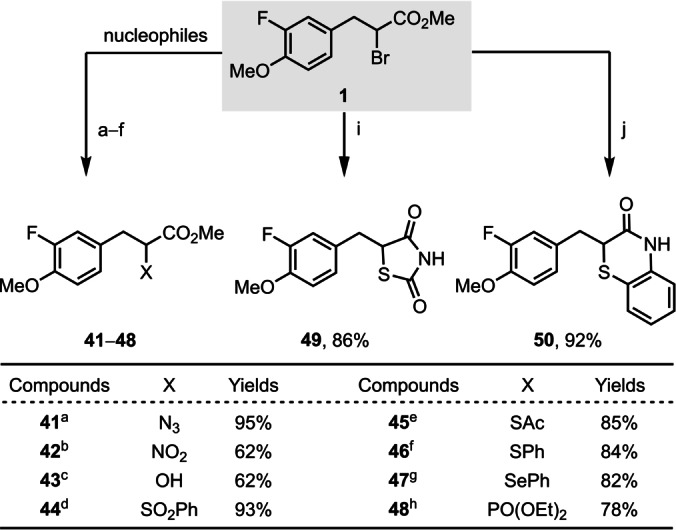
Transformations of this protocol. ^a^ NaN_3_, phenyl acetylene, CuI (20 mol %), ^*t*^BuOH, H_2_O; ^b^ NaNO_2_, phloroglucinol; ^c^ HCO_2_Na, MeOH; ^d^ PhSO_2_Na, DMF; ^e^ AcSK, DMF; ^f^ PhSH, K_2_CO_3_, DMF; ^g^ PhSeSePh, NaBH_4_; ^h^ P(OEt)_3_; ^i^ thiourea, EtOH, then 6 N HCl; ^j^ 2‐aminothiophenol, DMF.

In conclusion, we have realized a Meerwein‐type bromoarylation of activated alkenes, including at a late‐stage for the first time, with arylthianthrenium salts, catalyzed by an organic photocatalyst **PTH**. The reaction proceeds in the absence of copper with broad functional group tolerance. Combined with site‐selective thianthrenation of arenes^[16a]^ and versatile follow‐up transformations, we believe the transformation will be a valuable approach in rapid drug discovery.

## Conflict of interest

The authors declare no conflict of interest.

## Supporting information

As a service to our authors and readers, this journal provides supporting information supplied by the authors. Such materials are peer reviewed and may be re‐organized for online delivery, but are not copy‐edited or typeset. Technical support issues arising from supporting information (other than missing files) should be addressed to the authors.

Supporting Information

## Data Availability

The data that support the findings of this study are available in the supplementary material of this article.
