# Supplementary material for: Meerwein‐type Bromoarylation with Arylthianthrenium Salts
Source: Angew Chem Int Ed Engl. 2022 Oct 20;61(47):e202209882. doi: 10.1002/anie.202209882 (PMC9828184; doi:10.1002/anie.202209882)
Supplement: Supplementary file 1 — Supporting Information [file ANIE-61-0-s001.pdf]

Angewandte Chemie
Eine Zeitschrift der Gesellschaft Deutscher Chemiker

Supporting Information

Meerwein-type Bromoarylation with Arylthianthrenium Salts

*Y. Cai, T. Ritter**

TABLE OF CONTENTS

TABLE OF CONTENTS....1

MATERIALS AND METHODS....8

EXPERIMENTAL DATA....9

Bromoarylation reaction with arylthianthrenium salts....9

General procedure for bromoarylation reaction with arylthianthrenium salts....9

(±)-2-Bromo-arylpropanoate **1**....10

(±)-2-Bromo-arylpropanoate **2**....11

(±)-2-Bromo-arylpropanoate **3**....11

(±)-2-Bromo-biarylpropanoate **4**....12

(±)-Flurbiprofen derivative **5**....13

(±)-Methyl meclofenamate derivative **6**....13

(±)-2-Bromo-heteroarylpropanoate **7**....14

(±)-2-Bromo-arylpropanoate **8**....15

(±)-2-Bromo-arylpropanoate **9**....16

(±)-Diclofenac amide derivative **10**....16

(±)-*N*-Methyl-nimesulide derivative **11**....17

(±)-2-Bromo-arylpropanoate **12**....18

(±)-2-Bromo-arylpropanoate **13**....18

(±)-2-Bromo-arylpropanoate **14**....19

(±)-Boscalid derivative **15**....20

(±)-Etofenprox derivative **16**....21

(±)-2-Bromo-arylpropanoate **17**....21

(±)-2-Bromo-arylpropanoate **18**....22

(±)-2-Bromo-arylpropanoate **19**....23

(±)-Pyriproxyphen derivative **20**....24

(±)-Benzbromarone derivative **21**....24

(±)-2-Bromo-arylpropanoate **22**....25

(±)-2-Bromo-arylpropanoate **23**....26

(±)-2-Bromo-arylpropanoate **24**....27

(±)-2-Bromo-arylpropanoate **25**....27

(±)-Indometacin methylester derivative **26**....28

(±)-2-Bromo-arylpropanoate **27**....29

(±)-2-Bromo-arylpropanoate **28**....30

(±)-2-Bromo-arylpropanenitrile **29**....30

(±)-3-Bromo-arylbutan-2-one **30**....31

(±)-2-Bromo-(phenylsulfonyl)ethylarene **31** 32
(±)-1-Bromo-arylethylphosphonate **32** 32
(±)-Butyrolactone **33** 33
(±)-2-Bromo-2-fluoro-arylpropanoate **34** 34
(±)-2-Bromo-arylpropanal **35** 35
(±)-2-Bromo-arylpropanamide **36** 35
(±)-2-Bromo-arylpropanamide **37** 36
(±)-2-Bromo-2-phenylethylarene **38** 37
(±)-2-Bromo-2-phenylethylarene **39** 37
(±)-2,4-Dibromobutylarene **40** 38

Synthetic utility 39

Synthesis of (±)-methyl 2-azidopropanoate **41** 39
Synthesis of (±)-methyl 2-nitropropanoate **42** 39
Synthesis of (±)-methyl 2-hydroxy-propanoate **43** 40
Synthesis of (±)-methyl 2-phenylsulfonylpropanoate **44** 41
Synthesis of (±)-methyl 2-acetylthiopropanoate **45** 41
Synthesis of (±)-methyl 2-phenylthiopropanoate **46** 42
Synthesis of (±)-methyl 2-phenylselanylpropanoate **47** 42
Synthesis of (±)-methyl 2-diethoxyphosphorylpropanoate **48** 43
Synthesis of (±)-thiazolidinediones derivative **49** 44
Synthesis of (±)-2*H*-benzo[*b*]-[1,4]thiazin-3(4*H*)-one **50** 44

Reaction condition optimization 45

Table S1. Screening of photocatalysts, solvents and reaction temperature 45
Table S2. Other (pseudo)halides 46
Table S3. Trials on hydroarylation 46

Unsuccessful substrates 46

Mechanistic studies 48

UV-vis Absorption Spectrum 48
Stern-Volmer Luminescence Quenching Studies 49
Stoichiometric reaction between $PTH^{\bullet+}$ and TBAB 53
Free bromine trapping experiments 54

SPECTROSCOPIC DATA 56

^{1}H NMR of (±)-2-bromo-arylpropanoate **1** 56

^{13}C NMR of (±)-2-bromo-arylpropanoate **1** 57

^{19}F NMR of (±)-2-bromo-arylpropanoate **1** 58

^{1}H NMR of (±)-2-bromo-arylpropanoate **2** 59

^{13}C NMR of (±)-2-bromo-arylpropanoate **2** 60

^{1}H NMR of (±)-2-bromo-arylpropanoate **3** 61

^{13}C NMR of (±)-2-bromo-arylpropanoate **3** 62

^{19}F NMR of (±)-2-bromo-arylpropanoate **3** 63

^{1}H NMR of (±)-2-bromo-arylpropanoate **4** 64

^{13}C NMR of (±)-2-bromo-arylpropanoate **4** 65

^{1}H NMR of (±)-flurbiprofen derivative **5** 66

^{13}C NMR of (±)-flurbiprofen derivative **5** 67

^{1}H NMR of (±)-methyl meclofenamate derivative **6** 68

^{13}C NMR of (±)-methyl meclofenamate derivative **6** 69

^{1}H NMR of (±)-2-bromo-arylpropanoate **7** 70

^{13}C NMR of (±)-2-bromo-arylpropanoate **7** 71

^{19}F NMR of (±)-2-bromo-arylpropanoate **7** 72

^{1}H NMR of (±)-2-bromo-arylpropanoate **8** 73

^{13}C NMR of (±)-2-bromo-arylpropanoate **8** 74

^{1}H NMR of (±)-2-bromo-arylpropanoate **9** 75

^{13}C NMR of (±)-2-bromo-arylpropanoate **9** 76

^{1}H NMR of (±)-diclofenac amide derivative **10** 77

^{13}C NMR of (±)-diclofenac amide derivative **10** 78

^{1}H NMR of (±)-*N*-methyl-nimesulide derivative **11** 79

^{13}C NMR of (±)-*N*-methyl-nimesulide derivative **11** 80

^{1}H NMR of (±)-2-bromo-arylpropanoate **12** 81

^{13}C NMR of (±)-2-bromo-arylpropanoate **12** 82

^{1}H NMR of (±)-2-bromo-arylpropanoate **13** 83

^{13}C NMR of (±)-2-bromo-arylpropanoate **13** 84

^{1}H NMR of (±)-2-bromo-arylpropanoate **14** 85

^{13}C NMR of (±)-2-bromo-arylpropanoate **14** 86

^{1}H NMR of (±)-boscalid derivative **15** 87

^{13}C NMR of (±)-boscalid derivative **15** 88

^{1}H NMR of (±)-etofenprox derivative **16** 89

^{13}C NMR of (±)-etofenprox derivative **16** 90

^{1}H NMR of (±)-2-bromo-arylpropanoate **17** 91

^{13}C NMR of (±)-2-bromo-arylpropanoate **17** 92

^{1}H NMR of (±)-2-bromo-arylpropanoate **18** 93

^{13}C NMR of (±)-2-bromo-arylpropanoate **18** 94

^{1}H NMR of (±)-2-bromo-arylpropanoate **19** 95

^{13}C NMR of (±)-2-bromo-arylpropanoate **19** 96

^{1}H NMR of (±)-pyriproxyphen derivative **20** 97

^{13}C NMR of (±)-pyriproxyphen derivative **20** 98

^{1}H NMR of (±)-benzbromarone derivative **21** 99

^{13}C NMR of (±)-benzbromarone derivative **21** 100

^{1}H NMR of (±)-2-bromo-arylpropanoate **22** 101

^{13}C NMR of (±)-2-bromo-arylpropanoate **22** 102

^{1}H NMR of (±)-2-bromo-arylpropanoate **23** 103

^{13}C NMR of (±)-2-bromo-arylpropanoate **23** 104

^{1}H NMR of (±)-2-bromo-arylpropanoate **24** 105

^{13}C NMR of (±)-2-bromo-arylpropanoate **24** 106

^{1}H NMR of (±)-2-bromo-arylpropanoate **25** 107

^{13}C NMR of (±)-2-bromo-arylpropanoate **25** 108

^{19}F NMR of (±)-2-bromo-arylpropanoate **25** 109

^{1}H NMR of (±)-Indometacin methylester derivative **26** 110

^{13}C NMR of (±)-Indometacin methylester derivative **26** 111

^{1}H NMR of (±)-2-bromo-arylpropanoate **27** 112

^{13}C NMR of (±)-2-bromo-arylpropanoate **27** 113

^{19}F NMR of (±)-2-bromo-arylpropanoate **27** 114

^{1}H NMR of (±)-2-bromo-arylpropanoate **28** 115

^{13}C NMR of (±)-2-bromo-arylpropanoate **28** 116

^{19}F NMR of (±)-2-bromo-arylpropanoate **28** 117

^{1}H NMR of (±)-2-bromo-arylpropanenitrile **29** 118

^{13}C NMR of (±)-2-bromo-arylpropanenitrile **29** 119

^{19}F NMR of (±)-2-bromo-arylpropanenitrile **29** 120

^{1}H NMR of (±)-3-bromo-arylbutan-2-one **30** 121

^{13}C NMR of (±)-3-bromo-arylbutan-2-one **30** 122

^{1}H NMR of (±)-2-bromo-(phenylsulfonyl)ethylarene **31** 123

^{13}C NMR of (±)-2-bromo-(phenylsulfonyl)ethylarene **31** 124

^{19}F NMR of (±)-2-bromo-(phenylsulfonyl)ethylarene **31** 125

^{1}H NMR of (±)-1-bromo-arylethylphosphonate **32** 126

^{13}C NMR of (±)-1-bromo-arylethylphosphonate **32** 127

^{19}F NMR of (±)-1-bromo-arylethylphosphonate **32** 128

^{31}P NMR of (±)-1-bromo-arylethylphosphonate **32** 129

^{1}H NMR of (±)-butyrolactone **33** 130

^{13}C NMR of (±)-butyrolactone **33** 131

^{19}F NMR of (±)-butyrolactone **33** 132

^{1}H NMR of (±)-2-bromo-2-fluoro-arylpropanoate **34** 133

^{13}C NMR of (±)-2-bromo-2-fluoro-arylpropanoate **34** 134

^{19}F NMR of (±)-2-bromo-2-fluoro-arylpropanoate **34** 135

^{1}H NMR of (±)-2-bromo-arylpropanal **35** 136

^{13}C NMR of (±)-2-bromo-arylpropanal **35** 137

^{19}F NMR of (±)-2-bromo-arylpropanal **35** 138

^{1}H NMR of (±)-2-bromo-arylpropanamide **36** 139

^{13}C NMR of (±)-2-bromo-arylpropanamide **36** 140

^{19}F NMR of (±)-2-bromo-arylpropanamide **36** 141

^{1}H NMR of (±)-2-bromo-arylpropanamide **37** 142

^{13}C NMR of (±)-2-bromo-arylpropanamide **37** 143

^{19}F NMR of (±)-2-bromo-arylpropanamide **37** 144

^{1}H NMR of (±)-2-bromo-2-phenylethylarene **38** 145

^{13}C NMR of (±)-2-bromo-2-phenylethylarene **38** 146

^{1}H NMR of (±)-2-bromo-2-phenylethylarene **39** 147

^{13}C NMR of (±)-2-bromo-2-phenylethylarene **39** 148

^{19}F NMR of (±)-2-bromo-2-phenylethylarene **39** 149

^{1}H NMR of (±)-2,4-dibromobutylarene **40** 150

^{13}C NMR of (±)-2,4-dibromobutylarene **40** 151

^{19}F NMR of (±)-2,4-dibromobutylarene **40** 152

^{1}H NMR of (±)-methyl 2-azidopropanoate **41** 153

^{13}C NMR of (±)-methyl 2-azidopropanoate **41** 154

^{19}F NMR of (±)-methyl 2-azidopropanoate **41** 155

^{1}H NMR of (±)-methyl 2-nitropropanoate **42** 156

^{13}C NMR of (±)-methyl 2-nitropropanoate **42** 157

^{1}H NMR of (±)-methyl 2-hydroxy-propanoate **43** 158

^{13}C NMR of (±)-methyl 2-hydroxy-propanoate **43** 159

^{19}F NMR of (±)-methyl 2-hydroxy-propanoate **43** 160

^{1}H NMR of (±)-methyl 2-phenylsulfonylpropanoate **44** 161

^{13}C NMR of (±)-methyl 2-phenylsulfonylpropanoate **44** 162

^{19}F NMR of (±)-methyl 2-phenylsulfonylpropanoate **44** 163

^{1}H NMR of (±)-methyl 2-acetylthiopropanoate **45** 164

^{13}C NMR of (±)-methyl 2-acetylthiopropanoate **45** 165

^{19}F NMR of (±)-methyl 2-acetylthiopropanoate **45** 166

^{1}H NMR of (±)-methyl 2-phenylthiopropanoate **46** 167

^{13}C NMR of (±)-methyl 2-phenylthiopropanoate **46** 168

^{19}F NMR of (±)-methyl 2-phenylthiopropanoate **46** 169

^{1}H NMR of (±)-methyl 2-phenylselanylpropanoate **47** 170

^{13}C NMR of (±)-methyl 2-phenylselanylpropanoate **47** 171

^{19}F NMR of (±)-methyl 2-phenylselanylpropanoate **47** 172

^{1}H NMR of (±)-methyl 2-diethoxyphosphorylpropanoate **48** 173

^{13}C NMR of (±)-methyl 2-diethoxyphosphorylpropanoate **48** 174

^{31}P NMR of (±)-methyl 2-diethoxyphosphorylpropanoate **48** 175

^{1}H NMR of (±)-thiazolidinediones derivative **49** 176

^{13}C NMR of (±)-thiazolidinediones derivative **49** 177

^{19}F NMR of (±)-thiazolidinediones derivative **49** 178

^{1}H NMR of (±)-2*H*-benzo[*b*]-[1,4]thiazin-3(4*H*)-one **50** 179

^{13}C NMR of (±)-2*H*-benzo[*b*]-[1,4]thiazin-3(4*H*)-one **50** 180

^{19}F NMR of (±)-2*H*-benzo[*b*]-[1,4]thiazin-3(4*H*)-one **50** 181

REFERENCES 182

MATERIALS AND METHODS

All air- and moisture-insensitive reactions were carried out under ambient atmosphere and monitored by thin-layer chromatography (TLC). Concentration under reduced pressure was performed by rotary evaporation at 25–40 °C at an appropriate pressure. Purified compounds were further dried under high vacuum (0.010–0.005 mbar). Yields refer to purified and spectroscopically pure compounds. All air- and moisture-sensitive manipulations were performed using oven-dried glassware (120 °C for a minimum of 12 hours) and standard Schlenk techniques under an atmosphere of argon.

Solvents

Anhydrous MeCN was obtained from *Phoenix Solvent Drying Systems*. Anhydrous acetone, DMF, DMSO, and AcOMe were dried by storage over 4Å molecular sieves. All deuterated solvents were purchased from *Euriso-Top*.

Chromatography

Thin layer chromatography (TLC) was performed using EMD TLC plates pre-coated with 250 μm thickness silica gel 60 F254 plates and visualized by fluorescence quenching under 254 nm UV light, permanganate stain, cerium ammonium molybdate stain, or phosphomolybdic acid stain. Flash chromatography was performed using silica gel (40–63 μm particle size) purchased from Geduran®.

Spectroscopy and Instruments

NMR spectra were recorded on a *Bruker Ascend* TM 500 spectrometer operating at 500 MHz, 471 MHz and 126 MHz, for ^{1}H, ^{19}F, and ^{13}C acquisitions, respectively. Chemical shifts are reported in ppm with the solvent residual peak as the internal standard. For ^{1}H NMR: $CDCl_3$, 7.26; CD_3CN, 1.96; CD_2Cl_2, 5.32; For ^{13}C NMR: $CDCl_3$, 77.16; CD_3CN, 1.32; CD_2Cl_2, 53.84.[1] Data is reported as follows: s = singlet, d = doublet, t = triplet, q = quartet, quin = quintet, sext = sextet, sept = septet, m = multiplet, bs = broad singlet; coupling constants in Hz; integration.

Starting materials

All substrates were used as received from commercial suppliers or prepared according to published procedures, unless otherwise stated. Chemicals were purchased from *Sigma-Aldrich*, *TCI*, or *Alfa Aesar*. Thianthrene-S-oxide (**TTO**)[2] and phenylphenothiazinium radical cation (**PTH**) were prepared according to the literature.[3]

EXPERIMENTAL DATA

Bromoarylation reaction with arylthianthrenium salts

General procedure for bromoarylation reaction with arylthianthrenium salts

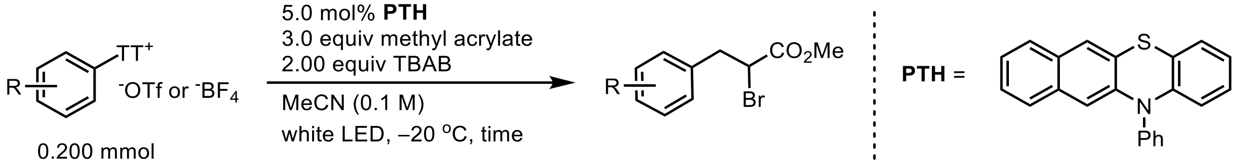

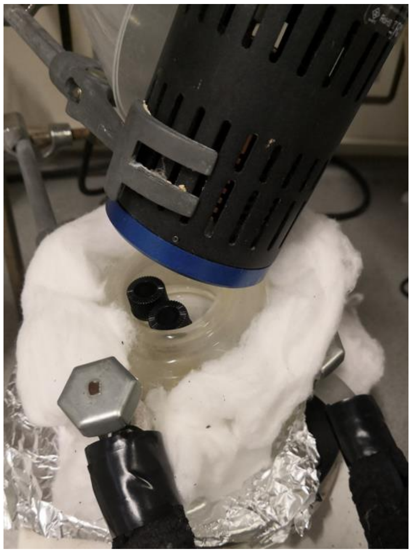

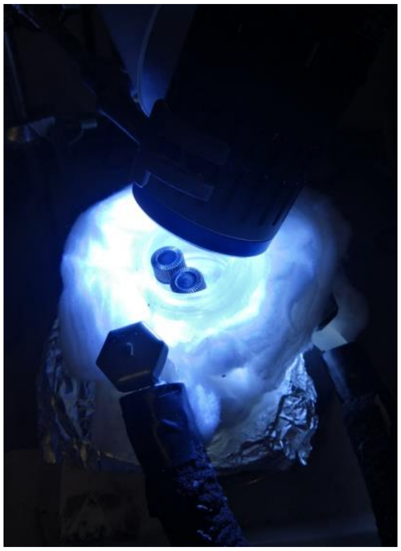

To a 4-mL borosilicate vial containing a Teflon-coated magnetic stirring bar were added aryl thianthrenium salt (0.200 mmol), photoredox catalyst **PTH (**3.2 mg, 10 µmol, 5.0 mol%**)**, and tetrabutylammonium bromide (TBAB, 128 mg, 0.400 mmol, 2.00 equiv). The vial was transferred into a nitrogen-filled glovebox. Alternatively, the vial could be evacuated and backfilled with inert gas using a Schlenk line. For simplicity, in our research, we have opted to execute the transformation for most compounds by using a glovebox. Control experiments showed that yields were within error of measurement. Dry MeCN (2 mL, c = 0.1 M) and the alkene (0.60 mmol, 3.0 equiv) were added. The vial was sealed with a Teflon-lined screw cap and transferred to a cryocooler precooled at –20 °C where the reaction mixture was stirred for 5 min without irradiation and then irradiated with a white LED (40 W) for 2–8 h until a substantial precipitate formed. Subsequently, silica gel (5–10 mL) was added, and the reaction mixture was concentrated to dryness under reduced pressure. The resulting residue was purified by chromatography on silica gel (hexanes/EtOAc) to afford the desired product.

(±)-2-Bromo-arylpropanoate 1

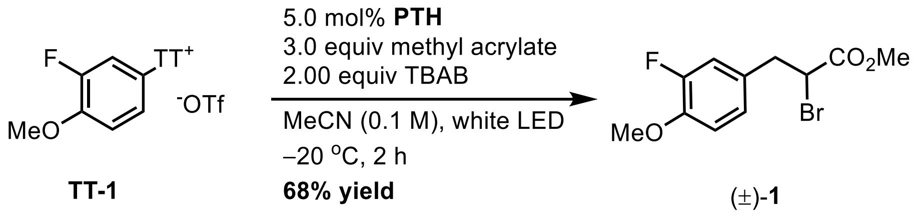

To a 4-mL borosilicate vial containing a Teflon-coated magnetic stirring bar were added **TT-1** (98.0 mg, 0.200 mmol, 1.00 equiv), **PTH** (3.2 mg, 10 μmol, 5.0 mol%), and tetrabutylammonium bromide (TBAB, 128 mg, 0.400 mmol, 2.00 equiv). The vial was transferred into a nitrogen-filled glovebox. Dry MeCN (2 mL, c = 0.1 M) and methyl acrylate (54 μL, 52 mg, 0.60 mmol, 3.0 equiv) were added. The vial was sealed with a Teflon-lined screw cap, removed from the glovebox, and transferred to a cryocooler precooled at –20 °C where the reaction mixture was stirred for 5 min without irradiation and then irradiated with white LEDs for 2 h. Subsequently, silica gel (~5 mL) was added and the reaction mixture was concentrated to dryness under reduced pressure. The residue was purified by flash column chromatography on silica gel (hexanes/EtOAc = 20:1) to afford (±)-**1** as a colorless oil in 68% yield (39.3 mg).

R*f* = 0.20 (hexanes/EtOAc = 20:1).

NMR Spectroscopy:

^{1}H NMR (500 MHz, $CDCl_3$, 23 °C, δ): 7.01 – 6.83 (m, 3H), 4.34 (dd, *J* = 8.4, 7.0 Hz, 1H), 3.86 (s, 3H), 3.73 (s, 3H), 3.38 (dd, *J* = 14.2, 8.4 Hz, 1H), 3.16 (dd, *J* = 14.3, 7.0 Hz, 1H).

^{13}C NMR (125 MHz, $CDCl_3$, 23 °C, δ): 169.7, 152.2 (d, *J* = 246.3 Hz), 146.9 (d, *J* = 10.4 Hz), 129.6 (d, *J* = 6.1 Hz), 125.0 (d, *J* = 3.6 Hz), 116.8 (d, *J* = 18.5 Hz), 113.4 (d, *J* = 2.3 Hz), 56.2, 53.0, 44.8, 40.1.

^{19}F NMR (470 MHz, $CDCl_3$, 23 °C, δ): –134.8 (dd, *J* = 11.5, 7.8 Hz).

HRMS-EI (m/z) calc'd for $C_{11}H_{12}O_3FBr$ $[M]^+$, 289.9948; found, 289.9951; deviation: –0.8 ppm.

Gram-scale reaction:

To a 250 mL two-neck flask containing a Teflon-coated magnetic stirring bar were added **TT-1** (3.43 g, 7.00 mmol, 1.00 equiv), **PTH** (112 mg, 35.0 mmol, 5.00 mol%), and tetrabutylammonium bromide (TBAB, 4.48 g, 14.0 mmol, 2.00 equiv) under argon. Dry and degassed MeCN (70 mL, c = 0.1 M) and methyl acrylate (1.89 mL, 1.81 g, 21.0 mmol, 3.00 equiv) were added. The flask was cooled in a dry ice bath with ethylene glycol/ethanol (vol:vol = 9:1). Dry ice was replenished constantly to maintain the temperature around –20 °C. The reaction mixture was irradiated under white LEDs for 3 h (monitored by TLC until **TT-1** was fully consumed). Thianthrene (953 mg, 63%) could be recovered by simple filtration and the filtrate was concentrated to dryness under reduced pressure. Water (100 mL) was added and extracted by ethyl acetate (2 x 100 mL). The organic layer was washed by water (100 mL) and brine (100 mL), dried over Na_2SO_4, and concentrated under reduced pressure. The resulting residue was purified by flash column chromatography on silica gel (pure hexanes) to give additional thianthrene (450 mg, 30%), then eluted with hexanes/EtOAc = 20:1 to afford (±)-**1** as a light yellow liquid in 63% yield (1.28 g).

(±)-2-Bromo-arylpropanoate 2

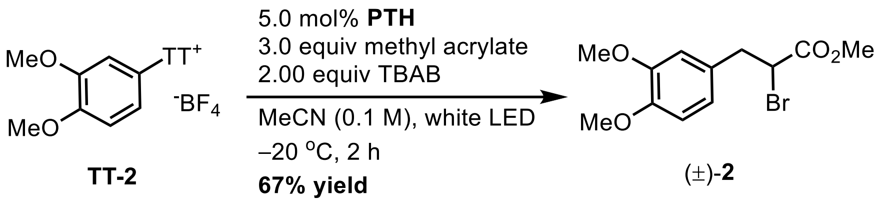

To a 4-mL borosilicate vial containing a Teflon-coated magnetic stirring bar were added **TT-2** (88.0 mg, 0.200 mmol, 1.00 equiv), **PTH** (3.2 mg, 10 μmol, 5.0 mol%), and tetrabutylammonium bromide (TBAB, 128 mg, 0.400 mmol, 2.00 equiv). The vial was transferred into a nitrogen-filled glovebox. Dry MeCN (2 mL, c = 0.1 M) and methyl acrylate (54 μL, 52 mg, 0.60 mmol, 3.0 equiv) were added. The vial was sealed with a Teflon-lined screw cap, removed from the glovebox, and transferred to a cryocooler precooled at –20 °C where the reaction mixture was stirred for 5 min without irradiation and then irradiated with white LEDs for 2 h. Subsequently, silica gel (~5 mL) was added and the reaction mixture was concentrated to dryness under reduced pressure. The residue was purified by flash column chromatography on silica gel (hexanes/EtOAc = 10:1) to afford (±)-**2** as a light yellow oil in 67% yield (40.6 mg).

R*f* = 0.30 (hexanes/EtOAc = 5:1).

NMR Spectroscopy:

^{1}H NMR (500 MHz, $CDCl_3$, 23 °C, δ): 6.82 (d, *J* = 8.1 Hz, 1H), 6.77 (dd, *J* = 8.1, 1.9 Hz, 1H), 6.74 (d, *J* = 2.0 Hz, 1H), 4.39 (dd, *J* = 8.7, 6.8 Hz, 1H), 3.88 (s, 3H), 3.87 (s, 3H), 3.74 (d, *J* = 1.3 Hz, 3H), 3.42 (dd, *J* = 14.1, 8.7 Hz, 1H), 3.20 (dd, *J* = 14.1, 6.7 Hz, 1H).

^{13}C NMR (125 MHz, $CDCl_3$, 23 °C, δ): 170.0, 148.9, 148.3, 129.2, 121.4, 112.3, 111.2, 55.9, 55.9, 52.9, 45.2, 40.8.

HRMS-EI (m/z) calc'd for $C_{12}H_{15}O_4Br$ $[M]^+$, 302.0148; found, 302.0146; deviation: 0.6 ppm.

(±)-2-Bromo-arylpropanoate 3

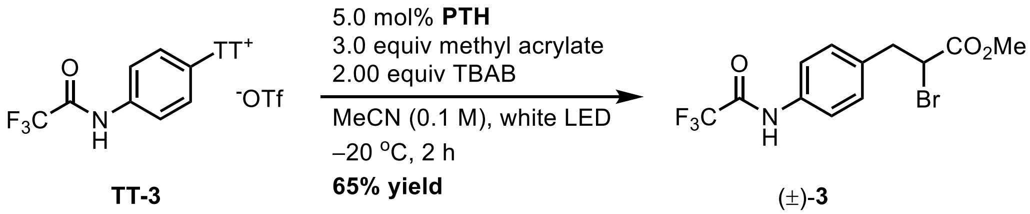

To a 4-mL borosilicate vial containing a Teflon-coated magnetic stirring bar were added **TT-3** (98.0 mg, 0.200 mmol, 1.00 equiv), **PTH** (3.2 mg, 10 μmol, 5.0 mol%), and tetrabutylammonium bromide (TBAB, 128 mg, 0.400 mmol, 2.00 equiv). The vial was transferred into a nitrogen-filled glovebox. Dry MeCN (2 mL, c = 0.1 M) and methyl acrylate (54 μL, 52 mg, 0.60 mmol, 3.0 equiv) were added. The vial was sealed with a Teflon-lined screw cap, removed from the glovebox, and transferred to a cryocooler precooled at –20 °C where the reaction mixture was stirred for 5 min and irradiated by white LEDs for 2 h. Subsequently, silica gel (~5 mL) was added, and the

reaction mixture was concentrated to dryness under reduced pressure. The residue was purified by flash column chromatography on silica gel (hexanes/EtOAc = 5:1) to afford (±)-**3** as a light yellow oil in 65% yield (46.0 mg).

R*f* = 0.20 (hexanes/EtOAc = 5:1).

NMR Spectroscopy:

^{1}H NMR (500 MHz, $CDCl_3$, 23 °C, δ): 7.92 (s, 1H), 7.45 (d, *J* = 8.5 Hz, 2H), 7.17 (d, *J* = 8.5 Hz, 2H), 4.31 (dd, *J* = 8.2, 7.2 Hz, 1H), 3.66 (s, 3H), 3.38 (dd, *J* = 14.2, 8.2 Hz, 1H), 3.17 (dd, *J* = 14.2, 7.2 Hz, 1H).

^{13}C NMR (125 MHz, $CDCl_3$, 23 °C, δ): 169.8, 154.8 (q, *J* = 37.2 Hz), 134.9, 134.3, 130.2, 120.7, 115.7 (q, *J* = 288.5 Hz), 53.0, 44.7, 40.4.

^{19}F NMR (470 MHz, $CDCl_3$, 23 °C, δ): –75.7.

HRMS-ESIpos (m/z) calc'd for $C_{12}H_{11}NO_3BrF_3Na$ $[M+Na]^+$, 375.9767; found, 375.9769; deviation: –0.5 ppm.

(±)-2-Bromo-biarylpropanoate 4

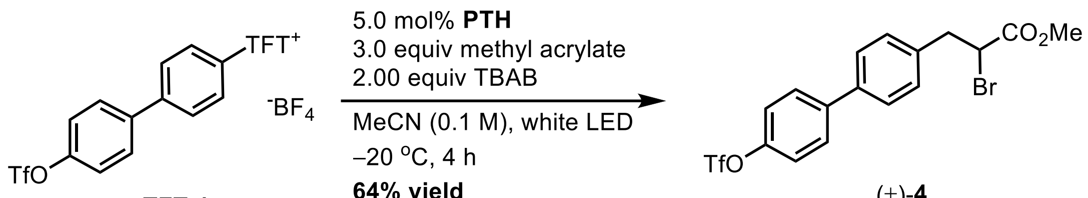

TFT-4 (±)-**4**

To a 4-mL borosilicate vial containing a Teflon-coated magnetic stirring bar were added **TFT-4** (135 mg, 0.200 mmol, 1.00 equiv), **PTH** (3.2 mg, 10 μmol, 5.0 mol%), and tetrabutylammonium bromide (TBAB, 128 mg, 0.400 mmol, 2.00 equiv). The vial was transferred into a nitrogen-filled glovebox. Dry MeCN (2 mL, c = 0.1 M) and methyl acrylate (54 μL, 52 mg, 0.60 mmol, 3.0 equiv) were added. The vial was sealed with a Teflon-lined screw cap, removed from the glovebox, and transferred to a cryocooler precooled at –20 °C where the reaction mixture was stirred for 5 min without irradiation and then irradiated with white LEDs for 4 h. Subsequently, silica gel (~5 mL) was added and the reaction mixture was concentrated to dryness under reduced pressure. The residue was purified by flash column chromatography on silica gel (hexanes/EtOAc = 20:1) to afford (±)-**4** as colorless solid in 64% yield (60.0 mg).

R*f* = 0.37 (hexanes/EtOAc = 10:1).

NMR Spectroscopy:

^{1}H NMR (500 MHz, $CDCl_3$, 23 °C, δ): 7.65 (d, *J* = 8.9 Hz, 2H), 7.53 (d, *J* = 8.2 Hz, 2H), 7.36 (d, *J* = 8.8 Hz, 2H), 7.34 (d, *J* = 8.1 Hz, 2H), 4.47 (dd, *J* = 8.2, 7.1 Hz, 1H), 3.78 (s, 3H), 3.55 (dd, *J* = 14.2, 8.2 Hz, 1H), 3.32 (dd, *J* = 14.2, 7.2 Hz, 1H).

^{13}C NMR (125 MHz, $CDCl_3$, 23 °C, δ): 169.8, 149.0, 141.1, 138.3, 136.7, 129.8, 128.8, 127.4, 121.7, 118.8 (q, J = 320.9 Hz), 53.0, 44.8, 40.7.

^{19}F NMR (470 MHz, $CDCl_3$, 23 °C, δ): –72.8.

HRMS-ESIpos (m/z) calc'd for $C_{17}H_{14}O_5SBrF_3Na$ $[M+Na]^+$, 488.9590; found, 488.9594; deviation: –0.8 ppm.

(±)-Flurbiprofen derivative 5

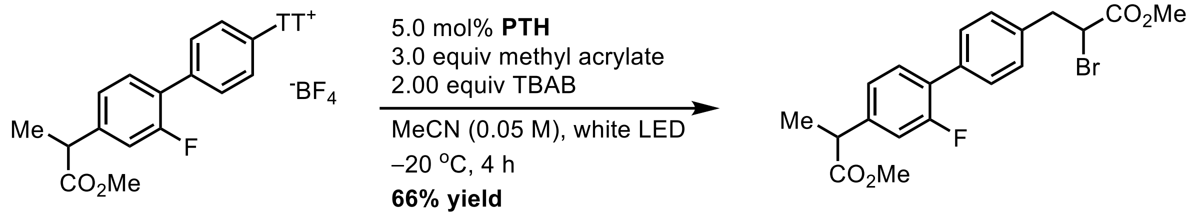

TT-5 (±)-**5**

To a 4-mL borosilicate vial containing a Teflon-coated magnetic stirring bar were added **TT-5** (55.8 mg, 0.100 mmol, 1.00 equiv), **PTH** (1.6 mg, 5.0 μmol, 5.0 mol%), and tetrabutylammonium bromide (TBAB, 64 mg, 0.40 mmol, 2.0 equiv). The vial was transferred into a nitrogen-filled glovebox. Dry MeCN (2 mL, c = 0.05 M) and methyl acrylate (27 μL, 26 mg, 0.30 mmol, 3.0 equiv) were added. The vial was sealed with a Teflon-lined screw cap, removed from the glovebox, and transferred to a cryocooler precooled at –20 °C where the reaction mixture was stirred for 5 min without irradiation and then irradiated with white LEDs for 4 h. Subsequently, silica gel (~5 mL) was added and the reaction mixture was concentrated to dryness under reduced pressure. The residue was purified by flash column chromatography on silica gel (hexanes/EtOAc = 20:1) to afford (±)-**5** as a colorless oil in 66% yield (27.9 mg).

$\mathbf{R}_f$ = 0.28 (hexanes/EtOAc = 20:1).

NMR Spectroscopy:

^{1}H NMR (500 MHz, $CDCl_3$, 23 °C, δ): 7.51 (dd, *J* = 8.2, 1.7 Hz, 2H), 7.40 (t, *J* = 8.0 Hz, 1H), 7.30 (d, *J* = 8.2 Hz, 2H), 7.19 – 7.11 (m, 2H), 4.47 (dd, *J* = 8.4, 7.0 Hz, 1H), 3.81 – 3.75 (m, 4H), 3.72 (s, 3H), 3.54 (dd, *J* = 14.1, 8.3 Hz, 1H), 3.31 (dd, *J* = 14.2, 7.0 Hz, 1H), 1.56 (d, *J* = 7.2 Hz, 3H).

^{13}C NMR (125 MHz, $CDCl_3$, 23 °C, δ): 174.4, 169.8, 159.7 (d, *J* = 248.2 Hz), 141.9 (d, *J* = 7.7 Hz), 136.2, 134.6, 130.7 (d, *J* = 4.0 Hz), 129.3, 129.2 (d, *J* = 2.9 Hz), 127.3 (d, *J* = 13.6 Hz), 123.6 (d, *J* = 3.5 Hz), 115.3 (d, *J* = 23.4 Hz), 53.0, 52.2, 44.9, 44.9, 40.8, 18.4.

^{19}F NMR (470 MHz, $CDCl_3$, 23 °C, δ): –117.3 – –117.6 (m).

HRMS-ESIpos (m/z) calc'd for $C_{20}H_{20}O_4FBrNa$ $[M+Na]^+$, 445.0421; found, 445.0419; deviation: 0.6 ppm.

(±)-Methyl meclofenamate derivative 6

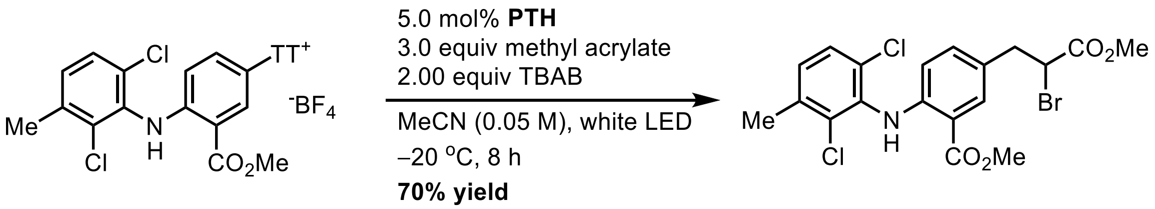

TT-6 (±)-**6**

To a 4-mL borosilicate vial containing a Teflon-coated magnetic stirring bar were added **TT-6** (63.2 mg, 0.100 mmol, 1.00 equiv), **PTH** (1.6 mg, 5.0 μmol, 5.0 mol%), and tetrabutylammonium bromide (TBAB, 64 mg, 0.40 mmol, 2.0 equiv). The vial was transferred into a nitrogen-filled glovebox. Dry MeCN (2 mL, c = 0.05 M) and methyl acrylate (27 μL, 26 mg, 0.30 mmol, 3.0 equiv) were added. The vial was sealed with a Teflon-lined screw cap, removed from the glovebox, and transferred to a cryocooler precooled at –20 °C where the reaction mixture was stirred for 5 min without irradiation and then irradiated with white LEDs for 8 h. Subsequently, silica gel (~5 mL) was added and the reaction mixture was concentrated to dryness under reduced pressure. The residue was purified by flash column chromatography on silica gel (hexanes/EtOAc = 30:1) to afford (±)-**6** as a colorless oil in 70% yield (34.6 mg).

R*f* = 0.18 (hexanes/EtOAc = 20:1).

NMR Spectroscopy:

^{1}H NMR (500 MHz, $CDCl_3$, 23 ºC, δ): 9.24 (s, 1H), 7.77 (d, *J* = 2.2 Hz, 1H), 7.23 (d, *J* = 8.2 Hz, 1H), 7.09 – 6.98 (m, 2H), 6.19 (d, *J* = 8.6 Hz, 1H), 4.27 (dd, *J* = 8.6, 6.8 Hz, 1H), 3.86 (s, 3H), 3.65 (s, 3H), 3.29 (dd, *J* = 14.2, 8.6 Hz, 1H), 3.08 (dd, *J* = 14.2, 6.8 Hz, 1H), 2.33 (d, *J* = 0.8 Hz, 3H).

^{13}C NMR (125 MHz, $CDCl_3$, 23 ºC, δ): 169.9, 168.6, 146.7, 136.5, 135.0, 134.9, 134.2, 131.9, 131.1, 128.6, 127.8, 125.4, 114.1, 111.6, 52.9, 51.9, 45.2, 40.2, 20.6.

HRMS-EESIpos (m/z) calc'd for $C_{19}H_{18}BrCl_2NO_4Na$ $[M+Na]^+$, 495.9689; found, 495.9694; deviation: –1.1 ppm.

(±)-2-Bromo-heteroarylpropanoate 7

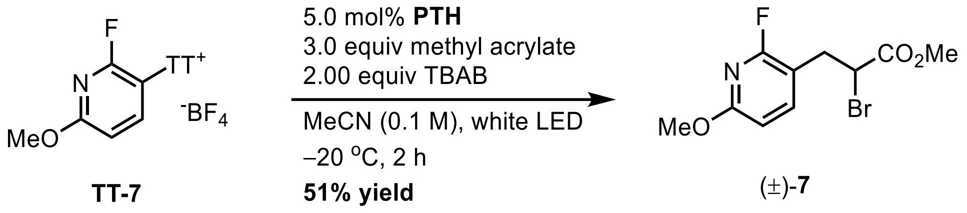

To a 4-mL borosilicate vial containing a Teflon-coated magnetic stirring bar were added **TT-7** (85.4 mg, 0.200 mmol, 1.00 equiv), **PTH** (3.2 mg, 10 μmol, 5.0 mol%), and tetrabutylammonium bromide (TBAB, 128 mg, 0.400 mmol, 2.00 equiv). The vial was transferred into a nitrogen-filled glovebox. Dry MeCN (2 mL, c = 0.1 M) and methyl acrylate (54 μL, 52 mg, 0.60 mmol, 3.0 equiv) were added. The vial was sealed with a Teflon-lined screw cap, removed from the glovebox, and transferred to a cryocooler precooled at –20 °C where the reaction mixture was stirred for 5 min without irradiation and then irradiated with white LEDs for 2 h. Subsequently, silica gel (~5 mL) was added and the reaction mixture was concentrated to dryness under reduced pressure. The residue was purified by flash column chromatography on silica gel (hexanes/EtOAc = 40:1) to afford (±)-**7** as a light yellow oil in 51% yield (29.5 mg).

R*f* = 0.20 (hexanes/EtOAc = 30:1).

NMR Spectroscopy:

^{1}H NMR (500 MHz, $CDCl_3$, 23 ºC, δ): 7.55 (t, *J* = 7.9 Hz, 1H), 6.44 (dd, *J* = 7.8, 2.8 Hz, 1H), 4.56 (t, *J* = 7.6 Hz, 1H), 3.97 (s, 3H), 3.76 (s, 3H), 3.38 (dd, *J* = 14.1, 7.7 Hz, 1H), 3.21 (dd, *J* = 14.1, 7.5 Hz, 1H).

^{13}C NMR (125 MHz, $CDCl_3$, 23 ºC, δ): 169.8, 161.6 (d, *J* = 240.7 Hz), 160.8 (d, *J* = 13.8 Hz), 144.0 (d, *J* = 8.1 Hz), 115.4 (d, *J* = 5.6 Hz), 99.9 (d, *J* = 35.6 Hz), 54.1, 52.9, 42.7 (d, *J* = 1.9 Hz), 35.1.

^{19}F NMR (470 MHz, $CDCl_3$, 23 ºC, δ): –72.1.

HRMS-EI (m/z) calc'd for $C_{10}H_{11}NO_3FBr$ $[M]^+$, 290.9901; found, 290.9903; deviation: –0.6 ppm.

(±)-2-Bromo-arylpropanoate 8

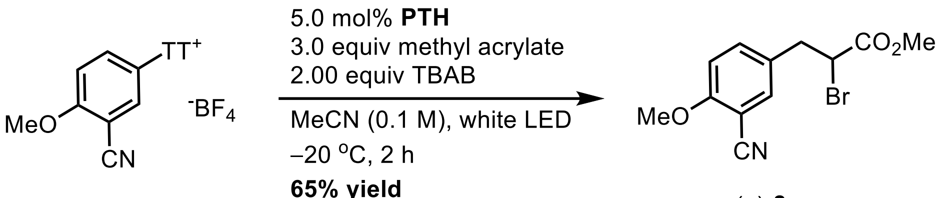

TT-8 (±)-**8**

To a 4-mL borosilicate vial containing a Teflon-coated magnetic stirring bar were added **TT-8** (86.6 mg, 0.200 mmol, 1.00 equiv), **PTH** (3.2 mg, 10 μmol, 5.0 mol%), and tetrabutylammonium bromide (TBAB, 128 mg, 0.400 mmol, 2.00 equiv). The vial was transferred into a nitrogen-filled glovebox. Dry MeCN (2 mL, c = 0.1 M) and methyl acrylate (54 μL, 52 mg, 0.60 mmol, 3.0 equiv) were added. The vial was sealed with a Teflon-lined screw cap, removed from the glovebox, and transferred to a cryocooler precooled at –20 °C where the reaction mixture was stirred for 5 min without irradiation and then irradiated with white LEDs for 2 h. Subsequently, silica gel (~5 mL) was added and the reaction mixture was concentrated to dryness under reduced pressure. The residue was purified by flash column chromatography on silica gel (hexanes/EtOAc = 5:1) to afford (±)-**8** as a colorless oil in 65% yield (38.6 mg).

R*f* = 0.17 (hexanes/EtOAc = 5:1).

NMR Spectroscopy:

^{1}H NMR (500 MHz, $CDCl_3$, 23 ºC, δ): 7.45 – 7.39 (m, 2H), 6.94 (d, *J* = 8.5 Hz, 1H), 4.35 (t, *J* = 7.6 Hz, 1H), 3.94 (s, 3H), 3.77 (s, 3H), 3.42 (dd, *J* = 14.4, 7.9 Hz, 1H), 3.21 (dd, *J* = 14.4, 7.3 Hz, 1H).

^{13}C NMR (125 MHz, $CDCl_3$, 23 ºC, δ): 169.5, 160.5, 135.4, 134.3, 129.3, 116.1, 111.6, 102.0, 56.1, 53.1, 44.6, 39.6.

HRMS-EI (m/z) calc'd for $C_{12}H_{12}NO_3Br$ $[M]^+$, 296.9995; found, 296.9995; deviation: 0.2 ppm.

(±)-2-Bromo-arylpropanoate 9

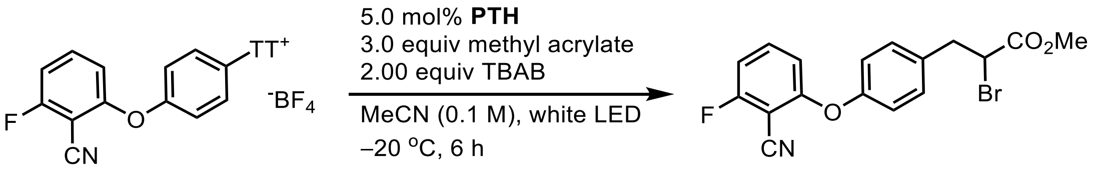

TT-9 (±)-**9**

To a 4-mL borosilicate vial containing a Teflon-coated magnetic stirring bar were added **TT-9** (103 mg, 0.200 mmol, 1.00 equiv), **PTH** (3.2 mg, 10 μmol, 5.0 mol%), and tetrabutylammonium bromide (TBAB, 128 mg, 0.400 mmol, 2.00 equiv). The vial was transferred into a nitrogen-filled glovebox. Dry MeCN (2 mL, c = 0.1 M) and methyl acrylate (54 μL, 52 mg, 0.60 mmol, 3.0 equiv) were added. The vial was sealed with a Teflon-lined screw cap, removed from the glovebox, and transferred to a cryocooler precooled at –20 °C where the reaction mixture was stirred for 5 min without irradiation and then irradiated with white LEDs for 6 h. Subsequently, silica gel (~5 mL) was added and the reaction mixture was concentrated to dryness under reduced pressure. The residue was purified by flash column chromatography on silica gel (hexanes/EtOAc = 5:1) to afford (±)-**9** as a colorless oil in 57% yield (42.9 mg).

R*f* = 0.23 (hexanes/EtOAc = 5:1).

NMR Spectroscopy:

^{1}H NMR (500 MHz, $CDCl_3$, 23 °C, δ): 7.45 (td, *J* = 8.5, 6.4 Hz, 1H), 7.29 (d, *J* = 8.4 Hz, 2H), 7.07 (d, *J* = 8.6 Hz, 2H), 6.91 (td, *J* = 8.4, 0.8 Hz, 1H), 6.63 (dt, *J* = 8.6, 0.8 Hz, 1H), 4.42 (dd, *J* = 8.2, 7.2 Hz, 1H), 3.78 (s, 3H), 3.50 (dd, *J* = 14.2, 8.1 Hz, 1H), 3.28 (dd, *J* = 14.2, 7.2 Hz, 1H).

^{13}C NMR (125 MHz, $CDCl_3$, 23 °C, δ): 169.7, 164.1 (d, *J* = 259.8 Hz), 160.8 (d, *J* = 4.2 Hz), 153.7, 134.9 (d, *J* = 10.1 Hz), 131.1, 120.5, 112.0, 111.9, 111.0, 109.8 (d, *J* = 19.2 Hz), 93.8 (d, *J* = 18.0 Hz), 53.1, 44.8, 40.4.

^{19}F NMR (470 MHz, $CDCl_3$, 23 °C, δ): –104.6 (t, *J* = 7.4 Hz).

HRMS-CI (m/z) calc'd for $C_{17}H_{14}NO_3FBr$ $[M+H]^+$, 378.0136; found, 378.0142; deviation: –1.7 ppm.

(±)-Diclofenac amide derivative 10

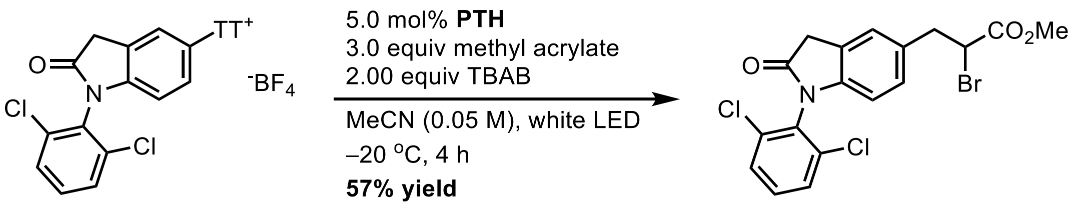

TT-10 (±)-**10**

To a 4-mL borosilicate vial containing a Teflon-coated magnetic stirring bar were added **TT-10** (58.0 mg, 0.100 mmol, 1.00 equiv), **PTH** (1.6 mg, 5.0 μmol, 5.0 mol%), and tetrabutylammonium bromide (TBAB, 64 mg, 0.40 mmol, 2.0 equiv). The vial was transferred into a nitrogen-filled glovebox. Dry MeCN (2 mL, c = 0.05 M) and

methyl acrylate (27 µL, 26 mg, 0.30 mmol, 3.0 equiv) were added. The vial was sealed with a Teflon-lined screw cap, removed from the glovebox, and transferred to a cryocooler precooled at –20 °C where the reaction mixture was stirred for 5 min without irradiation and then irradiated with white LEDs for 4 h. Subsequently, silica gel (~5 mL) was added and the reaction mixture was concentrated to dryness under reduced pressure. The residue was purified by flash column chromatography on silica gel (hexanes/EtOAc = 4:1) to afford (±)-**10** as a colorless oil in 57% yield (25.2 mg).

R*f* = 0.20 (hexanes/EtOAc = 4:1).

NMR Spectroscopy:

^{1}**H NMR** (500 MHz, $CDCl_3$, 23 °C, δ): 7.53 (d, *J* = 8.2 Hz, 2H), 7.40 (dd, *J* = 8.6, 7.7 Hz, 1H), 7.23 (s, 1H), 7.07 (d, *J* = 8.0 Hz, 1H), 6.36 (d, *J* = 8.0 Hz, 1H), 4.40 (dd, *J* = 8.4, 6.9 Hz, 1H), 3.78 (s, 2H), 3.77 (s, 3H), 3.48 (dd, *J* = 14.2, 8.4 Hz, 1H), 3.25 (dd, *J* = 14.3, 6.9 Hz, 1H).

^{13}**C NMR** (125 MHz, $CDCl_3$, 23 °C, δ): 173.4, 169.8, 142.6, 135.4, 131.5, 130.8, 130.4, 129.0, 128.9, 125.8, 124.7, 109.2, 53.0, 45.1, 40.8, 35.7.

HRMS-ESIpos (m/z) calc'd for $C_{18}H_{14}BrCl_2NO_3Na$ $[M+Na]^+$, 463.9426; found, 463.9432; deviation: –1.2 ppm.

(±)-*N*-Methyl-nimesulide derivative 11

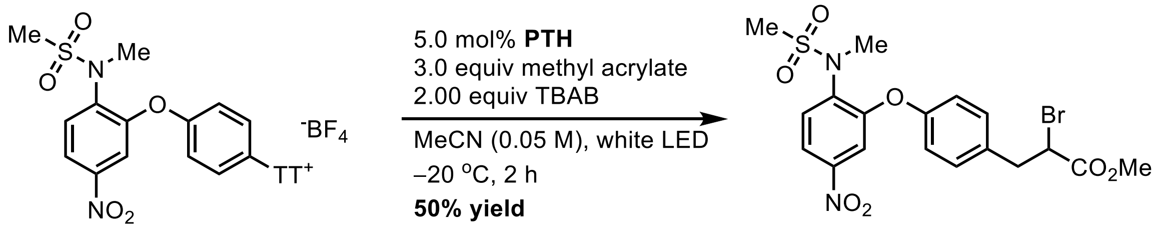

TT-11 (±)-**11**

To a 4-mL borosilicate vial containing a Teflon-coated magnetic stirring bar were added **TT-11** (62.2 mg, 0.100 mmol, 1.00 equiv), **PTH** (1.6 mg, 5.0 µmol, 5.0 mol%), and tetrabutylammonium bromide (TBAB, 64 mg, 0.40 mmol, 2.0 equiv). The vial was transferred into a nitrogen-filled glovebox. Dry MeCN (2 mL, c = 0.05 M) and methyl acrylate (27 µL, 26 mg, 0.30 mmol, 3.0 equiv) were added. The vial was sealed with a Teflon-lined screw cap, removed from the glovebox, and transferred to a cryocooler precooled at –20 °C where the reaction mixture was stirred for 5 min without irradiation and then irradiated with white LEDs for 2 h. Subsequently, silica gel (~5 mL) was added and the reaction mixture was concentrated to dryness under reduced pressure. The residue was purified by flash column chromatography on silica gel (hexanes/EtOAc = 3:1) to afford (±)-**11** as a light yellow oil in 50% yield (24.3 mg).

R*f* = 0.13 (hexanes/EtOAc = 3:1).

NMR Spectroscopy:

^{1}**H NMR** (500 MHz, $CDCl_3$, 23 °C, δ): 7.88 (dd, *J* = 8.8, 2.5 Hz, 1H), 7.55 (dd, *J* = 5.6, 3.1 Hz, 2H), 7.24 (d, *J* = 8.5 Hz, 2H), 6.97 (d, *J* = 8.5 Hz, 2H), 4.35 (dd, *J* = 8.1, 7.2 Hz, 1H), 3.70 (s, 3H), 3.43 (dd, *J* = 14.2, 8.1 Hz,

1H), 3.29 (s, 3H), 3.22 (dd, *J* = 14.2, 7.2 Hz, 1H), 2.93 (s, 3H).

^{13}C NMR (125 MHz, $CDCl_3$, 23 °C, δ): 169.7, 154.8, 153.7, 147.8, 136.8, 134.4, 132.6, 131.6, 119.9, 118.3, 112.8, 53.1, 44.8, 40.3, 38.7, 37.8.

HRMS-ESIpos (m/z) calc'd for $C_{18}H_{19}N_2O_7SBrNa$ [M+Na]$^+$, 508.9989; found, 508.9990; deviation: –0.3 ppm.

(±)-2-Bromo-arylpropanoate 12

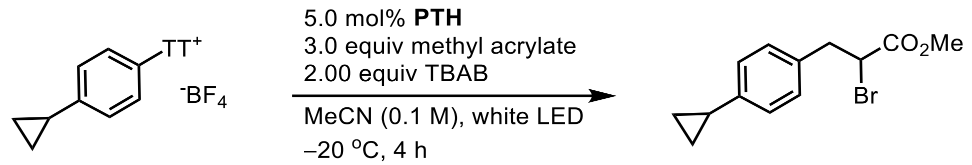

TT-12 (±)-**12**

To a 4-mL borosilicate vial containing a Teflon-coated magnetic stirring bar were added **TT-12** (83.4 mg, 0.200 mmol, 1.00 equiv), **PTH** (3.2 mg, 10 μmol, 5.0 mol%), and tetrabutylammonium bromide (TBAB, 128 mg, 0.400 mmol, 2.00 equiv). The vial was transferred into a nitrogen-filled glovebox. Dry MeCN (2 mL, c = 0.1 M) and methyl acrylate (54 μL, 52 mg, 0.60 mmol, 3.0 equiv) were added. The vial was sealed with a Teflon-lined screw cap, removed from the glovebox, and transferred to a cryocooler precooled at –20 °C where the reaction mixture was stirred for 5 min without irradiation and then irradiated with white LEDs for 4 h. Subsequently, silica gel (~5 mL) was added and the reaction mixture was concentrated to dryness under reduced pressure. The residue was purified by flash column chromatography on silica gel (hexanes/EtOAc = 40:1) to afford (±)-**12** as a colorless oil in 61% yield (34.0mg).

R*f* = 0.20 (hexanes/EtOAc = 40:1).

NMR Spectroscopy:

^{1}H NMR (500 MHz, $CDCl_3$, 23 °C, δ): 7.01 (d, *J* = 8.1 Hz, 2H), 6.93 (d, *J* = 8.1 Hz, 2H), 4.29 (dd, *J* = 8.6, 6.9 Hz, 1H), 3.65 (s, 3H), 3.34 (dd, *J* = 14.1, 8.5 Hz, 1H), 3.12 (dd, *J* = 14.2, 6.9 Hz, 1H), 1.79 (tt, *J* = 8.4, 5.1 Hz, 1H), 1.01 – 0.76 (m, 2H), 0.63 – 0.56 (m, 2H).

^{13}C NMR (125 MHz, $CDCl_3$, 23 °C, δ): 169.9, 143.2, 133.6, 129.1, 125.9, 52.9, 45.2, 40.7, 15.1, 9.3.

HRMS-EI (m/z) calc'd for $C_{13}H_{15}O_2Br$ [M]$^+$, 282.0250; found, 282.0248; deviation: 0.7 ppm.

(±)-2-Bromo-arylpropanoate 13

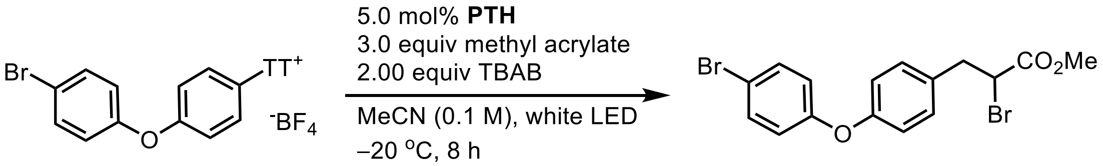

TT-13 (±)-**13**

To a 4-mL borosilicate vial containing a Teflon-coated magnetic stirring bar were added **TT-13** (110 mg, 0.200

mmol, 1.00 equiv), **PTH** (3.2 mg, 10 μmol, 5.0 mol%), and tetrabutylammonium bromide (TBAB, 128 mg, 0.400 mmol, 2.00 equiv). The vial was transferred into a nitrogen-filled glovebox. Dry MeCN (2 mL, c = 0.1 M) and methyl acrylate (54 μL, 52 mg, 0.60 mmol, 3.0 equiv) were added. The vial was sealed with a Teflon-lined screw cap, removed from the glovebox, and transferred to a cryocooler precooled at –20 °C where the reaction mixture was stirred for 5 min without irradiation and then irradiated with white LEDs for 8 h. Subsequently, silica gel (~5 mL) was added and the reaction mixture was concentrated to dryness under reduced pressure. The residue was purified by flash column chromatography on silica gel (hexanes/EtOAc = 50:1) to afford (±)-1**3** as a colorless oil in 54% yield (44.8 mg).

R*f* = 0.18 (hexanes/EtOAc = 50:1).

NMR Spectroscopy:

^{1}**H NMR** (500 MHz, $CDCl_3$, 23 °C, δ): 7.45 (d, *J* = 9.0 Hz, 2H), 7.20 (d, *J* = 8.6 Hz, 2H), 6.96 (d, *J* = 8.6 Hz, 2H), 6.90 (d, *J* = 8.9 Hz, 2H), 4.40 (dd, *J* = 8.4, 7.1 Hz, 1H), 3.77 (s, 3H), 3.47 (dd, *J* = 14.2, 8.4 Hz, 1H), 3.25 (dd, *J* = 14.2, 7.1 Hz, 1H).

^{13}**C NMR** (125 MHz, $CDCl_3$, 23 °C, δ): 169.8, 156.3, 156.1, 132.7, 132.0, 130.7, 120.6, 119.0, 115.9, 53.0, 45.1, 40.4.

HRMS-EI (m/z) calc'd for $C_{16}H_{14}O_3Br_2$ $[M+H]^+$, 411.9304; found, 411.9309; deviation: –1.2 ppm.

(±)-2-Bromo-arylpropanoate 14

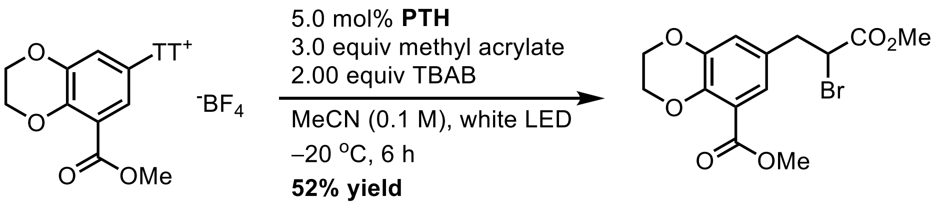

TT-14 (±)-**14**

To a 4-mL borosilicate vial containing a Teflon-coated magnetic stirring bar were added **TT-14** (96.0 mg, 0.200 mmol, 1.00 equiv), **PTH** (3.2 mg, 10 μmol, 5.0 mol%), and tetrabutylammonium bromide (TBAB, 128 mg, 0.400 mmol, 2.00 equiv). The vial was transferred into a nitrogen-filled glovebox. Dry MeCN (2 mL, c = 0.1 M) and methyl acrylate (54 μL, 52 mg, 0.60 mmol, 3.0 equiv) were added. The vial was sealed with a Teflon-lined screw cap, removed from the glovebox, and transferred to a cryocooler precooled at –20 °C where the reaction mixture was stirred for 5 min without irradiation and then irradiated with white LEDs for 6 h. Subsequently, silica gel (~5 mL) was added and the reaction mixture was concentrated to dryness under reduced pressure. The residue was purified by flash column chromatography on silica gel (hexanes/EtOAc = 3:1) to afford (±)-1**4** as a colorless oil in 52% yield (35.6 mg).

R*f* = 0.17 (hexanes/EtOAc = 3:1).

NMR Spectroscopy:

^{1}**H NMR** (500 MHz, $CDCl_3$, 23 °C, δ): 7.18 (d, *J* = 2.3 Hz, 1H), 6.82 (d, *J* = 2.2 Hz, 1H), 4.31 – 4.25 (m, 3H),

4.23 – 4.19 (m, 2H), 3.81 (s, 3H), 3.68 (s, 3H), 3.29 (dd, *J* = 14.3, 8.2 Hz, 1H), 3.07 (dd, *J* = 14.2, 7.1 Hz, 1H).

^{13}C NMR (125 MHz, $CDCl_3$, 23 ºC, δ): 169.6, 165.7, 144.0, 143.3, 128.9, 124.2, 121.9, 119.9, 64.6, 63.8, 53.0, 52.1, 44.8, 40.1.

HRMS-ESIpos (m/z) calc'd for $C_{14}H_{16}O_6Br$ $[M+H]^+$, 359.0125; found, 359.0124; deviation: 0.2 ppm.

(±)-Boscalid derivative 15

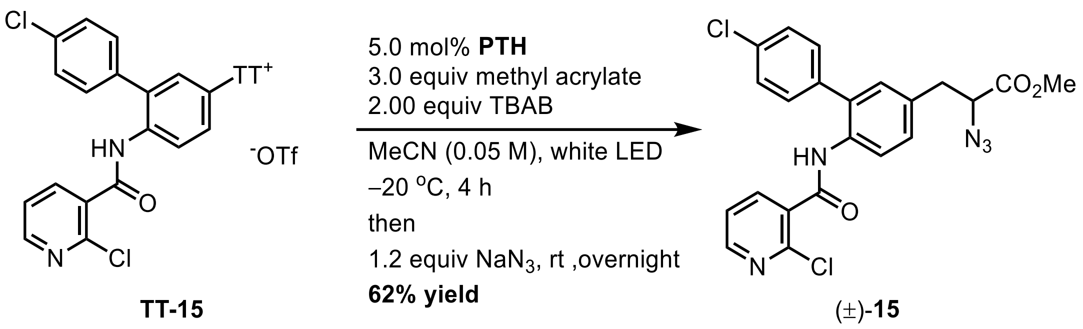

To a 4-mL borosilicate vial containing a Teflon-coated magnetic stirring bar were added **TT-15** (70.7 mg, 0.100 mmol, 1.00 equiv), **PTH** (1.6 mg, 5.0 μmol, 5.0 mol%), and tetrabutylammonium bromide (TBAB, 64 mg, 0.40 mmol, 2.0 equiv). The vial was transferred into a nitrogen-filled glovebox. Dry MeCN (2 mL, c = 0.05 M) and methyl acrylate (27 μL, 26 mg, 0.30 mmol, 3.0 equiv) were added. The vial was sealed with a Teflon-lined screw cap, removed from the glovebox, and transferred to a cryocooler precooled at –20 °C where the reaction mixture was stirred for 5 min without irradiation and then irradiated with white LEDs for 4 h. The vial was warmed to room temperature and NaN_3 (7.8 mg, 0.12 mmol, 1.2 equiv) was added. The reaction mixture was stirred overnight. Subsequently, silica gel (~5 mL) was added and the reaction mixture was concentrated to dryness under reduced pressure. The residue was purified by flash column chromatography on silica gel (hexanes/EtOAc = 2:1) to afford (±)-**15** as a colorless solid in 62% yield (29.0 mg).

R*f* = 0.22 (hexanes/EtOAc = 2:1).

NMR Spectroscopy:

^{1}H NMR (500 MHz, $CDCl_3$, 23 ºC, δ): 8.39 (dd, *J* = 4.7, 2.0 Hz, 1H), 8.34 (d, *J* = 8.4 Hz, 1H), 8.12 – 8.04 (m, 2H), 7.38 (d, *J* = 8.4 Hz, 2H), 7.33 – 7.23 (m, 4H), 7.07 (d, *J* = 2.1 Hz, 1H), 4.05 (dd, *J* = 8.7, 5.2 Hz, 1H), 3.74 (s, 3H), 3.14 (dd, *J* = 14.1, 5.2 Hz, 1H), 2.96 (dd, *J* = 14.1, 8.7 Hz, 1H).

^{13}C NMR (125 MHz, $CDCl_3$, 23 ºC, δ): 170.2, 162.4, 151.4, 146.7, 140.3, 135.9, 134.7, 133.6, 133.0, 132.4, 131.0, 130.9, 130.8, 129.6, 129.4, 123.0, 122.2, 63.1, 52.8, 37.1.

HRMS-ESIpos (m/z) calc'd for $C_{22}H_{17}N_5O_3Cl_2Na$ $[M+Na]^+$, 492.0601; found, 492.0604; deviation: –0.6 ppm.

(±)-Etofenprox derivative 16

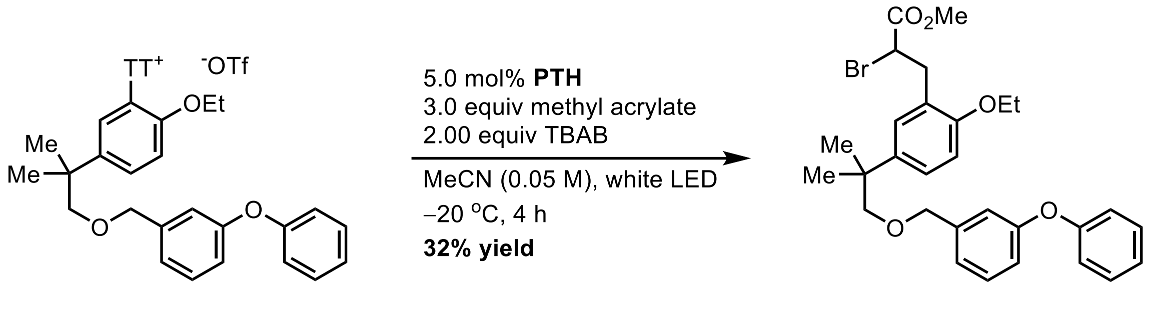

TT-16 (±)-**16**

To a 4-mL borosilicate vial containing a Teflon-coated magnetic stirring bar were added **TT-16** (67.7 mg, 0.100 mmol, 1.00 equiv), **PTH** (1.6 mg, 5.0 μmol, 5.0 mol%), and tetrabutylammonium bromide (TBAB, 64 mg, 0.40 mmol, 2.0 equiv). The vial was transferred into a nitrogen-filled glovebox. Dry MeCN (2 mL, c = 0.05 M) and methyl acrylate (27 μL, 26 mg, 0.30 mmol, 3.0 equiv) were added. The vial was sealed with a Teflon-lined screw cap, removed from the glovebox, and transferred to a cryocooler precooled at –20 °C where the reaction mixture was stirred for 5 min without irradiation and then irradiated with white LEDs for 4 h. Subsequently, silica gel (~5 mL) was added and the reaction mixture was concentrated to dryness under reduced pressure. The residue was purified by flash column chromatography on silica gel (hexanes/EtOAc = 40:1) to afford (±)-**16** as a colorless oil in 32% yield (17.3 mg).

R*f* = 0.30 (hexanes/EtOAc = 20:1).

NMR Spectroscopy:

^{1}H NMR (500 MHz, $CDCl_3$, 23 °C, δ): 7.34 (dd, *J* = 8.6, 7.4 Hz, 2H), 7.26 (t, *J* = 7.8 Hz, 1H), 7.20 (dd, *J* = 8.5, 2.5 Hz, 1H), 7.14 (d, *J* = 2.5 Hz, 1H), 7.13 – 7.07 (m, 1H), 7.04 – 6.96 (m, 3H), 6.94 – 6.91 (m, 1H), 6.91 – 6.86 (m, 1H), 6.73 (d, *J* = 8.5 Hz, 1H), 4.59 (dd, *J* = 8.2, 7.0 Hz, 1H), 4.44 (s, 2H), 4.02 (q, *J* = 6.9 Hz, 2H), 3.67 (s, 3H), 3.42 – 3.34 (m, 3H), 3.29 (dd, *J* = 13.6, 7.0 Hz, 1H), 1.41 (t, *J* = 7.0 Hz, 3H), 1.29 (d, *J* = 3.7 Hz, 6H).

^{13}C NMR (125 MHz, $CDCl_3$, 23 °C, δ): 170.3, 157.3, 157.2, 155.0, 141.0, 139.2, 129.7, 129.5, 129.4, 126.1, 124.3, 123.2, 122.0, 119.0, 117.7, 117.6, 110.6, 80.2, 72.8, 63.4, 52.7, 44.0, 38.5, 37.0, 26.2, 26.1, 14.9.

HRMS-ESIpos (m/z) calc'd for $C_{29}H_{33}BrO_5Na$ $[M+Na]^+$, 563.1403; found, 563.1408; deviation: –0.8 ppm.

(±)-2-Bromo-arylpropanoate 17

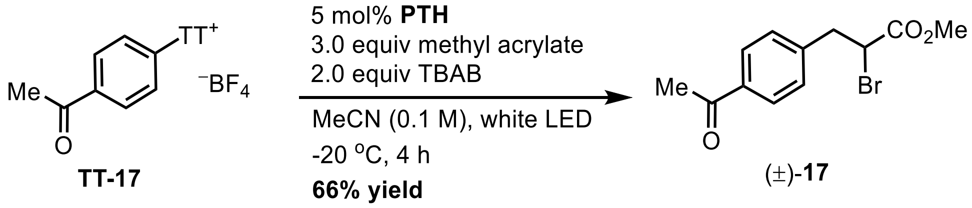

TT-17 (±)-**17**

To a 4-mL borosilicate vial containing a Teflon-coated magnetic stirring bar were added **TT-17** (84.4 mg, 0.200

mmol, 1.00 equiv), **PTH** (3.2 mg, 10 μmol, 5.0 mol%), and tetrabutylammonium bromide (TBAB, 128 mg, 0.400 mmol, 2.00 equiv). The vial was transferred into a nitrogen-filled glovebox. Dry MeCN (2 mL, c = 0.1 M) and methyl acrylate (54 μL, 52 mg, 0.60 mmol, 3.0 equiv) were added. The vial was sealed with a Teflon-lined screw cap, removed from the glovebox, and transferred to a cryocooler precooled at –20 °C where the reaction mixture was stirred for 5 min without irradiation and then irradiated with white LEDs for 4 h. Subsequently, silica gel (~5 mL) was added and the reaction mixture was concentrated to dryness under reduced pressure. The residue was purified by flash column chromatography on silica gel (hexanes/EtOAc = 10:1) to afford (±)-**17** as a colorless oil in 66% yield (37.3 mg).

R*f* = 0.17 (hexanes/EtOAc = 10:1).

NMR Spectroscopy:

^{1}H NMR (500 MHz, $CDCl_3$, 23 °C, δ): 7.84 (d, *J* = 8.3 Hz, 2H), 7.24 (d, *J* = 8.1 Hz, 2H), 4.36 (t, *J* = 7.7 Hz, 1H), 3.67 (s, 3H), 3.45 (dd, *J* = 14.2, 8.0 Hz, 1H), 3.23 (dd, *J* = 14.2, 7.4 Hz, 1H), 2.52 (s, 3H).

^{13}C NMR (125 MHz, $CDCl_3$, 23 °C, δ): 197.6, 169.6, 142.0, 136.2, 129.4, 128.7, 53.1, 44.3, 40.9, 26.6.

HRMS-CI (m/z) calc'd for $C_{12}H_{14}O_3Br$ $[M+H]^+$, 285.0121; found, 285.0124; deviation: –1.0 ppm.

(±)-2-Bromo-arylpropanoate 18

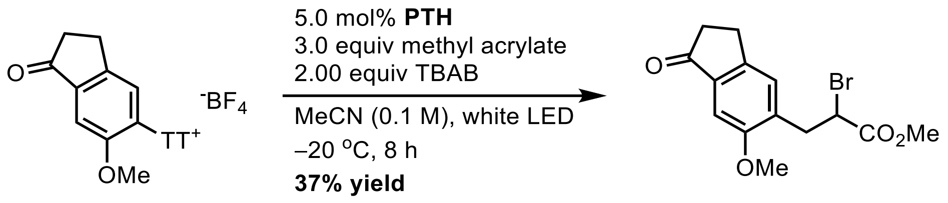

TT-18 (±)-**18**

To a 4-mL borosilicate vial containing a Teflon-coated magnetic stirring bar were added **TT-18** (92.4 mg, 0.200 mmol, 1.00 equiv), **PTH** (3.2 mg, 10 μmol, 5.0 mol%), and tetrabutylammonium bromide (TBAB, 128 mg, 0.400 mmol, 2.00 equiv). The vial was transferred into a nitrogen-filled glovebox. Dry MeCN (2 mL, c = 0.1 M) and methyl acrylate (54 μL, 52 mg, 0.60 mmol, 3.0 equiv) were added. The vial was sealed with a Teflon-lined screw cap, removed from the glovebox, and transferred to a cryocooler precooled at –20 °C where the reaction mixture was stirred for 5 min without irradiation and then irradiated with white LEDs for 8 h. Subsequently, silica gel (~5 mL) was added and the reaction mixture was concentrated to dryness under reduced pressure. The residue was purified by flash column chromatography on silica gel (hexanes/EtOAc = 5:2) to afford (±)-**18** as a colorless solid in 37% yield (23.9 mg).

R*f* = 0.11 (hexanes/EtOAc = 3:1).

NMR Spectroscopy:

^{1}H NMR (500 MHz, $CDCl_3$, 23 °C, δ): 7.58 (s, 1H), 6.91 (s, 1H), 4.58 (t, *J* = 7.6 Hz, 1H), 3.95 (s, 3H), 3.76 (s, 3H), 3.46 (dd, *J* = 14.0, 7.5 Hz, 1H), 3.30 (dd, *J* = 14.0, 7.7 Hz, 1H), 3.14 – 3.04 (m, 2H), 2.74 – 2.63 (m, 2H).

^{13}**C NMR** (125 MHz, $CDCl_3$, 23 °C, δ): 205.0, 169.8, 163.1, 157.7, 130.0, 126.5, 125.5, 107.3, 55.8, 52.9, 43.5, 36.4, 36.3, 25.9.

HRMS-EI (m/z) calc'd for $C_{14}H_{15}O_4Br$ $[M]^+$, 326.0148; found, 326.0150; deviation: –0.4 ppm.

(±)-2-Bromo-arylpropanoate 19

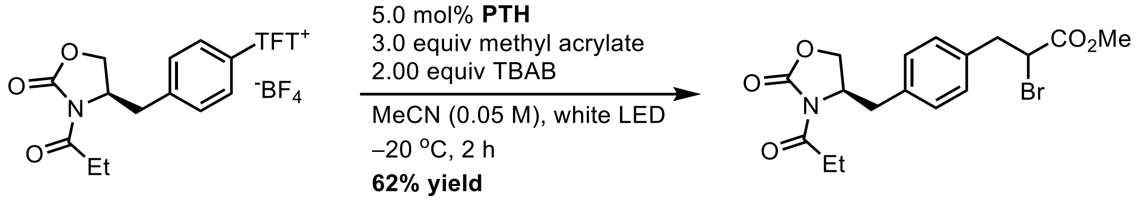

TT-19 (±)-**19**

To a 4-mL borosilicate vial containing a Teflon-coated magnetic stirring bar were added **TT-19** (60.6 mg, 0.100 mmol, 1.00 equiv), **PTH** (1.6 mg, 5.0 μmol, 5.0 mol%), and tetrabutylammonium bromide (TBAB, 64 mg, 0.40 mmol, 2.0 equiv). The vial was transferred into a nitrogen-filled glovebox. Dry MeCN (2 mL, c = 0.05 M) and methyl acrylate (27 μL, 26 mg, 0.30 mmol, 3.0 equiv) were added. The vial was sealed with a Teflon-lined screw cap, removed from the glovebox, and transferred to a cryocooler precooled at –20 °C where the reaction mixture was stirred for 5 min without irradiation and then irradiated with white LEDs for 2 h. Subsequently, silica gel (~5 mL) was added and the reaction mixture was concentrated to dryness under reduced pressure. The residue was purified by flash column chromatography on silica gel (hexanes/EtOAc = 5:1) to afford (±)-**19** as a colorless oil in 62% yield (24.7 mg).

R*f* = 0.22 (hexanes/EtOAc = 5:1).

NMR Spectroscopy:

^{1}**H NMR** (500 MHz, $CDCl_3$, 23 °C, δ): 7.14 – 7.05 (m, 4H), 4.63 – 4.54 (m, 1H), 4.35 – 4.27 (m, 1H), 4.18 – 4.11 (m, 1H), 4.08 (t, *J* = 2.9 Hz, 0.63H, major isomer), 4.07 (t, *J* = 2.9 Hz, 0.37H, minor isomer), 3.66 (s, 3H), 3.37 (dd, *J* = 14.2, 8.3 Hz, 1H), 3.22 – 3.12 (m, 2H), 2.98 – 2.79 (m, 2H), 2.70 (dd, *J* = 13.4, 9.5 Hz, 1H), 1.13 (t, *J* = 7.3 Hz, 3H).

^{13}**C NMR** (125 MHz, $CDCl_3$, 23 °C, δ): 174.1, 169.8, 153.4, 135.9, 134.4, 129.8, 129.7, 66.2, 55.0(55.0), 53.0, 44.9(44.9), 40.7(40.6), 37.6, 29.2, 8.3.

HRMS-ESIpos (m/z) calc'd for $C_{17}H_{20}NO_5BrNa$ $[M+Na]^+$, 420.0417; found, 420.0417; deviation: 0.1 ppm.

(±)-Pyriproxyphen derivative 20

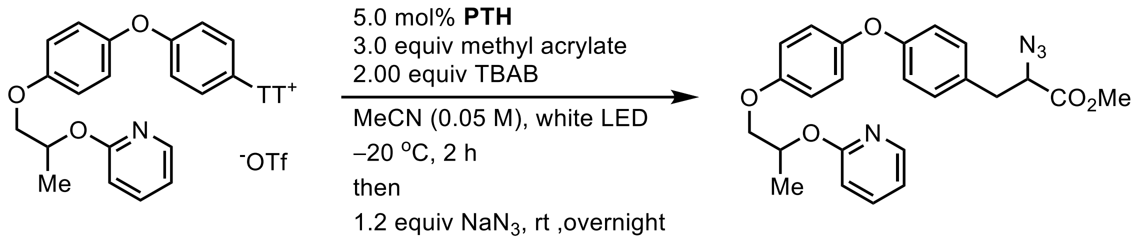

TT-20 (±)-**20**

To a 4-mL borosilicate vial containing a Teflon-coated magnetic stirring bar were added **TT-20** (68.5 mg, 0.100 mmol, 1.00 equiv), **PTH** (1.6 mg, 5.0 μmol, 5.0 mol%), and tetrabutylammonium bromide (TBAB, 64 mg, 0.40 mmol, 2.0 equiv). The vial was transferred into a nitrogen-filled glovebox. Dry MeCN (2 mL, c = 0.05 M) and methyl acrylate (27 μL, 26 mg, 0.30 mmol, 3.0 equiv) were added. The vial was sealed with a Teflon-lined screw cap, removed from the glovebox, and transferred to a cryocooler precooled at –20 °C where the reaction mixture was stirred for 5 min without irradiation and then irradiated with white LEDs for 2 h. The vial was warmed to room temperature and NaN_3 (7.8 mg, 0.12 mmol, 1.2 equiv) was added. The reaction mixture was stirred overnight. Subsequently, silica gel (~5 mL) was added and the reaction mixture was concentrated to dryness under reduced pressure. The residue was purified by flash column chromatography on silica gel (hexanes/EtOAc = 20:1) to afford (±)-**20** as a colorless oil in 60% yield (26.8 mg).

R*f* = 0.20 (hexanes/EtOAc = 20:1).

NMR Spectroscopy:

^{1}**H NMR** (500 MHz, $CDCl_3$, 23 °C, δ): 8.08 (dd, *J* = 5.1, 1.9 Hz, 1H), 7.50 (ddd, *J* = 8.6, 7.1, 2.0 Hz, 1H), 7.07 (d, *J* = 8.6 Hz, 2H), 6.91 – 6.78 (m, 7H), 6.68 (d, *J* = 8.2 Hz, 1H), 5.52 (h, *J* = 6.3 Hz, 1H), 4.11 (dd, *J* = 9.8, 5.3 Hz, 1H), 4.00 (dd, *J* = 9.9, 4.8 Hz, 1H), 3.97 (dd, *J* = 8.6, 5.3 Hz, 1H), 3.71 (s, 3H), 3.06 (dd, *J* = 14.1, 5.3 Hz, 1H), 2.89 (dd, *J* = 14.1, 8.7 Hz, 1H), 1.41 (d, *J* = 6.4 Hz, 3H).

^{13}**C NMR** (125 MHz, $CDCl_3$, 23 °C, δ): 170.4, 163.0, 157.8, 155.3, 150.1, 146.4, 139.0, 130.4, 129.8, 120.8, 117.7, 116.8, 115.8, 111.8, 71.1, 69.7, 63.4, 52.7, 36.9, 17.0.

HRMS-ESIpos (m/z) calc'd for $C_{24}H_{24}N_4O_5Na$ $[M+Na]^+$, 471.1639; found, 471.1640; deviation: –0.2 ppm.

(±)-Benzbromarone derivative 21

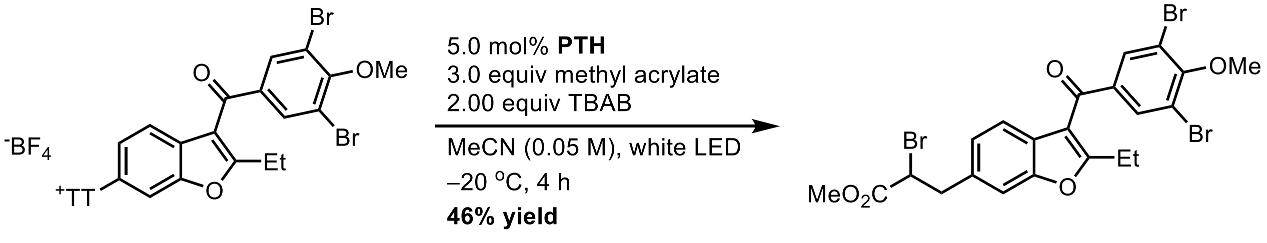

TT-21 (±)-**21**

To a 4-mL borosilicate vial containing a Teflon-coated magnetic stirring bar were added **TT-21** (73.8 mg, 0.100 mmol, 1.00 equiv), **PTH** (1.6 mg, 5.0 μmol, 5.0 mol%), and tetrabutylammonium bromide (TBAB, 64 mg, 0.40 mmol, 2.0 equiv). The vial was transferred into a nitrogen-filled glovebox. Dry MeCN (2 mL, c = 0.05 M) and methyl acrylate (27 μL, 26 mg, 0.30 mmol, 3.0 equiv) were added. The vial was sealed with a Teflon-lined screw cap, removed from the glovebox, and transferred to a cryocooler precooled at –20 °C where the reaction mixture was stirred for 5 min without irradiation and then irradiated with white LEDs for 4 h. Subsequently, silica gel (~5 mL) was added and the reaction mixture was concentrated to dryness under reduced pressure. The residue was purified by flash column chromatography on silica gel (hexanes/EtOAc = 20:1) to afford (±)-**21** as a colorless oil in 46% yield (27.6 mg).

R*f* = 0.18 (hexanes/EtOAc = 20:1).

NMR Spectroscopy:

^{1}H NMR (500 MHz, $CDCl_3$, 23 ºC, δ): 7.90 (s, 2H), 7.32 – 7.25 (m, 2H), 7.02 (dd, *J* = 7.9, 1.6 Hz, 1H), 4.36 (dd, *J* = 8.4, 7.0 Hz, 1H), 3.91 (s, 3H), 3.67 (s, 3H), 3.50 (dd, *J* = 14.1, 8.4 Hz, 1H), 3.28 (dd, *J* = 14.2, 7.0 Hz, 1H), 2.80 (q, *J* = 7.5 Hz, 2H), 1.28 (t, *J* = 7.5 Hz, 3H).

^{13}C NMR (125 MHz, $CDCl_3$, 23 ºC, δ): 187.9, 169.8, 167.2, 157.8, 153.8, 137.1, 133.8, 133.6, 125.7, 125.1, 121.1, 118.5, 115.3, 111.9, 60.9, 53.0, 45.0, 41.1, 22.1, 12.2.

HRMS-ESIpos (m/z) calc'd for $C_{22}H_{20}Br_3O_5$ $[M+H]^+$, 600.8855; found, 600.8850; deviation: –0.9 ppm.

(±)-2-Bromo-arylpropanoate 22

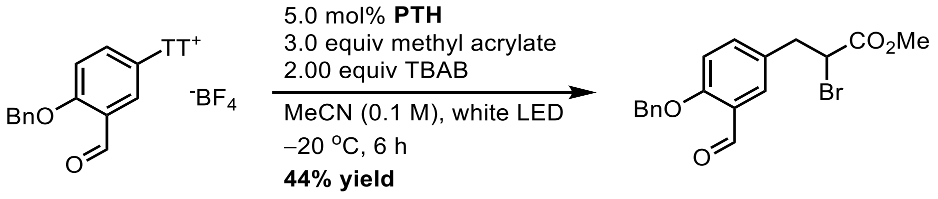

TT-22 (±)-**22**

To a 4-mL borosilicate vial containing a Teflon-coated magnetic stirring bar were added **TT-22** (102 mg, 0.200 mmol, 1.00 equiv), **PTH** (3.2 mg, 10 μmol, 5.0 mol%), and tetrabutylammonium bromide (TBAB, 128 mg, 0.400 mmol, 2.00 equiv). The vial was transferred into a nitrogen-filled glovebox. Dry MeCN (2 mL, c = 0.1 M) and methyl acrylate (54 μL, 52 mg, 0.60 mmol, 3.0 equiv) were added. The vial was sealed with a Teflon-lined screw cap, removed from the glovebox, and transferred to a cryocooler precooled at –20 °C where the reaction mixture was stirred for 5 min without irradiation and then irradiated with white LEDs for 6 h. Subsequently, silica gel (~5 mL) was added and the reaction mixture was concentrated to dryness under reduced pressure. The residue was purified by flash column chromatography on silica gel (hexanes/EtOAc = 10:1) to afford (±)-**22** as a light yellow semisolid in 44% yield (32.9 mg).

R*f* = 0.14 (hexanes/EtOAc = 10:1).

NMR Spectroscopy:

^{1}H NMR (500 MHz, $CDCl_3$, 23 ºC, δ): 10.44 (s, 1H), 7.62 (d, *J* = 2.4 Hz, 1H), 7.38 – 7.30 (m, 5H), 7.30 – 7.25 (m, 1H), 6.93 (d, *J* = 8.6 Hz, 1H), 5.10 (s, 2H), 4.30 (dd, *J* = 8.0, 7.3 Hz, 1H), 3.66 (s, 3H), 3.35 (dd, *J* = 14.3, 8.0 Hz, 1H), 3.14 (dd, *J* = 14.3, 7.4 Hz, 1H).

^{13}C NMR (125 MHz, $CDCl_3$, 23 ºC, δ): 189.3, 169.6, 160.3, 136.7, 135.9, 129.4, 128.7, 128.7, 128.3, 127.3, 125.1, 113.3, 70.6, 53.0, 45.0, 39.9.

HRMS-ESIpos (m/z) calc'd for $C_{18}H_{17}O_4BrNa$ $[M+Na]^+$, 399.0202; found, 399.0202; deviation: 0.1 ppm.

(±)-2-Bromo-arylpropanoate 23

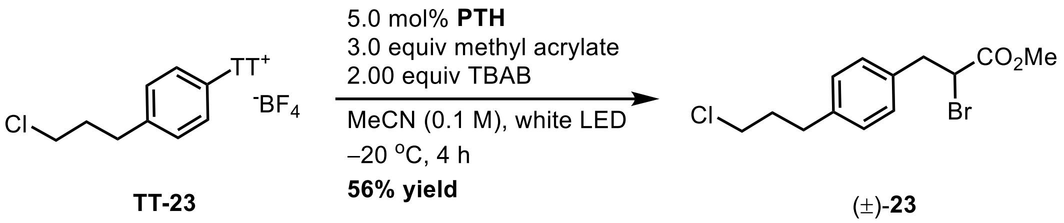

TT-23 (±)-**23**

To a 4-mL borosilicate vial containing a Teflon-coated magnetic stirring bar were added **TT-23** (90.9 mg, 0.200 mmol, 1.00 equiv), **PTH** (3.2 mg, 10 μmol, 5.0 mol%), and tetrabutylammonium bromide (TBAB, 128 mg, 0.400 mmol, 2.00 equiv). The vial was transferred into a nitrogen-filled glovebox. Dry MeCN (2 mL, c = 0.1 M) and methyl acrylate (54 μL, 52 mg, 0.60 mmol, 3.0 equiv) were added. The vial was sealed with a Teflon-lined screw cap, removed from the glovebox, and transferred to a cryocooler precooled at –20 °C where the reaction mixture was stirred for 5 min without irradiation and then irradiated with white LEDs for 4 h. Subsequently, silica gel (~5 mL) was added and the reaction mixture was concentrated to dryness under reduced pressure. The residue was purified by flash column chromatography on silica gel (hexanes/EtOAc = 40:1) to afford (±)-**23** as a colorless oil in 56% yield (35.4 mg).

R*f* = 0.21 (hexanes/EtOAc = 40:1).

NMR Spectroscopy:

^{1}H NMR (500 MHz, $CDCl_3$, 23 ºC, δ): 7.16 (s, 4H), 4.41 (dd, *J* = 8.4, 7.0 Hz, 1H), 3.76 (s, 3H), 3.54 (t, *J* = 6.5 Hz, 2H), 3.46 (dd, *J* = 14.1, 8.4 Hz, 1H), 3.24 (dd, *J* = 14.2, 7.1 Hz, 1H), 2.89 – 2.67 (m, 2H), 2.18 – 2.03 (m, 2H).

^{13}C NMR (125 MHz, $CDCl_3$, 23 ºC, δ): 169.9, 139.8, 134.5, 129.3, 128.9, 52.9, 45.2, 44.2, 40.7, 33.9, 32.4.

HRMS-EI (m/z) calc'd for $C_{13}H_{16}O_2BrCl$ $[M]^+$, 318.0017; found, 318.0017; deviation: –0.2 ppm.

(±)-2-Bromo-arylpropanoate 24

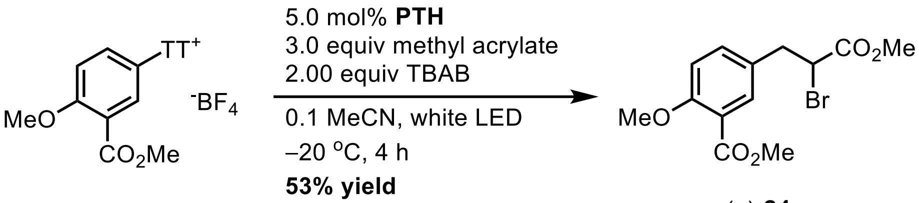

TT-24 (±)-**24**

To a 4-mL borosilicate vial containing a Teflon-coated magnetic stirring bar were added **TT-24** (92.8 mg, 0.200 mmol, 1.00 equiv), **PTH** (3.2 mg, 10 µmol, 5.0 mol%), and tetrabutylammonium bromide (TBAB, 128 mg, 0.400 mmol, 2.00 equiv). The vial was transferred into a nitrogen-filled glovebox. Dry MeCN (2 mL) and methyl acrylate (54 µL, 52 mg, 0.60 mmol, 3.0 equiv) were added. The vial was sealed with a Teflon-lined screw cap, removed from the glovebox, and transferred to a cryocooler precooled at –20 °C where the reaction mixture was stirred for 5 min without irradiation and then irradiated with white LEDs for 4 h. Subsequently, silica gel (~5 mL) was added and the reaction mixture was concentrated to dryness under reduced pressure. The residue was purified by flash column chromatography on silica gel (hexanes/EtOAc = 5:1) to afford (±)-**24** as a light yellow oil in 53% yield (34.5 mg).

R*f* = 0.17 (hexanes/EtOAc = 5:1).

NMR Spectroscopy:

^{1}H NMR (500 MHz, $CDCl_3$, 23 °C, δ): 7.66 (d, *J* = 2.4 Hz, 1H), 7.34 (dd, *J* = 8.5, 2.4 Hz, 1H), 6.94 (d, *J* = 8.5 Hz, 1H), 4.38 (dd, *J* = 8.3, 7.1 Hz, 1H), 3.91 (s, 3H), 3.90 (s, 3H), 3.75 (s, 3H), 3.43 (dd, *J* = 14.2, 8.3 Hz, 1H), 3.22 (dd, *J* = 14.2, 7.1 Hz, 1H).

^{13}C NMR (125 MHz, $CDCl_3$, 23 °C, δ): 169.7, 166.4, 158.4, 134.4, 132.3, 128.4, 120.1, 112.3, 56.1, 53.0, 52.1, 45.0, 40.0.

HRMS-ESIpos (m/z) calc'd for $C_{13}H_{15}BrNaO_5$ $[M+Na]^+$, 352.9995; found, 352.9994; deviation: 0.3 ppm.

(±)-2-Bromo-arylpropanoate 25

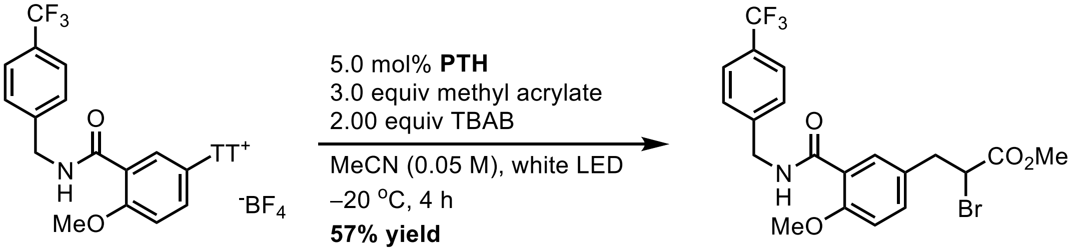

TT-25 (±)-**25**

To a 8-mL borosilicate vial containing a Teflon-coated magnetic stirring bar were added **TT-25** (122 mg, 0.200 mmol, 1.00 equiv), **PTH** (3.2 mg, 10 µmol, 5.0 mol%), and tetrabutylammonium bromide (TBAB, 128 mg, 0.400 mmol, 2.00 equiv). The vial was transferred into a nitrogen-filled glovebox. Dry MeCN (4 mL, c = 0.05 M) and

methyl acrylate (54 μL, 52 mg, 0.60 mmol, 3.0 equiv) were added. The vial was sealed with a Teflon-lined screw cap, removed from the glovebox, and transferred to a cryocooler precooled at –20 °C where the reaction mixture was stirred for 5 min without irradiation and then irradiated with white LEDs for 4 h. Subsequently, silica gel (~5 mL) was added and the reaction mixture was concentrated to dryness under reduced pressure. The residue was purified by flash column chromatography on silica gel (hexanes/EtOAc = 2:1) to afford (±)-**25** as a colorless solid in 55% yield (52.1 mg).

R*f* = 0.26 (hexanes/EtOAc = 2:1).

NMR Spectroscopy:

^{1}H NMR (500 MHz, $CDCl_3$, 23 °C, δ): 8.21 (t, *J* = 6.0 Hz, 1H), 8.02 (d, *J* = 2.4 Hz, 1H), 7.51 (d, *J* = 8.1 Hz, 2H), 7.39 (d, *J* = 8.0 Hz, 2H), 7.25 (dd, *J* = 8.5, 2.5 Hz, 1H), 6.86 (d, *J* = 8.5 Hz, 1H), 4.65 (d, *J* = 5.8 Hz, 2H), 4.33 (t, *J* = 7.7 Hz, 1H), 3.86 (s, 3H), 3.67 (s, 3H), 3.36 (dd, *J* = 14.3, 7.9 Hz, 1H), 3.15 (dd, *J* = 14.3, 7.4 Hz, 1H).

^{13}C NMR (125 MHz, $CDCl_3$, 23 °C, δ): 169.8, 165.2, 156.8, 142.9, 134.0, 133.0, 129.8, 129.5 (q, *J* = 32.3 Hz), 127.7, 125.6 (q, *J* = 3.9 Hz), 124.2 (q, *J* = 272.0 Hz), 121.2, 111.7, 56.1, 53.0, 45.2, 43.3, 40.0.

^{19}F NMR (470 MHz, $CDCl_3$, 23 °C, δ): –62.4.

HRMS-ESIpos (m/z) calc'd for $C_{20}H_{19}NO_4BrF_3Na$ $[M+Na]^+$, 496.0342; found, 496.0341; deviation: 0.2 ppm.

(±)-Indometacin methylester derivative 26

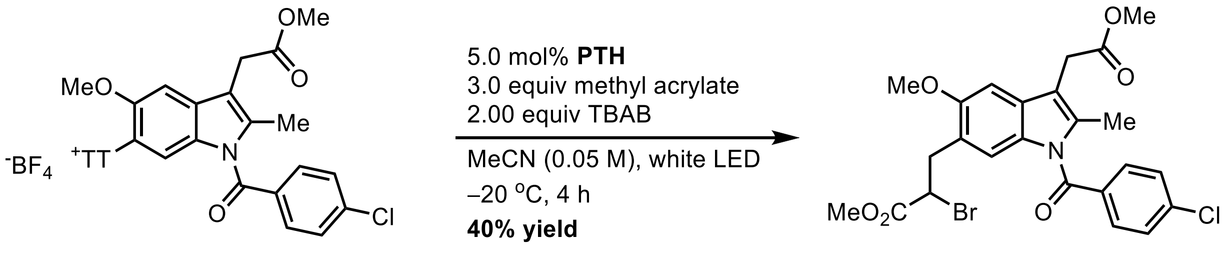

TT-26 (±)-**26**

To a 4-mL borosilicate vial containing a Teflon-coated magnetic stirring bar were added **TT-26** (67.2 mg, 0.100 mmol, 1.00 equiv), **PTH** (1.6 mg, 5.0 μmol, 5.0 mol%), and tetrabutylammonium bromide (TBAB, 64 mg, 0.40 mmol, 2.0 equiv). The vial was transferred into a nitrogen-filled glovebox. Dry MeCN (2 mL, c = 0.05 M) and methyl acrylate (27 μL, 26 mg, 0.30 mmol, 3.0 equiv) were added. The vial was sealed with a Teflon-lined screw cap, removed from the glovebox, and transferred to a cryocooler precooled at –20 °C where the reaction mixture was stirred for 5 min without irradiation and then irradiated with white LEDs for 4 h. Subsequently, silica gel (~5 mL) was added and the reaction mixture was concentrated to dryness under reduced pressure. The residue was purified by flash column chromatography on silica gel (hexanes/EtOAc = 5:1) to afford (±)-**26** as a colorless solid in 40% yield (21.4 mg).

R*f* = 0.21 (hexanes/EtOAc = 5:1).

NMR Spectroscopy:

^{1}H NMR (500 MHz, $CDCl_3$, 23 ºC, δ): 7.68 (d, *J* = 8.5 Hz, 2H), 7.49 (d, *J* = 8.5 Hz, 2H), 6.93 (s, 1H), 6.86 (s, 1H), 4.55 (t, *J* = 7.5 Hz, 1H), 3.91 (s, 3H), 3.73 (s, 6H), 3.69 (s, 2H), 3.36 (dd, *J* = 13.9, 7.3 Hz, 1H), 3.13 (dd, *J* = 13.9, 7.7 Hz, 1H), 2.38 (s, 3H).

^{13}C NMR (125 MHz, $CDCl_3$, 23 ºC, δ): 171.4, 170.1, 168.3, 154.2, 139.4, 135.6, 133.7, 131.2, 130.1, 129.7, 129.2, 121.2, 117.2, 112.3, 99.0, 55.6, 52.8, 52.2, 44.4, 37.0, 30.2, 13.3.

HRMS-ESIpos (m/z) calc'd for $C_{24}H_{23}NO_6BrClNa$ $[M+Na]^+$, 558.0290; found, 558.0294; deviation: –0.8 ppm.

(±)-2-Bromo-arylpropanoate 27

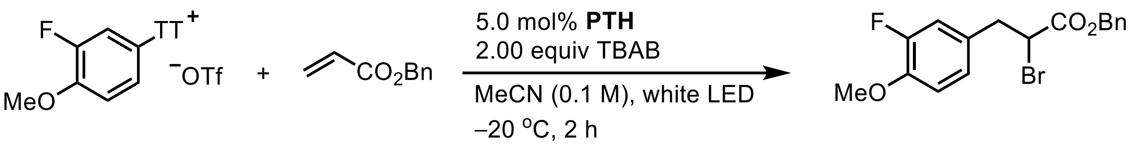

TT-1 3.0 equiv (±)-**27**

To a 4-mL borosilicate vial containing a Teflon-coated magnetic stirring bar were added **TT-1** (98.0 mg, 0.200 mmol, 1.00 equiv), **PTH** (3.2 mg, 10 μmol, 5.0 mol%), and tetrabutylammonium bromide (TBAB, 128 mg, 0.400 mmol, 2.00 equiv). The vial was transferred into a nitrogen-filled glovebox. Dry MeCN (2 mL, c = 0.1 M) and benzyl acrylate (90 μL, 97 mg, 0.60 mmol, 3.0 equiv) were added. The vial was sealed with a Teflon-lined screw cap, removed from the glovebox, and transferred to a cryocooler precooled at –20 °C where the reaction mixture was stirred for 5 min without irradiation and then irradiated with white LEDs for 2 h. Subsequently, silica gel (~5 mL) was added and the reaction mixture was concentrated to dryness under reduced pressure. The residue was purified by flash column chromatography on silica gel (hexanes/EtOAc = 40:1) to afford (±)-**27** as a colorless oil in 63% yield (46.1 mg).

R_f = 0.15 (hexanes/EtOAc = 40:1).

NMR Spectroscopy:

^{1}H NMR (500 MHz, $CDCl_3$, 23 ºC, δ): 7.39 – 7.35 (m, 3H), 7.32 – 7.29 (m, 2H), 6.98 – 6.88 (m, 2H), 6.86 (t, *J* = 8.3 Hz, 1H), 5.18 (s, 2H), 4.40 (dd, *J* = 8.7, 6.8 Hz, 1H), 3.88 (s, 3H), 3.41 (dd, *J* = 14.2, 8.7 Hz, 1H), 3.20 (dd, *J* = 14.2, 6.8 Hz, 1H).

^{13}C NMR (125 MHz, $CDCl_3$, 23 ºC, δ): 169.1, 152.1 (d, *J* = 246.2 Hz), 146.9 (d, *J* = 10.6 Hz), 134.9, 129.4 (d, *J* = 6.2 Hz), 128.6, 128.5, 128.3, 125.0 (d, *J* = 3.6 Hz), 116.9 (d, *J* = 18.5 Hz), 113.4 (d, *J* = 2.3 Hz), 67.7, 56.2, 45.0, 40.2.

^{19}F NMR (470 MHz, $CDCl_3$, 23 ºC, δ): –134.66 (dd, *J* = 11.8, 8.2 Hz).

HRMS-ESIpos (m/z) calc'd for $C_{17}H_{16}O_3BrFNa$ $[M+Na]^+$, 389.0159; found, 389.0157; deviation: 0.6 ppm.

(±)-2-Bromo-arylpropanoate 28

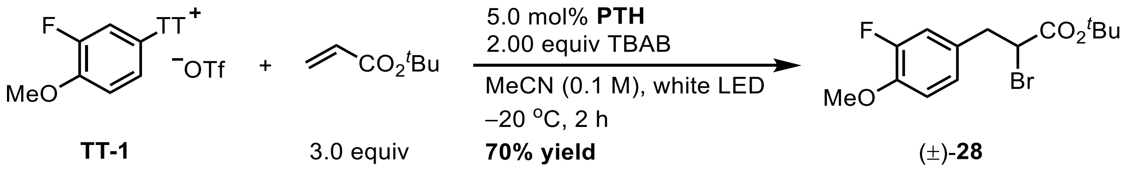

To a 4-mL borosilicate vial containing a Teflon-coated magnetic stirring bar were added **TT-1** (98.0 mg, 0.200 mmol, 1.00 equiv), **PTH** (3.2 mg, 10 µmol, 5.0 mol%), and tetrabutylammonium bromide (TBAB, 128 mg, 0.400 mmol, 2.00 equiv). The vial was transferred into a nitrogen-filled glovebox. Dry MeCN (2 mL, c = 0.1 M) and tertiary butyl acrylate (88 µL, 77 mg, 0.60 mmol, 3.0 equiv) were added. The vial was sealed with a Teflon-lined screw cap, removed from the glovebox, and transferred to a cryocooler precooled at –20 °C where the reaction mixture was stirred for 5 min without irradiation and then irradiated with white LEDs for 2 h. Subsequently, silica gel (~5 mL) was added and the reaction mixture was concentrated to dryness under reduced pressure. The residue was purified by flash column chromatography on silica gel (hexanes/EtOAc = 30:1) to afford (±)-**28** as a colorless oil in 70% yield (46.4 mg).

R*f* = 0.20 (hexanes/EtOAc = 30:1).

NMR Spectroscopy:

^{1}H NMR (500 MHz, $CDCl_3$, 23 °C, δ): 7.02 – 6.93 (m, 2H), 6.90 (t, *J* = 8.3 Hz, 1H), 4.24 (dd, *J* = 8.7, 6.8 Hz, 1H), 3.89 (s, 3H), 3.36 (dd, *J* = 14.2, 8.7 Hz, 1H), 3.14 (dd, *J* = 14.2, 6.8 Hz, 1H), 1.44 (s, 9H).

^{13}C NMR (125 MHz, $CDCl_3$, 23 °C, δ): 168.2, 152.1 (d, *J* = 246.2 Hz), 146.8 (d, *J* = 10.7 Hz), 129.9 (d, *J* = 6.4 Hz), 125.0 (d, *J* = 3.6 Hz), 116.9 (d, *J* = 18.5 Hz), 113.4 (d, *J* = 2.3 Hz), 82.6, 56.3, 46.8, 40.2, 27.7.

^{19}F NMR (470 MHz, $CDCl_3$, 23 °C, δ): –135.06 (dd, *J* = 12.0, 8.5 Hz).

HRMS-EI (m/z) calc'd for $C_{14}H_{18}O_3FBr$ $[M]^+$, 332.0418; found, 332.0421; deviation: –1.0 ppm.

(±)-2-Bromo-arylpropanenitrile 29

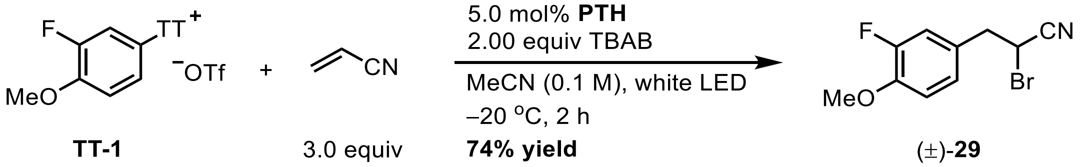

To a 4-mL borosilicate vial containing a Teflon-coated magnetic stirring bar were added **TT-1** (98.0 mg, 0.200 mmol, 1.00 equiv), **PTH** (3.2 mg, 10 µmol, 5.0 mol%), and tetrabutylammonium bromide (TBAB, 128 mg, 0.400 mmol, 2.00 equiv). The vial was transferred into a nitrogen-filled glovebox. Dry MeCN (2 mL, c = 0.1 M) and acrylonitrile (39 µL, 32 mg, 0.60 mmol, 3.0 equiv) were added. The vial was sealed with a Teflon-lined screw cap, removed from the glovebox, and transferred to a cryocooler precooled at –20 °C where the reaction mixture was stirred for 5 min without irradiation and then irradiated with white LEDs for 2 h. Subsequently, silica gel (~5 mL) was added and the reaction mixture was concentrated to dryness under reduced pressure. The residue was

purified by flash column chromatography on silica gel (hexanes/EtOAc = 20:1) to afford (±)-**29** as a colorless oil in 74% yield (38.1 mg).

R*f* = 0.15 (hexanes/EtOAc = 20:1).

NMR Spectroscopy:

^{1}**H NMR** (500 MHz, $CDCl_3$, 23 °C, δ): 6.96 (t, *J* = 2.5 Hz, 1H), 6.94 (s, 1H), 6.88 (t, *J* = 8.4 Hz, 1H), 4.30 (t, *J* = 7.3 Hz, 1H), 3.82 (s, 3H), 3.23 (dd, *J* = 7.4, 2.7 Hz, 2H).

^{13}**C NMR** (125 MHz, $CDCl_3$, 23 °C, δ): 152.2 (d, *J* = 247.3 Hz), 147.7 (d, *J* = 10.7 Hz), 127.2 (d, *J* = 6.4 Hz), 125.4 (d, *J* = 3.6 Hz), 117.1 (d, *J* = 18.9 Hz), 116.8, 113.7 (d, *J* = 2.3 Hz), 56.3, 41.7, 27.2.

^{19}**F NMR** (470 MHz, $CDCl_3$, 23 °C, δ): –134.0 (dd, *J* = 11.5, 8.7 Hz).

HRMS-EI (m/z) calc'd for $C_{10}H_9NOFBr$ $[M]^+$, 256.9846; found, 256.9847; deviation: –0.5 ppm.

(±)-3-Bromo-arylbutan-2-one 30

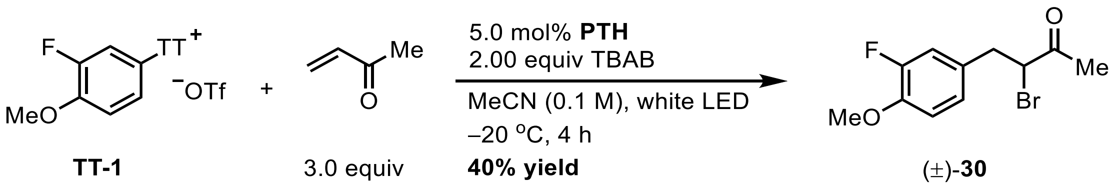

To a 4-mL borosilicate vial containing a Teflon-coated magnetic stirring bar were added **TT-1** (98.0 mg, 0.200 mmol, 1.00 equiv), **PTH** (3.2 mg, 10 μmol, 5.0 mol%), and tetrabutylammonium bromide (TBAB, 128 mg, 0.400 mmol, 2.00 equiv). The vial was transferred into a nitrogen-filled glovebox. Dry MeCN (2 mL, c = 0.1 M) and methyl vinyl ketone (49 μL, 42 mg, 0.60 mmol, 3.0 equiv) were added. The vial was sealed with a Teflon-lined screw cap, removed from the glovebox, and transferred to a cryocooler precooled at –20 °C where the reaction mixture was stirred for 5 min without irradiation and then irradiated with white LEDs for 4 h. Subsequently, silica gel (~5 mL) was added and the reaction mixture was concentrated to dryness under reduced pressure. The residue was purified by flash column chromatography on silica gel (hexanes/EtOAc = 20:1) to afford (±)-**30** as a colorless oil in 40% yield (21.8 mg).

R*f* = 0.18 (hexanes/EtOAc = 20:1).

NMR Spectroscopy:

^{1}**H NMR** (500 MHz, $CDCl_3$, 23 °C, δ): 6.91 – 6.79 (m, 3H), 4.33 (t, *J* = 7.4 Hz, 1H), 3.80 (s, 3H), 3.30 (dd, *J* = 14.5, 7.2 Hz, 1H), 3.01 (dd, *J* = 14.4, 7.5 Hz, 1H), 2.25 (s, 3H).

^{13}**C NMR** (125 MHz, $CDCl_3$, 23 °C, δ): 201.1, 152.2 (d, *J* = 246.2 Hz), 146.8 (d, *J* = 10.4 Hz), 129.9 (d, *J* = 6.5 Hz), 125.0 (d, *J* = 3.6 Hz), 116.9 (d, *J* = 18.4 Hz), 113.4 (d, *J* = 2.5 Hz), 56.3, 52.9, 38.5, 27.1.

^{19}**F NMR** (470 MHz, $CDCl_3$, 23 °C, δ): –134.7 (dd, *J* = 11.9, 7.7 Hz).

HRMS-EI (m/z) calc'd for $C_{11}H_{12}BrFO_2$ $[M]^+$, 274.0005; found, 274.0006; deviation: –0.5 ppm.

(±)-2-Bromo-(phenylsulfonyl)ethylarene 31

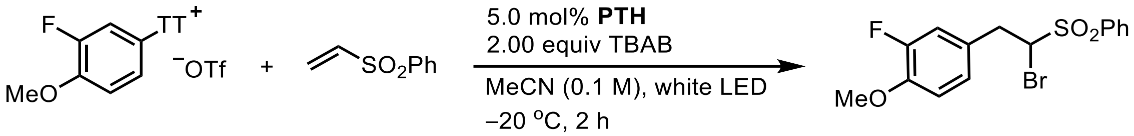

TT-1 3.0 equiv **62% yield** (±)-**31**

To a 4-mL borosilicate vial containing a Teflon-coated magnetic stirring bar were added **TT-1** (98.0 mg, 0.200 mmol, 1.00 equiv), **PTH** (3.2 mg, 10 μmol, 5.0 mol%), and tetrabutylammonium bromide (TBAB, 128 mg, 0.400 mmol, 2.00 equiv). The vial was transferred into a nitrogen-filled glovebox. Dry MeCN (2 mL, c = 0.1 M) and phenyl vinyl sulfone (101 mg, 0.60 mmol, 3.0 equiv) were added. The vial was sealed with a Teflon-lined screw cap, removed from the glovebox, and transferred to a cryocooler precooled at –20 °C where the reaction mixture was stirred for 5 min without irradiation and then irradiated with white LEDs for 2 h. Subsequently, silica gel (~5 mL) was added and the reaction mixture was concentrated to dryness under reduced pressure. The residue was purified by flash column chromatography on silica gel (hexanes/acetone = 10:1) to afford (±)-**31** as a light yellow solid in 62% yield (46.1 mg).

R*f* = 0.31 (hexanes/acetone = 5:1).

NMR Spectroscopy:

^{1}**H NMR** (500 MHz, $CDCl_3$, 23 °C, δ): 7.94 (d, *J* = 7.1 Hz, 2H), 7.66 (t, *J* = 7.5 Hz, 1H), 7.54 (t, *J* = 7.9 Hz, 2H), 6.91 – 6.78 (m, 3H), 4.72 (dd, *J* = 11.4, 2.7 Hz, 1H), 3.82 – 3.74 (m, 4H), 2.93 (dd, *J* = 14.6, 11.4 Hz, 1H).

^{13}**C NMR** (125 MHz, $CDCl_3$, 23 °C, δ): 152.2 (d, *J* = 246.8 Hz), 147.1 (d, *J* = 10.6 Hz), 135.2, 134.7, 130.1, 129.2, 127.8 (d, *J* = 6.4 Hz), 125.2 (d, *J* = 3.5 Hz), 117.0 (d, *J* = 18.8 Hz), 113.5 (d, *J* = 2.3 Hz), 66.3, 56.2, 36.6.

^{19}**F NMR** (470 MHz, $CDCl_3$, 23 °C, δ): –134.40 (dd, *J* = 11.7, 8.1 Hz).

HRMS-ESIpos (m/z) calc'd for $C_{15}H_{14}O_3SBrFNa$ $[M+Na]^+$, 394.9723; found, 394.9720; deviation: 0.8 ppm.

(±)-1-Bromo-arylethylphosphonate 32

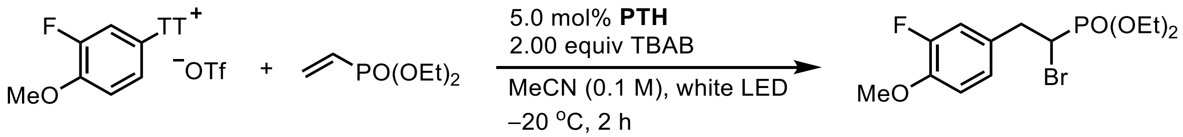

TT-1 3.0 equiv **67% yield** (±)-**32**

To a 4-mL borosilicate vial containing a Teflon-coated magnetic stirring bar were added **TT-1** (98.0 mg, 0.200

mmol, 1.00 equiv), **PTH** (3.2 mg, 10 μmol, 5.0 mol%), and tetrabutylammonium bromide (TBAB, 128 mg, 0.400 mmol, 2.00 equiv). The vial was transferred into a nitrogen-filled glovebox. Dry MeCN (2 mL, c = 0.1 M) and diethyl vinylphosphonate (92 μL, 98 mg, 0.60 mmol, 3.0 equiv) were added. The vial was sealed with a Teflon-lined screw cap, removed from the glovebox, and transferred to a cryocooler precooled at –20 °C where the reaction mixture was stirred for 5 min without irradiation and then irradiated with white LEDs for 2 h. Subsequently, silica gel (~5 mL) was added and the reaction mixture was concentrated to dryness under reduced pressure. The residue was purified by flash column chromatography on silica gel (hexanes/EtOAc = 1:2) to afford (±)-**32** as a light yellow oil in 67% yield (49.2 mg).

R*f* = 0.33 (hexanes/EtOAc = 1:2).

NMR Spectroscopy:

^{1}H NMR (500 MHz, $CDCl_3$, 23 °C, δ): 7.02 – 6.84 (m, 3H), 4.35 – 4.14 (m, 4H), 3.92 (ddd, *J* = 11.4, 9.8, 3.5 Hz, 1H), 3.88 (s, 3H), 3.51 (ddd, *J* = 15.0, 7.1, 3.4 Hz, 1H), 3.00 (ddd, *J* = 15.0, 11.3, 8.3 Hz, 1H), 1.37 (td, *J* = 7.1, 2.2 Hz, 6H).

^{13}C NMR (125 MHz, $CDCl_3$, 23 °C, δ): 152.1 (d, *J* = 246.1 Hz), 146.7 (d, *J* = 10.7 Hz), 130.2 (dd, *J* = 14.7, 6.3 Hz), 124.9 (d, *J* = 3.7 Hz), 116.8 (d, *J* = 18.4 Hz), 113.3 (d, *J* = 2.3 Hz), 64.0 (d, *J* = 6.8 Hz), 63.5 (d, *J* = 6.8 Hz), 56.2, 42.7 (d, *J* = 155.2 Hz), 37.8, 16.5 (d, *J* = 5.3 Hz), 16.4 (d, *J* = 5.4 Hz).

^{19}F NMR (470 MHz, $CDCl_3$, 23 °C, δ): –135.1 (dd, *J* = 11.9, 8.1 Hz).

^{31}P NMR (203 MHz, $CDCl_3$, 23 °C, δ): 19.36.

HRMS-ESIpos (m/z) calc'd for $C_{13}H_{19}O_4PBrFNa$ $[M+Na]^+$, 391.0081; found, 391.0077; deviation: 1.0 ppm.

(±)-Butyrolactone 33

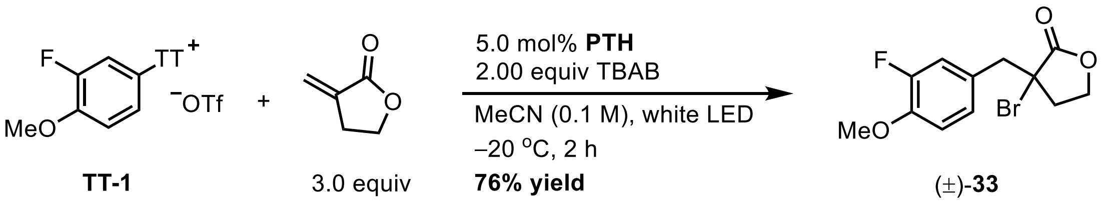

To a 4-mL borosilicate vial containing a Teflon-coated magnetic stirring bar were added **TT-1** (98.0 mg, 0.200 mmol, 1.00 equiv), **PTH** (3.2 mg, 10 μmol, 5.0 mol%), and tetrabutylammonium bromide (TBAB, 128 mg, 0.400 mmol, 2.00 equiv). The vial was transferred into a nitrogen-filled glovebox. Dry MeCN (2 mL, c = 0.1 M) and 2-methylenebutyrolactone (53 μL, 59 mg, 0.60 mmol, 3.0 equiv) were added. The vial was sealed with a Teflon-lined screw cap, removed from the glovebox, and transferred to a cryocooler precooled at –20 °C where the reaction mixture was stirred for 5 min without irradiation and then irradiated with white LEDs for 2 h. Subsequently, silica gel (~5 mL) was added and the reaction mixture was concentrated to dryness under reduced pressure. The residue was purified by flash column chromatography on silica gel (hexanes/EtOAc = 3:1) to afford (±)-**33** as a light yellow oil in 76% yield (46.1 mg).

R*f* = 0.26 (hexanes/EtOAc = 3:1).

NMR Spectroscopy:

^{1}H NMR (500 MHz, $CDCl_3$, 23 °C, δ): 7.11 – 6.98 (m, 2H), 6.93 (t, *J* = 8.6 Hz, 1H), 4.48 – 4.36 (m, 1H), 4.29 (t, *J* = 8.5 Hz, 1H), 3.89 (s, 3H), 3.50 (d, *J* = 14.3 Hz, 1H), 3.40 (d, *J* = 14.3 Hz, 1H), 2.49 (ddd, *J* = 14.5, 10.3, 7.9 Hz, 1H), 2.41 (dd, *J* = 14.5, 5.4 Hz, 1H).

^{13}C NMR (125 MHz, $CDCl_3$, 23 °C, δ): 173.9, 152.1 (d, *J* = 246.7 Hz), 147.2 (d, *J* = 10.4 Hz), 127.7 (d, *J* = 6.0 Hz), 126.4 (d, *J* = 3.6 Hz), 118.0 (d, *J* = 18.5 Hz), 113.5 (d, *J* = 2.3 Hz), 65.6, 57.8, 56.2, 43.3, 37.0.

^{19}F NMR (470 MHz, $CDCl_3$, 23 °C, δ): –134.4 (dd, *J* = 11.8, 8.7 Hz).

HRMS-EI (m/z) calc'd for $C_{12}H_{12}O_3FBr$ $[M]^+$, 301.9948; found, 301.9949; deviation: –0.2 ppm.

(±)-2-Bromo-2-fluoro-arylpropanoate 34

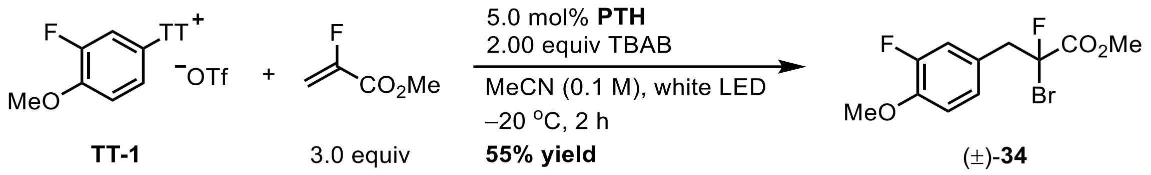

To a 4-mL borosilicate vial containing a Teflon-coated magnetic stirring bar were added **TT-1** (98.0 mg, 0.200 mmol, 1.00 equiv), **PTH** (3.2 mg, 10 µmol, 5.0 mol%), and tetrabutylammonium bromide (TBAB, 128 mg, 0.400 mmol, 2.00 equiv). The vial was transferred into a nitrogen-filled glovebox. Dry MeCN (2 mL, c = 0.1 M) and methyl 2-fluoroacrylate (56 µL, 62 mg, 0.60 mmol, 3.0 equiv) were added. The vial was sealed with a Teflon-lined screw cap, removed from the glovebox, and transferred to a cryocooler precooled at –20 °C where the reaction mixture was stirred for 5 min without irradiation and then irradiated with white LEDs for 2 h. Subsequently, silica gel (~5 mL) was added and the reaction mixture was concentrated to dryness under reduced pressure. The residue was purified by flash column chromatography on silica gel (hexanes/EtOAc = 20:1) to afford (±)-**34** as a colorless oil in 60% yield (36.9 mg).

R*f* = 0.12 (hexanes/EtOAc = 20:1).

NMR Spectroscopy:

^{1}H NMR (500 MHz, $CDCl_3$, 23 °C, δ): 7.06 – 6.97 (m, 2H), 6.92 (t, *J* = 8.4 Hz, 1H), 3.90 (s, 3H), 3.86 (s, 3H), 3.70 (d, *J* = 3.2 Hz, 1H), 3.66 (s, 1H).

^{13}C NMR (125 MHz, $CDCl_3$, 23 °C, δ): 166.1 (d, *J* = 27.4 Hz), 152.0 (d, *J* = 246.2 Hz), 147.5 (d, *J* = 10.7 Hz), 126.5 (d, *J* = 3.6 Hz), 125.4 (d, *J* = 6.5 Hz), 118.2 (d, *J* = 19.0 Hz), 113.2 (d, *J* = 2.3 Hz), 96.6 (d, *J* = 268.0 Hz), 56.2, 53.7, 46.6 (d, *J* = 19.7 Hz).

^{19}F NMR (470 MHz, $CDCl_3$, 23 °C, δ): –118.0 (dd, *J* = 24.0, 20.8 Hz, 1H), -134.8 (dd, *J* = 11.8, 8.4 Hz, 1H).

HRMS-EI (m/z) calc'd for $C_{11}H_{11}O_3F_2Br$ $[M]^+$, 307.9854; found, 307.9850; deviation: 1.3 ppm.

(±)-2-Bromo-arylpropanal 35

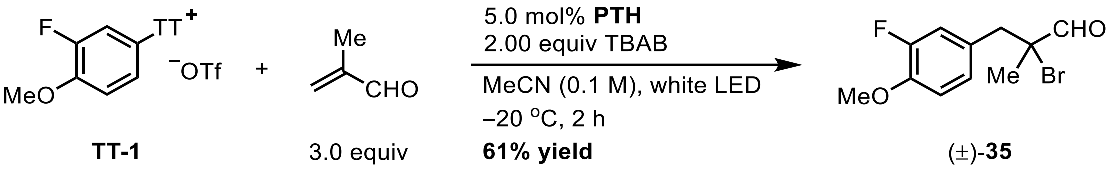

To a 4-mL borosilicate vial containing a Teflon-coated magnetic stirring bar were added **TT-1** (98.0 mg, 0.200 mmol, 1.00 equiv), **PTH** (3.2 mg, 10 μmol, 5.0 mol%), and tetrabutylammonium bromide (TBAB, 128 mg, 0.400 mmol, 2.00 equiv). The vial was transferred into a nitrogen-filled glovebox. Dry MeCN (2 mL, c = 0.1 M) and methacrolein (49 μL, 42 mg, 0.60 mmol, 3.0 equiv) were added. The vial was sealed with a Teflon-lined screw cap, removed from the glovebox, and transferred to a cryocooler precooled at –20 °C where the reaction mixture was stirred for 5 min without irradiation and then irradiated with white LEDs for 2 h. Subsequently, silica gel (~5 mL) was added and the reaction mixture was concentrated to dryness under reduced pressure. The residue was purified by flash column chromatography on silica gel (hexanes/EtOAc = 40:1) to afford (±)-**35** as a colorless oil in 61% yield (33.2 mg).

R*f* = 0.32 (hexanes/EtOAc = 20:1).

NMR Spectroscopy:

^{1}H NMR (500 MHz, $CDCl_3$, 23 °C, δ): 9.38 (s, 1H), 6.92 – 6.88 (m, 1H), 6.86 – 6.80 (m, 2H), 3.81 (s, 3H), 3.27 – 3.13 (m, 2H), 1.64 (s, 3H).

^{13}C NMR (125 MHz, $CDCl_3$, 23 °C, δ): 192.7, 151.9 (d, *J* = 246.3 Hz), 147.0 (d, *J* = 10.7 Hz), 127.7 (d, *J* = 6.1 Hz), 126.3 (d, *J* = 3.6 Hz), 118.2 (d, *J* = 18.7 Hz), 113.2 (d, *J* = 2.3 Hz), 68.0, 56.2, 43.8, 23.7.

^{19}F NMR (470 MHz, $CDCl_3$, 23 °C, δ): –134.8 (dd, *J* = 12.1, 6.8 Hz).

HRMS-EI (m/z) calc'd for $C_{11}H_{12}O_2FBr$ $[M]^+$, 273.9999; found, 274.0000; deviation: –0.1 ppm.

(±)-2-Bromo-arylpropanamide 36

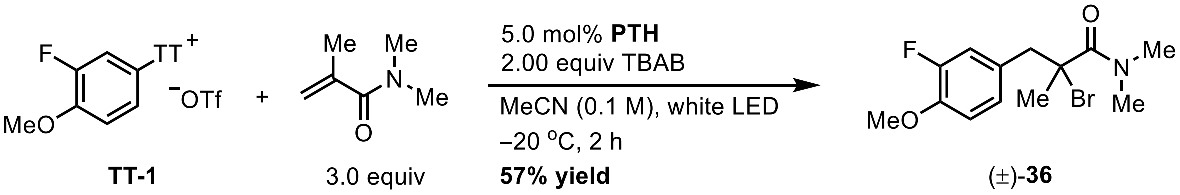

To a 4-mL borosilicate vial containing a Teflon-coated magnetic stirring bar were added **TT-1** (98.0 mg, 0.200 mmol, 1.00 equiv), **PTH** (3.2 mg, 10 μmol, 5.0 mol%), and tetrabutylammonium bromide (TBAB, 128 mg, 0.400 mmol, 2.00 equiv). The vial was transferred into a nitrogen-filled glovebox. Dry MeCN (2 mL, c = 0.1 M) and *N,N*-dimethylmethacrylamide (68 mg, 0.60 mmol, 3.0 equiv) were added. The vial was sealed with a Teflon-lined screw cap, removed from the glovebox, and transferred to a cryocooler precooled at –20 °C where the reaction

mixture was stirred for 5 min without irradiation and then irradiated with white LEDs for 2 h. Subsequently, silica gel (~5 mL) was added and the reaction mixture was concentrated to dryness under reduced pressure. The residue was purified by flash column chromatography on silica gel (hexanes/EtOAc = 2:1) to afford (±)-**36** as a light yellow oil in 57% yield (36.3 mg).

R*f* = 0.30 (hexanes/EtOAc = 2:1).

NMR Spectroscopy:

^{1}H NMR (500 MHz, $CDCl_3$, 23 °C, δ): 6.98 (dd, *J* = 12.3, 2.0 Hz, 1H), 6.96 – 6.86 (m, 2H), 3.89 (s, 3H), 3.49 (d, *J* = 14.3 Hz, 1H), 3.38 (d, *J* = 14.3 Hz, 1H), 3.23 (br, 6H), 1.88 (s, 3H).

^{13}C NMR (125 MHz, $CDCl_3$, 23 °C, δ): 169.3, 151.8 (d, *J* = 245.5 Hz), 146.8 (d, *J* = 10.7 Hz), 128.9 (d, *J* = 6.1 Hz), 126.4 (d, *J* = 3.5 Hz), 118.3 (d, *J* = 18.5 Hz), 113.0 (d, *J* = 2.2 Hz), 60.8, 56.2, 47.2, 39.4 (br), 29.8.

^{19}F NMR (470 MHz, $CDCl_3$, 23 °C, δ): –135.4 (dd, *J* = 12.2, 8.3 Hz).

HRMS-CI (m/z) calc'd for $C_{13}H_{18}NO_2FBr$ $[M+H]^+$, 318.0500; found, 318.0504; deviation: –1.4 ppm.

(±)-2-Bromo-arylpropanamide 37

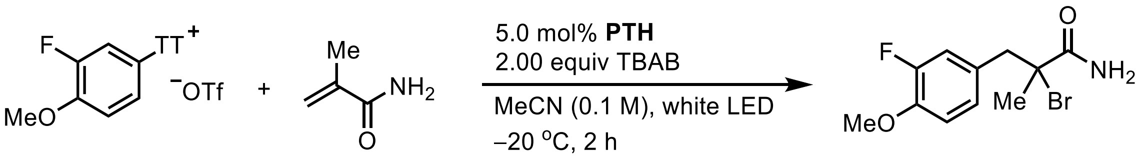

TT-1 3.0 equiv (±)-**37**

To a 4-mL borosilicate vial containing a Teflon-coated magnetic stirring bar were added **TT-1** (98.0 mg, 0.200 mmol, 1.00 equiv), **PTH** (3.2 mg, 10 μmol, 5.0 mol%), and tetrabutylammonium bromide (TBAB, 128 mg, 0.400 mmol, 2.00 equiv). The vial was transferred into a nitrogen-filled glovebox. Dry MeCN (2 mL, c = 0.1 M) and methacrylamide (51 mg, 0.60 mmol, 3.0 equiv) were added. The vial was sealed with a Teflon-lined screw cap, removed from the glovebox, and transferred to a cryocooler precooled at –20 °C where the reaction mixture was stirred for 5 min without irradiation and then irradiated with white LEDs for 2 h. Subsequently, silica gel (~5 mL) was added and the reaction mixture was concentrated to dryness under reduced pressure. The residue was purified by flash column chromatography on silica gel (hexanes/EtOAc = 2:1) to afford (±)-**37** as a light yellow oil in 58% yield (33.5 mg).

R*f* = 0.25 (hexanes/EtOAc = 2:1).

NMR Spectroscopy:

^{1}H NMR (500 MHz, $CDCl_3$, 23 °C, δ): 6.99 (dd, *J* = 12.3, 2.2 Hz, 1H), 6.92 (d, *J* = 8.3 Hz, 1H), 6.81 (t, *J* = 8.5 Hz, 1H), 6.46 (s, 1H), 5.83 (s, 1H), 3.80 (s, 3H), 3.36 (d, *J* = 14.2 Hz, 1H), 3.08 (d, *J* = 14.2 Hz, 1H), 1.91 (s, 3H).

^{13}C NMR (125 MHz, $CDCl_3$, 23 °C, δ): 173.4, 151.7 (d, *J* = 245.0 Hz), 146.9 (d, *J* = 10.7 Hz), 128.9 (d, *J* = 6.2 Hz), 126.5 (d, *J* = 3.5 Hz), 118.3 (d, *J* = 18.5 Hz), 112.8 (d, *J* = 2.3 Hz), 67.6, 56.2, 48.0, 30.9.

^{19}F NMR (470 MHz, $CDCl_3$, 23 °C, δ): –135.7 (dd, *J* = 12.5, 8.8 Hz).

HRMS-CI (m/z) calc'd for $C_{11}H_{14}NO_2FBr$ $[M+H]^+$, 290.0187; found, 290.0190; deviation: –1.1 ppm.

(±)-2-Bromo-2-phenylethylarene 38

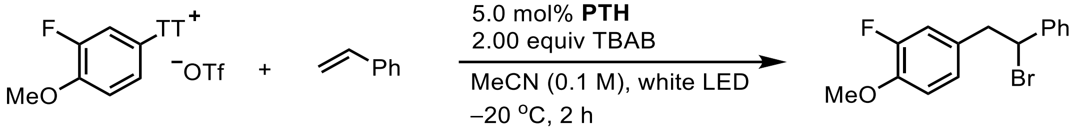

To a 4-mL borosilicate vial containing a Teflon-coated magnetic stirring bar were added **TT-1** (98.0 mg, 0.200 mmol, 1.00 equiv), **PTH** (3.2 mg, 10 µmol, 5.0 mol%), and tetrabutylammonium bromide (TBAB, 128 mg, 0.400 mmol, 2.00 equiv). The vial was transferred into a nitrogen-filled glovebox. Dry MeCN (2 mL, c = 0.1 M) and styrene (69 µL, 62 mg, 0.60 mmol, 3.0 equiv) were added. The vial was sealed with a Teflon-lined screw cap, removed from the glovebox, and transferred to a cryocooler precooled at –20 °C where the reaction mixture was stirred for 5 min without irradiation and then irradiated with white LEDs for 2 h. Subsequently, silica gel (~5 mL) was added and the reaction mixture was concentrated to dryness under reduced pressure. The residue was purified by flash column chromatography on silica gel (hexanes/EtOAc = 40:1) to afford (±)-**38** as a colorless oil in 68% yield (41.9 mg).

R*f* = 0.36 (hexanes/EtOAc = 10:1).

NMR Spectroscopy:

^{1}H NMR (500 MHz, $CDCl_3$, 23 °C, δ): 7.44 – 7.38 (m, 2H), 7.37 – 7.32 (m, 1H), 7.32 – 7.27 (m, 1H), 6.89 – 6.82 (m, 3H), 5.09 (t, *J* = 7.6 Hz, 1H), 3.87 (s, 3H), 3.51 (dd, *J* = 14.3, 7.7 Hz, 1H), 3.44 (dd, *J* = 14.3, 7.4 Hz, 1H).

^{13}C NMR (125 MHz, $CDCl_3$, 23 °C, δ): 152.0 (d, *J* = 246.0 Hz), 146.5 (d, *J* = 10.4 Hz), 141.2, 131.0 (d, *J* = 6.1 Hz), 128.7, 128.5, 127.5, 125.0 (d, *J* = 3.5 Hz), 116.9 (d, *J* = 18.4 Hz), 113.2 (d, *J* = 2.2 Hz), 56.2, 55.2, 45.5.

^{19}F NMR (470 MHz, $CDCl_3$, 23 °C, δ): –135.3 (dd, *J* = 11.7, 7.1 Hz).

HRMS-EI (m/z) calc'd for $C_{15}H_{14}OFBr$ $[M]^+$, 308.0207; found, 308.0208; deviation: –0.4 ppm.

(±)-2-Bromo-2-phenylethylarene 39

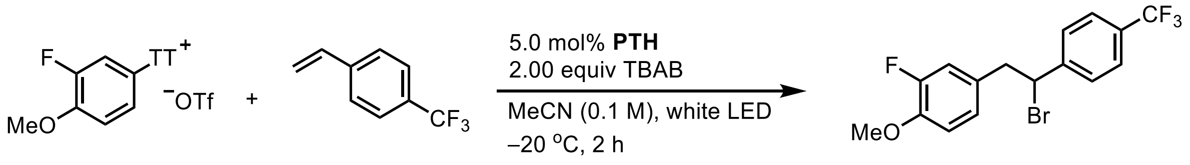

To a 4-mL borosilicate vial containing a Teflon-coated magnetic stirring bar were added **TT-1** (98.0 mg, 0.200

mmol, 1.00 equiv), **PTH** (3.2 mg, 10 μmol, 5.0 mol%), and tetrabutylammonium bromide (TBAB, 128 mg, 0.400 mmol, 2.00 equiv). The vial was transferred into a nitrogen-filled glovebox. Dry MeCN (2 mL, c = 0.1 M) and 4-(trifluoromethyl)styrene (90 μL, 104 mg, 0.60 mmol, 3.0 equiv) were added. The vial was sealed with a Teflon-lined screw cap, removed from the glovebox, and transferred to a cryocooler precooled at –20 °C where the reaction mixture was stirred for 5 min without irradiation and then irradiated with white LEDs for 2 h. Subsequently, silica gel (~5 mL) was added and the reaction mixture was concentrated to dryness under reduced pressure. The residue was purified by flash column chromatography on silica gel (hexanes/EtOAc = 20:1) to afford (±)-**39** as a colorless oil in 62% yield (46.5 mg).

R*f* = 0.17 (hexanes/EtOAc = 20:1).

NMR Spectroscopy:

^{1}H NMR (500 MHz, $CDCl_3$, 23 °C, δ): 7.61 (d, *J* = 8.1 Hz, 2H), 7.49 (d, *J* = 8.1 Hz, 2H), 6.90 – 6.83 (m, 2H), 6.83 – 6.77 (m, 1H), 5.09 (t, *J* = 7.6 Hz, 1H), 3.87 (s, 3H), 3.50 (dd, *J* = 14.3, 7.6 Hz, 1H), 3.41 (dd, *J* = 14.3, 7.6 Hz, 1H).

^{13}C NMR (125 MHz, $CDCl_3$, 23 °C, δ): 152.1 (d, *J* = 246.1 Hz), 146.7 (d, *J* = 10.3 Hz), 145.1, 130.5 (q, *J* = 32.5 Hz), 130.3 (d, *J* = 6.0 Hz), 127.9, 125.7 (q, *J* = 4.0 Hz), 125.0 (d, *J* = 3.6 Hz), 123.8 (q, *J* = 272.2 Hz), 116.8 (d, *J* = 18.4 Hz), 113.3 (d, *J* = 2.3 Hz), 56.2, 53.3, 45.3.

^{19}F NMR (470 MHz, $CDCl_3$, 23 °C, δ): –62.7 (s, 3F), –134.9 (dd, *J* = 11.9, 8.6 Hz, 1H).

HRMS-EI (m/z) calc'd for $C_{16}H_{13}OF_4Br$ $[M]^+$, 376.0081; found, 376.0083; deviation: –0.5 ppm.

(±)-2,4-Dibromobutylarene 40

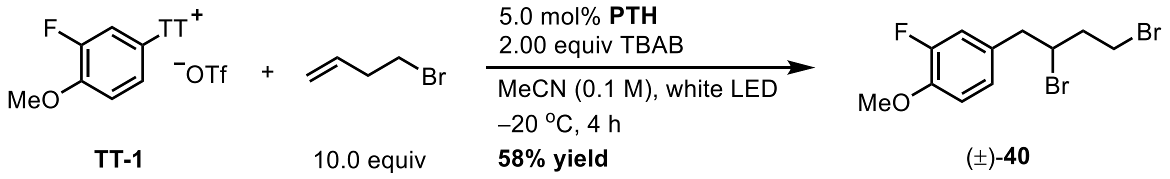

To a 4-mL borosilicate vial containing a Teflon-coated magnetic stirring bar were added **TT-1** (98.0 mg, 0.200 mmol, 1.00 equiv), **PTH** (3.2 mg, 10 μmol, 5.0 mol%), and tetrabutylammonium bromide (TBAB, 128 mg, 0.400 mmol, 2.00 equiv). The vial was transferred into a nitrogen-filled glovebox. Dry MeCN (2 mL, c = 0.1 M) and 4-bromo-1-butene (203 μL, 270 mg, 2.00 mmol, 10.0 equiv) were added. The vial was sealed with a Teflon-lined screw cap, removed from the glovebox, and transferred to a cryocooler precooled at –20 °C where the reaction mixture was stirred for 5 min without irradiation and then irradiated with white LEDs for 4 h. Subsequently, silica gel (~5 mL) was added and the reaction mixture was concentrated to dryness under reduced pressure. The residue was purified by flash column chromatography on silica gel (hexanes/EtOAc = 40:1) to afford (±)-**40** as a colorless oil in 45% yield (30.5 mg).

R*f* = 0.20 (hexanes/EtOAc = 40:1).

NMR Spectroscopy:

^{1}H NMR (500 MHz, $CDCl_3$, 23 °C, δ): 6.96 – 6.79 (m, 3H), 4.26 (dtd, *J* = 9.4, 7.0, 3.9 Hz, 1H), 3.81 (s, 3H), 3.60 – 3.47 (m, 2H), 3.19 – 2.95 (m, 2H), 2.31 – 2.11 (m, 2H).

^{13}C NMR (125 MHz, $CDCl_3$, 23 °C, δ): 152.2 (d, *J* = 246.2 Hz), 146.7 (d, *J* = 10.6 Hz), 130.6 (d, *J* = 6.4 Hz), 124.9 (d, *J* = 3.5 Hz), 116.9 (d, *J* = 18.3 Hz), 113.4 (d, *J* = 2.3 Hz), 56.3, 54.2, 44.3, 40.5, 31.1.

^{19}F NMR (470 MHz, $CDCl_3$, 23 °C, δ): –134.9 – –135.0 (m).

HRMS-EI (m/z) calc'd for $C_{11}H_{13}OFBr_2$ $[M]^+$, 337.9312; found, 337.9316; deviation: –1.1 ppm.

Synthetic utility

Synthesis of (±)-methyl 2-azidopropanoate 41

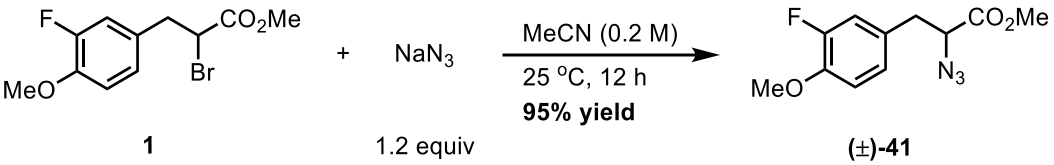

To a 4-mL borosilicate vial containing a Teflon-coated magnetic stirring bar were added **1** (29.0 mg, 0.100 mmol, 1.00 equiv), sodium azide (7.8 mg, 0.12 mmol, 1.2 equiv) and MeCN (0.5 mL, c = 0.2 M). The vial was sealed with a Teflon-lined screw cap and stirred at 25 °C for 12 h. The resulting solution was diluted with ethyl acetate (10 mL), washed by water (10 mL) and brine (10 mL), dried over Na_2SO_4, and concentrated under reduced pressure. The residue was purified by flash column chromatography on silica gel (hexanes/EtOAc = 20:1) to afford (±)-**41** as a colorless oil in 95% yield (24.0 mg)

R*f* = 0.18 (hexanes/EtOAc = 20:1).

NMR Spectroscopy:

^{1}H NMR (500 MHz, $CDCl_3$, 23 °C, δ): 7.08 – 6.89 (m, 3H), 4.07 (dd, *J* = 8.5, 5.4 Hz, 1H), 3.89 (s, 3H), 3.80 (s, 3H), 3.11 (dd, *J* = 14.2, 5.4 Hz, 1H), 2.96 (dd, *J* = 14.2, 8.5 Hz, 1H).

^{13}C NMR (125 MHz, $CDCl_3$, 23 °C, δ): 170.2, 152.2 (d, *J* = 246.2 Hz), 146.9 (d, *J* = 10.5 Hz), 128.7 (d, *J* = 6.5 Hz), 125.0 (d, *J* = 3.6 Hz), 116.9 (d, *J* = 18.5 Hz), 113.5 (d, *J* = 2.3 Hz), 63.2, 56.3, 52.7, 36.7.

^{19}F NMR (470 MHz, $CDCl_3$, 23 °C, δ): -134.8 (dd, *J* = 12.0, 7.8 Hz).

HRMS-ESIpos (m/z) calc'd for $C_{11}H_{12}N_3O_3FNa$ $[M+Na]^+$, 276.0755; found, 276.0751; deviation: 1.2 ppm

Synthesis of (±)-methyl 2-nitropropanoate 42

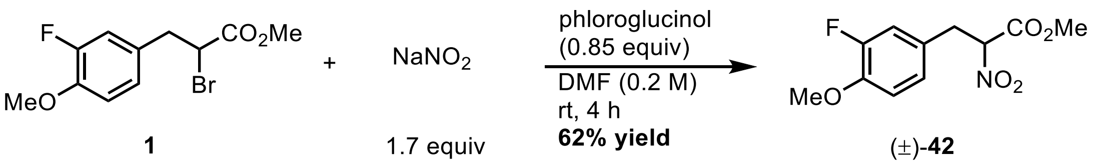

To a 4-mL borosilicate vial containing a Teflon-coated magnetic stirring bar were added phloroglucinol (11 mg, 0.085 mmol, 0.85 equiv), sodium nitrite (12 mg, 0.17 mmol, 1.7 equiv) and DMF (0.5 mL, c = 0.2 M). The vial was

sealed with a Teflon-lined screw cap and stirred at room temperature for 10 min. **1** (29.0 mg, 0.100 mmol, 1.00 equiv) was added and the mixture was stirred for 4 h. The resulting solution was diluted with ethyl acetate (10 mL), washed by water (10 mL) and brine (10 mL), dried over Na_2SO_4, and concentrated under reduced pressure. The residue was purified by flash column chromatography on silica gel (hexanes/EtOAc = 5:1) to afford (±)-**42** as a light yellow oil in 62% yield (16.1 mg).

R*f* = 0.20 (hexanes/EtOAc = 5:1).

NMR Spectroscopy:

^{1}H NMR (500 MHz, $CDCl_3$, 23 ºC, δ): 6.95 – 6.77 (m, 3H), 5.22 (dd, *J* = 9.6, 5.6 Hz, 1H), 3.80 (s, 3H), 3.77 (s, 3H), 3.42 (dd, *J* = 14.7, 9.6 Hz, 1H), 3.34 (dd, *J* = 14.8, 5.7 Hz, 1H).

^{13}C NMR (125 MHz, $CDCl_3$, 23 ºC, δ): 164.3, 152.3 (d, *J* = 247.2 Hz), 147.3 (d, *J* = 10.1 Hz), 126.6 (d, *J* = 6.3 Hz), 124.8 (d, *J* = 3.9 Hz), 116.6 (d, *J* = 18.6 Hz), 113.8 (d, *J* = 2.4 Hz), 88.9, 56.2, 53.8, 35.4.

^{19}F NMR (470 MHz, $CDCl_3$, 23 ºC, δ): –134.0 (dd, *J* = 11.7, 7.4 Hz).

HRMS-ESIpos (m/z) calc'd for $C_{11}H_{12}FNO_5Na$ $[M+Na]^+$, 280.0592; found, 280.0591; deviation: 0.2 ppm.

Synthesis of (±)-methyl 2-hydroxy-propanoate 43

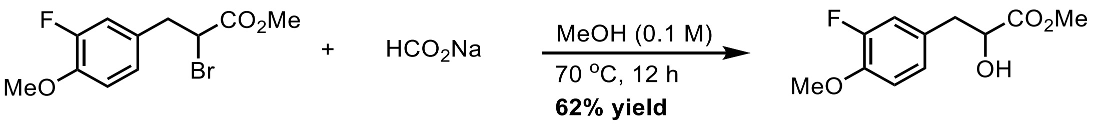

1   3.0 equiv   (±)-**43**

To a 4-mL borosilicate vial containing a Teflon-coated magnetic stirring bar were added **1** (29.0 mg, 0.100 mmol, 1.00 equiv), sodium formate (20 mg, 0.30 mmol, 3.0 equiv) and MeOH (1 mL, c = 0.1 M). The vial was sealed with a Teflon-lined screw cap and stirred at 70 ºC for 12 h. The resulting solution was diluted with ethyl acetate (10 mL), washed by water (10 mL) and brine (10 mL), dried over Na_2SO_4, and concentrated under reduced pressure. The residue was purified by flash column chromatography on silica gel (hexanes/EtOAc = 3:1) to afford (±)-**43** as a colorless oil in 62% yield (14.0 mg)

R*f* = 0.27 (hexanes/EtOAc = 3:1).

NMR Spectroscopy:

^{1}H NMR (500 MHz, $CDCl_3$, 23 ºC, δ): 7.01 – 6.93 (m, 2H), 6.90 (t, *J* = 8.3 Hz, 1H), 4.44 (dd, *J* = 6.6, 4.3 Hz, 1H), 3.89 (s, 3H), 3.80 (s, 3H), 3.08 (dd, *J* = 14.1, 4.3 Hz, 1H), 2.92 (dd, *J* = 14.1, 6.6 Hz, 1H), 2.77 (s, 1H).

^{13}C NMR (125 MHz, $CDCl_3$, 23 ºC, δ): 174.4, 152.1 (d, *J* = 245.5 Hz), 146.6 (d, *J* = 10.7 Hz), 129.2 (d, *J* = 6.2 Hz), 125.2 (d, *J* = 3.6 Hz), 117.2 (d, *J* = 18.1 Hz), 113.3 (d, *J* = 2.3 Hz), 71.1, 56.3, 52.6, 39.4.

^{19}F NMR (470 MHz, $CDCl_3$, 23 ºC, δ): –135.4 (dd, *J* = 12.2, 8.2 Hz).

HRMS-EI (m/z) calc'd for $C_{11}H_{13}O_4F$ $[M]^+$, 228.0792; found, 228.0791; deviation: 0.6 ppm.

Synthesis of (±)-methyl 2-phenylsulfonylpropanoate 44

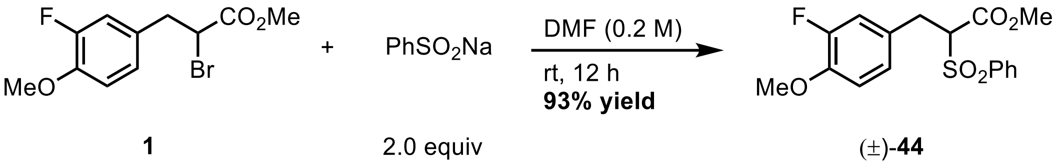

To a 4-mL borosilicate vial containing a Teflon-coated magnetic stirring bar were added **1** (29.0 mg, 0.100 mmol, 1.00 equiv), sodium benzosulfonate (32.8 mg, 0.200 mmol, 2.00 equiv) and DMF (0.5 mL, c = 0.2 M). The vial was sealed with a Teflon-lined screw cap and stirred at room temperature for 12 h. The resulting solution was diluted with ethyl acetate (10 mL), washed by water (10 mL) and brine (10 mL), dried over Na_2SO_4, and concentrated under reduced pressure. The residue was purified by flash column chromatography on silica gel (hexanes/EtOAc = 3:1) to afford (±)-**44** as a colorless solid in 93% yield (32.7 mg).

$\mathbf{R}_{f}$ = 0.47 (hexanes/EtOAc = 2:1).

NMR Spectroscopy:

^{1}H NMR (500 MHz, $CDCl_3$, 23 ºC, δ): 7.97 – 7.89 (m, 2H), 7.77 – 7.69 (m, 1H), 7.66 – 7.58 (m, 2H), 6.89 – 6.83 (m, 3H), 4.17 (dd, J = 11.8, 3.5 Hz, 1H), 3.85 (s, 3H), 3.56 (s, 3H), 3.34 (dd, J = 13.8, 3.5 Hz, 1H), 3.17 (dd, J = 13.8, 11.8 Hz, 1H).

^{13}C NMR (125 MHz, $CDCl_3$, 23 ºC, δ): 165.7, 152.2 (d, J = 246.7 Hz), 146.9 (d, J = 10.7 Hz), 136.9, 134.5, 129.3, 129.2, 128.2 (d, J = 6.3 Hz), 124.6 (d, J = 3.5 Hz), 116.5 (d, J = 18.5 Hz), 113.6 (d, J = 2.0 Hz), 72.1, 56.2, 53.0, 31.7.

^{19}F NMR (470 MHz, $CDCl_3$, 23 ºC, δ): –134.3 – –134.5 (m).

HRMS-ESIpos (m/z) calc'd for $C_{17}H_{17}FO_5SNa$ $[M+Na]^+$, 375.0673; found, 375.0673; deviation: 0.0 ppm.

Synthesis of (±)-methyl 2-acetylthiopropanoate 45

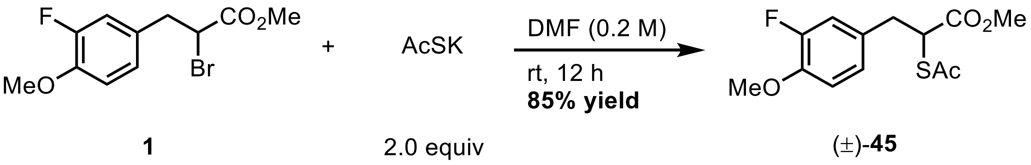

To a 4-mL borosilicate vial containing a Teflon-coated magnetic stirring bar were added **1** (29.0 mg, 0.100 mmol, 1.00 equiv), potassium thioacetate (22.8 mg, 0.200 mmol, 2.00 equiv) and DMF (0.5 mL, c = 0.2 M). The vial was sealed with a Teflon-lined screw cap and stirred at room temperature for 12 h. The resulting solution was diluted with ethyl acetate (10 mL), washed by water (10 mL) and brine (10 mL), dried over Na_2SO_4, and concentrated under reduced pressure. The residue was purified by flash column chromatography on silica gel (hexanes/EtOAc = 10:1) to afford (±)-**45** as a light yellow oil in 85% yield (24.5 mg)

$\mathbf{R}_{f}$ = 0.17 (hexanes/EtOAc = 10:1).

NMR Spectroscopy:

^{1}H NMR (500 MHz, $CDCl_3$, 23 ºC, δ): 7.00 – 6.92 (m, 2H), 6.89 (t, *J* = 8.3 Hz, 1H), 4.40 (dd, *J* = 8.2, 7.0 Hz, 1H), 3.88 (s, 3H), 3.70 (s, 3H), 3.18 (dd, *J* = 14.1, 8.2 Hz, 1H), 2.97 (dd, *J* = 14.1, 6.9 Hz, 1H), 2.35 (s, 3H).

^{13}C NMR (125 MHz, $CDCl_3$, 23 ºC, δ): 193.3, 171.3, 152.1 (d, *J* = 246.0 Hz), 146.6 (d, *J* = 10.7 Hz), 130.1 (d, *J* = 6.4 Hz), 124.8 (d, *J* = 3.5 Hz), 116.9 (d, *J* = 18.5 Hz), 113.3 (d, *J* = 2.2 Hz), 56.2, 52.7, 47.0, 37.1, 30.2.

^{19}F NMR (470 MHz, $CDCl_3$, 23 ºC, δ): –135.1 (dd, *J* = 11.8, 8.4 Hz).

HRMS-ESIpos (m/z) calc'd for $C_{13}H_{15}FO_4SNa$ $[M+Na]^+$, 309.0567; found, 309.0567; deviation: 0.1 ppm.

Synthesis of (±)-methyl 2-phenylthiopropanoate 46

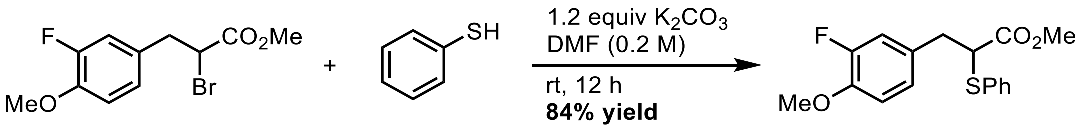

1 1.2 equiv (±)-**46**

To a 4-mL borosilicate vial containing a Teflon-coated magnetic stirring bar were added **1** (29.0 mg, 0.100 mmol, 1.00 equiv), benzenethiol (13 mg, 12 μL, 0.12 mmol, 1.2 equiv), K_2CO_3 (17 mg, 0.12 mmol, 1.2 equiv), and DMF (0.5 mL, c = 0.2 M). The vial was sealed with a Teflon-lined screw cap and stirred at room temperature for 12 h. The resulting solution was diluted with ethyl acetate (10 mL), washed by water (10 mL) and brine (10 mL), dried over Na_2SO_4, and concentrated under reduced pressure. The residue was purified by flash column chromatography on silica gel (hexanes/EtOAc = 10:1) to afford (±)-**46** as a colorless oil in 84% yield (27.0 mg)

R*f* = 0.22 (hexanes/EtOAc = 10:1).

NMR Spectroscopy:

^{1}H NMR (500 MHz, $CDCl_3$, 23 ºC, δ): 7.39 – 7.33 (m, 2H), 7.27 – 7.20 (m, 3H), 6.89 – 6.74 (m, 3H), 3.80 – 3.73 (m, 4H), 3.52 (s, 3H), 3.04 (dd, *J* = 14.0, 9.3 Hz, 1H), 2.91 (dd, *J* = 14.1, 6.3 Hz, 1H).

^{13}C NMR (125 MHz, $CDCl_3$, 23 ºC, δ): 171.9, 152.2 (d, *J* = 245.6 Hz), 146.5 (d, *J* = 10.8 Hz), 133.2, 132.9, 130.6 (d, *J* = 6.3 Hz), 129.0, 128.3, 124.8 (d, *J* = 3.5 Hz), 116.7 (d, *J* = 18.5 Hz), 113.4 (d, *J* = 2.7 Hz), 56.2, 52.2, 52.1, 37.1.

^{19}F NMR (470 MHz, $CDCl_3$, 23 ºC, δ): –135.1 (dd, *J* = 12.0, 8.0 Hz).

HRMS-EI (m/z) calc'd for $C_{17}H_{17}O_3SF$ $[M]^+$, 320.0877; found, 320.0877; deviation: –0.1 ppm.

Synthesis of (±)-methyl 2-phenylselanylpropanoate 47

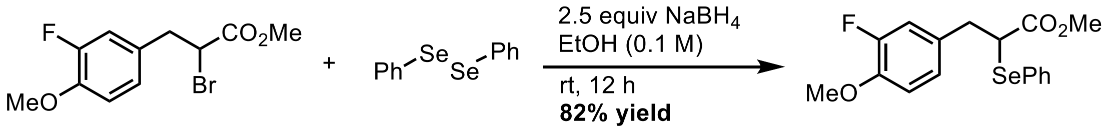

1 0.55 equiv (±)-**47**

To a 10-mL Schlenk tube containing a Teflon-coated magnetic stirring bar were added diphenyl diselenide (17 mg, 0.055 mmol, 0.55 equiv) and EtOH (1 mL, c = 0.1 M) under N_2. Sodium borohydride (9.5 mg, 0.25 mmol, 2.5 equiv) was added in three portions at 0 °C. The reaction was warmed to room temperature and stirred for 1 h. **1** (29.0 mg, 0.100 mmol, 1.00 equiv) was added and the mixture was stirred for 12 h. The resulting solution was diluted with ethyl acetate (10 mL), washed by water (10 mL) and brine (10 mL), dried over Na_2SO_4, and concentrated under reduced pressure. The residue was purified by flash column chromatography on silica gel (hexanes/EtOAc = 15:1) to afford (±)-**47** as a light yellow oil in 82% yield (30.1 mg).

R*f* = 0.3 (hexanes/EtOAc = 15:1).

NMR Spectroscopy:

^{1}H NMR (500 MHz, $CDCl_3$, 23 °C, δ): 7.57 (dd, *J* = 8.3, 1.4 Hz, 2H), 7.39 – 7.33 (m, 1H), 7.33 – 7.24 (m, 2H), 6.93 – 6.81 (m, 3H), 3.85 (s, 3H), 3.82 (dd, *J* = 9.8, 6.0 Hz, 1H), 3.57 (s, 3H), 3.16 (dd, *J* = 14.3, 9.8 Hz, 1H), 3.00 (dd, *J* = 14.2, 6.0 Hz, 1H).

^{13}C NMR (125 MHz, $CDCl_3$, 23 °C, δ): 172.6, 152.2 (d, *J* = 246.0 Hz), 146.4 (d, *J* = 10.6 Hz), 135.8, 131.5 (d, *J* = 6.1 Hz), 129.1, 128.8, 127.6, 124.6 (d, *J* = 3.6 Hz), 116.6 (d, *J* = 18.1 Hz), 113.4 (d, *J* = 2.2 Hz), 56.2, 52.1, 44.1, 37.3.

^{19}F NMR (470 MHz, $CDCl_3$, 23 °C, δ): –135.2 (dd, *J* = 12.0, 7.5 Hz).

HRMS-ESIpos (m/z) calc'd for $C_{17}H_{17}O_3FSeNa$ $[M+Na]^+$, 391.0219; found, 391.0216; deviation: –0.9 ppm.

Synthesis of (±)-methyl 2-diethoxyphosphorylpropanoate 48

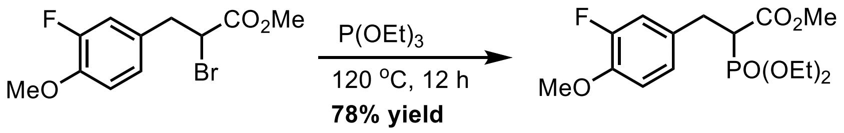

1 (±)-**48**

To a 2-mL borosilicate vial containing a Teflon-coated magnetic stirring bar were added **1** (29.0 mg, 0.100 mmol, 1.00 equiv) and triethyl phosphite (0.2 mL). The vial was sealed with a Teflon-lined screw cap and stirred at 150 °C for 10 h. The resulting solution was diluted with ethyl acetate (10 mL), washed by water (10 mL) and brine (10 mL), dried over Na_2SO_4, and concentrated under reduced pressure. The residue was purified by flash column chromatography on silica gel (hexanes/EtOAc = 1:5) to afford (±)-**48** as a colorless oil in 78% yield (27.2 mg).

R*f* = 0.32 (hexanes/EtOAc = 1:5).

NMR Spectroscopy:

^{1}H NMR (500 MHz, $CDCl_3$, 23 °C, δ): 6.96 – 6.83 (m, 3H), 4.24 – 4.14 (m, 4H), 3.87 (s, 3H), 3.67 (s, 3H), 3.31 – 3.06 (m, 3H), 1.36 (td, *J* = 7.1, 1.8 Hz, 6H).

^{13}C NMR (125 MHz, $CDCl_3$, 23 °C, δ): 168.9 (d, *J* = 4.7 Hz), 152.2 (d, *J* = 245.9 Hz), 146.4 (d, *J* = 10.7 Hz), 131.4 (dd, *J* = 16.4, 6.1 Hz), 124.2 (d, *J* = 3.5 Hz), 116.3 (d, *J* = 18.5 Hz), 113.5 (d, *J* = 2.2 Hz), 63.0 (d, *J* = 6.6 Hz), 62.9 (d, *J* = 6.7 Hz), 56.2, 52.5, 47.7 (d, *J* = 128.9 Hz), 31.9 (d, *J* = 4.3 Hz), 16.4 (d, *J* = 5.5 Hz),16.4

(d, J = 5.8 Hz).

^{19}F NMR (470 MHz, $CDCl_3$, 23 °C, δ): –135.1 (dd, J = 11.8, 8.3 Hz).

^{31}P NMR (203 MHz, $CDCl_3$, 23 °C, δ): 21.3.

HRMS-ESIpos (m/z) calc'd for $C_{17}H_{17}O_5FSNa$ $[M+Na]^+$, 375.0673; found, 375.0673; deviation: –0.0 ppm.

Synthesis of (±)-thiazolidinediones derivative 49

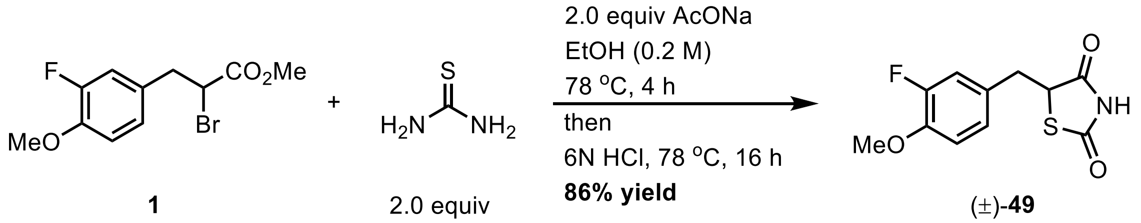

To a 4-mL borosilicate vial containing a Teflon-coated magnetic stirring bar were added **1** (29.0 mg, 0.100 mmol, 1.00 equiv), thiourea (15.2 mg, 0.200 mmol, 2.00 equiv), AcONa (16.4 mg, 0.200 mmol, 2.00 equiv), and ethanol (1 mL, c = 0.2 M). The vial was sealed with a Teflon-lined screw cap and stirred at 78 °C for 4 h. After cooling down to room temperature, 6N HCl (1 mL) was added and the mixture was stirred at 78 °C for 16 h. The resulting solution was diluted with ethyl acetate (10 mL), washed by water (10 mL) and brine (10 mL), dried over Na_2SO_4, and concentrated under reduced pressure. The residue was purified by flash column chromatography on silica gel (hexanes/EtOAc = 3:1) to afford (±)-**49** as a colorless solid in 86% yield (22.0 mg).

R_f = 0.30 (hexanes/EtOAc = 3:1).

NMR Spectroscopy:

^{1}H NMR (500 MHz, $CDCl_3$, 23 °C, δ): 8.61 (s, 1H), 6.94 – 6.80 (m, 3H), 4.43 (dd, J = 9.3, 4.0 Hz, 1H), 3.81 (s, 3H), 3.36 (dd, J = 14.3, 4.0 Hz, 1H), 3.04 (dd, J = 14.3, 9.3 Hz, 1H).

^{13}C NMR (125 MHz, $CDCl_3$, 23 °C, δ): 174.1, 170.3, 152.2 (d, J = 246.8 Hz), 147.2 (d, J = 10.6 Hz), 128.4 (d, J = 6.2 Hz), 125.1 (d, J = 3.5 Hz), 116.9 (d, J = 18.5 Hz), 113.6 (d, J = 2.4 Hz), 56.3, 53.3, 37.6.

^{19}F NMR (470 MHz, $CDCl_3$, 23 °C, δ): –134.1 (dd, J = 11.7, 8.1 Hz).

HRMS-EI (m/z) calc'd for $C_{11}H_{10}NO_3SF$ $[M]^+$, 255.0360; found, 255.0361; deviation: –0.3 ppm.

Synthesis of (±)-2*H*-benzo[*b*]-[1,4]thiazin-3(4*H*)-one 50

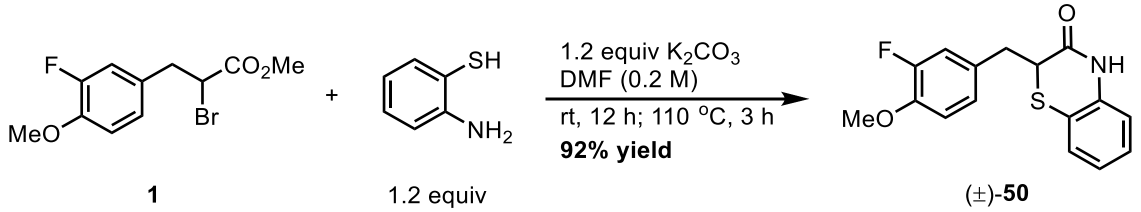

To a 4-mL borosilicate vial containing a Teflon-coated magnetic stirring bar were added **1** (29.0 mg, 0.100 mmol,

1.00 equiv), 2-aminobenzenethiol (15 mg, 13 μL, 0.12 mmol, 1.2 equiv), K_2CO_3 (17 mg, 0.12 mmol, 1.2 equiv), and DMF (0.5 mL, c = 0.2 M). The vial was sealed with a Teflon-lined screw cap, stirred at room temperature for 12 h and then 110 °C for 3 h. The resulting solution was diluted with ethyl acetate (10 mL), washed by water (10 mL) and brine (10 mL), dried over Na_2SO_4, and concentrated under reduced pressure. The residue was purified by flash column chromatography on silica gel (hexanes/EtOAc = 2:1) to afford (±)-**50** as a colorless solid in 92% yield (27.9 mg).

R*f* = 0.23 (hexanes/EtOAc = 2:1).

NMR Spectroscopy:

^{1}**H NMR** (500 MHz, $CDCl_3$, 23 °C, δ): 9.26 (s, 1H), 7.23 (dd, *J* = 7.7, 1.3 Hz, 1H), 7.12 (td, *J* = 7.7, 1.4 Hz, 1H), 6.96 (td, *J* = 7.6, 1.3 Hz, 1H), 6.89 – 6.77 (m, 4H), 3.77 (s, 3H), 3.53 (dd, *J* = 9.5, 5.5 Hz, 1H), 3.14 (dd, *J* = 14.2, 5.4 Hz, 1H), 2.70 (dd, *J* = 14.2, 9.5 Hz, 1H).

^{13}**C NMR** (125 MHz, $CDCl_3$, 23 °C, δ): 167.7, 152.1 (d, *J* = 245.7 Hz), 146.6 (d, *J* = 10.3 Hz), 135.9, 129.9 (d, *J* = 6.4 Hz), 128.3, 127.3, 125.0 (d, *J* = 3.6 Hz), 124.1, 118.3, 117.1 (d, *J* = 10.4 Hz), 117.0 (d, *J* = 7.7 Hz), 113.3 (d, *J* = 2.1 Hz), 56.2, 44.3, 34.7.

^{19}**F NMR** (470 MHz, $CDCl_3$, 23 °C, δ): –135.1 (dd, *J* = 12.0, 7.9 Hz).

HRMS-EI (m/z) calc'd for $C_{16}H_{14}NO_2SF$ $[M]^+$, 303.0724; found, 303.0726; deviation: –0.8 ppm.

Reaction condition optimization

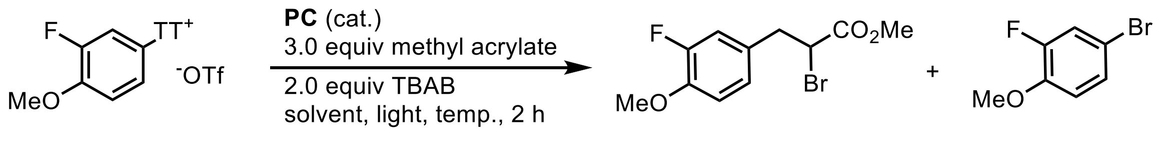

To a 4-mL borosilicate vial containing a Teflon-coated magnetic stirring bar were added **TT-1** (49.0 mg, 0.100 mmol, 1.00 equiv), photocatalyst, and tetrabutylammonium bromide (TBAB, 64 mg, 0.40 mmol, 2.0 equiv). The vial was transferred into a nitrogen-filled glovebox. Dry solvent (1 mL, c = 0.2 M) and methyl acrylate (27 μL, 26 mg, 0.30 mmol, 3.0 equiv) were added. The vial was sealed with a Teflon-lined screw cap, removed from the glovebox, and irradiated under LEDs for 2 h. Subsequently, the reaction mixture was concentrated under reduced pressure. The resulting residue was dissolved in $CDCl_3$ (0.5 mL); internal standard CH_2Br_2 (17.4 mg, 0.1 mmol, 1.0 equiv.) was added and the mixture was analyzed by ^{1}H NMR spectroscopy.

Table S1. Screening of photocatalysts, solvents and reaction temperature

Entry	photocatalysts	Solvents	T/°C	Conv. of **TT-1**(%)	Yield of (±)-**1** (%)	Yield of **1'** (%)
1[a]	1 mol% Ir(dFppy)$_3$	MeCN	33	95	42	46

2[b]	1 mol% Ir(dFppy)$_3$	MeCN	33	86	57	14
3	1 mol% Ir(dFppy)$_3$	MeCN	33	32	18	3
4	1 mol% Ru(bpy)$_3$Cl$_2$•6H$_2$O	MeCN	33	5	2	1
5	1 mol% [Ir{dFCF$_3$ppy}$_2$(dtbpy)]PF$_6$	MeCN	33	79	44	5
6[c]	5 mol% **TXO**	MeCN	33	>99	58	9
7	5 mol% **PTH**	MeCN	33	>99	59	5
8	–	MeCN	33	2	0	0
9[d]	5 mol% **PTH**	MeCN	33	0	0	0
10	5 mol% **PTH**	MeCN	0	>99	63	3
11	5 mol% **PTH**	MeCN	–20	94	66	3
**12[e]**	**5 mol% PTH**	**MeCN**	**–20**	**>99**	**74(68)**	**3**
13[e]	5 mol% **PTH**	Acetone	–20	>99	72	7
14[e]	5 mol% **PTH**	AcOMe	–20	94	69	3

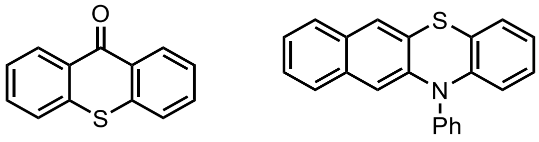

TXO **PTH**

[a]10 mol% Cu(MeCN)$_4$BF$_4$ was added; [b]10 mol% PhenCuCl$_2$ was added; [c]390 nm LED was used; [d]without light; [e]white LED

Table S2. Other (pseudo)halides

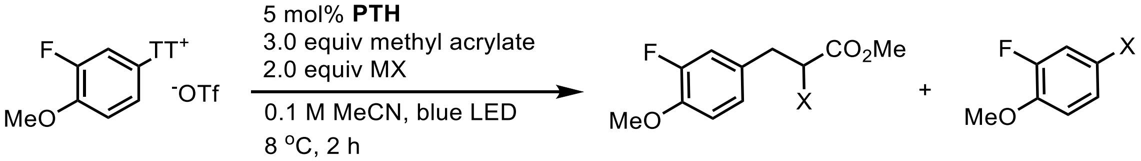

TT-1 (±)-**SX** **ArX**

Entry	(Pseudo)halides	Conv. of **TT-1**(%)	Yield of (±)-**SX** (%)	Yield of **ArX** (%)
1	TBAB	>99	62	4
2	NaBr	>99	53	5
3	KBr	88	40	3
4	HBr	92	41	9
5	TBACl	>99	21	0
6	TBAI	65	18	33
7	NaN$_3$	42	0	0
8	KSCN	>99	26	1

Table S3. Trials on hydroarylation

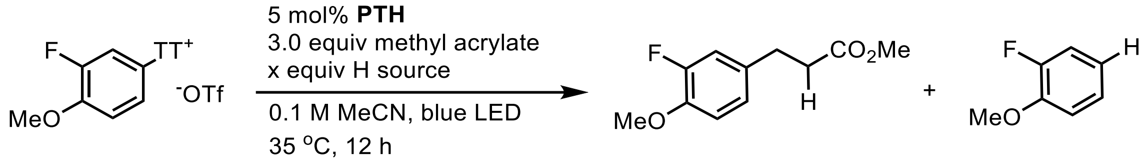

TT-1 **S1** **ArH**

Entry	H source (equiv)	Conv. of **TT-1**(%)	Yield of **S1** (%)	Yield of **ArH** (%)
1	DIPEA (2.0)	>99	15	53
2	HCO_2Na (3.0)	>99	0	62
3[a]	HCO_2Na (3.0)	>99	12	60
4[a]	Hantz ester (1.2)	>99	56	32

[a]DMSO/H_2O = 50/1 was used as the solvent, 5 mol% cyclohexenethiol.

Unsuccessful substrates

Aryl coupling partners:

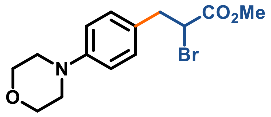

4%

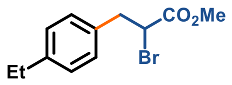

10%

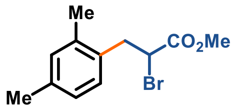

17%

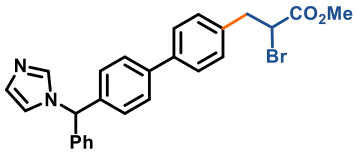

5%

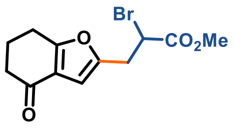

21%

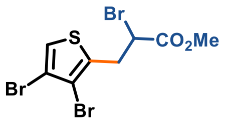

6%

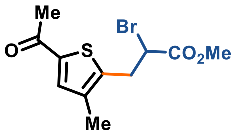

25%

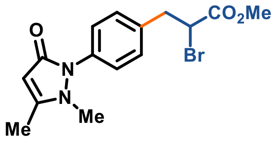

<5%

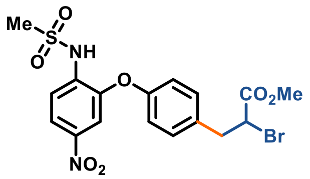

17%

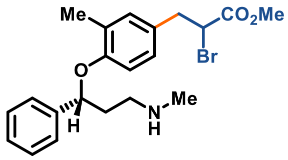

<5%

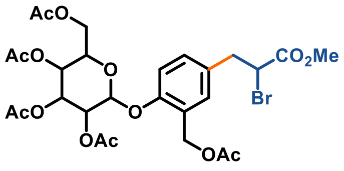

<5%

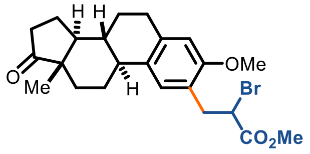

<5%

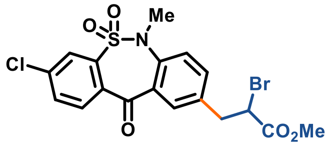

14%

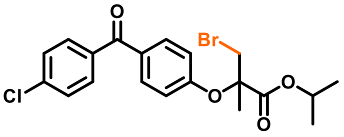

64%

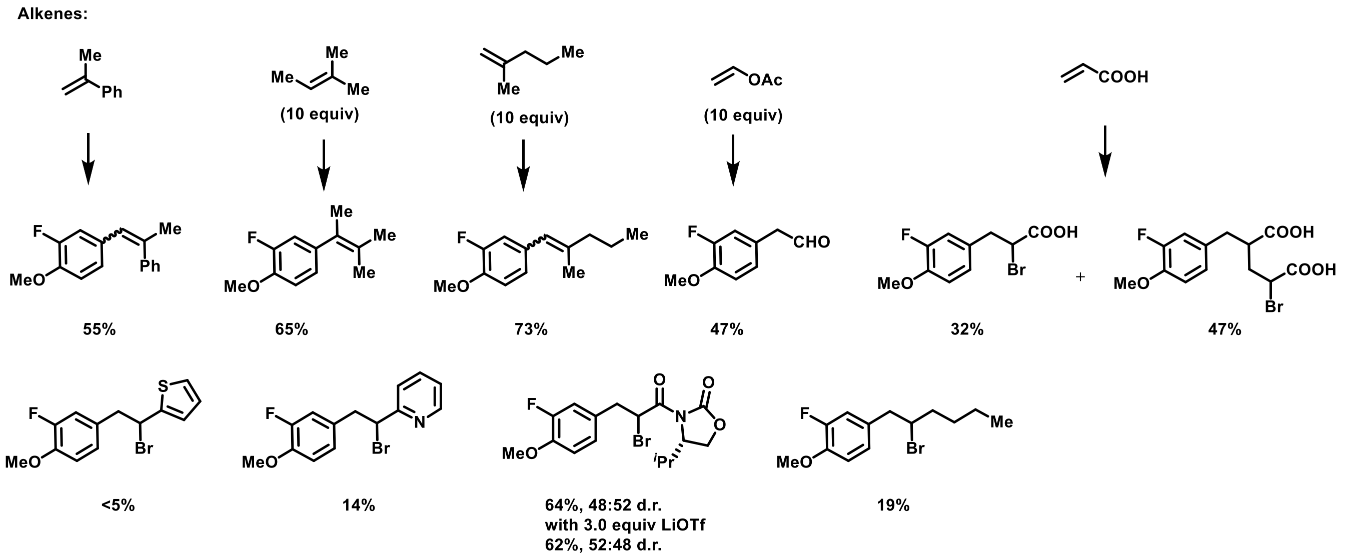

Mechanistic studies

UV-vis Absorption Spectrum

All UV-Vis spectrum measurements were recorded on a Shimadzu UV-Vis spectrophotometer UV-2600 with temperature controller using a quartz cuvette (10 × 10 mm, 3.5 mL) in acetonitrile as solvent.

UV-Vis spectra of the reaction mixture were measured with the following concentrations:

TT-1 (75 mg, 0.15 mmol, c = 50 mM) in MeCN (3 mL)

PTH (1.6 mg, 5.0 μmol, c = 1.7 mM) in MeCN (3 mL)

TT-1 (75 mg, 0.15 mmol, c = 50 mM) and **PTH** (1.6 mg, 5.0 μmol, c = 1.7 mM) in MeCN (3 mL)

TT-1 (75 mg, 0.15 mmol, c = 50 mM) and TBAB (64 mg, 0.20 mmol, 67 mM) in MeCN (3 mL)

TT-1 (75 mg, 0.15 mmol, c = 50 mM), **PTH** (1.6 mg, 5.0 μmol, c = 1.7 mM) and TBAB (64 mg, 0.20 mmol, 67 mM) in MeCN (3 mL)

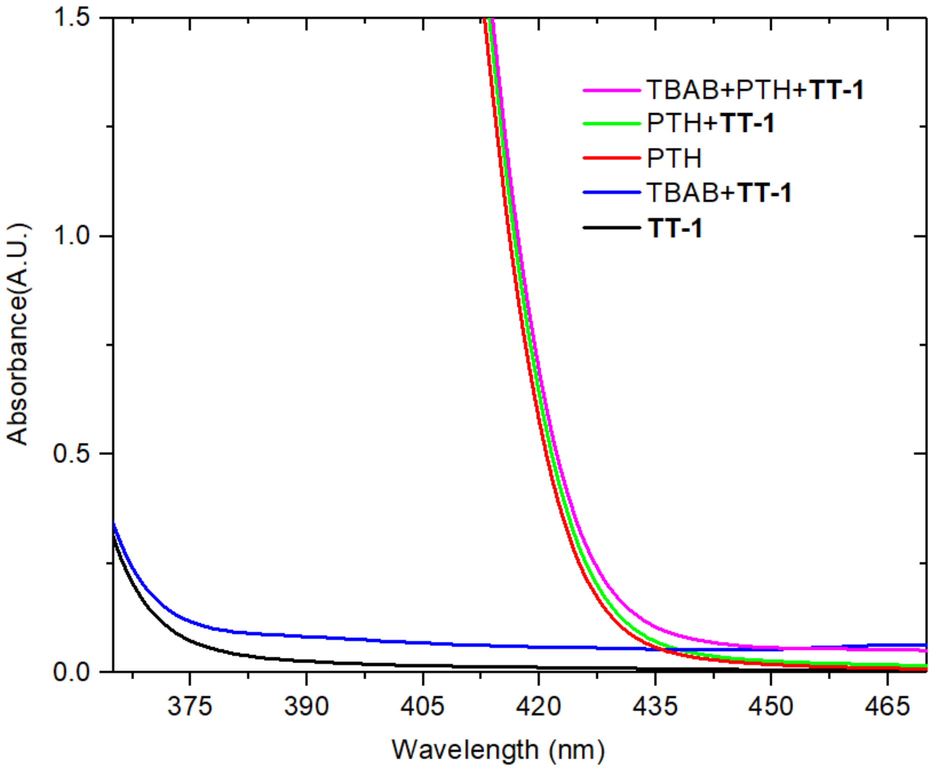

Figure S1. UV-Vis absorption spectra of the reaction mixture in MeCN

An electron-donor-acceptor (EDA) complex between electron-deficient **TT-1** and electron-rich **PTH** was not observed.

Stern-Volmer Luminescence Quenching Studies

Visible light luminescence intensities were recorded using an Edinburgh Instruments FS5 spectrofluorometer. All luminescence measurements were recorded using a screw-top quartz cuvette (Hellma fluorescence quartz cuvette, 10 x 10 mm, 3.5 mL). All solutions of **PTH**, **TT-1**, TBAB, and methyl acrylate were prepared in MeCN in a nitrogen-filled glovebox. The solutions were transferred to the screw-top cuvette inside the glovebox, the cuvette was sealed, and then, brought out of the glovebox for visible light luminescence measurements.

In a typical procedure, **TT-1** (588 mg, 1.20 mmol) was dissolved and diluted to a final volume of 10 mL (c = 0.12 M) with a stock solution of **PTH** in MeCN (c = 100 µM). Serial dilution of this 0.12 M **TT-1** solution was carried out by dilution of 7 mL of the 0.12 M **TT-1** solution to 10 mL (84 mM) with the 100 µM stock solution of **PTH**. All subsequent solutions were prepared by dilution of 7 mL of the preceding solution to a final volume of 10 mL. All solutions were excited at 400 nm and the emission was measured from 420 to 600 nm.

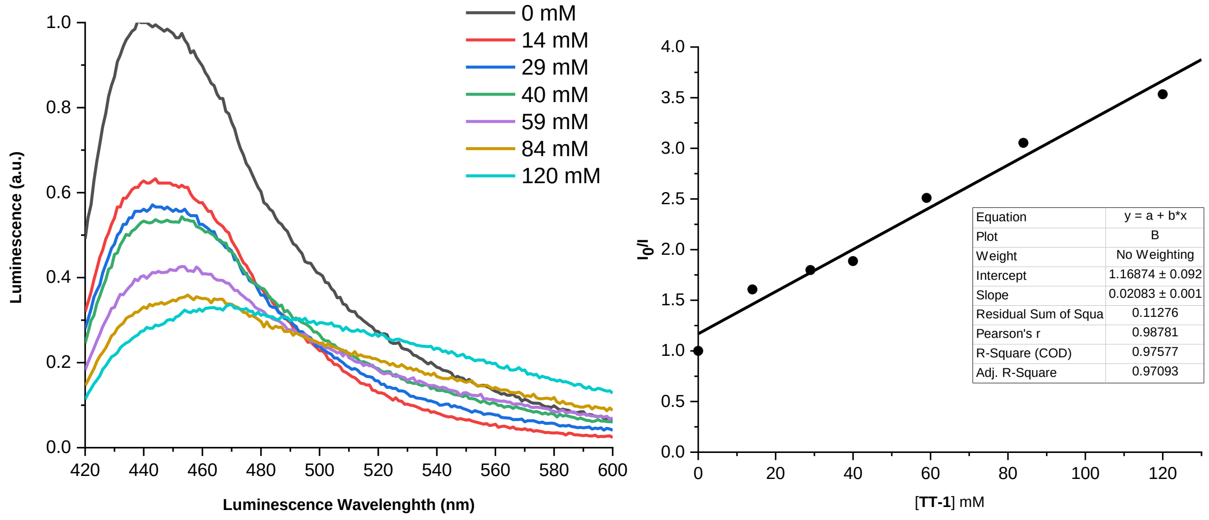

Figure S2. Emission spectra and Stern–Volmer plot for **PTH** luminescence quenching by **TT-1** (K_{sv} = 20.8, k_q = 1.6 x 10^9).

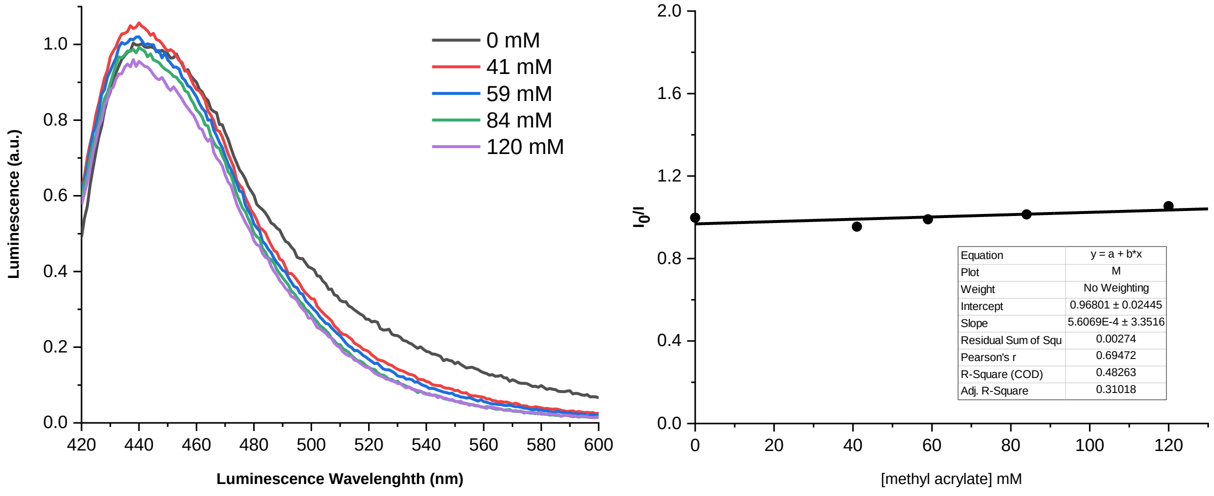

Figure S3. Emission spectra and Stern–Volmer plot for **PTH** luminescence quenching by methyl acrylate (K_{sv} = 0.56, k_q = 4.3 x 10^7).

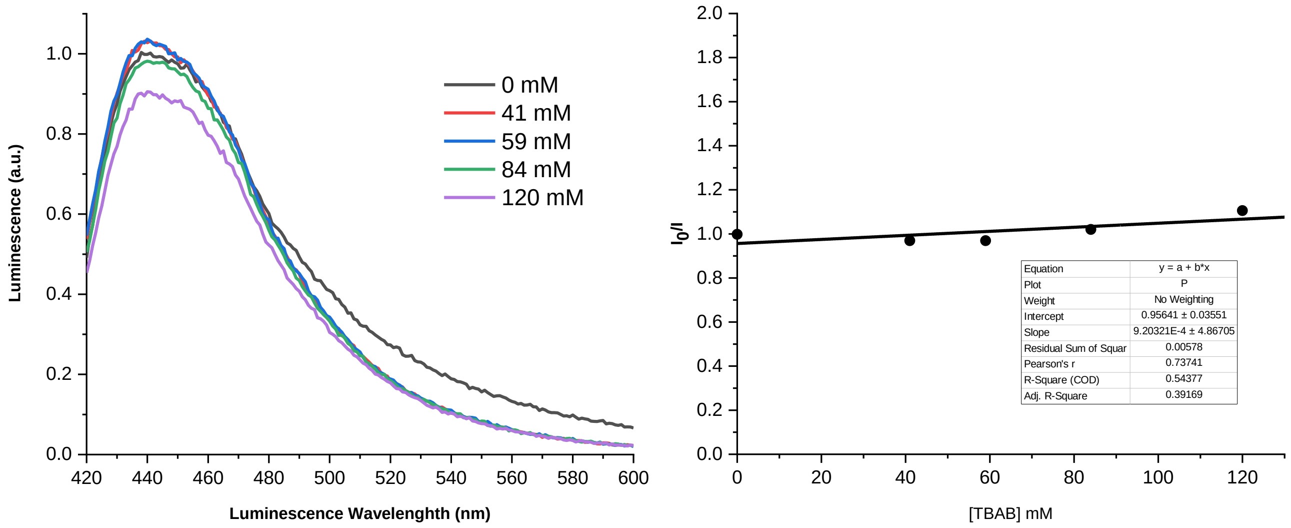

Figure S4. Emission spectra and Stern–Volmer plot for **PTH** luminescence quenching by TBAB (K_{sv} = 0.92, k_q = 7.1 x 10^7).

Synthesis of $PTH^{•+}$

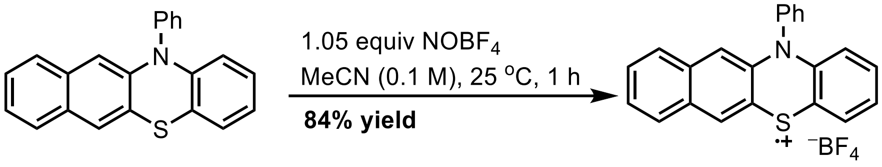

PTH **$PTH^{•+}$** $^-BF_4$

In a nitrogen-filled glove box, **PTH** (160 mg, 0.500 mmol, 1.00 equiv) was added to nitrosonium tetrafluoroborate (65.0 mg, 0.550 mmol, 1.05 equiv) in acetonitrile (5 mL, c = 0.1 M) to produce a dark purple solution. The glove box was purged while the reaction mixture was stirred for 1 h, at which point, diethyl ether (15 mL) was added to the stirred reaction mixture. The precipitate was collected by vacuum filtration and washed with diethyl ether until the filtrate was colorless (3 × 10 mL). The filter cake was transferred to a 20 mL scintillation vial and put under vacuum for 5 h, affording the title compound as a free-flowing black-purple solid in 84% yield (134 mg).

IR (neat, cm-1): 3072, 1543, 1485, 1339, 1285, 1204, 1032, 903,882, 787, 754, 698.

UV (nm): 555, 426.

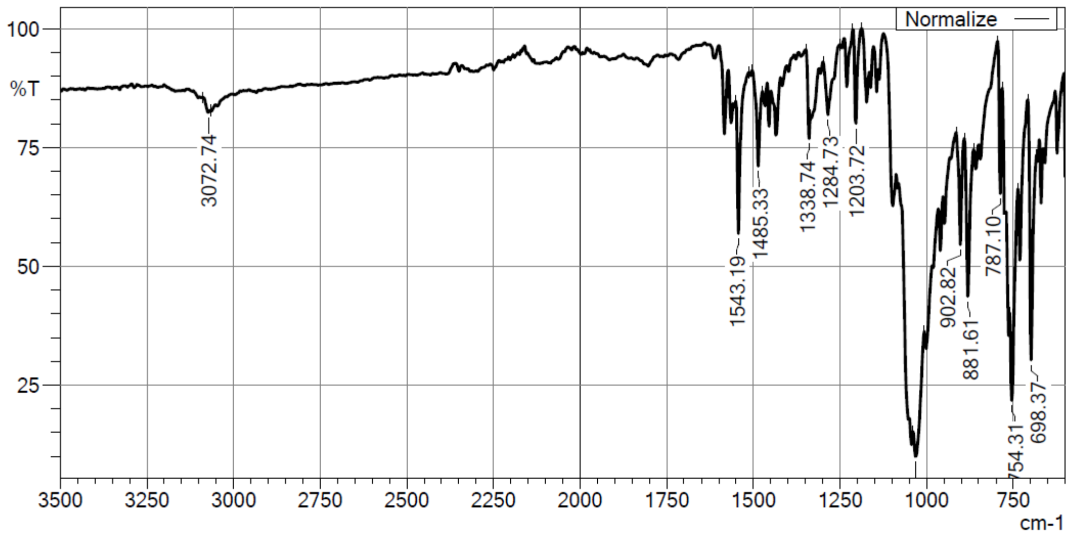

Figure S5. IR spectrum of **PTH**$^{\bullet+}$

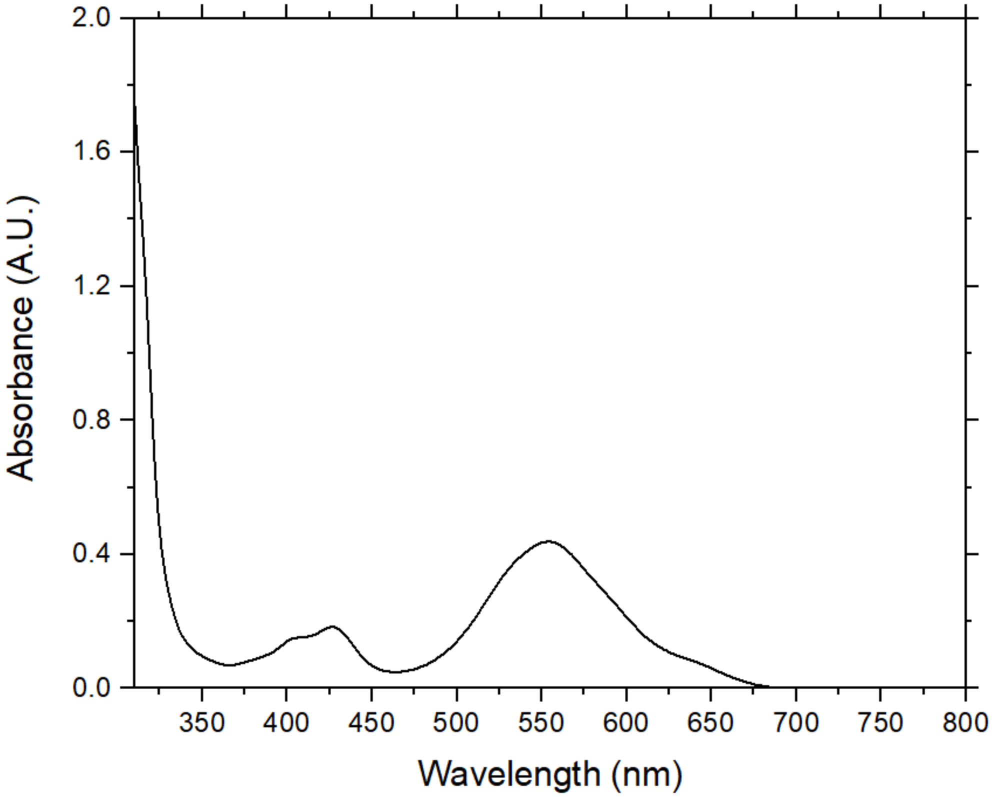

Figure S6. UV spectrum of **PTH**$^{\bullet+}$ in MeCN (0.17 mM)

Stoichiometric reaction between PTH•+ and TBAB

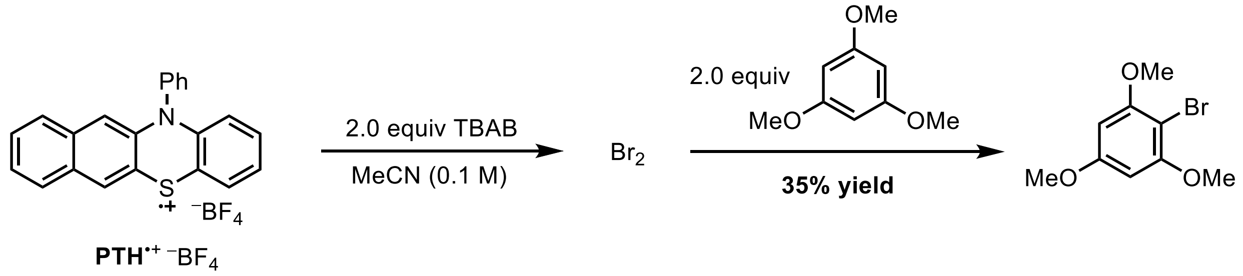

In a nitrogen-filled glove box, **PTH•+** (8.1 mg, 25 μmol, 1.0 equiv) and tetrabutylammonium bromide (TBAB, 16 mg, 50 μmol, 2.0 equiv) were mixed in acetonitrile (0.5 mL, c = 0.05 M). The color of the solution changed immediately from dark purple to brown. Then 1,3,5-trimethoxbenzene (8.4 mg, 50 μmol, 2.0 equiv) was added, and the reaction mixture was stirred for 1 h. Subsequently, the reaction mixture was concentrated under reduced pressure. The resulting residue was dissolved in $CDCl_3$ (0.5 mL); internal standard CH_2Br_2 (10.0 mg) was added and the mixture was analyzed by ^1H NMR spectroscopy and GC-MS. The ^1H NMR yield of 2-bromo-1,3,5-trimethoxybenzene was determined to be 35% by the integral of the protons (3.82 ppm) on methoxy group. The ^1H NMR yield of **PTH** was determined to be 70% by the integral of the proton (6.37 ppm) ortho to aniline group.

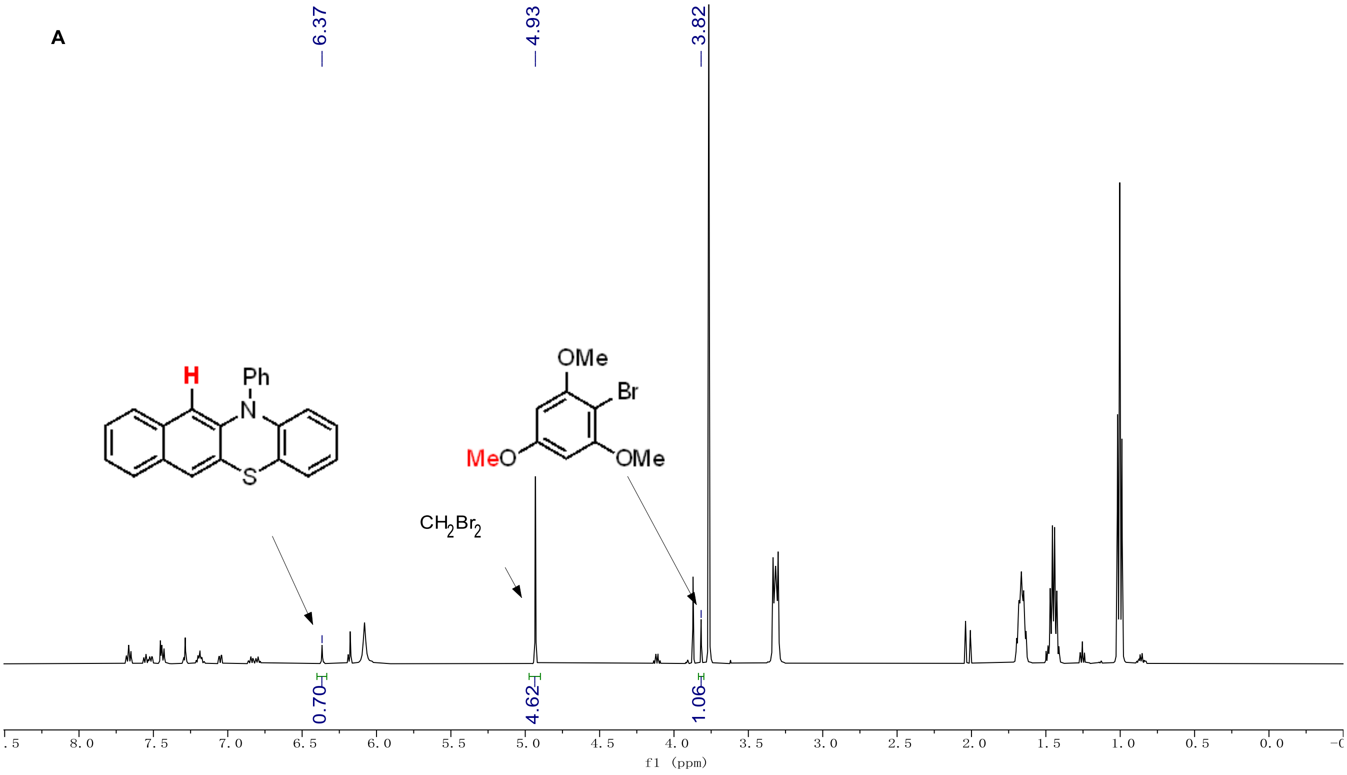

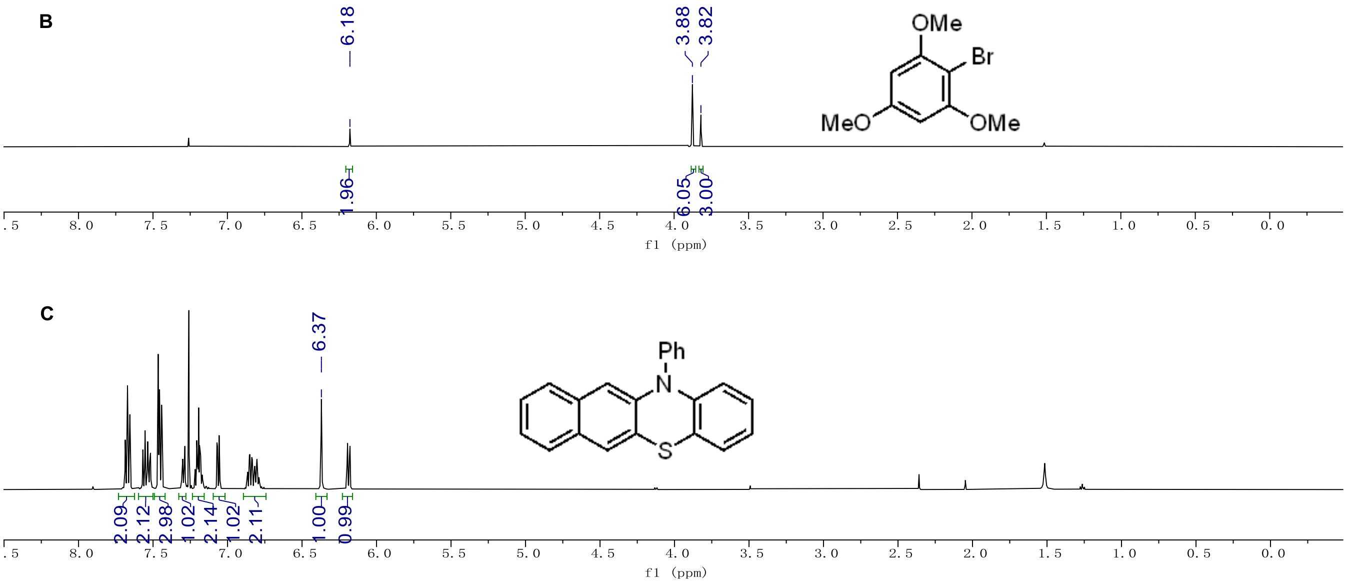

Figure S7. **A.** Reaction mixture of **PTH**$^{•+}$, TBAB and 1,3,5-trimethoxbenzene; **B**. ^{1}H NMR spectrum of 2-bromo-1,3,5-trimethoxybenzene; **C**. ^{1}H NMR spectrum of **PTH**

Free bromine trapping experiments

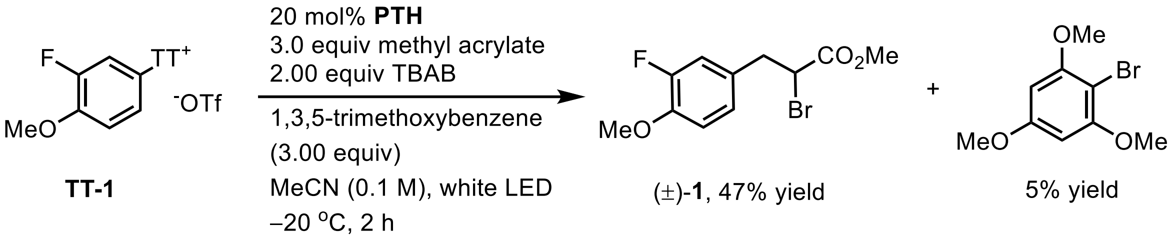

To a 4-mL borosilicate vial containing a Teflon-coated magnetic stirring bar were added **TT-1** (49.0 mg, 0.100 mmol, 1.00 equiv), **PTH** (6.5 mg, 20 μmol, 20 mol%), 1,3,5-trimethoxybenzene (50 mg, 0.30 mmol, 3.0 equiv), and tetrabutylammonium bromide (TBAB, 64 mg, 0.20 mmol, 2.0 equiv). The vial was transferred into a nitrogen-filled glovebox. Dry MeCN (1 mL, c = 0.1 M) and methyl acrylate (26 mg, 27 μL, 0.30 mmol, 3.0 equiv) were added. The vial was sealed with a Teflon-lined screw cap, removed from the glovebox, and transferred to a cryocooler precooled at –20 °C where the reaction mixture was stirred for 5 min without irradiation and then irradiated with white LEDs for 2 h. Subsequently, the reaction mixture was concentrated under reduced pressure. The resulting residue was dissolved in $CDCl_3$ (0.5 mL); internal standard CH_2Br_2 (15.0 mg) was added and the mixture was analyzed by ^{1}H NMR spectroscopy and GC-MS. The ^{1}H NMR yield of 2-bromo-1,3,5-trimethoxybenzene was determined to be 5% by the integral of the protons (3.82 ppm) on methoxy group. The ^{1}H NMR yield of (±)-**1** was determined to be 47% by the integral of the α-proton (4.36 ppm) of ester group.

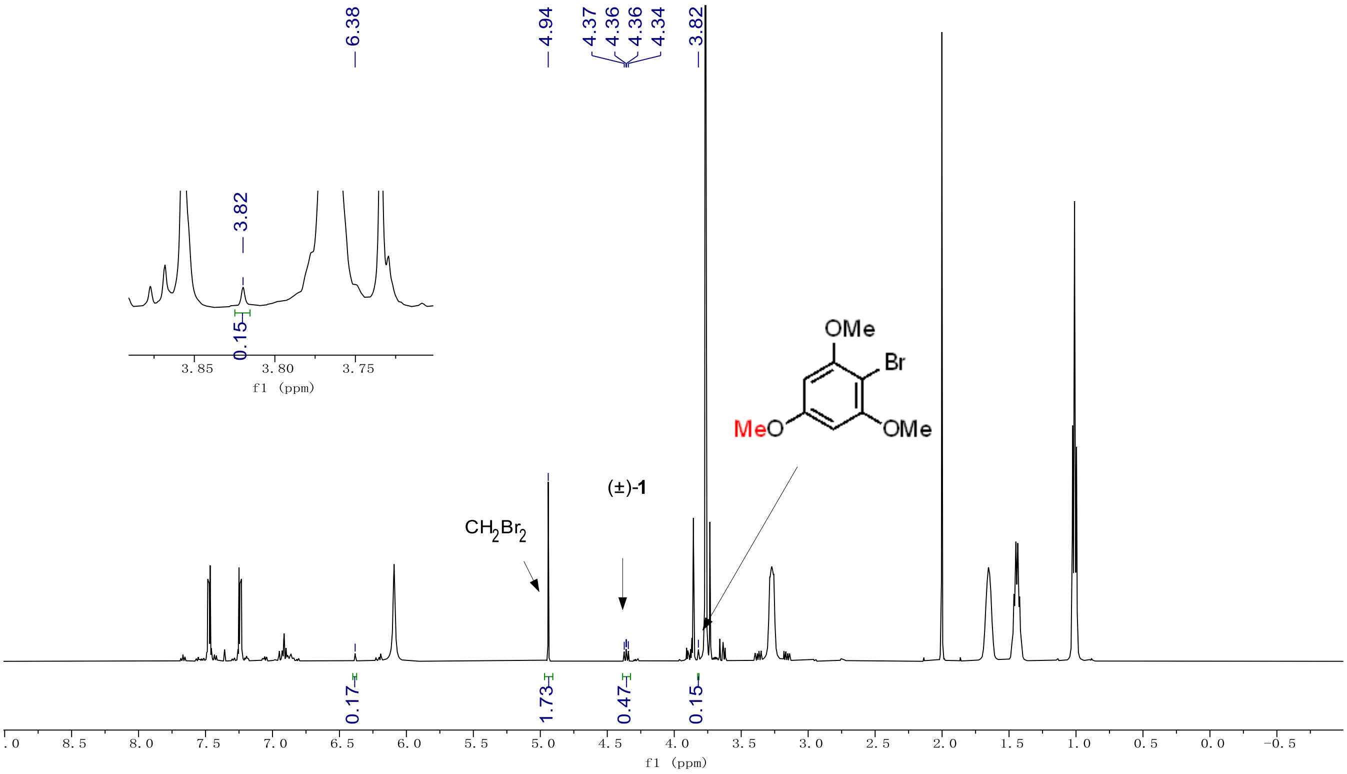

Figure S8. Reaction mixture under standard conditions in the presence of 1,3,5-trimethoxybenzene

SPECTROSCOPIC DATA

^{1}H NMR of (±)-2-bromo-arylpropanoate 1

$CDCl_3$, 23 ºC

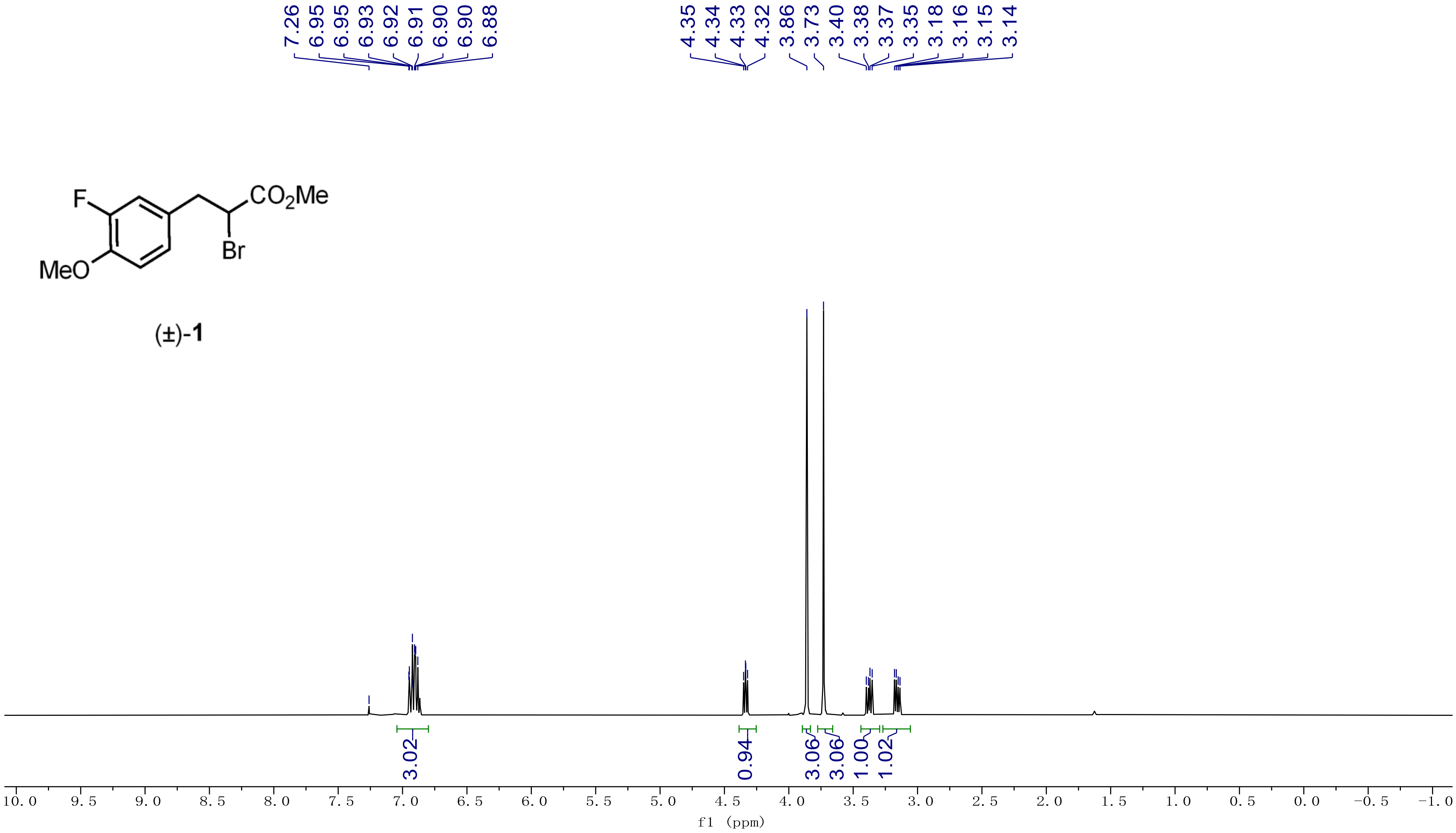

^{13}C NMR of (±)-2-bromo-arylpropanoate 1

$CDCl_3$, 23 ºC

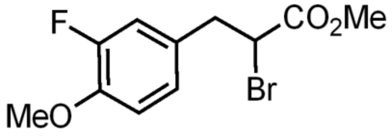

(±)-**1**

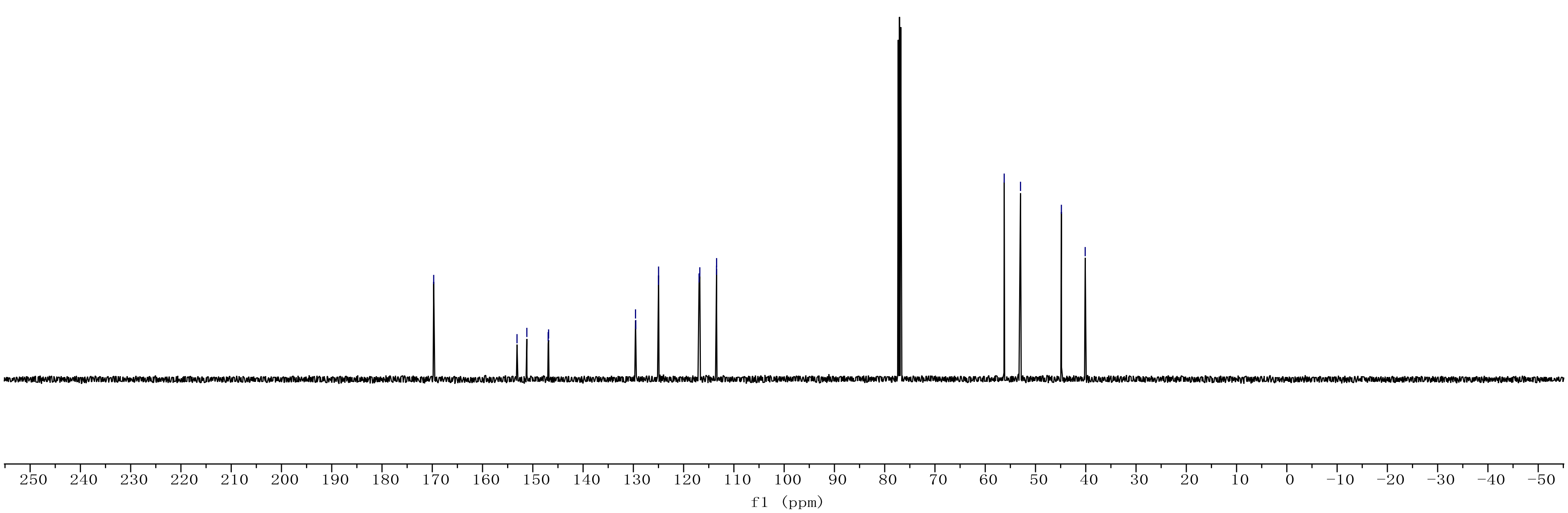

^{19}F NMR of (±)-2-bromo-arylpropanoate 1

$CDCl_3$, 23 ºC

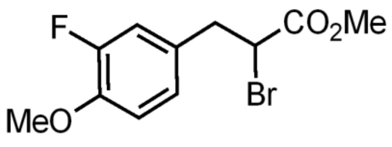

(±)-**1**

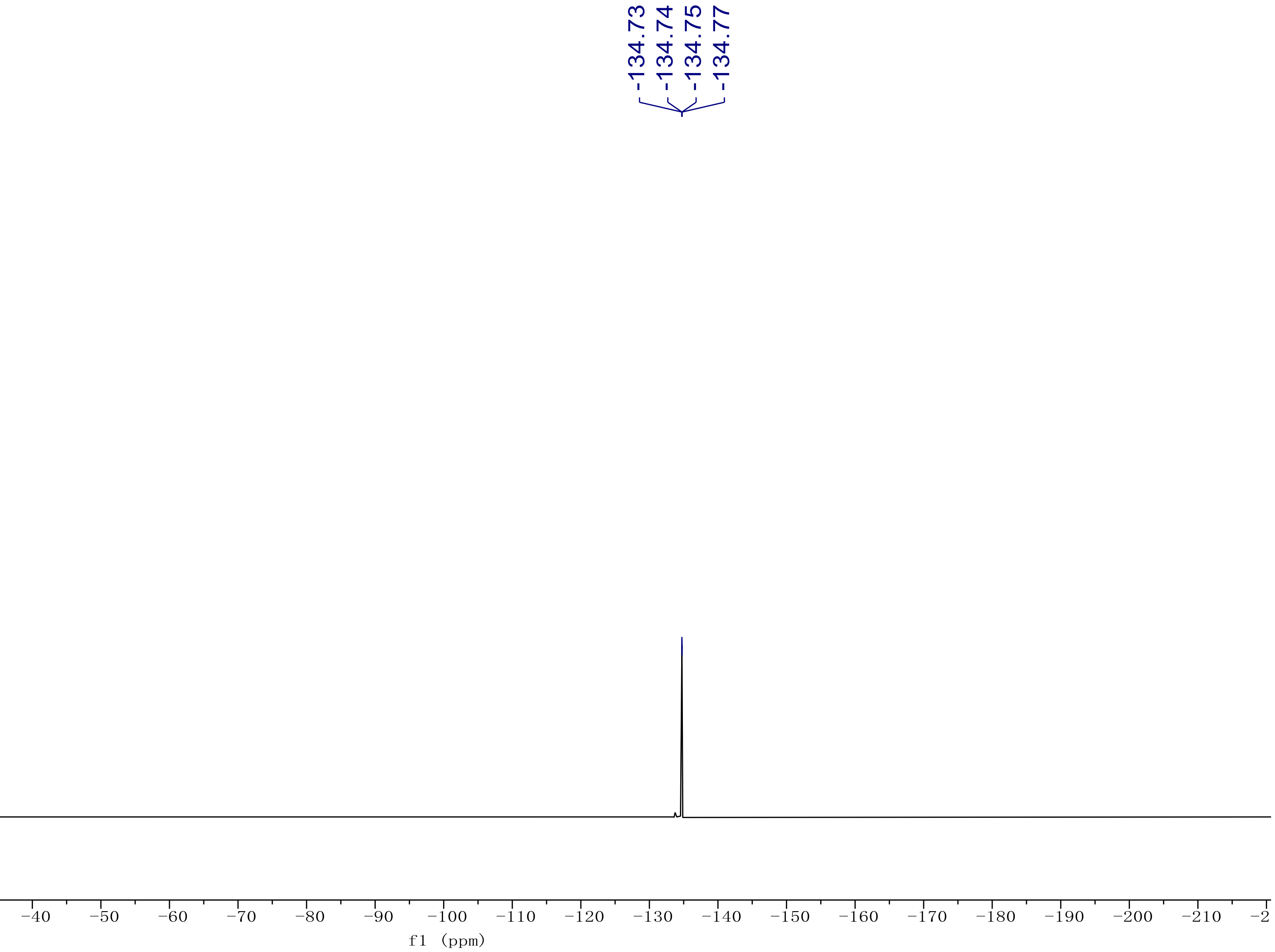

^{1}H NMR of (±)-2-bromo-arylpropanoate 2

$CDCl_3$, 23 ºC

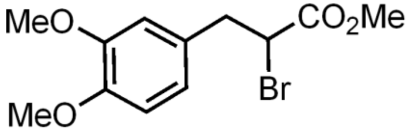

(±)-**2**

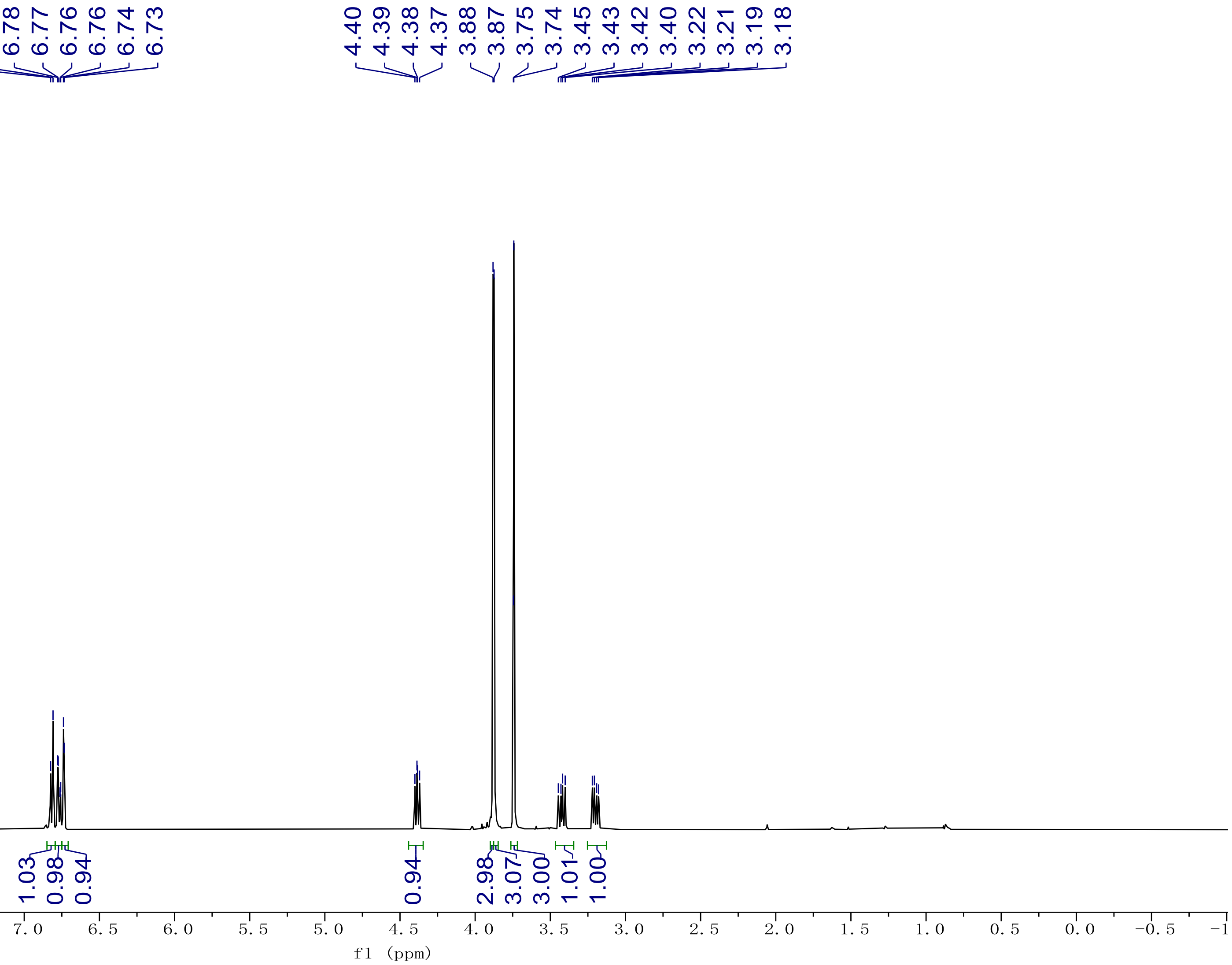

^{13}C NMR of (±)-2-bromo-arylpropanoate 2

$CDCl_3$, 23 ºC

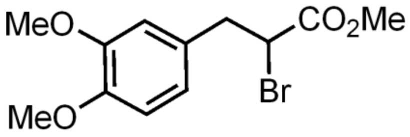

(±)-**2**

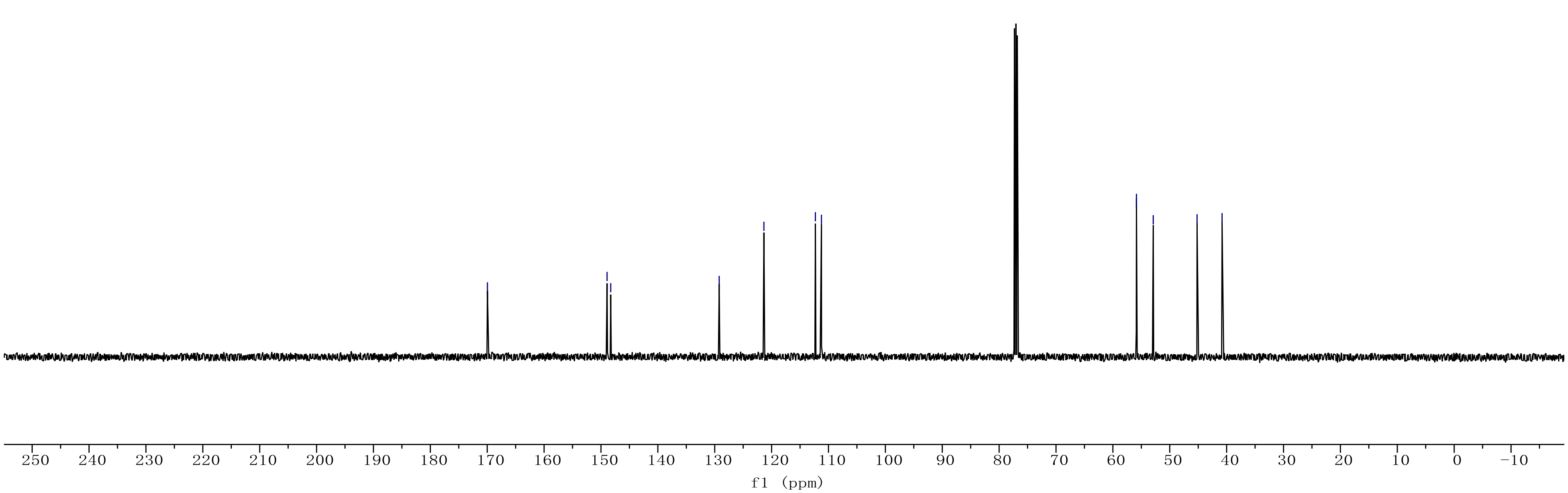

^{1}H NMR of (±)-2-bromo-arylpropanoate 3

$CDCl_3$, 23 ºC

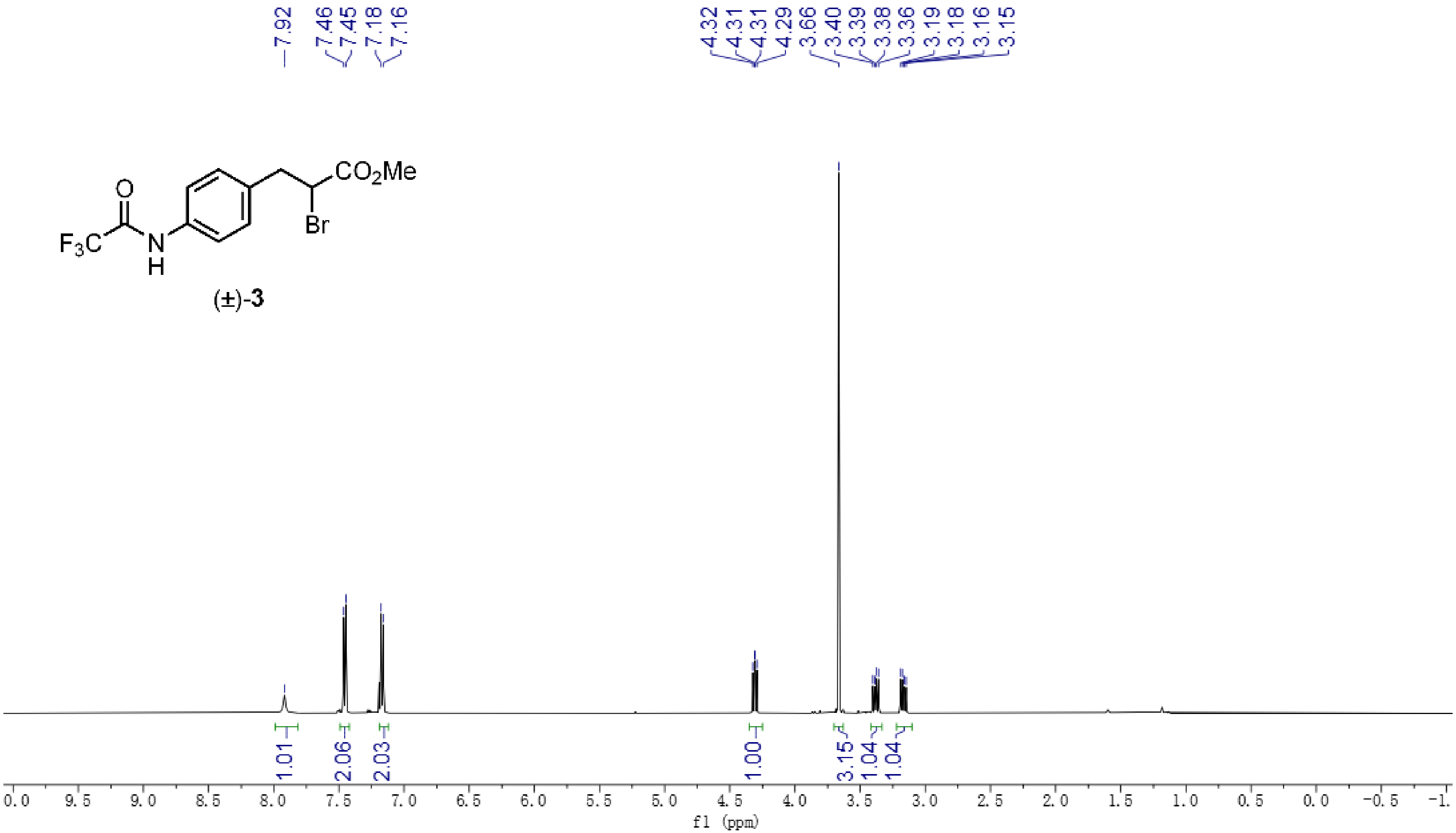

(±)-**3**

^{13}C NMR of (±)-2-bromo-arylpropanoate 3

$CDCl_3$, 23 ºC

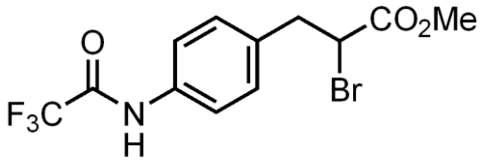

(±)-**3**

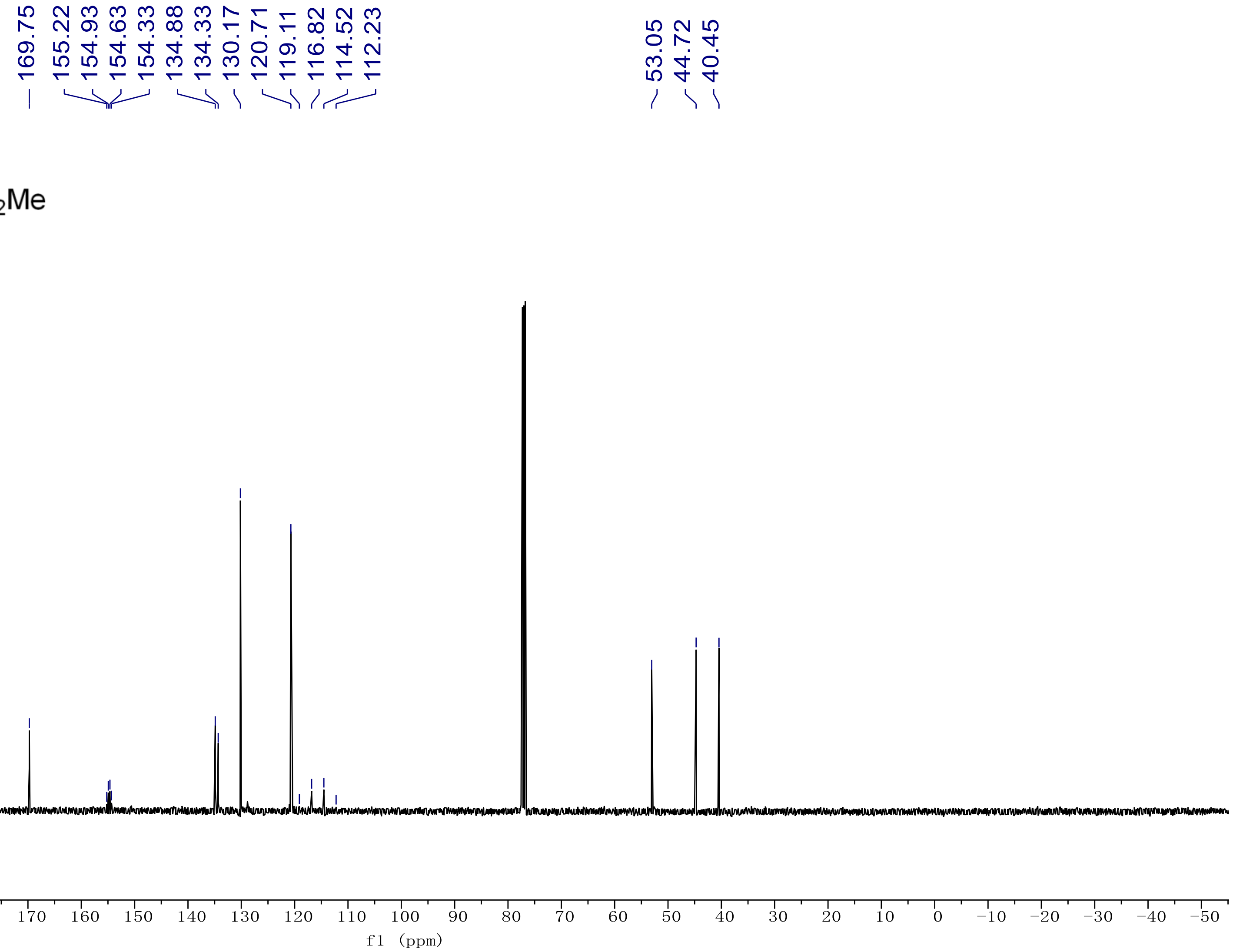

^{19}F NMR of (±)-2-bromo-arylpropanoate 3

$CDCl_3$, 23 ºC

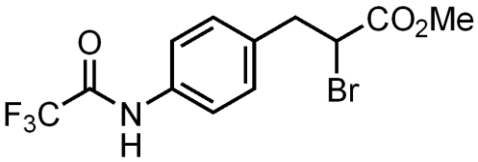

(±)-**3**

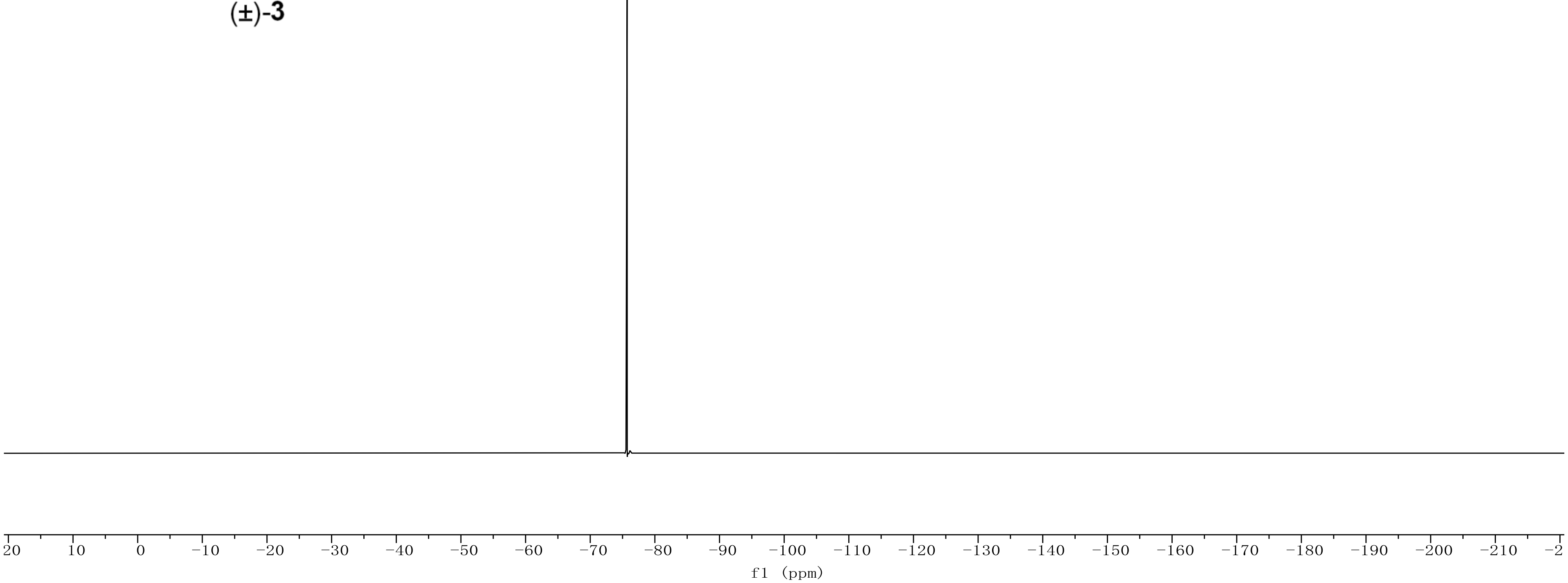

^{1}H NMR of (±)-2-bromo-arylpropanoate 4

$CDCl_3$, 23 ºC

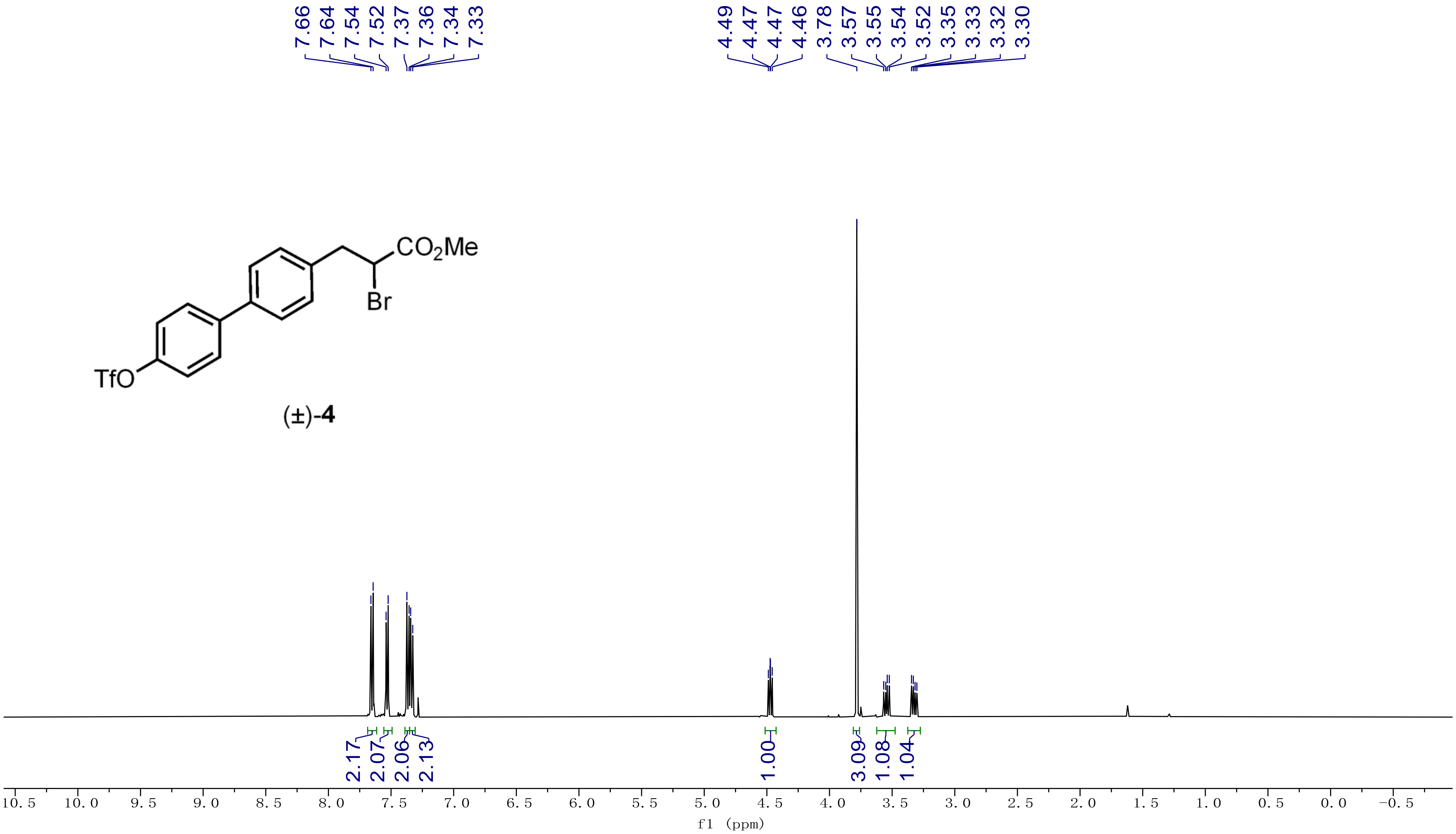

^{13}C NMR of (±)-2-bromo-arylpropanoate 4

CDCl₃, 23 ºC

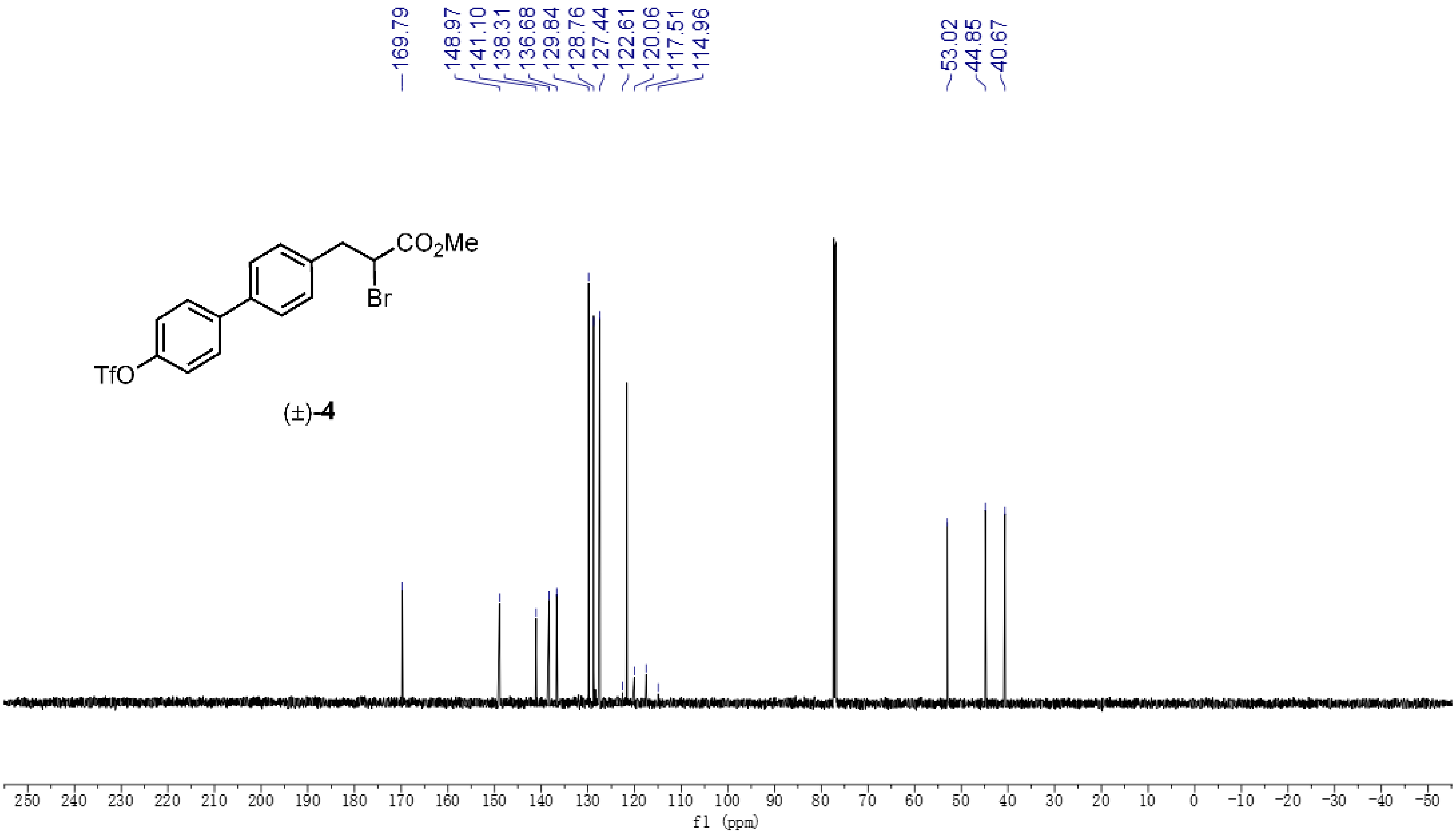

^{1}H NMR of (±)-flurbiprofen derivative 5

$CDCl_3$, 23 ºC

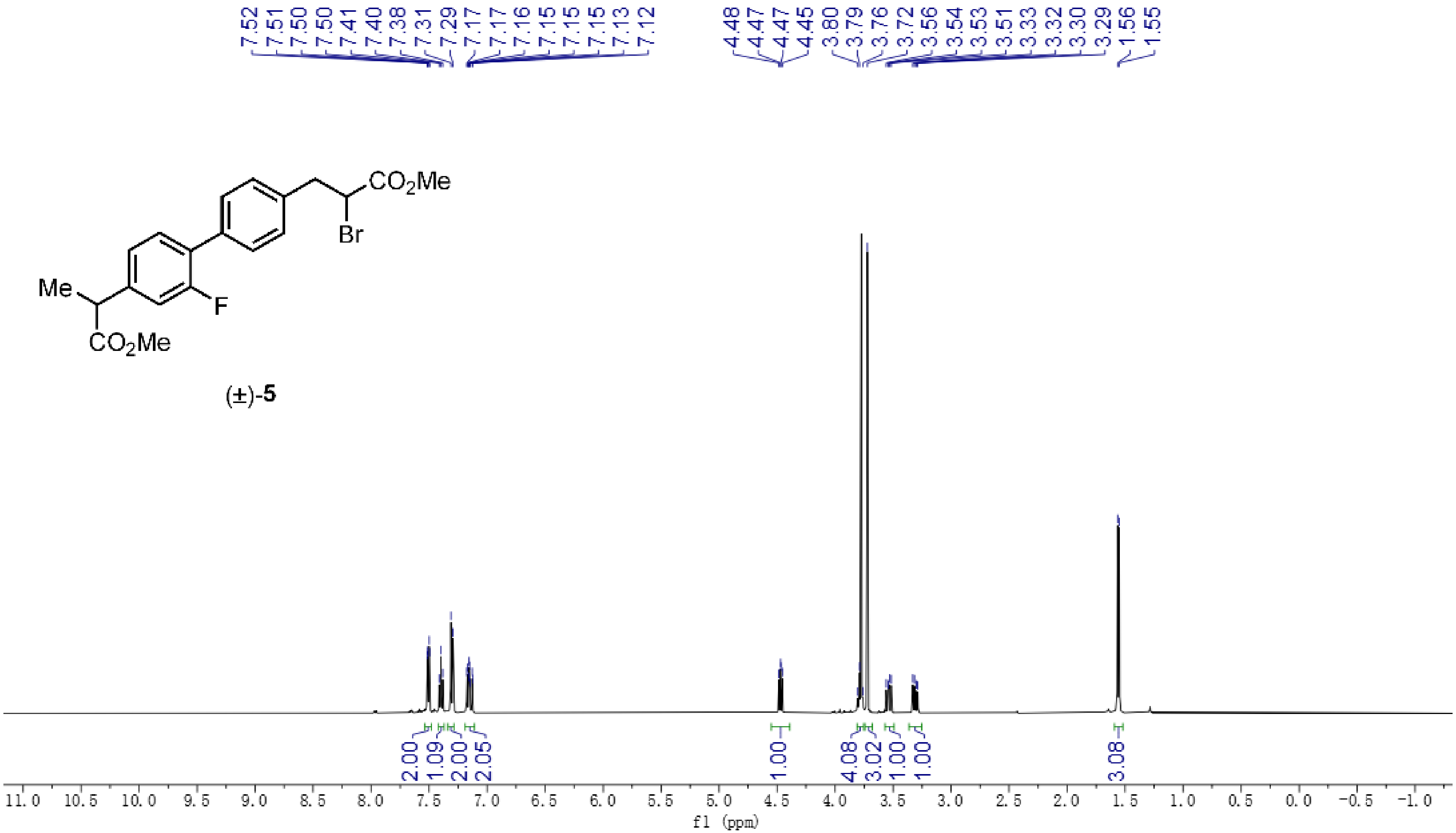

^{13}C NMR of (±)-flurbiprofen derivative 5

$CDCl_3$, 23 ºC

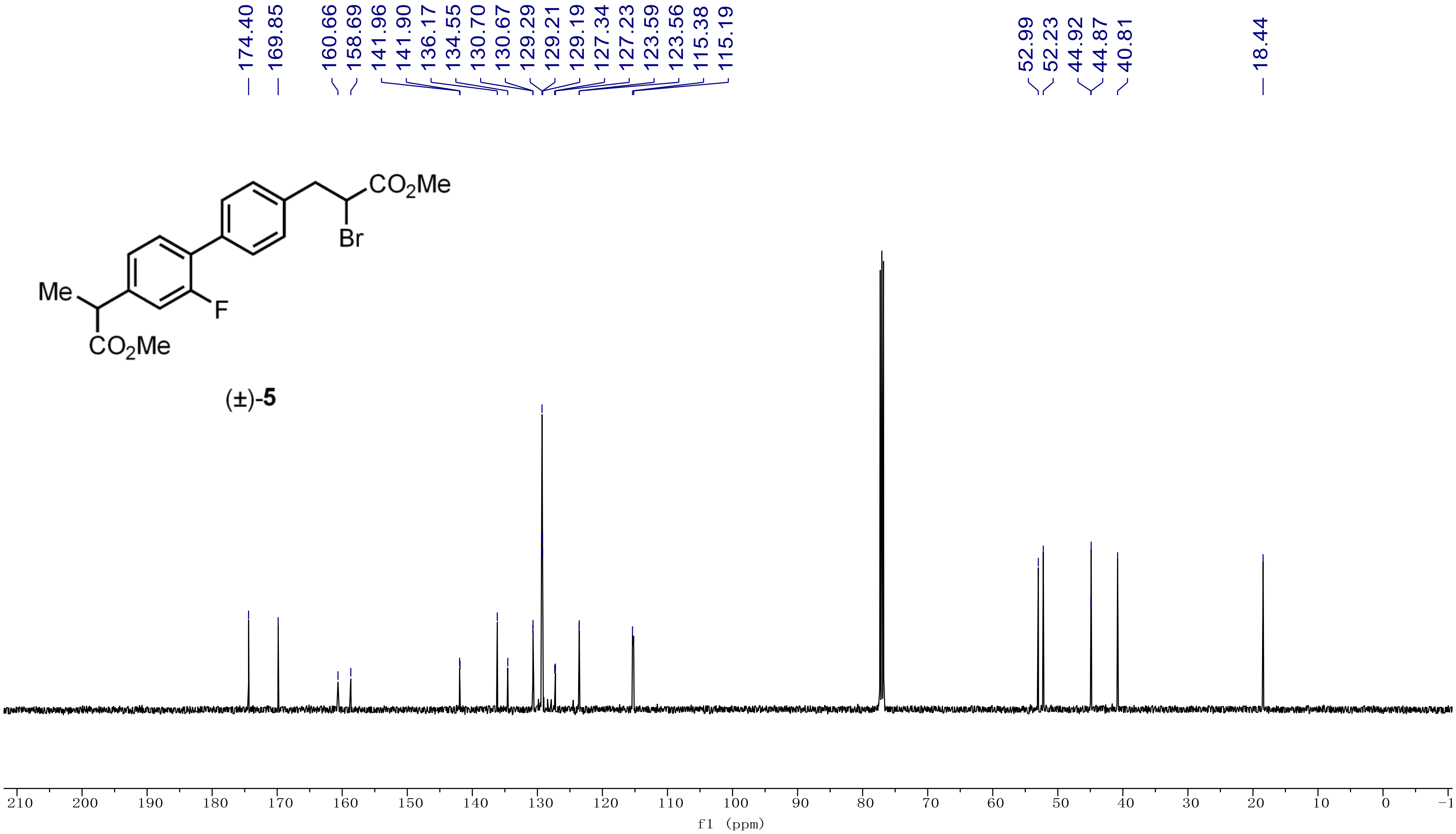

^{1}H NMR of (±)-methyl meclofenamate derivative 6

CDCl$_3$, 23 ºC

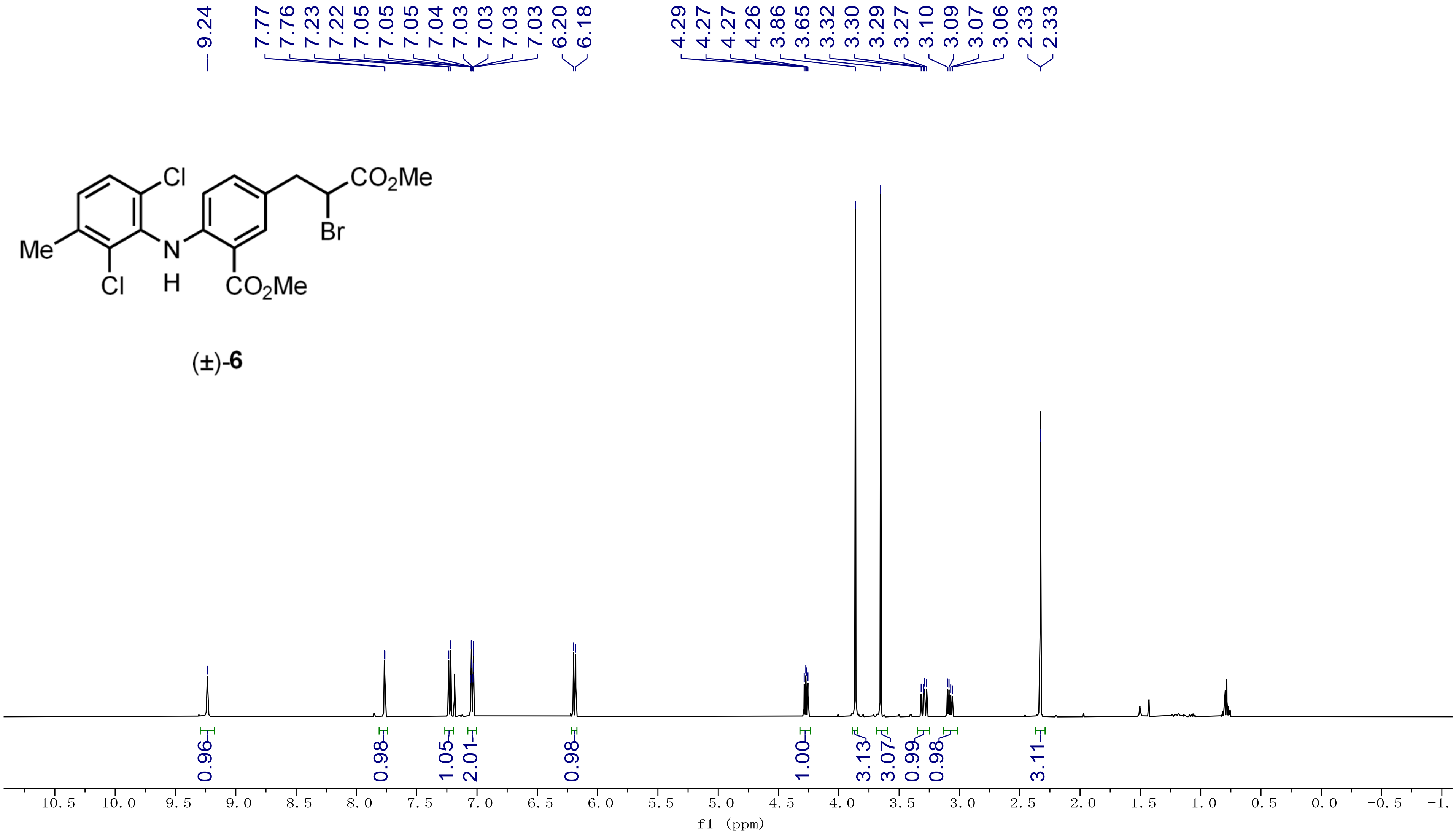

^{13}C NMR of (±)-methyl meclofenamate derivative 6

CDCl3, 23 ºC

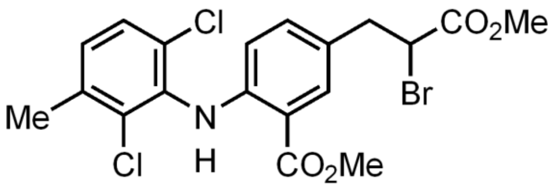

(±)-**6**

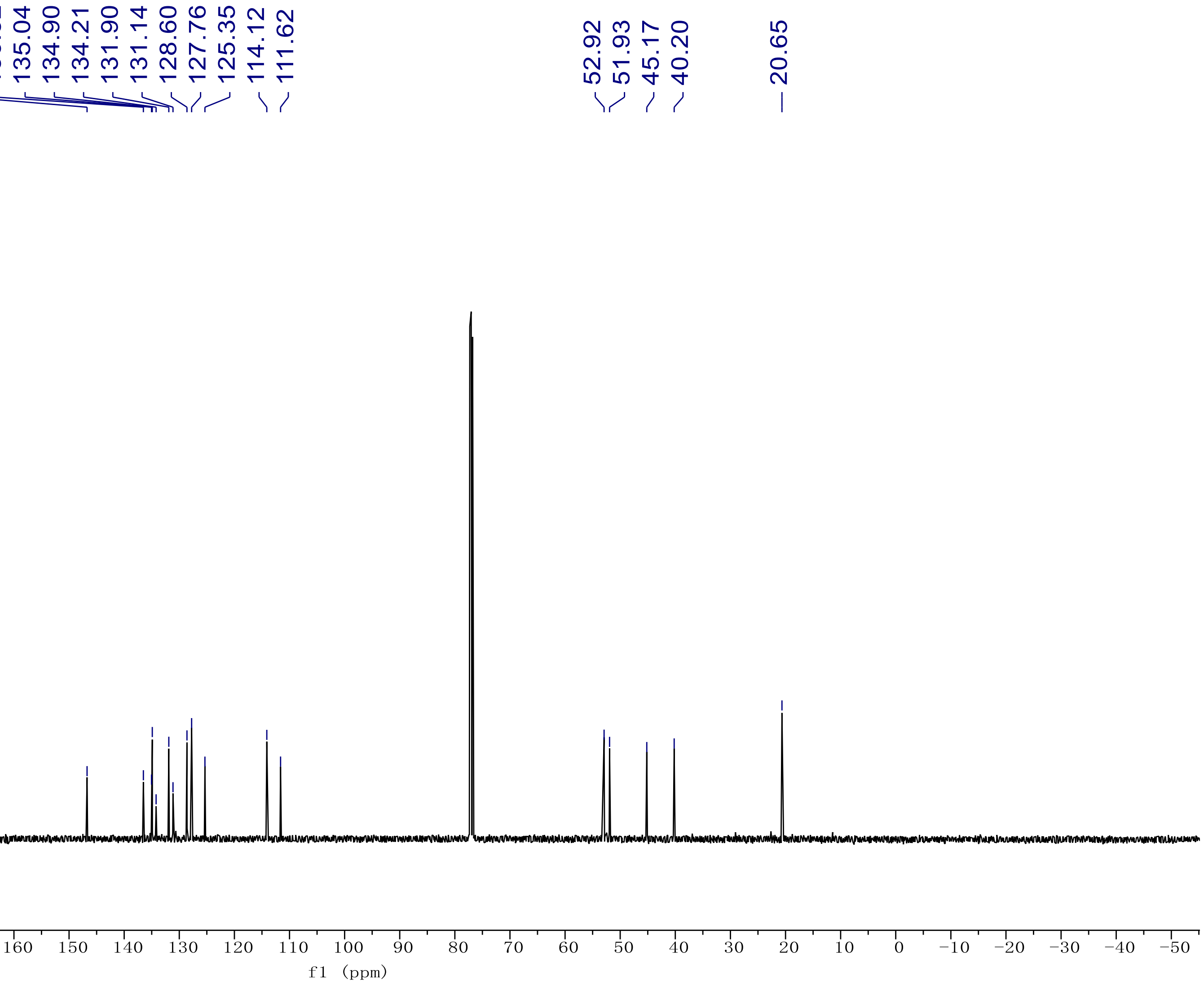

^{1}H NMR of (±)-2-bromo-arylpropanoate 7

$CDCl_3$, 23 ºC

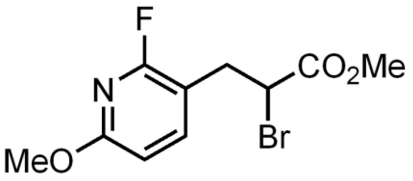

(±)-**7**

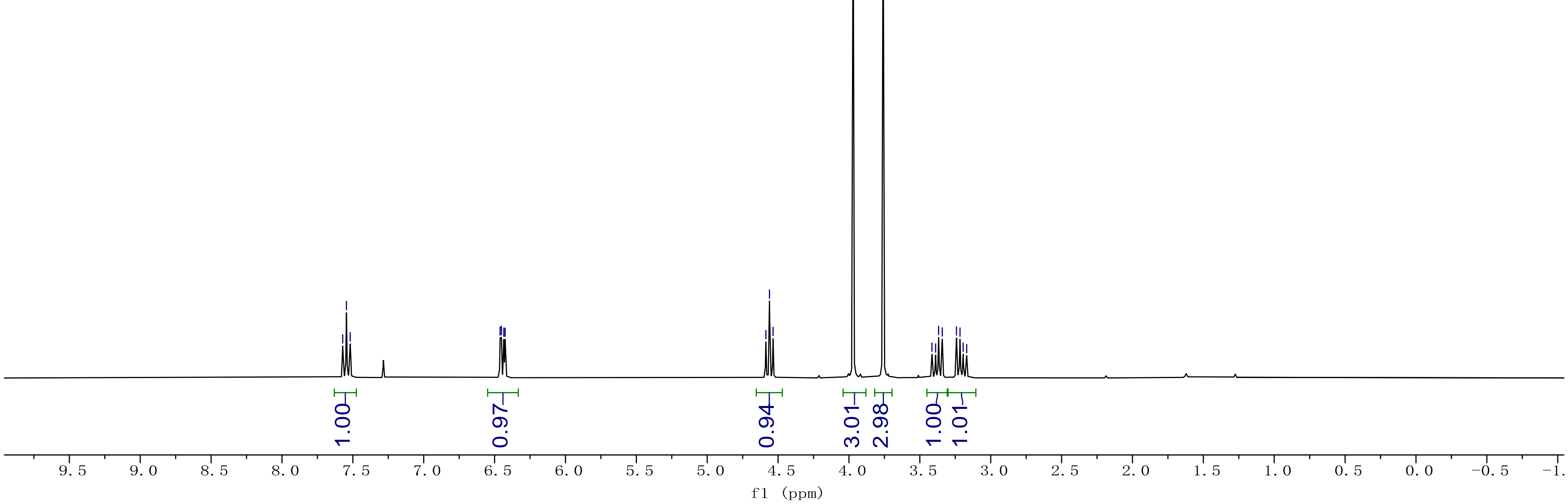

^{13}C NMR of (±)-2-bromo-arylpropanoate 7

CDCl$_3$, 23 ºC

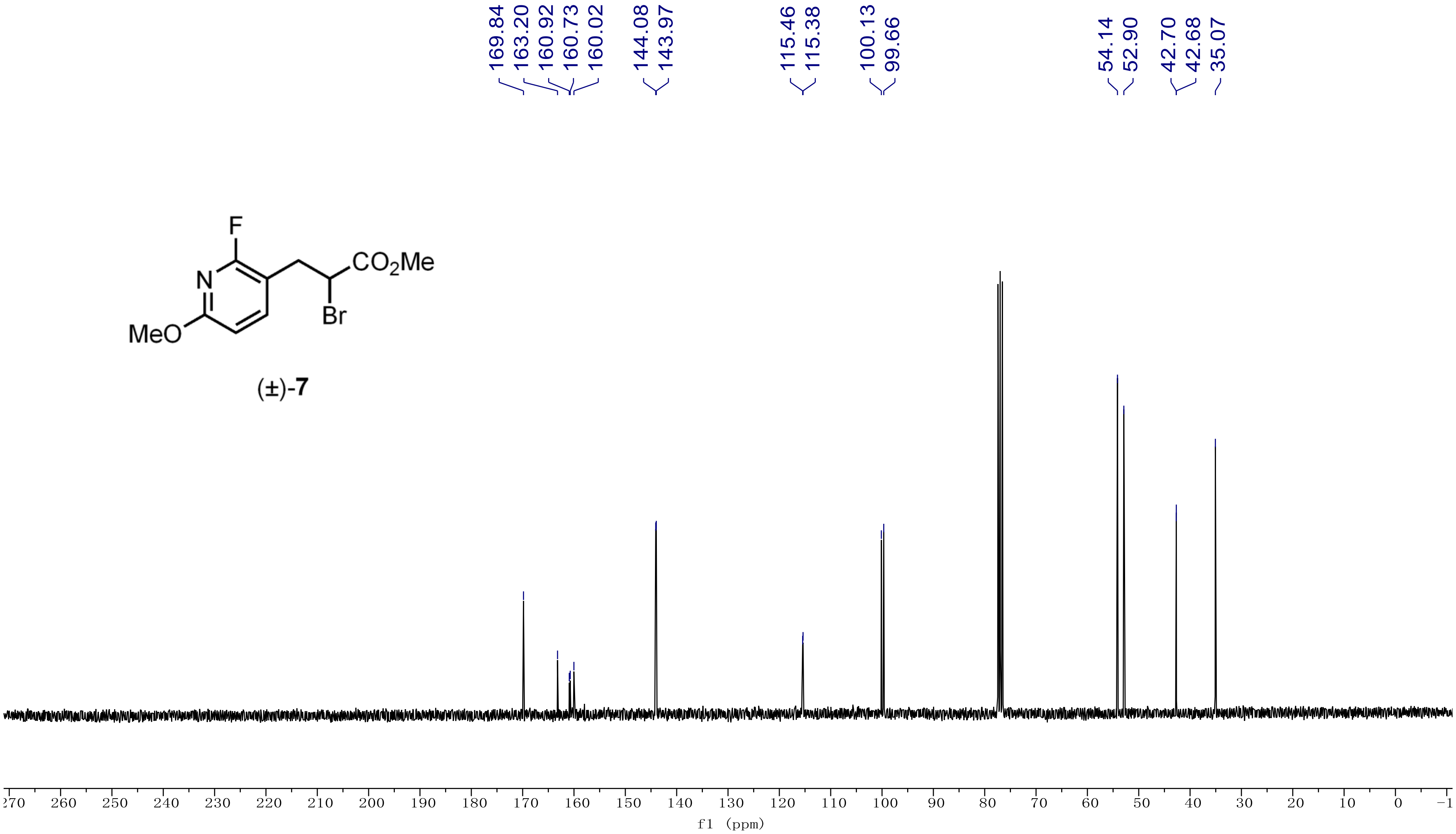

(±)-**7**

^{19}F NMR of (±)-2-bromo-arylpropanoate 7

$CDCl_3$, 23 ºC

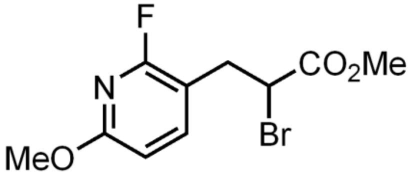

(±)-**7**

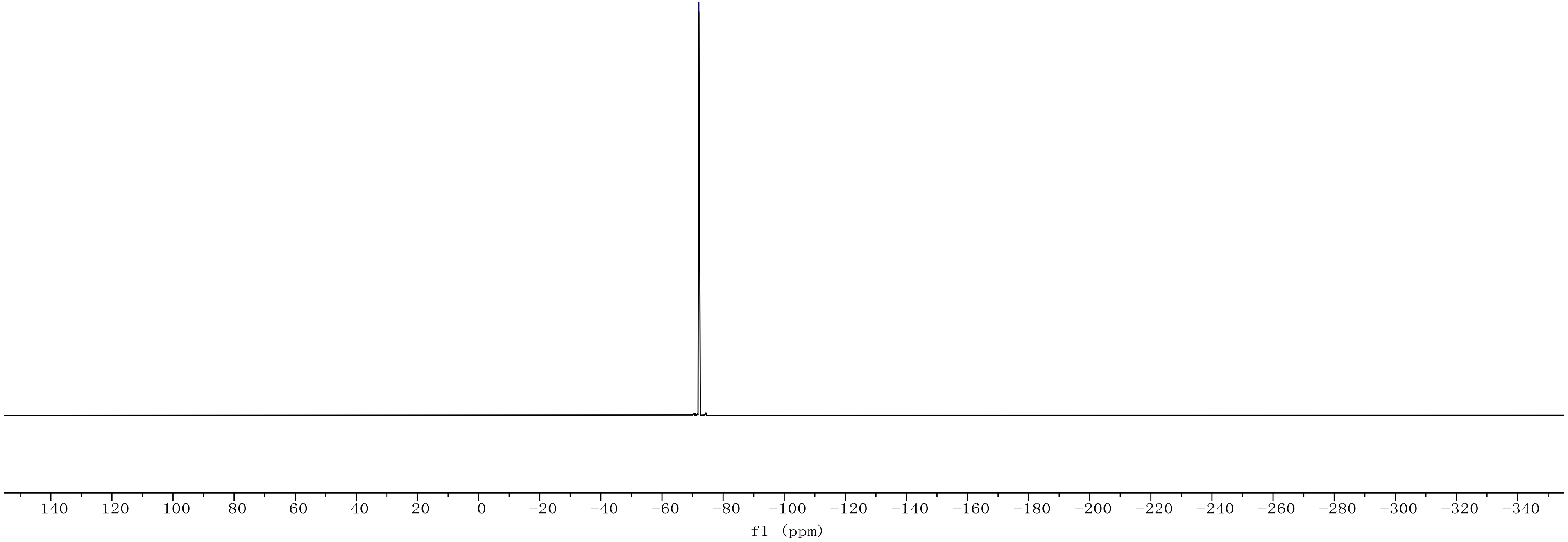

^{1}H NMR of (±)-2-bromo-arylpropanoate 8

CDCl$_3$, 23 ºC

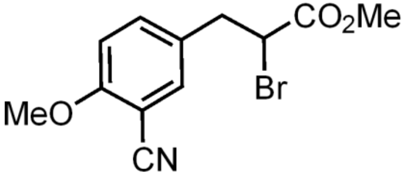

(±)-**8**

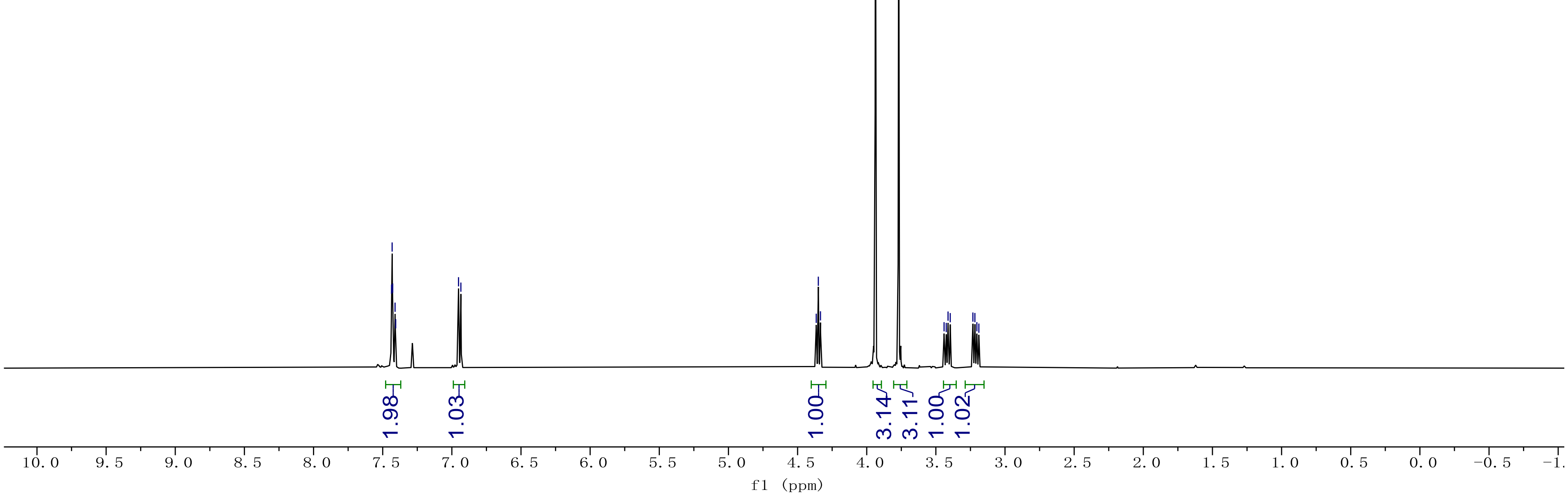

^{13}C NMR of (±)-2-bromo-arylpropanoate 8

$CDCl_3$, 23 ºC

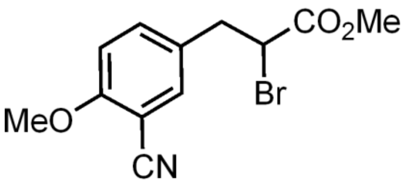

(±)-**8**

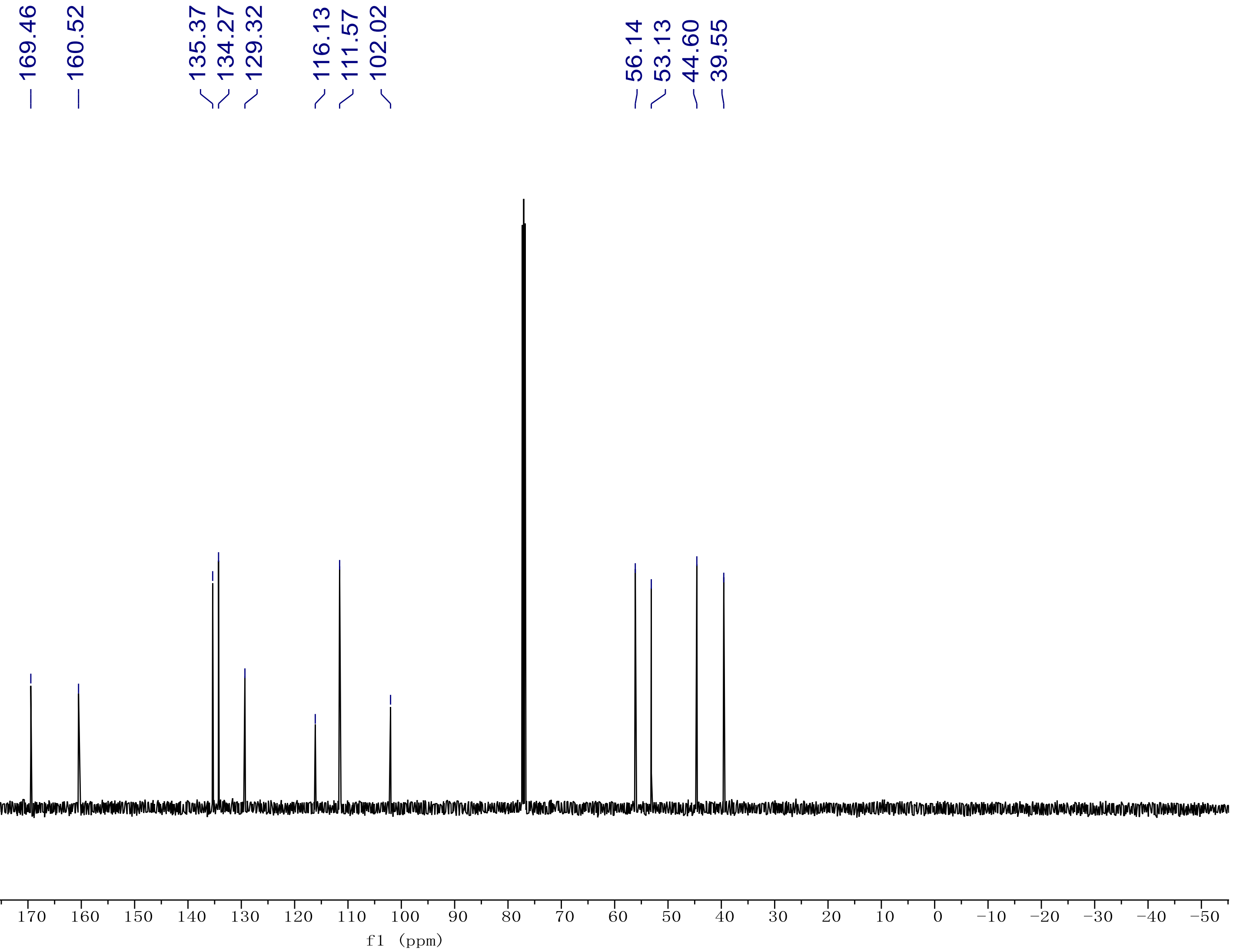

¹H NMR of (±)-2-bromo-arylpropanoate 9

$CDCl_3$, 23 ºC

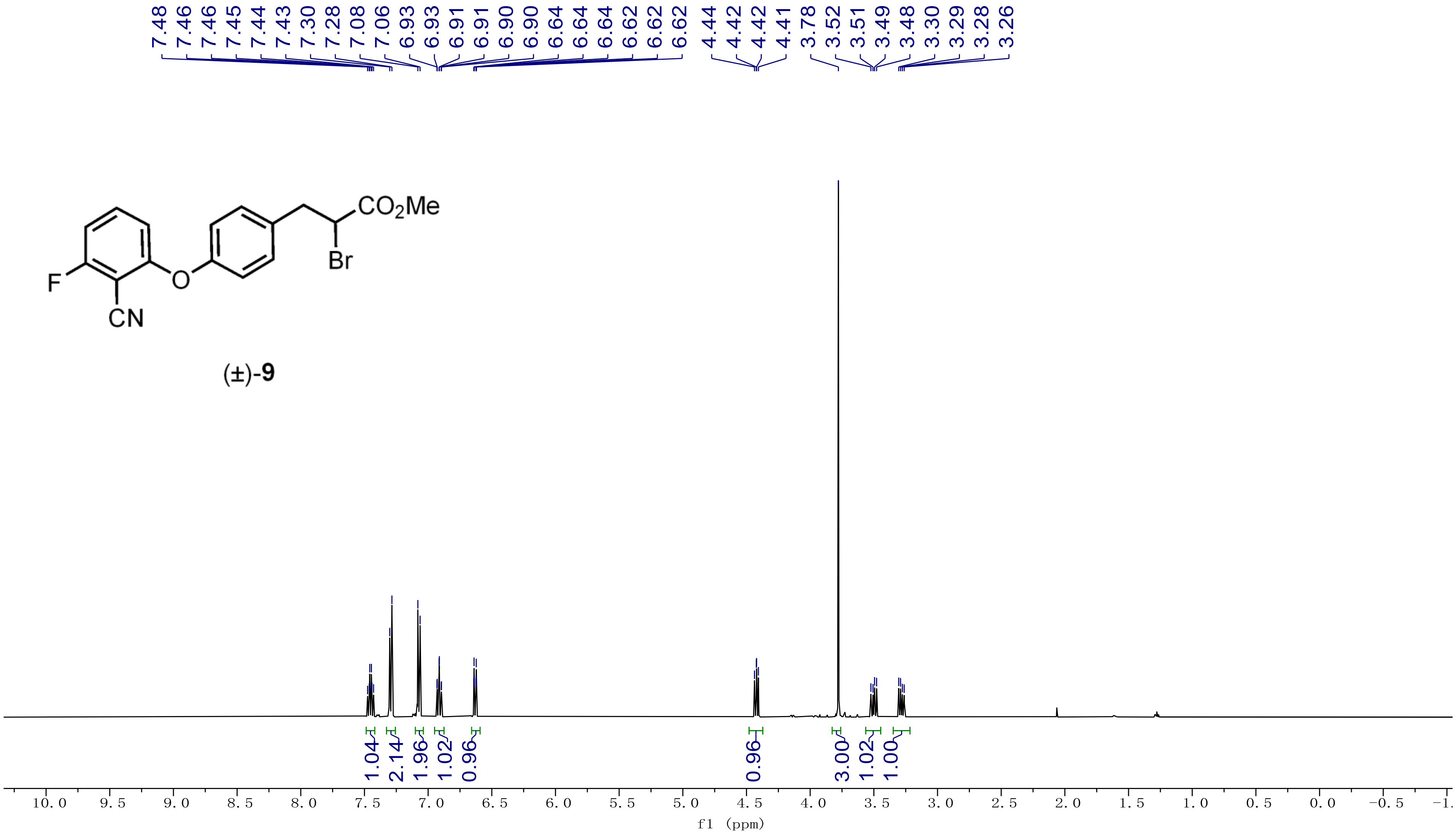

(±)-**9**

^{13}C NMR of (±)-2-bromo-arylpropanoate 9

$CDCl_3$, 23 ºC

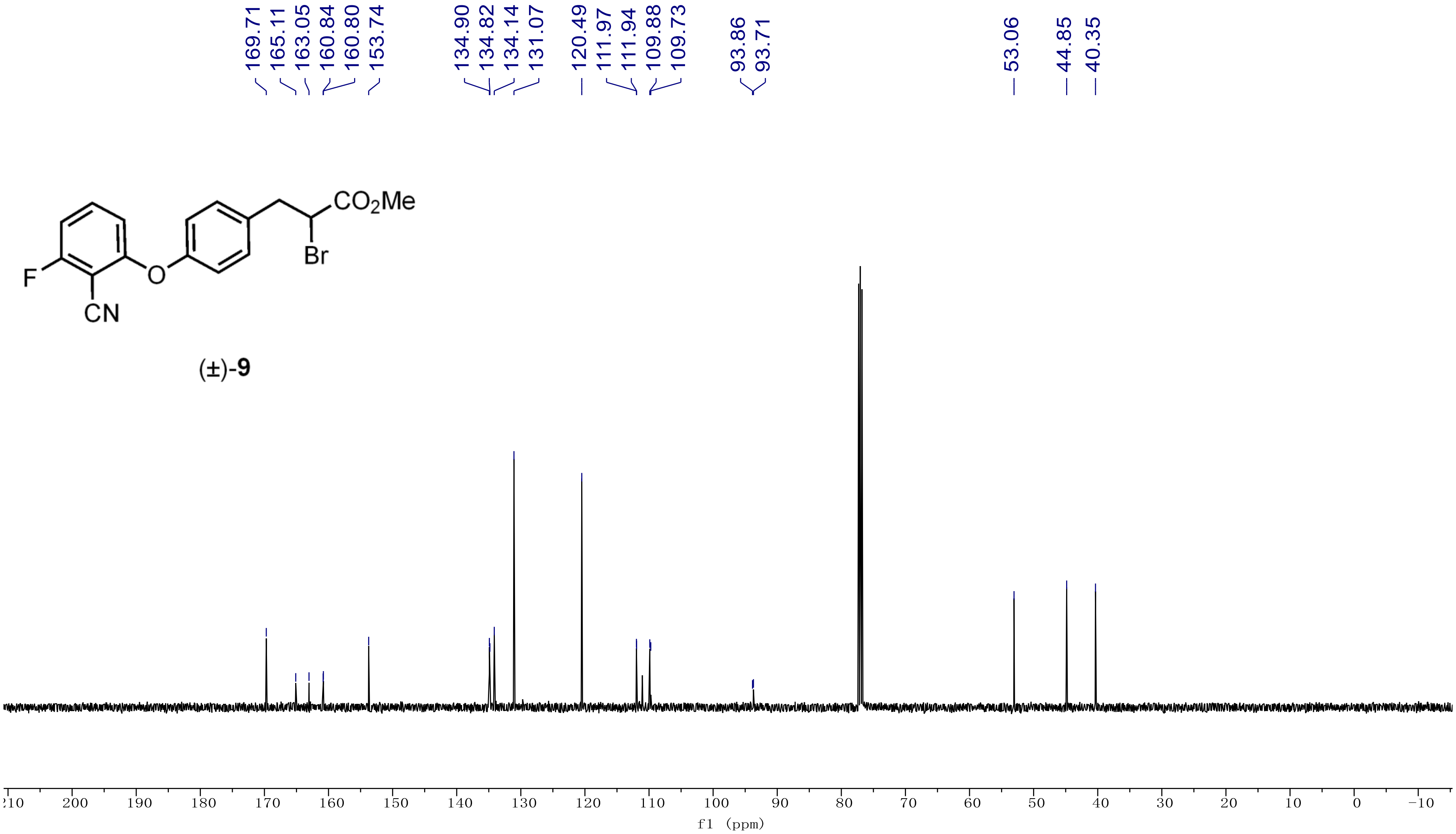

^{1}H NMR of (±)-diclofenac amide derivative 10

$CDCl_3$, 23 ºC

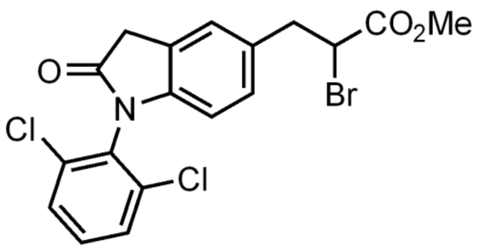

(±)-**10**

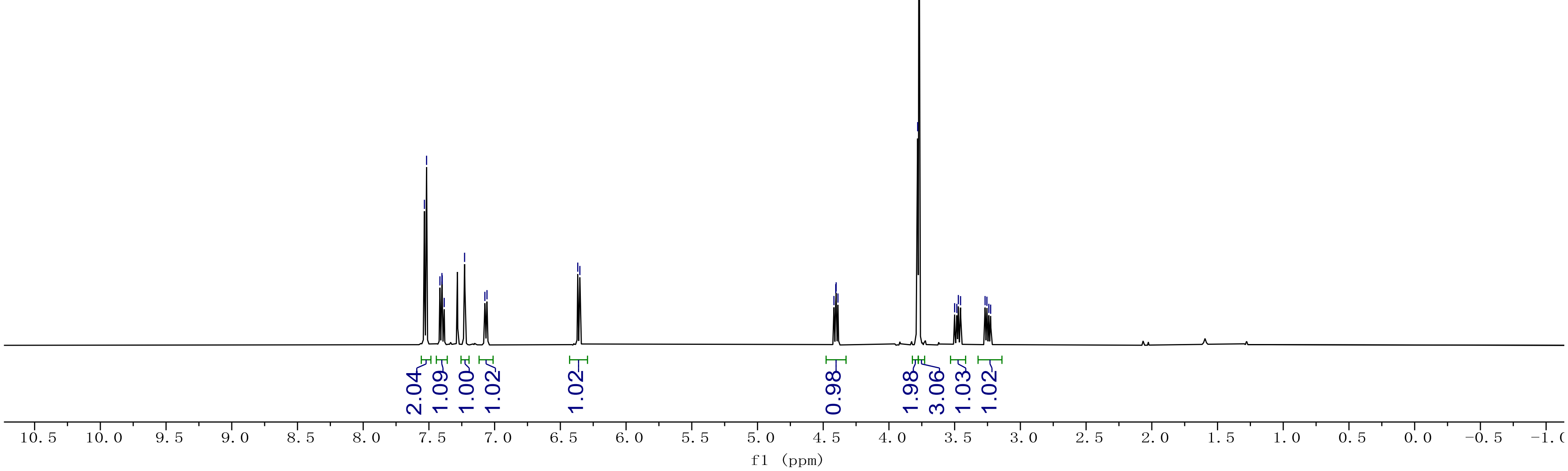

^{13}C NMR of (±)-diclofenac amide derivative 10

$CDCl_3$, 23 ºC

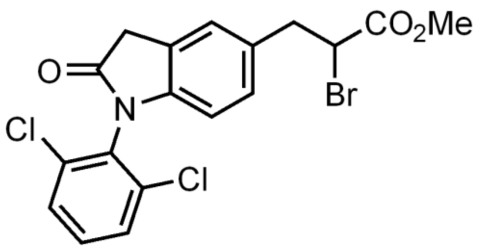

(±)-**10**

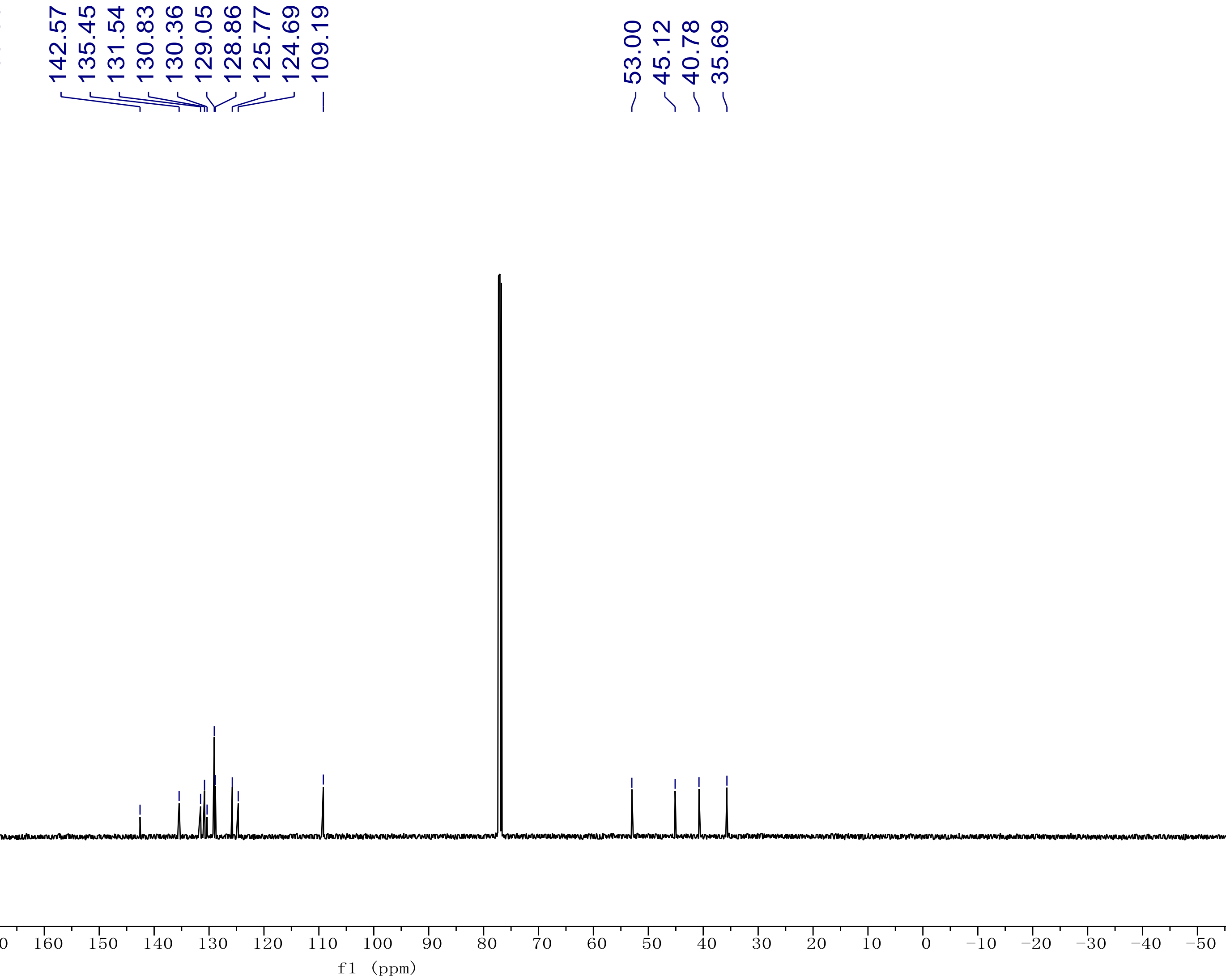

^{1}H NMR of (±)-*N*-methyl-nimesulide derivative 11

$CDCl_3$, 23 ºC

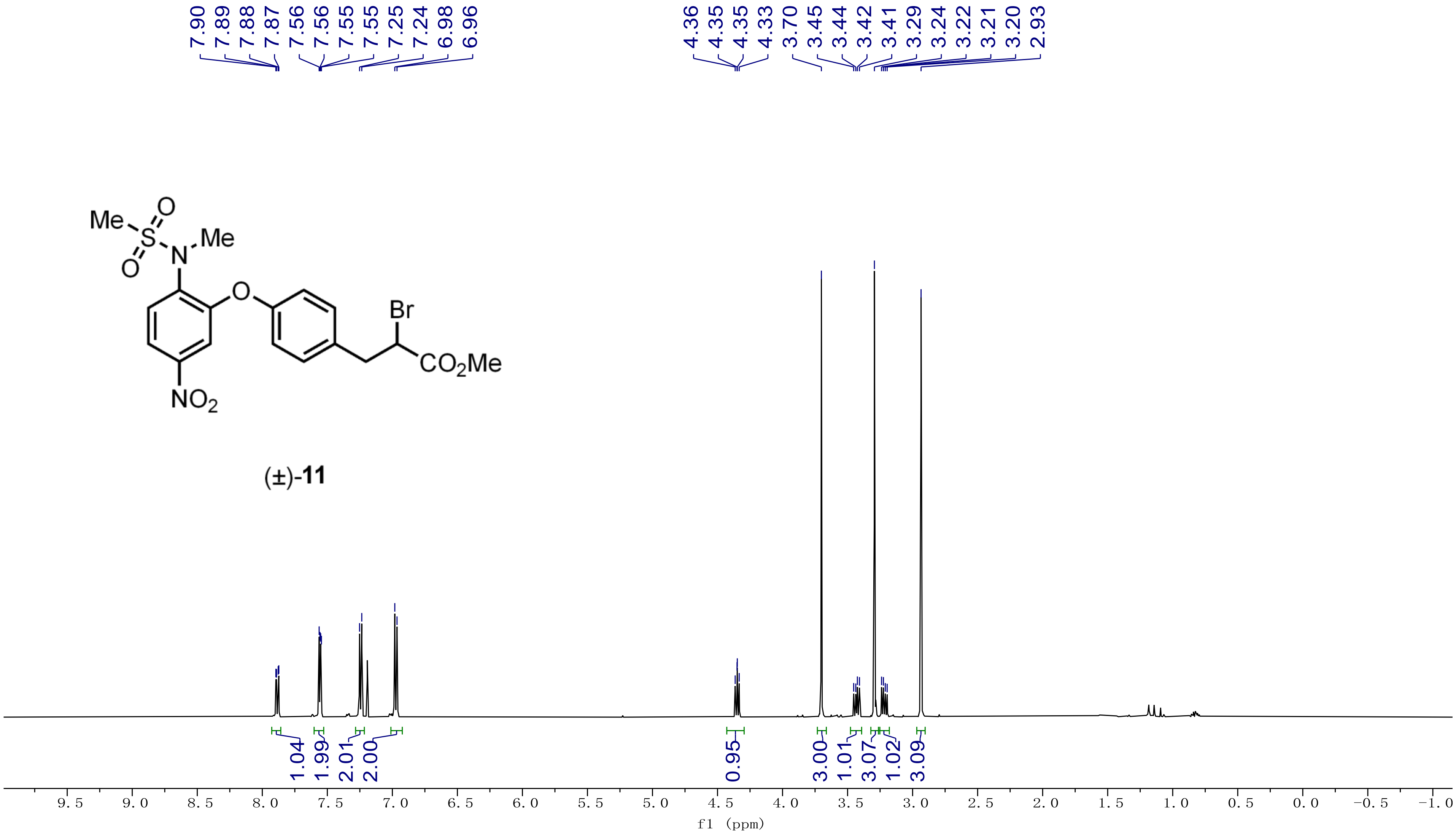

(±)-**11**

^{13}C NMR of (±)-*N*-methyl-nimesulide derivative 11

$CDCl_3$, 23 ºC

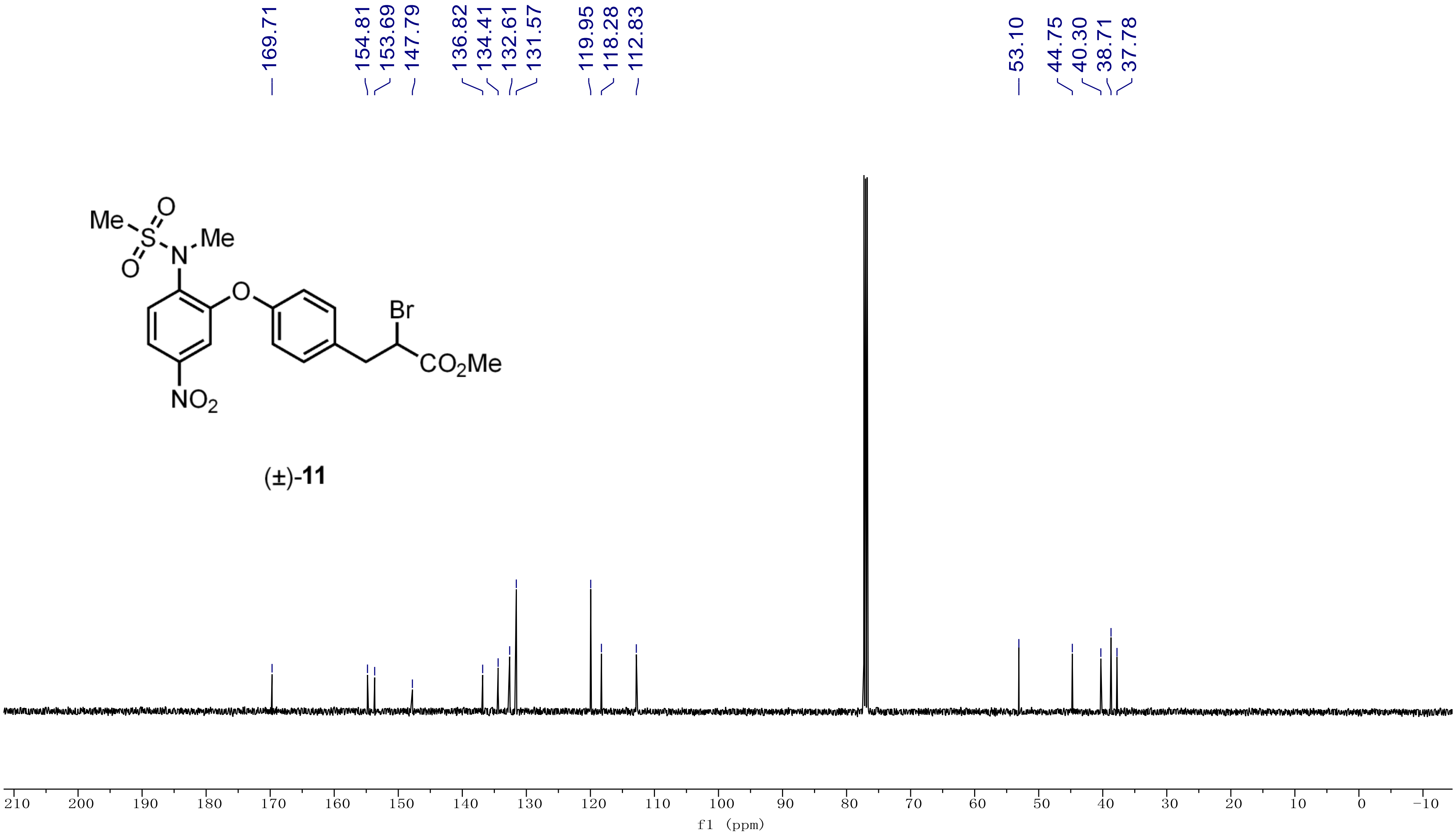

^{1}H NMR of (±)-2-bromo-arylpropanoate 12

$CDCl_3$, 23 ºC

(±)-**12**

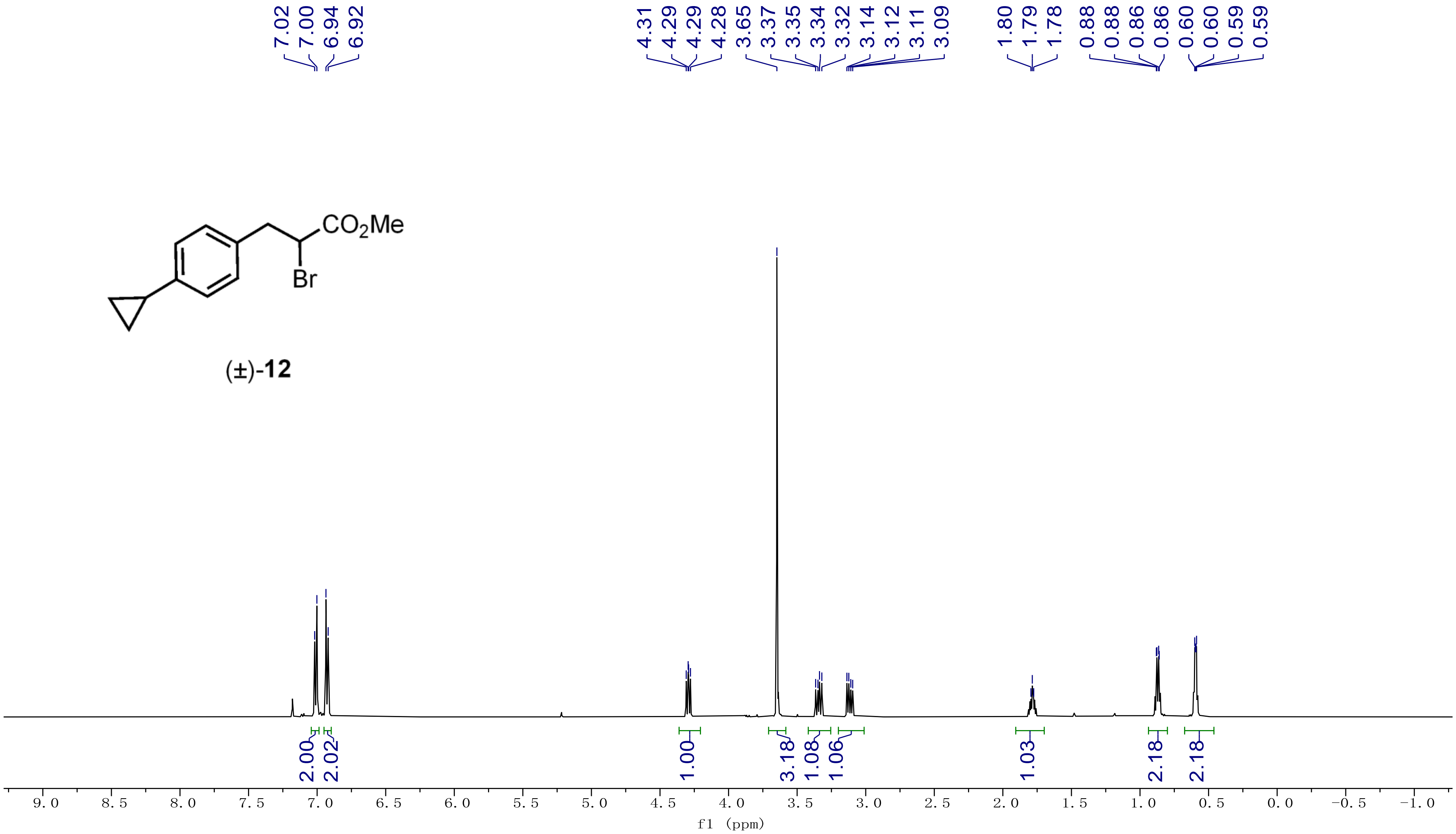

^{13}C NMR of (±)-2-bromo-arylpropanoate 12

$CDCl_3$, 23 ºC

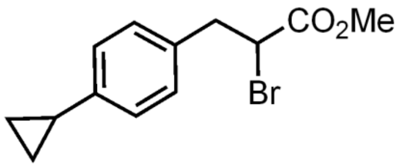

(±)-**12**

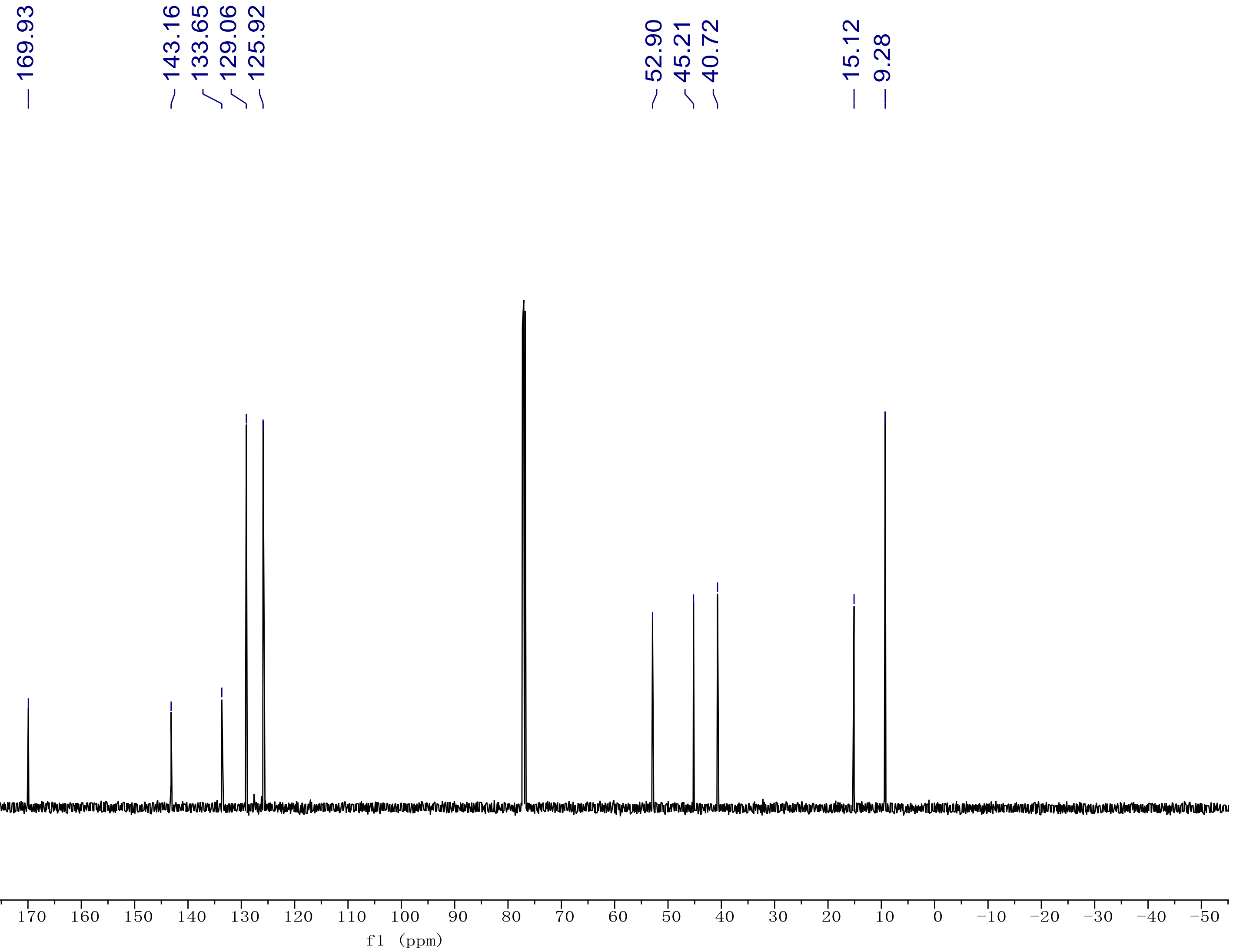

^{1}H NMR of (±)-2-bromo-arylpropanoate 13

$CDCl_3$, 23 ºC

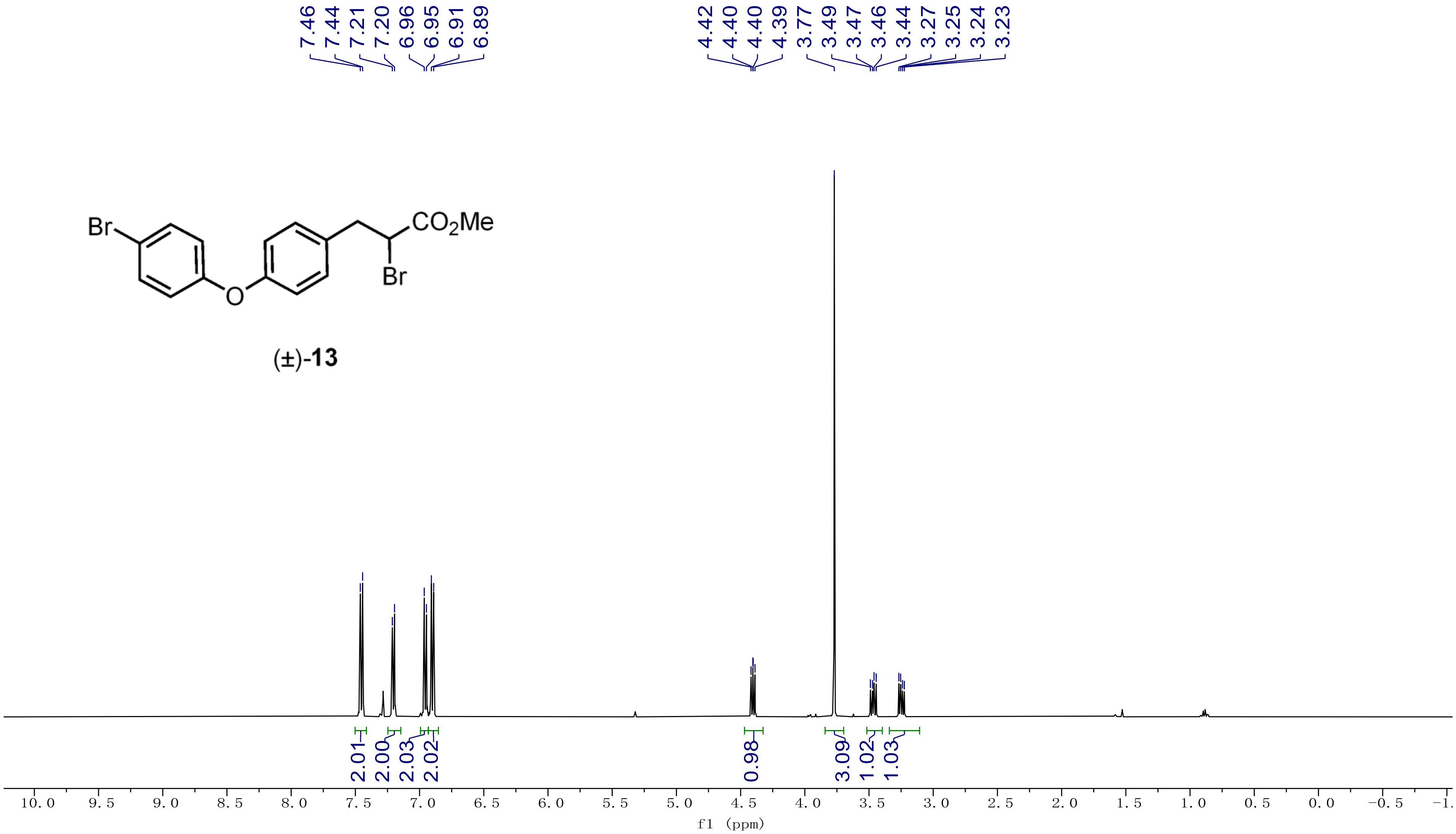

^{13}C NMR of (±)-2-bromo-arylpropanoate 13

$CDCl_3$, 23 ºC

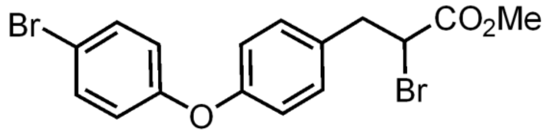

(±)-**13**

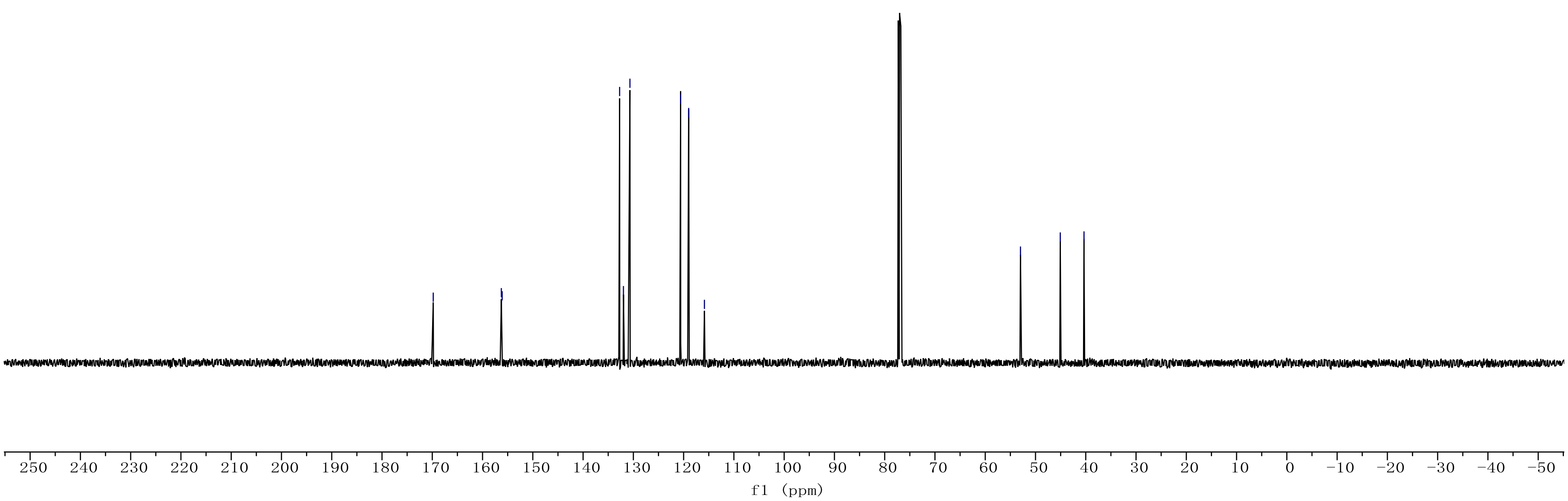

^{1}H NMR of (±)-2-bromo-arylpropanoate 14

CDCl3, 23 ºC

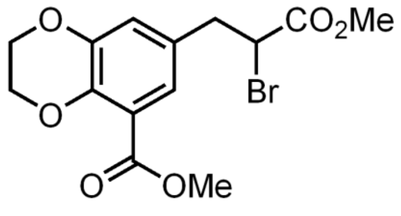

(±)-**14**

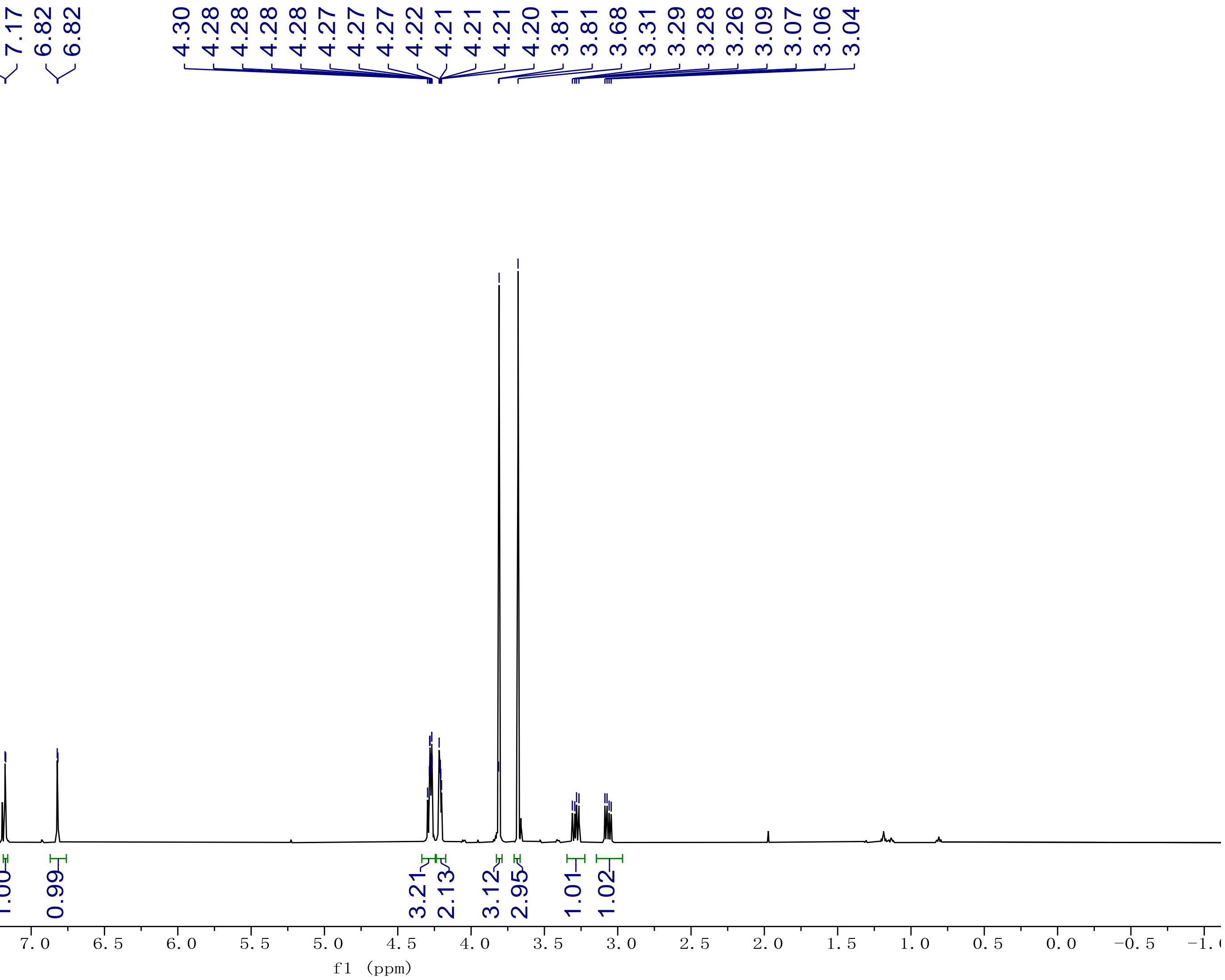

^{13}C NMR of (±)-2-bromo-arylpropanoate 14

$CDCl_3$, 23 ºC

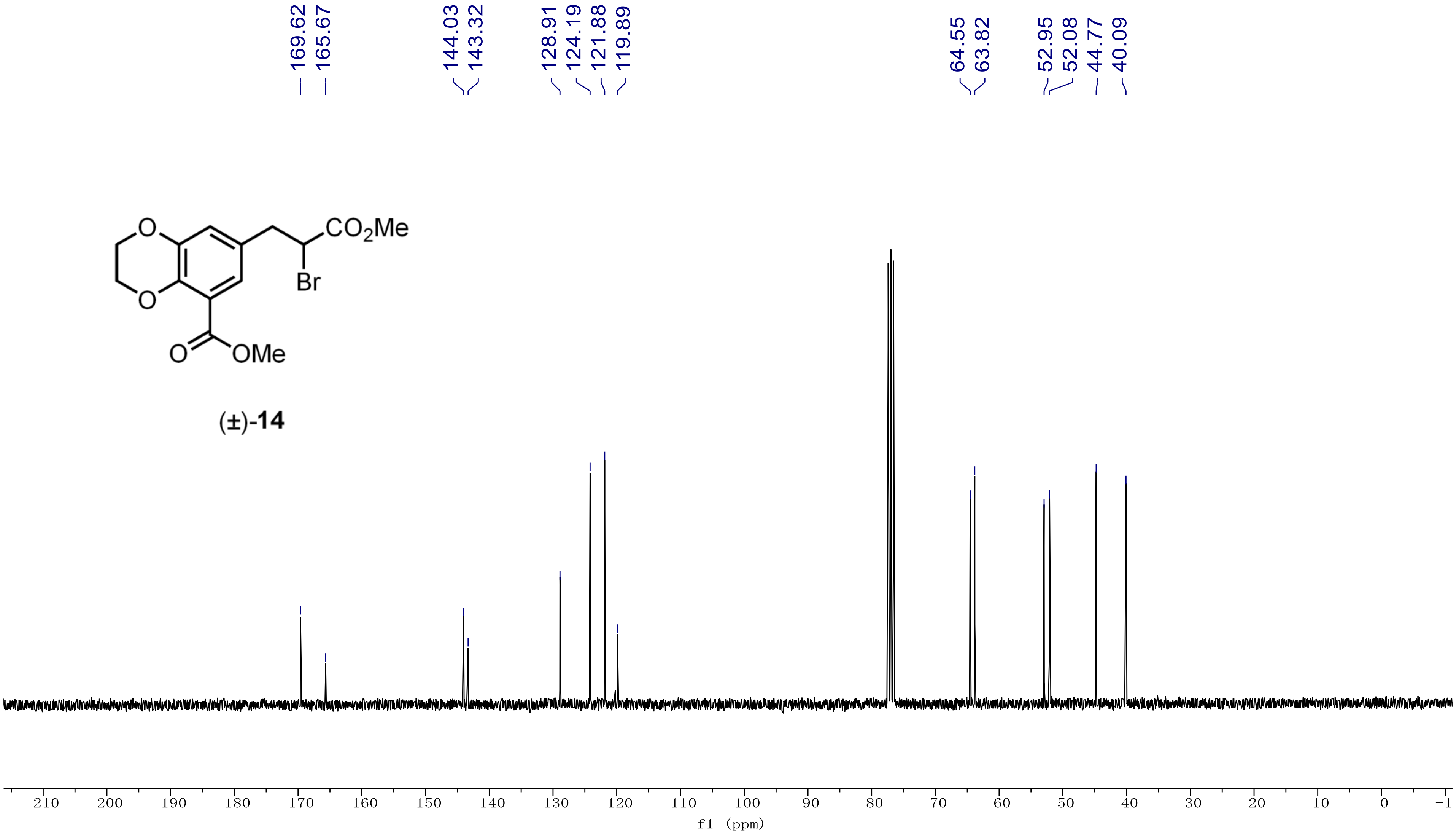

^{1}H NMR of (±)-boscalid derivative 15

CDCl3, 23 ºC

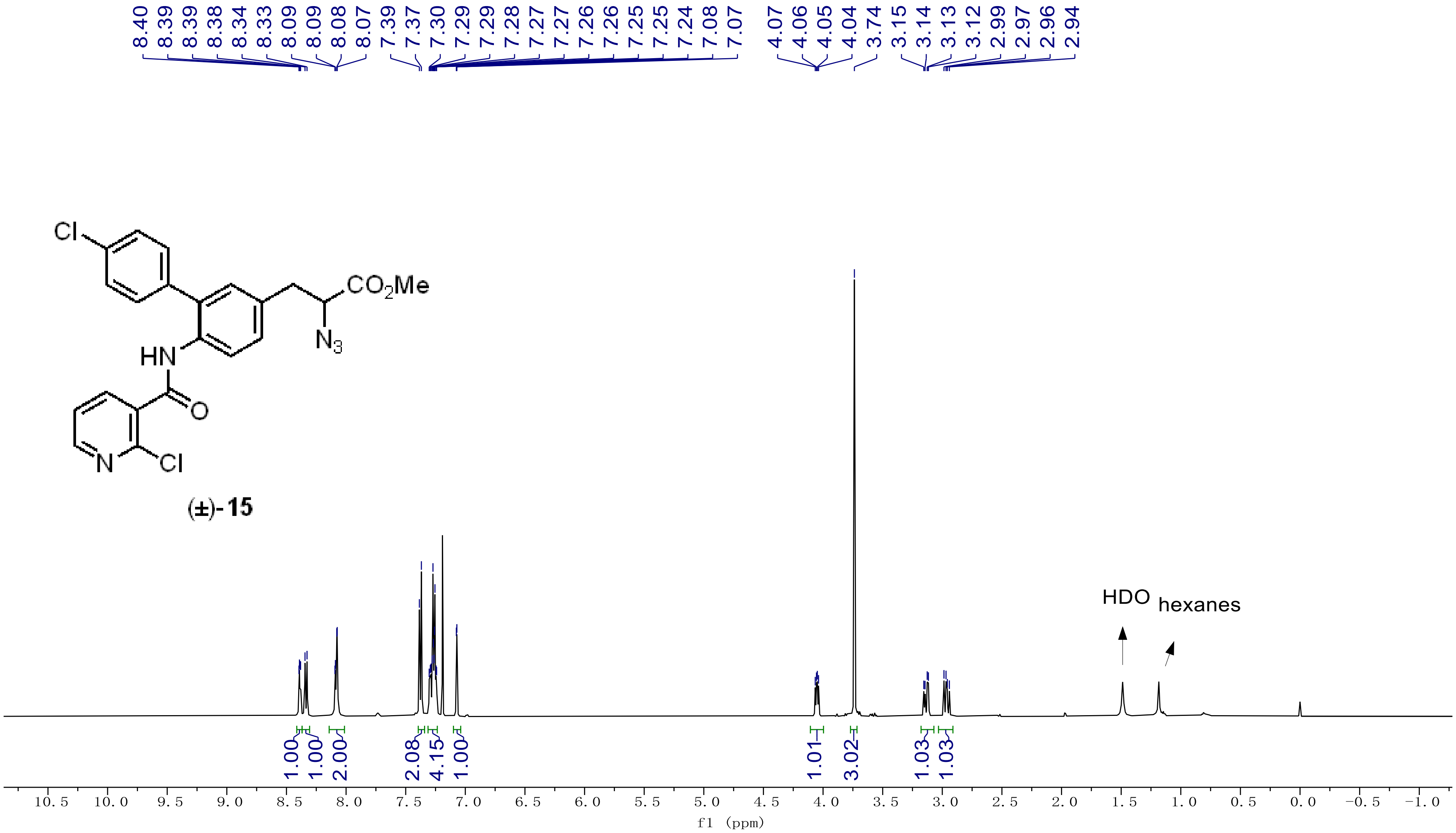

^{13}C NMR of (±)-boscalid derivative 15

$CDCl_3$, 23 ºC

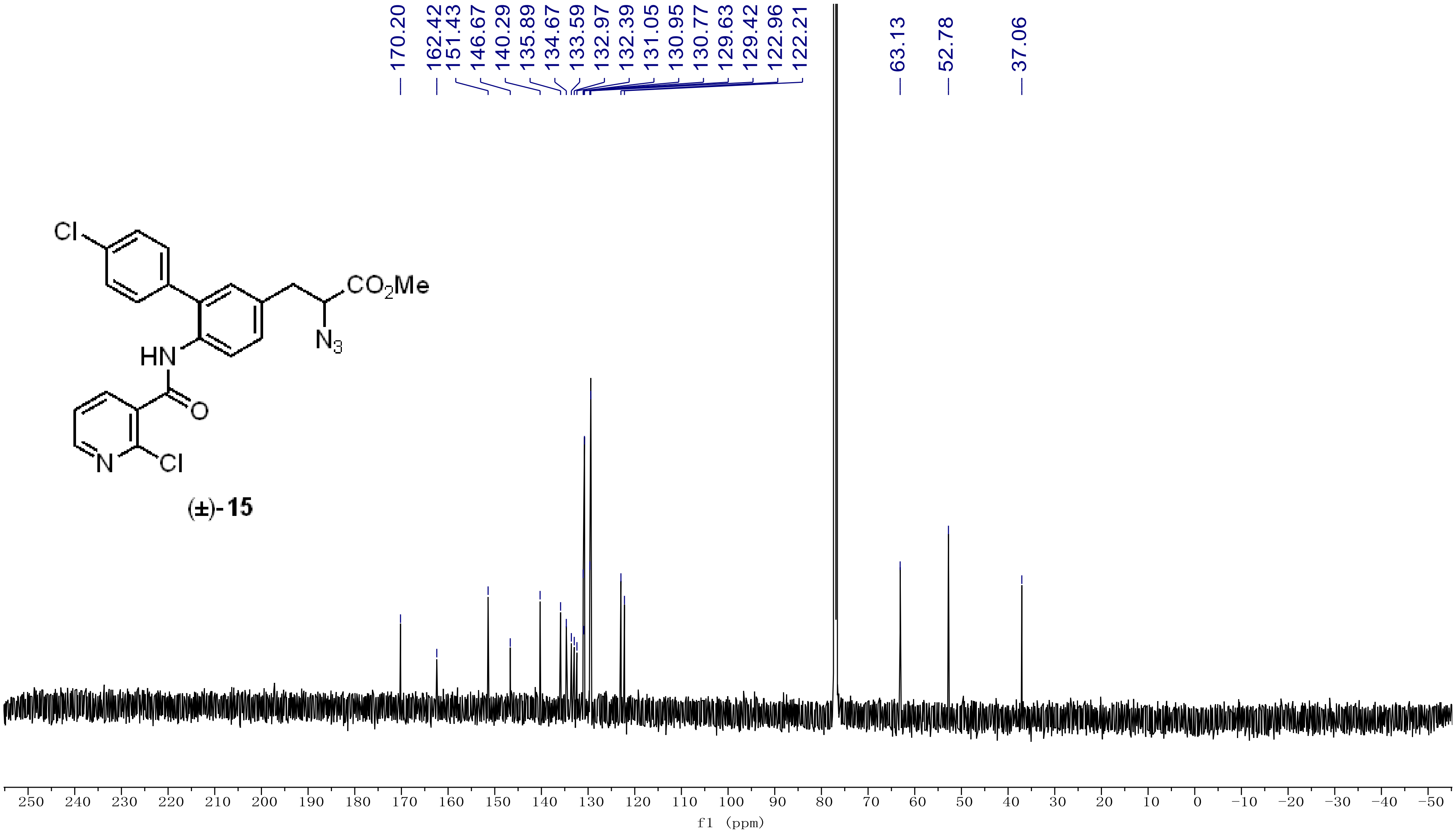

^{1}H NMR of (±)-etofenprox derivative 16

$CDCl_3$, 23 ºC

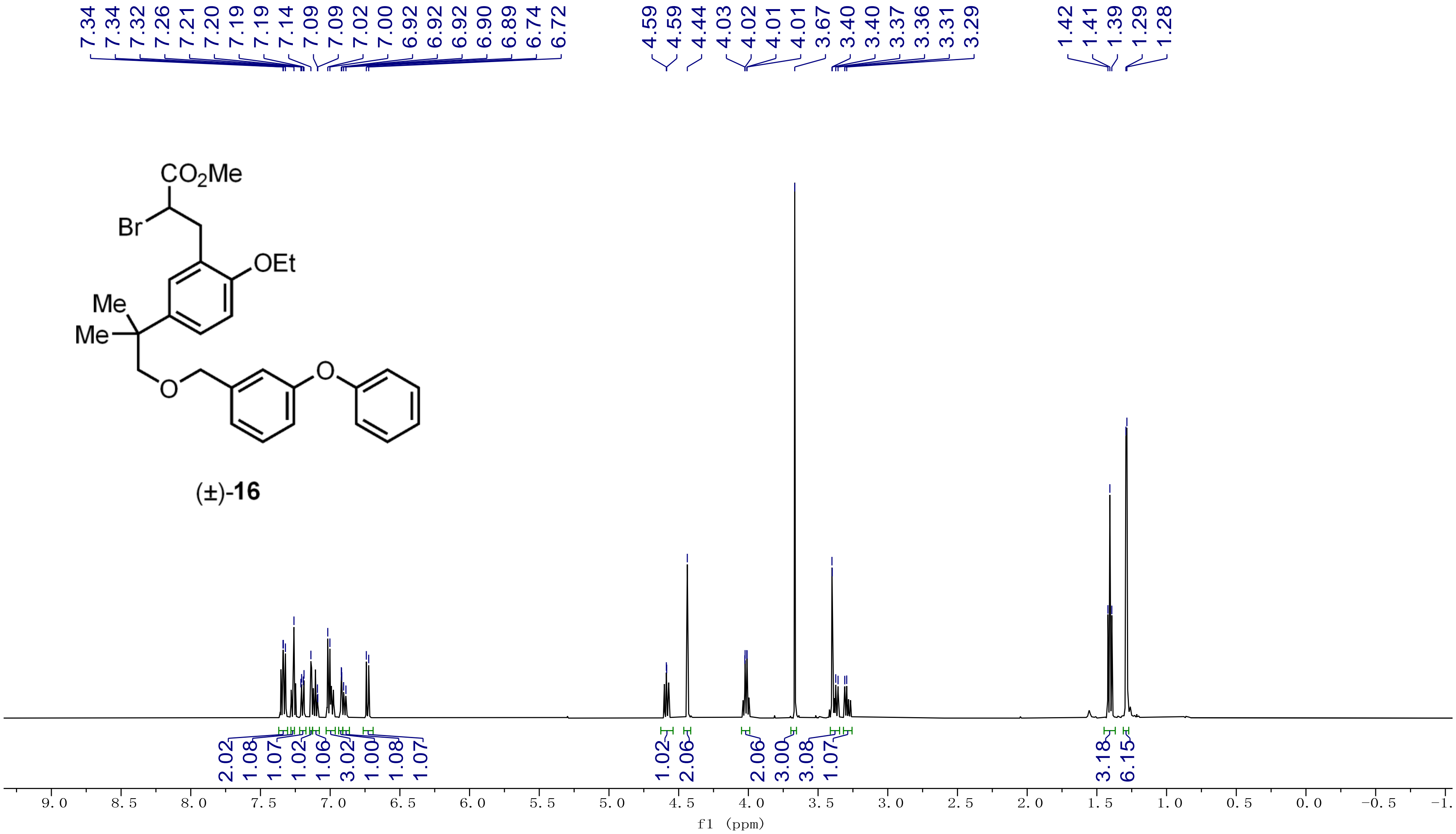

^{13}C NMR of (±)-etofenprox derivative 16

$CDCl_3$, 23 ºC

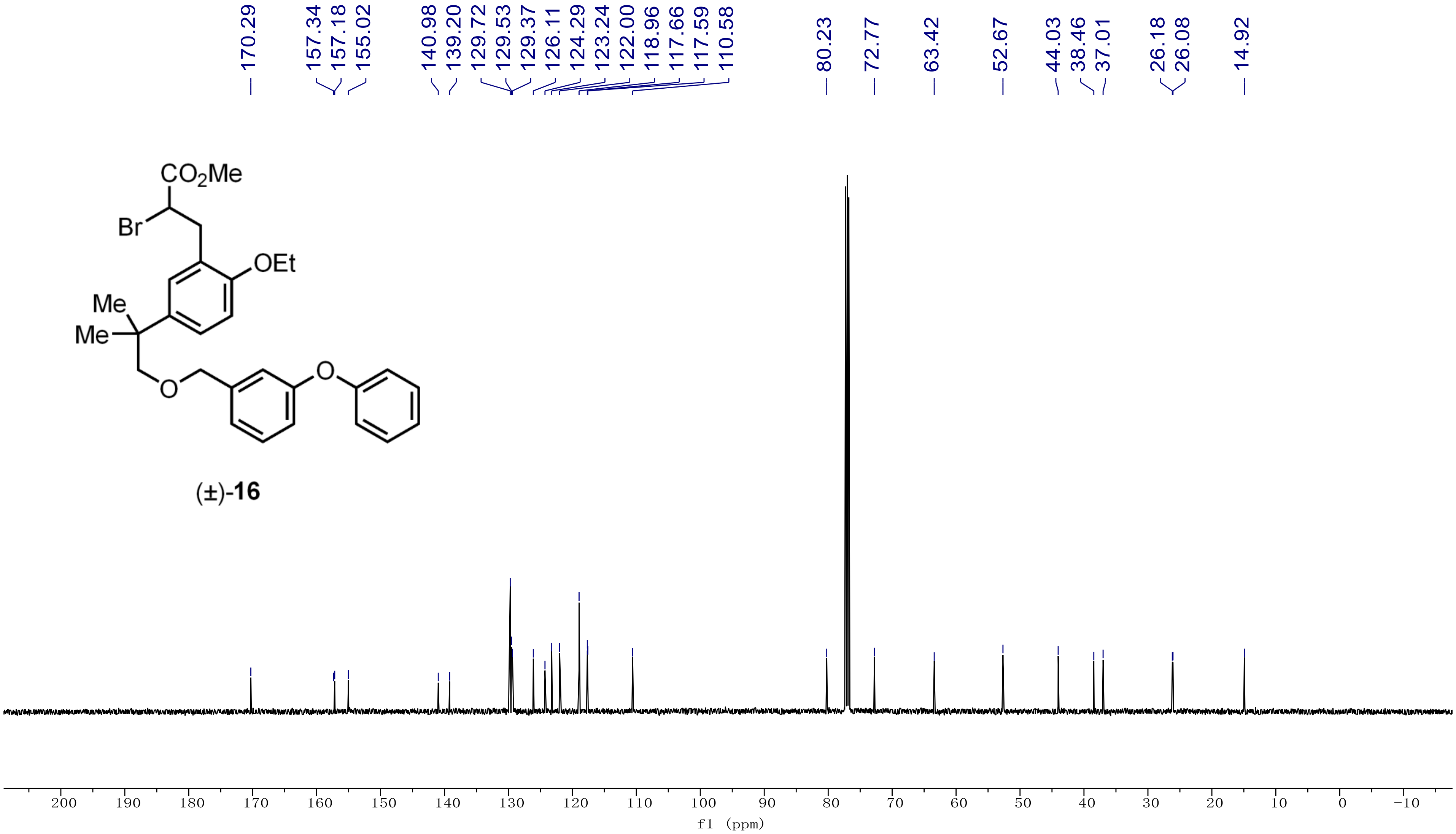

(±)-**16**

^{1}H NMR of (±)-2-bromo-arylpropanoate 17

$CDCl_3$, 23 ºC

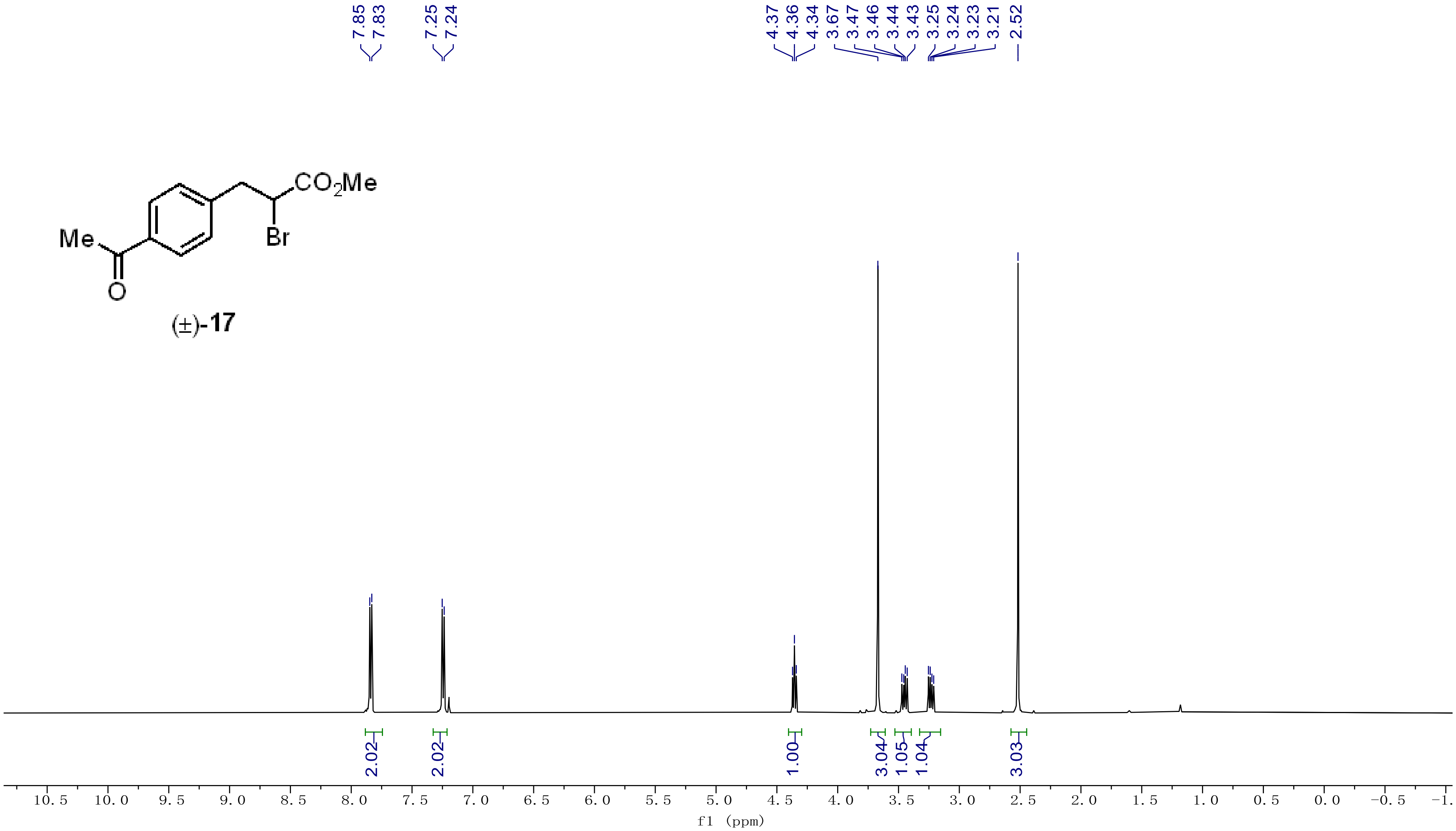

^{13}C NMR of (±)-2-bromo-arylpropanoate 17

$CDCl_3$, 23 ºC

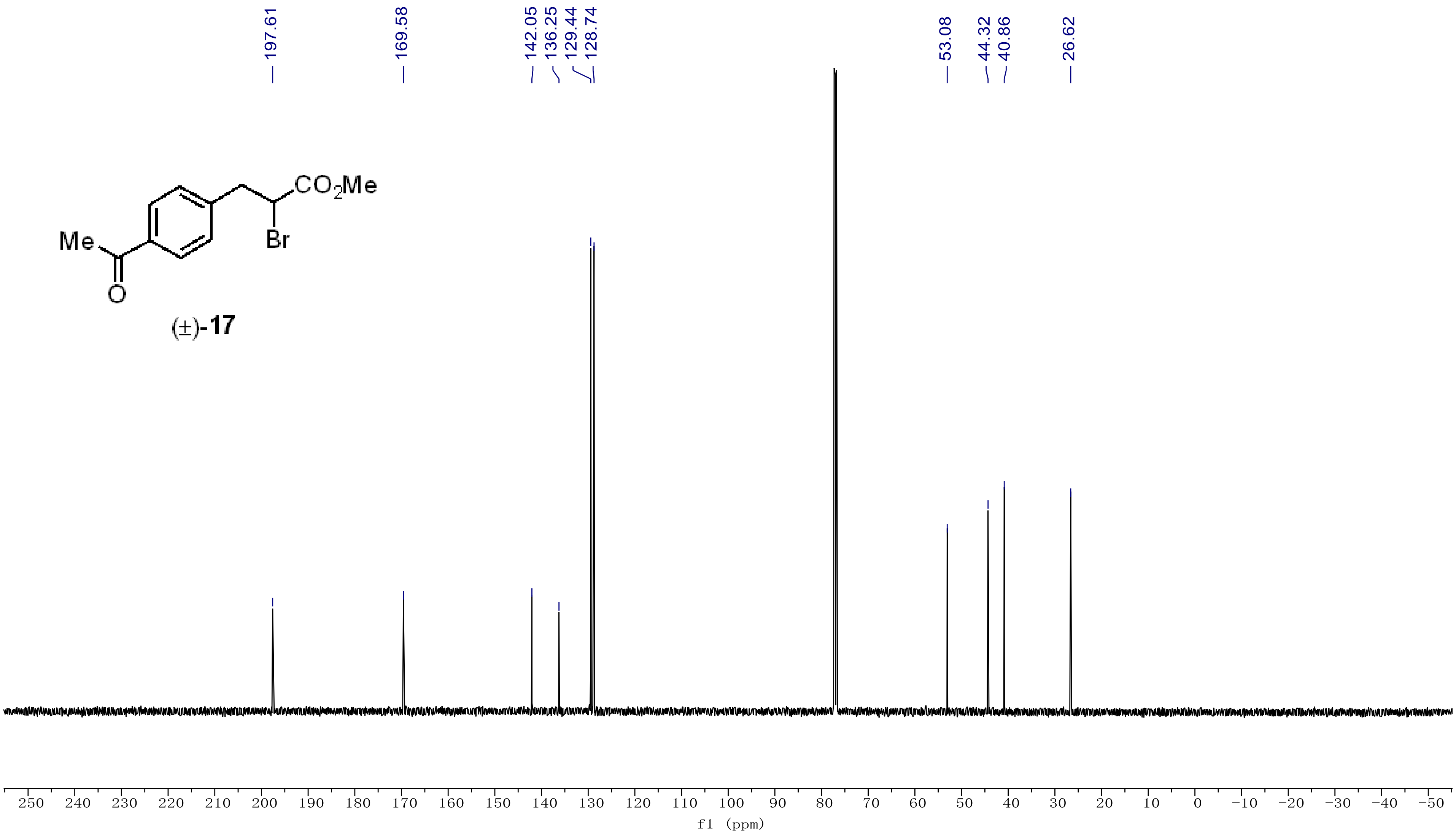

^{1}H NMR of (±)-2-bromo-arylpropanoate 18

$CDCl_3$, 23 ºC

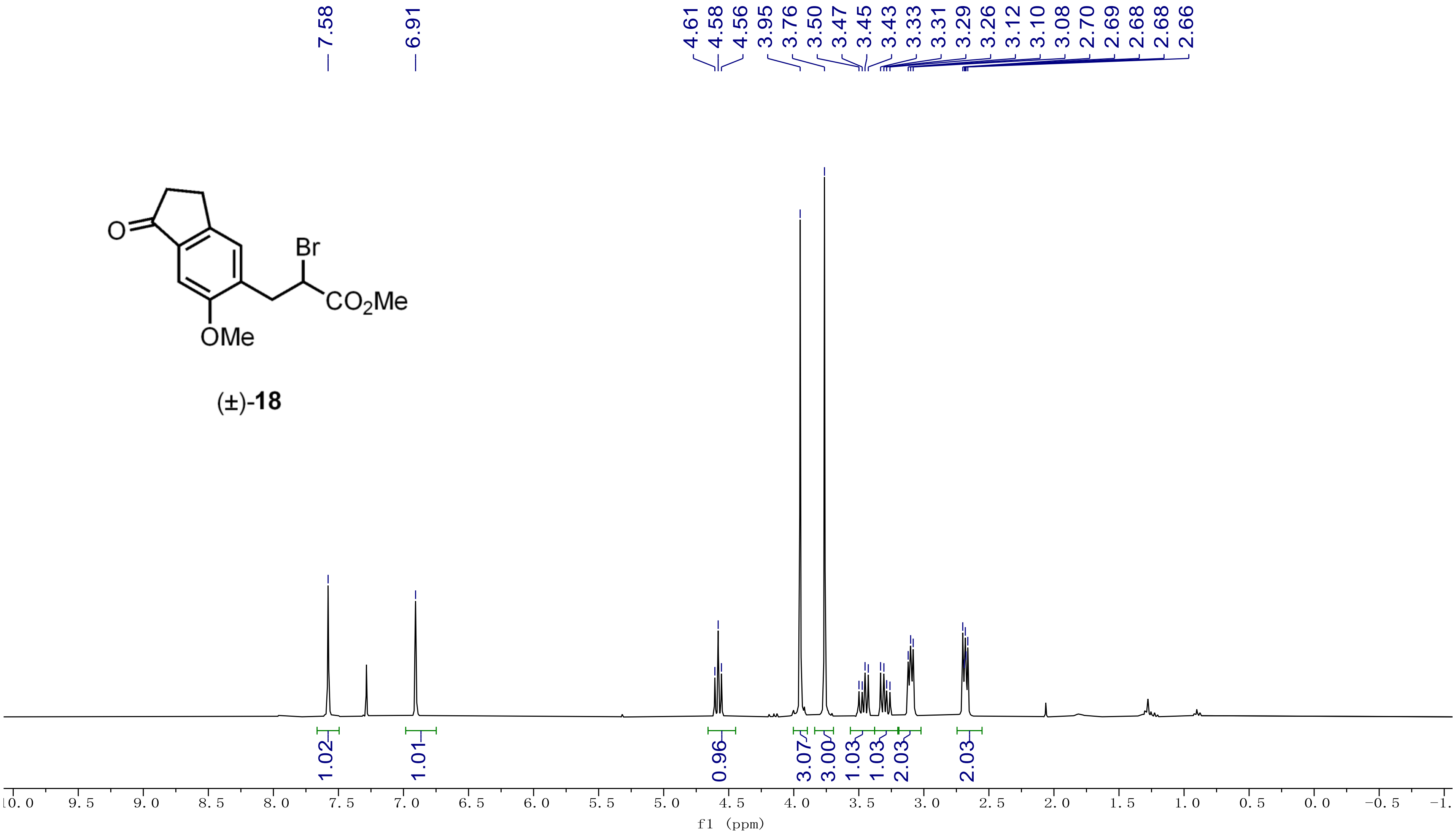

^{13}C NMR of (±)-2-bromo-arylpropanoate 18

CDCl$_3$, 23 ºC

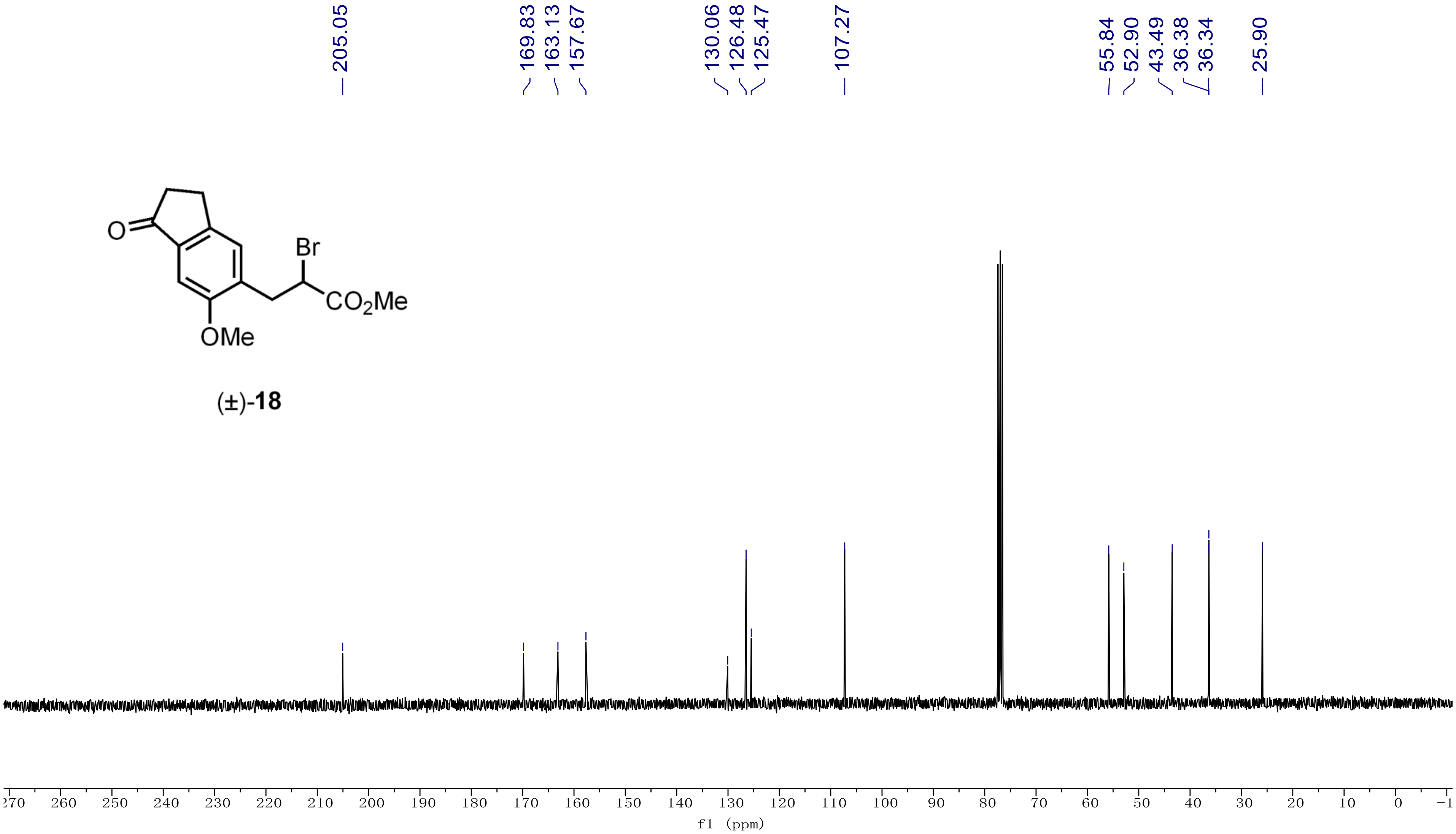

^{1}H NMR of (±)-2-bromo-arylpropanoate 19

$CDCl_3$, 23 ºC

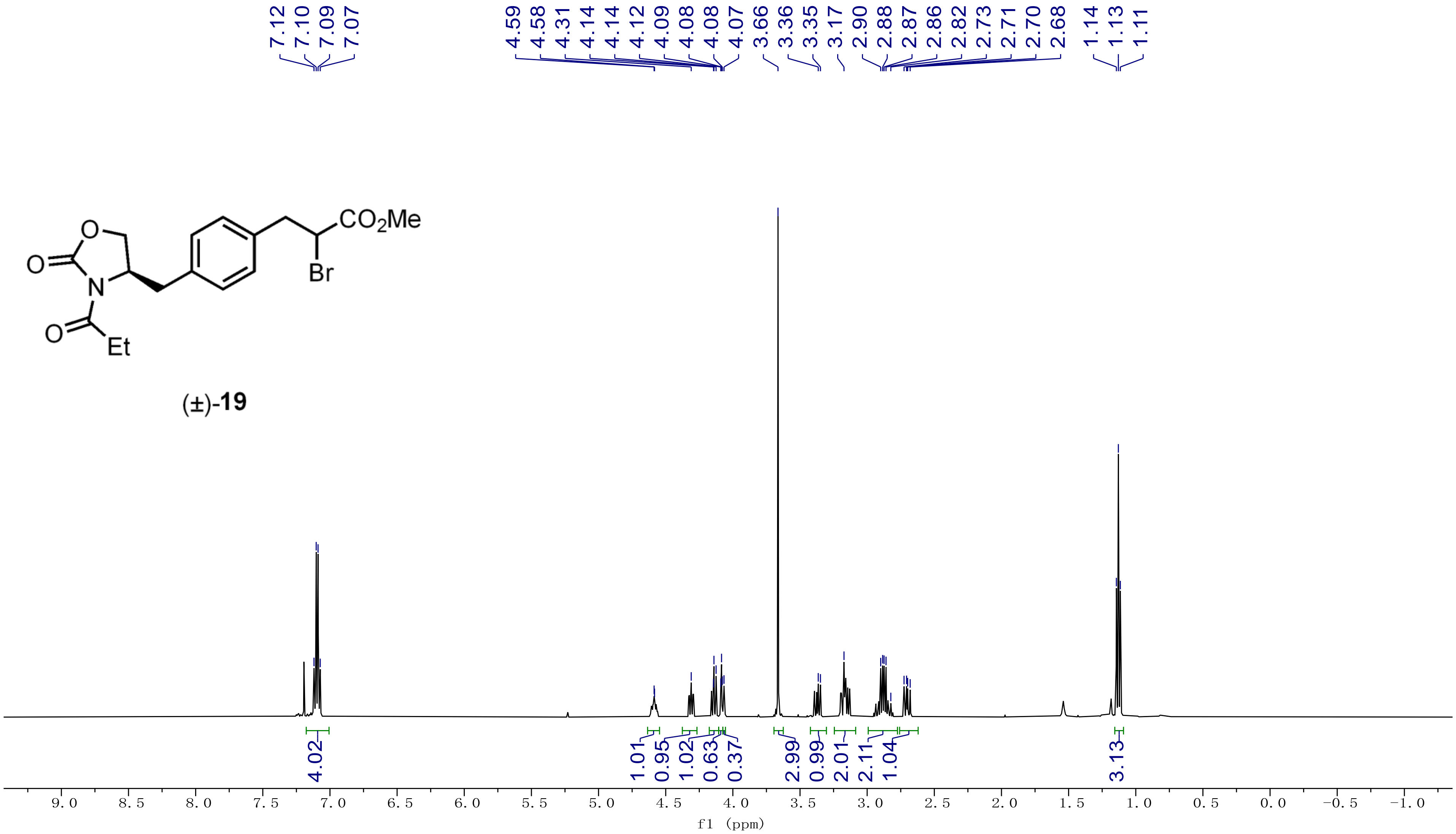

^{13}C NMR of (±)-2-bromo-arylpropanoate 19

$CDCl_3$, 23 ºC

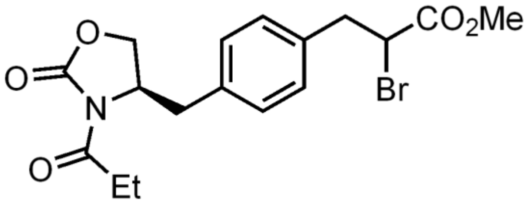

(±)-**19**

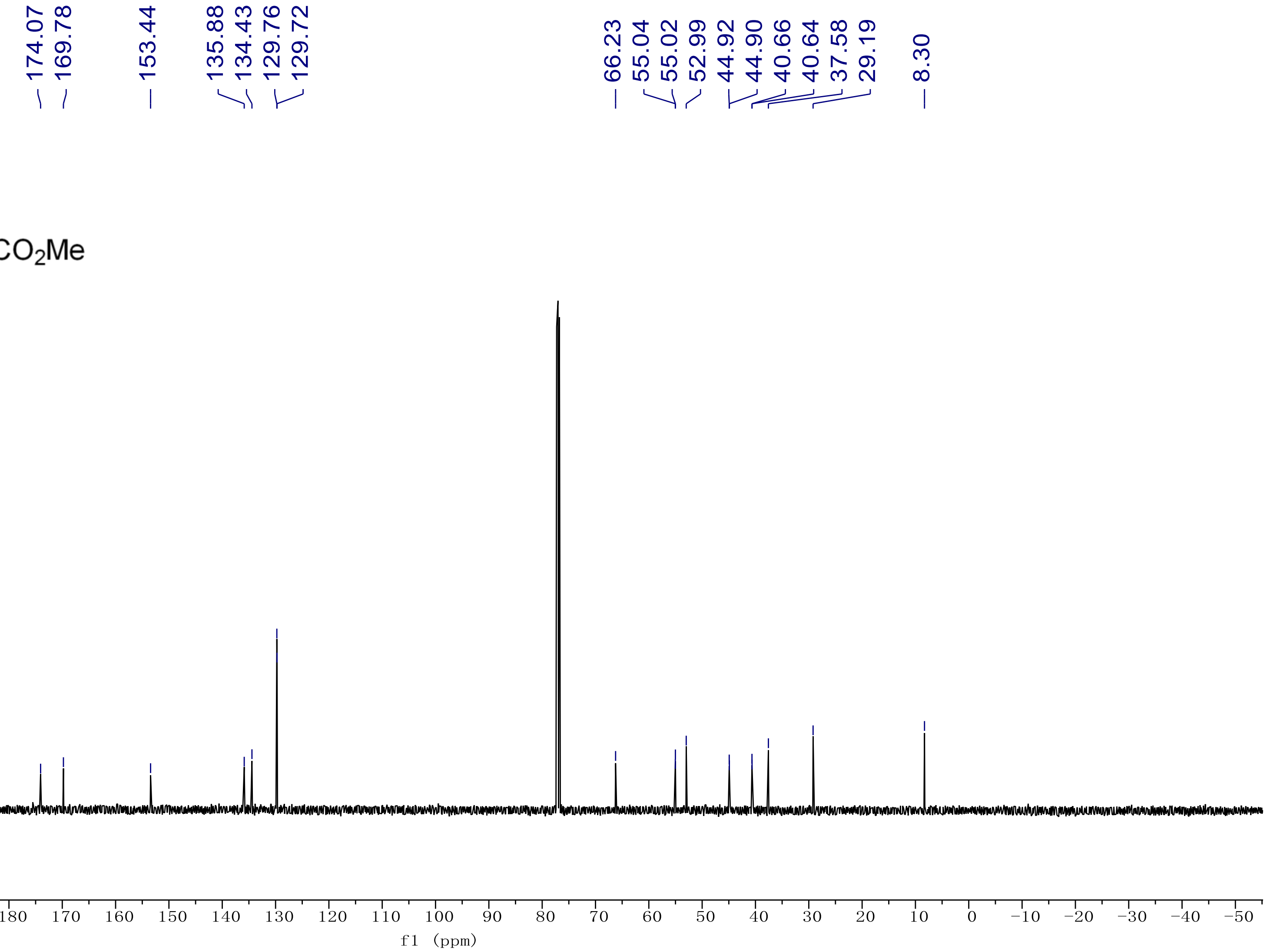

^{1}H NMR of (±)-pyriproxyphen derivative 20

CDCl$_3$, 23 ºC

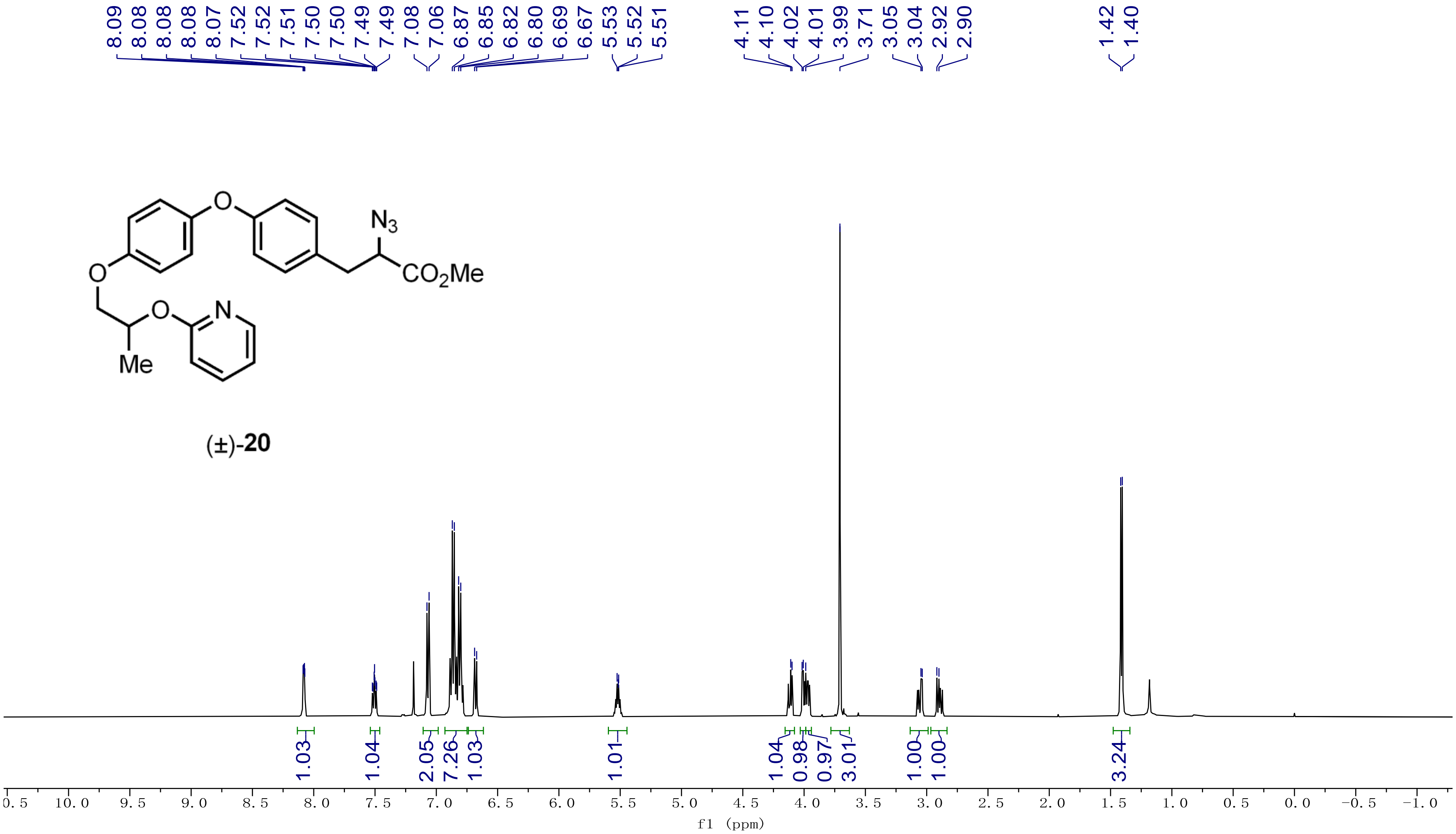

(±)-**20**

^{13}C NMR of (±)-pyriproxyphen derivative 20

$CDCl_3$, 23 ºC

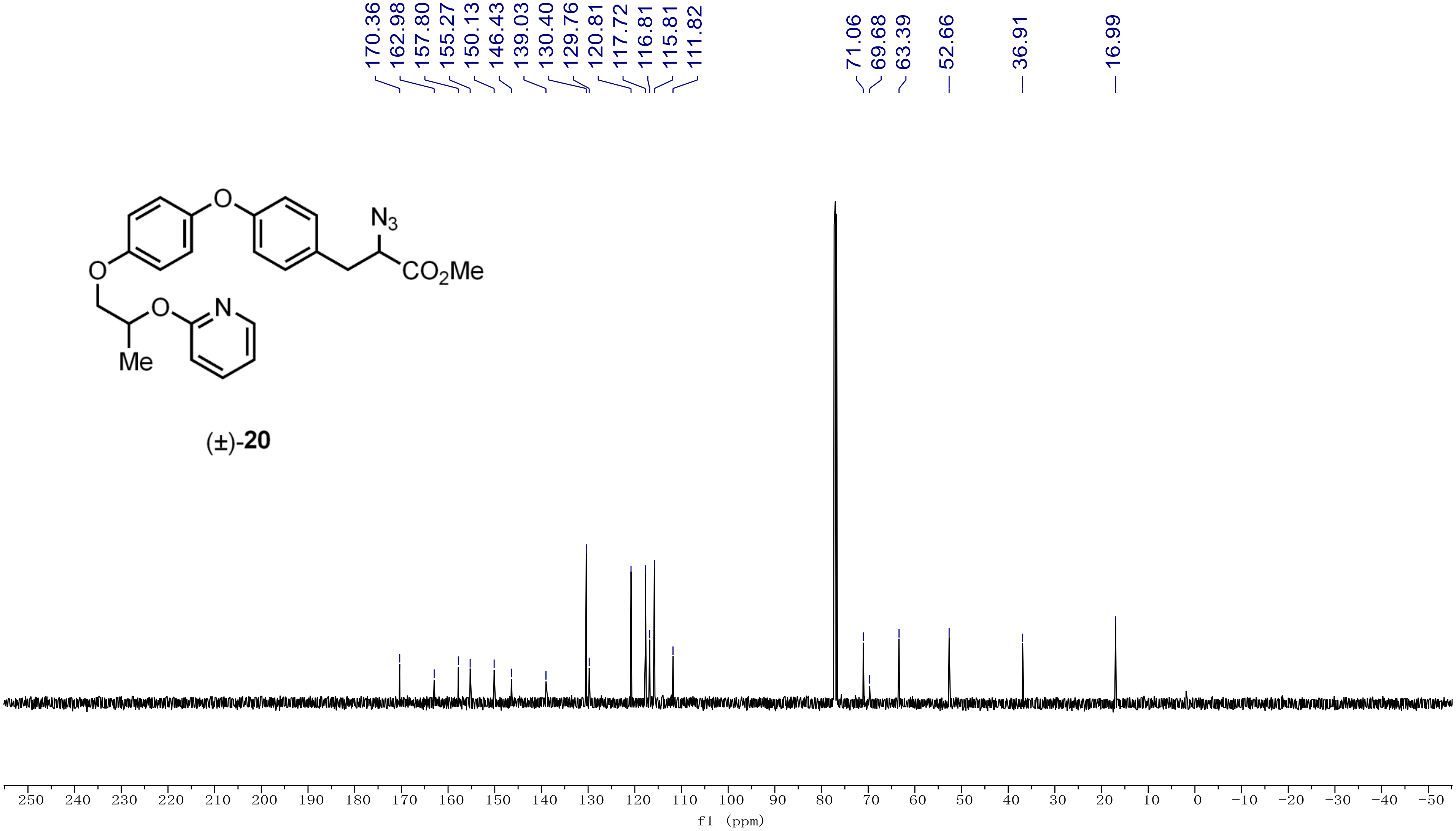

^{1}H NMR of (±)-benzbromarone derivative 21

CDCl$_3$, 23 ºC

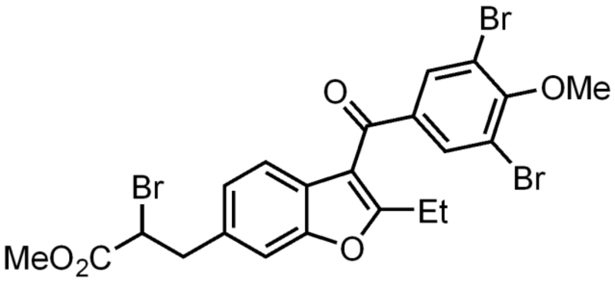

(±)-**21**

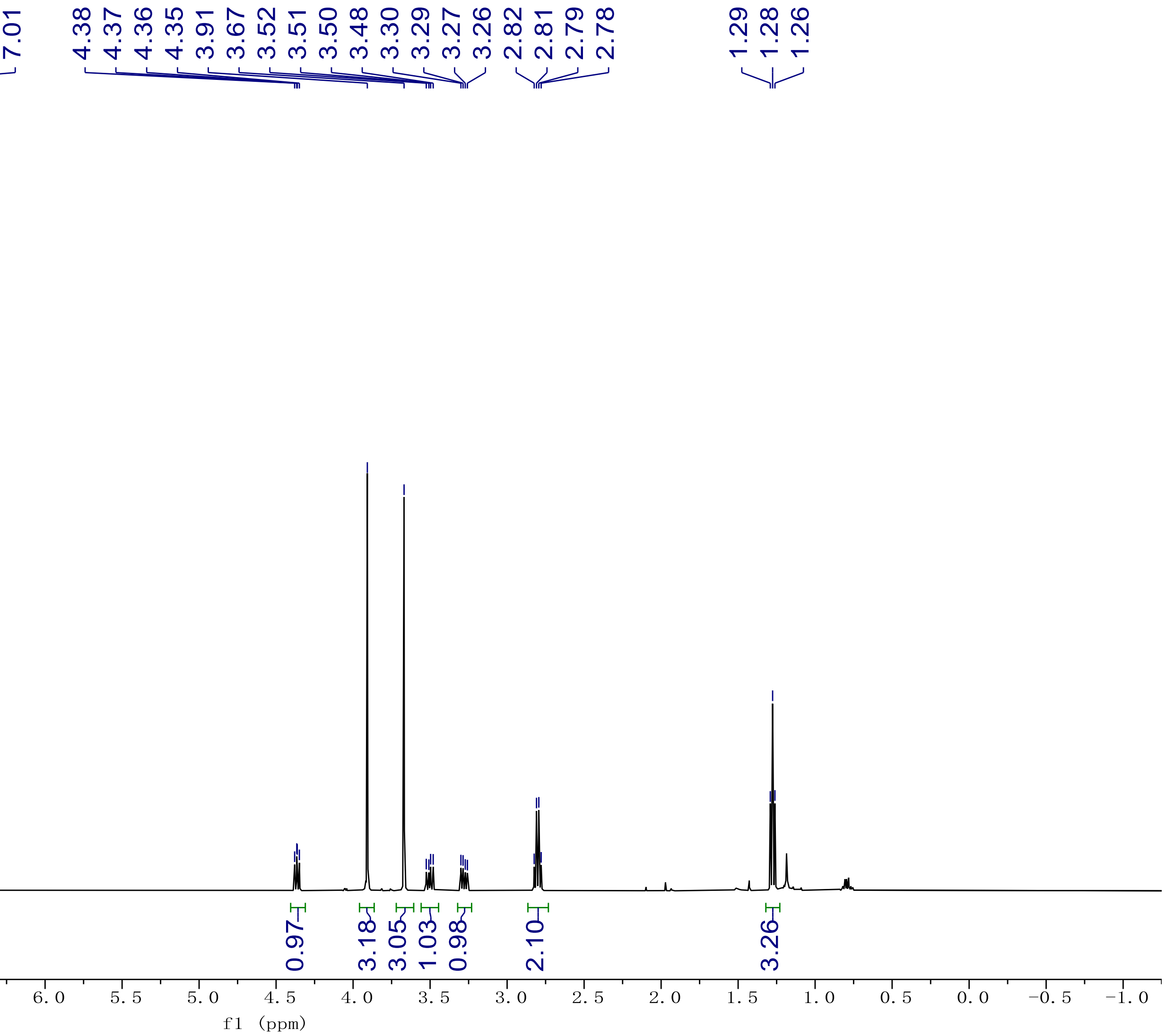

^{13}C NMR of (±)-benzbromarone derivative 21

$CDCl_3$, 23 ºC

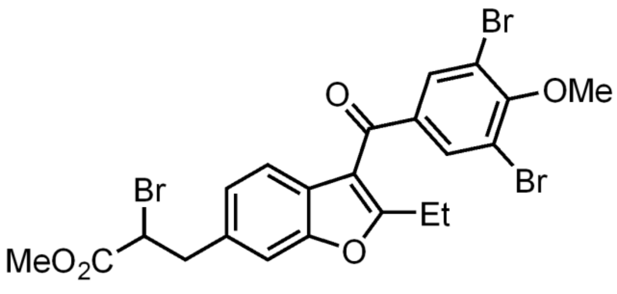

(±)-**21**

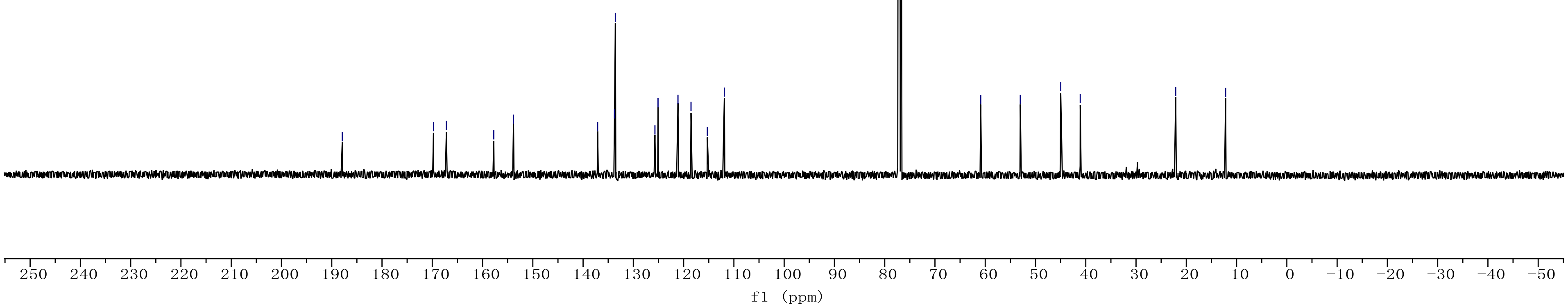

^{1}H NMR of (±)-2-bromo-arylpropanoate 22

$CDCl_3$, 23 ºC

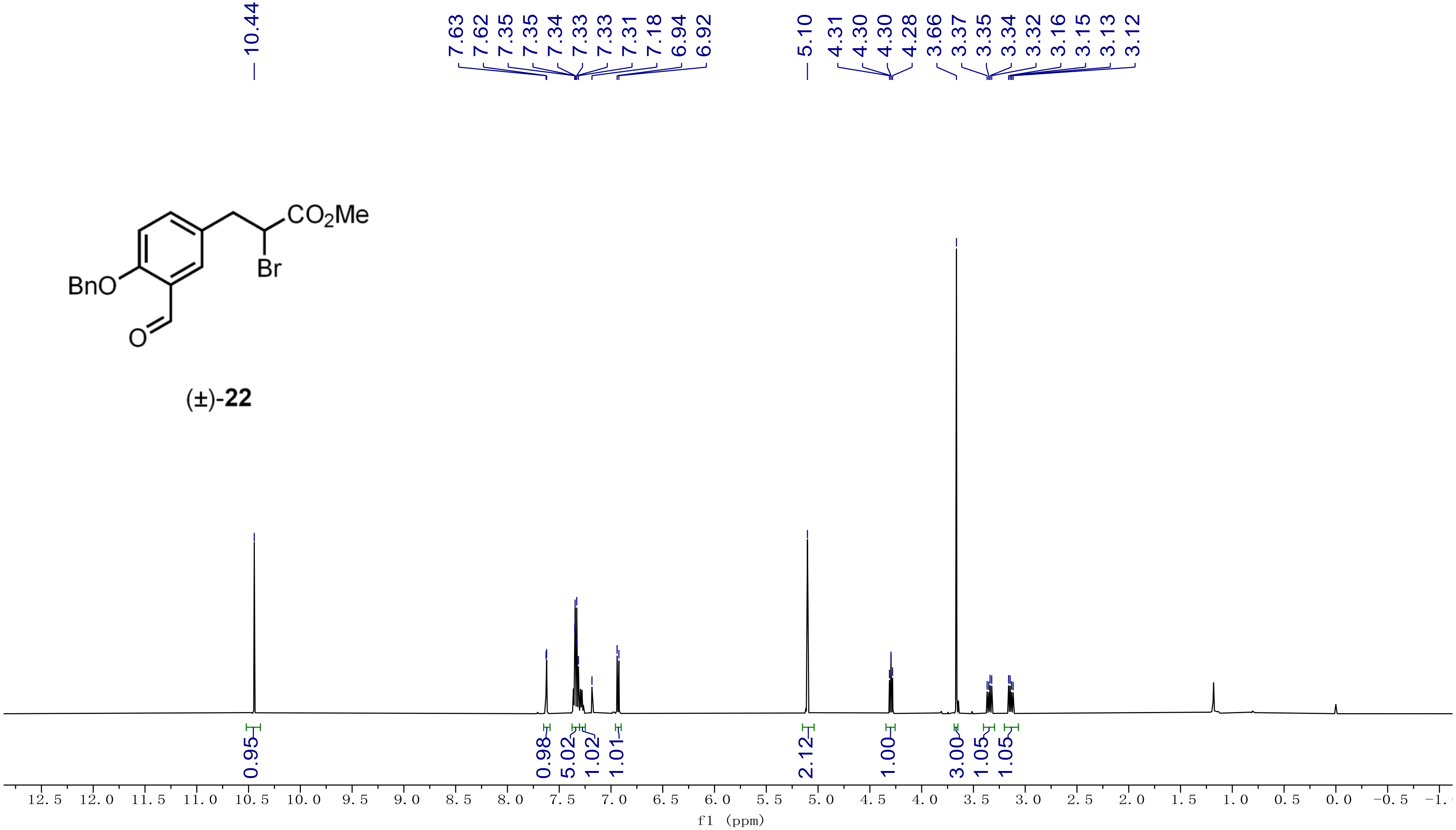

^{13}C NMR of (±)-2-bromo-arylpropanoate 22

CDCl3, 23 ºC

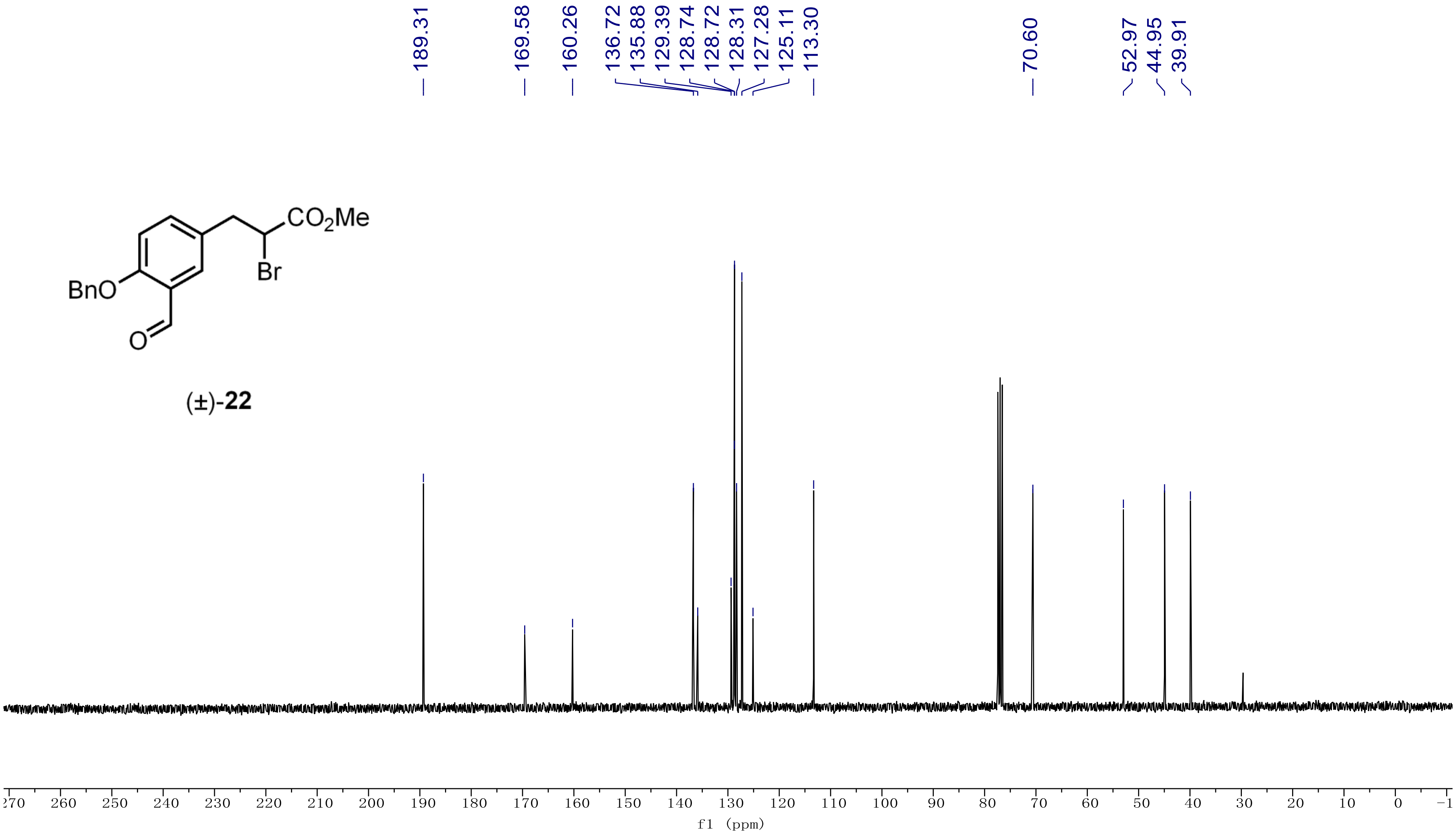

(±)-**22**

^{1}H NMR of (±)-2-bromo-arylpropanoate 23

$CDCl_3$, 23 ºC

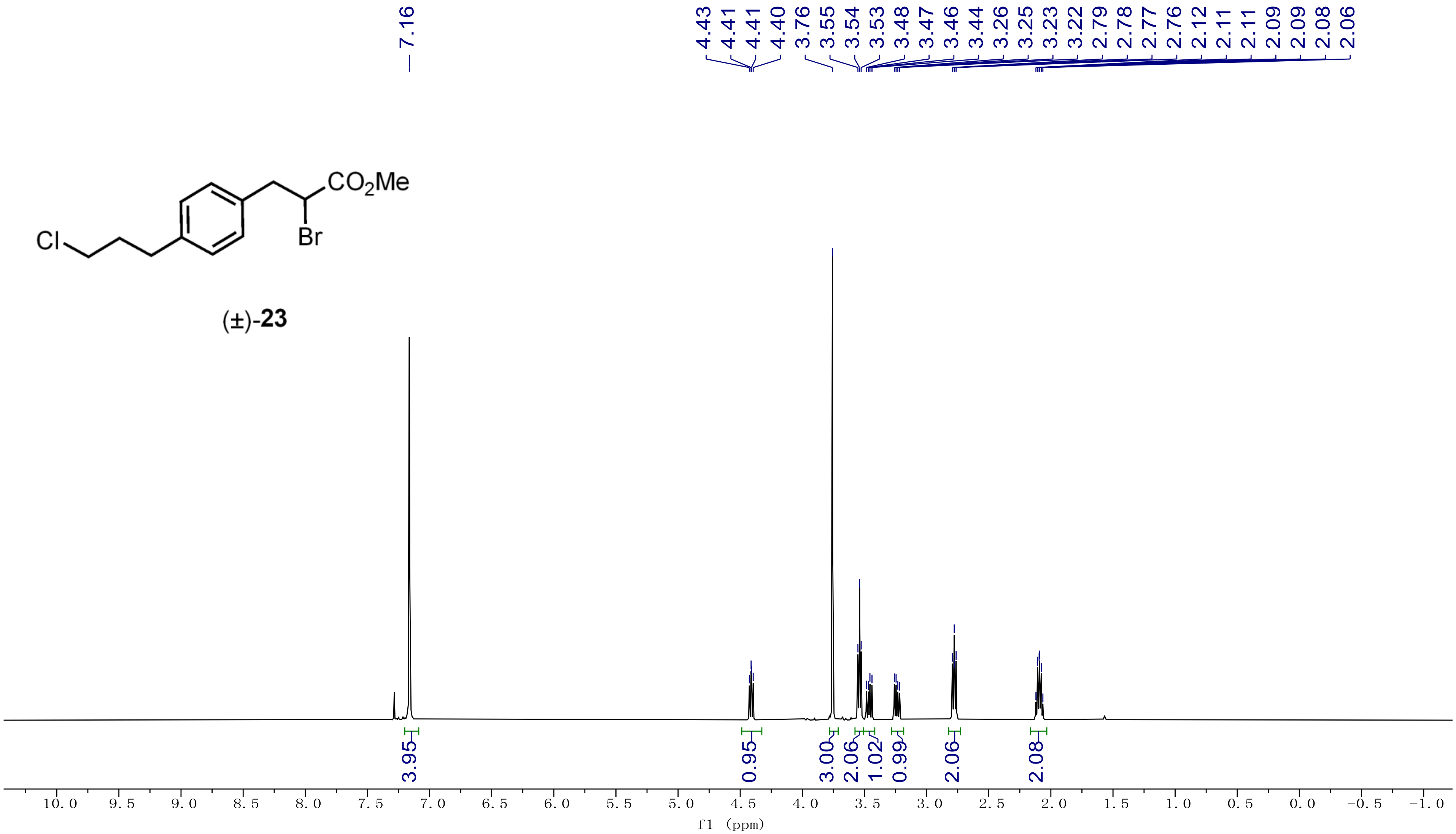

(±)-**23**

^{13}C NMR of (±)-2-bromo-arylpropanoate 23

$CDCl_3$, 23 ºC

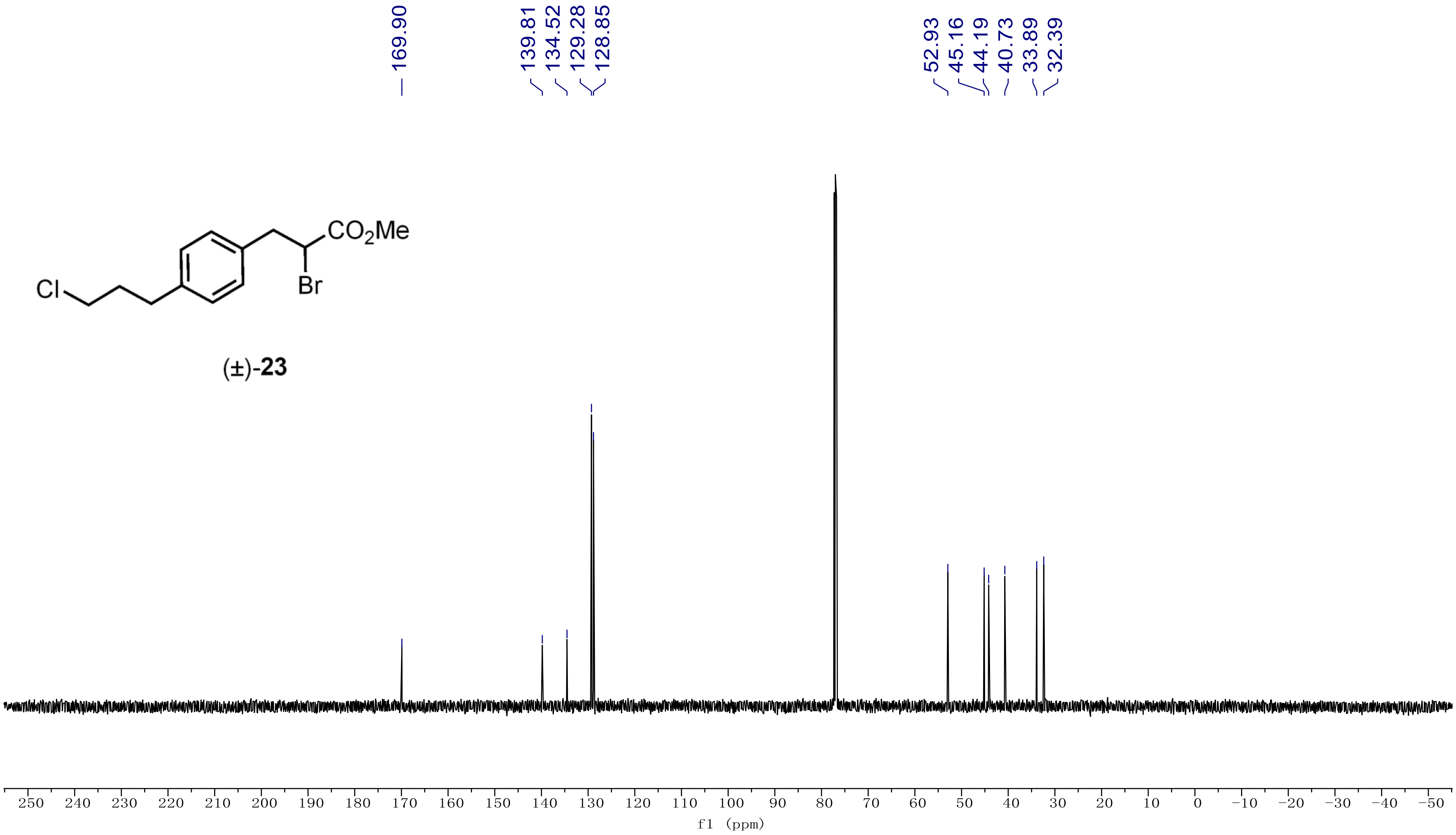

^{1}H NMR of (±)-2-bromo-arylpropanoate 24

$CDCl_3$, 23 ºC

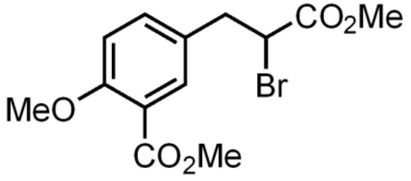

(±)-**24**

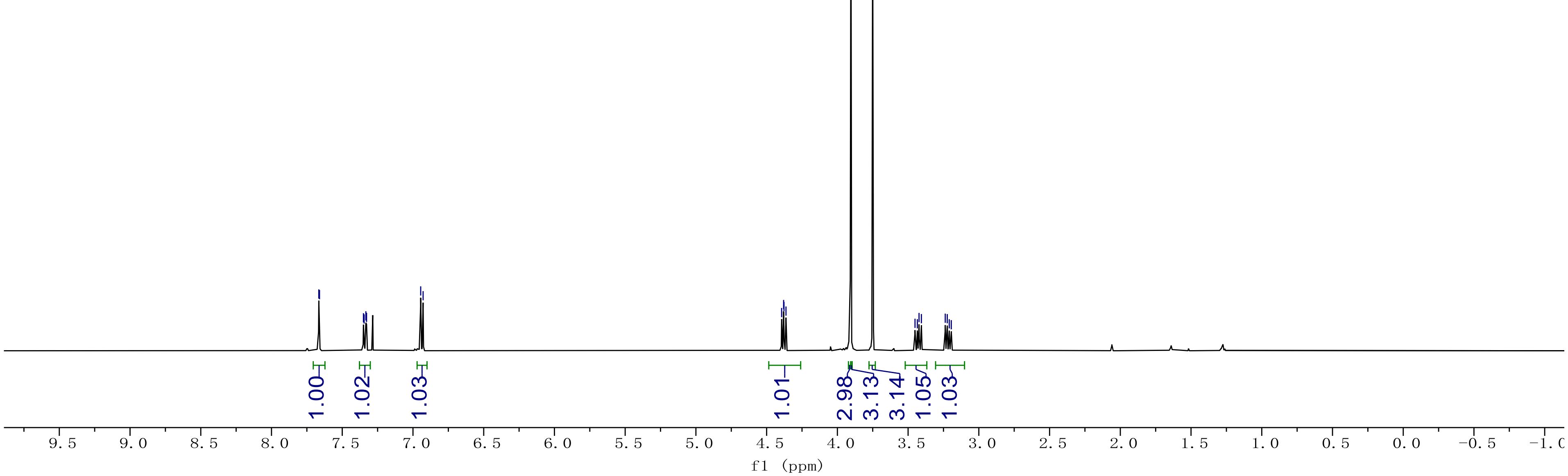

^{13}C NMR of (±)-2-bromo-arylpropanoate 24

CDCl$_3$, 23 ºC

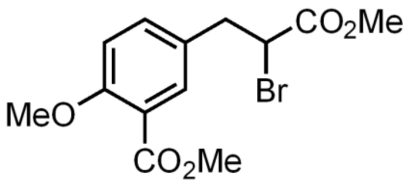

(±)-**24**

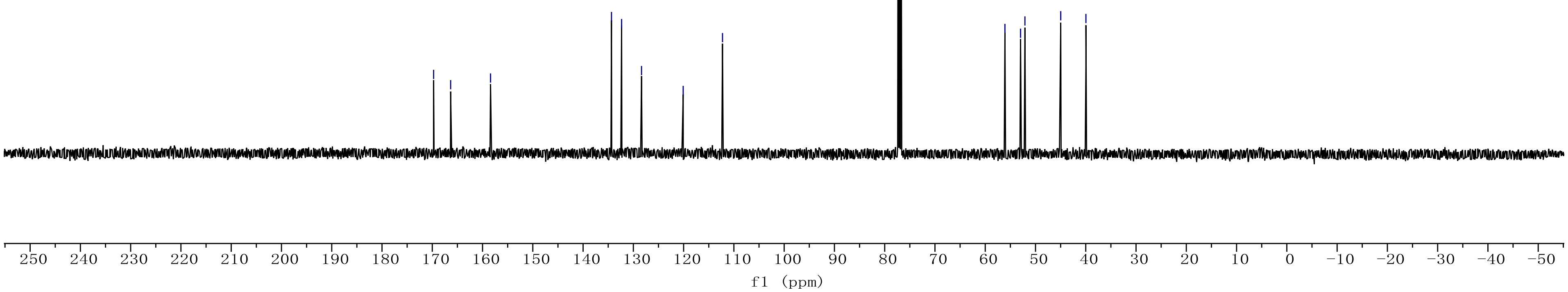

^{1}H NMR of (±)-2-bromo-arylpropanoate 25

CDCl3, 23 ºC

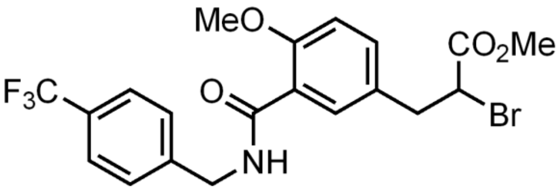

(±)-**25**

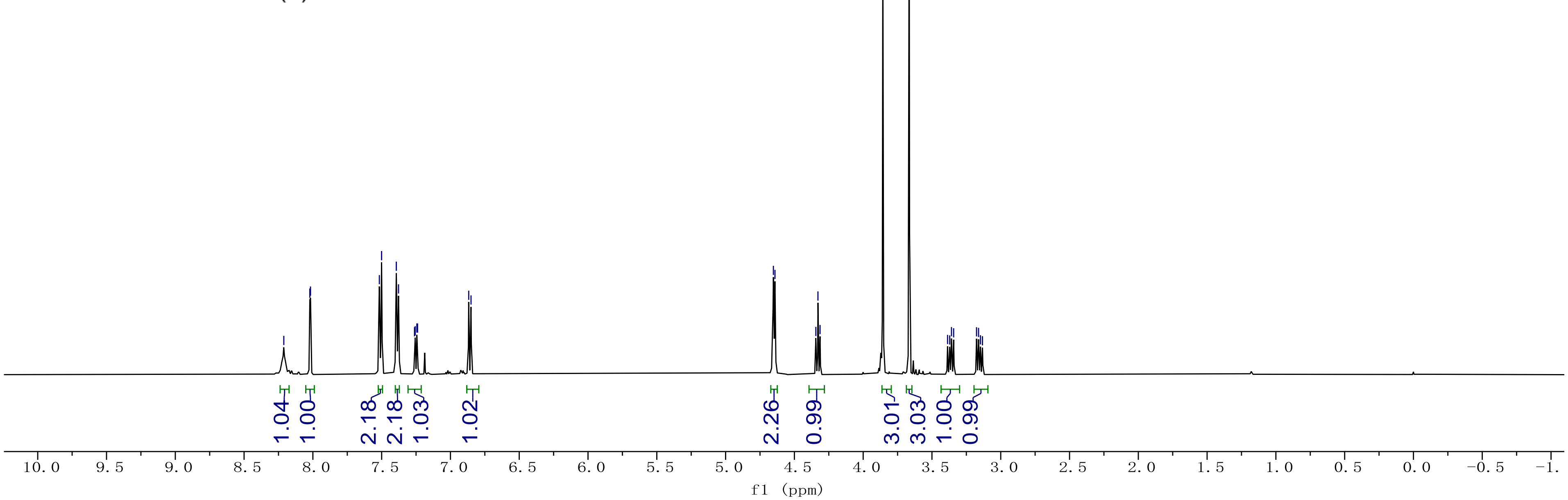

^{13}C NMR of (±)-2-bromo-arylpropanoate 25

CDCl$_3$, 23 ºC

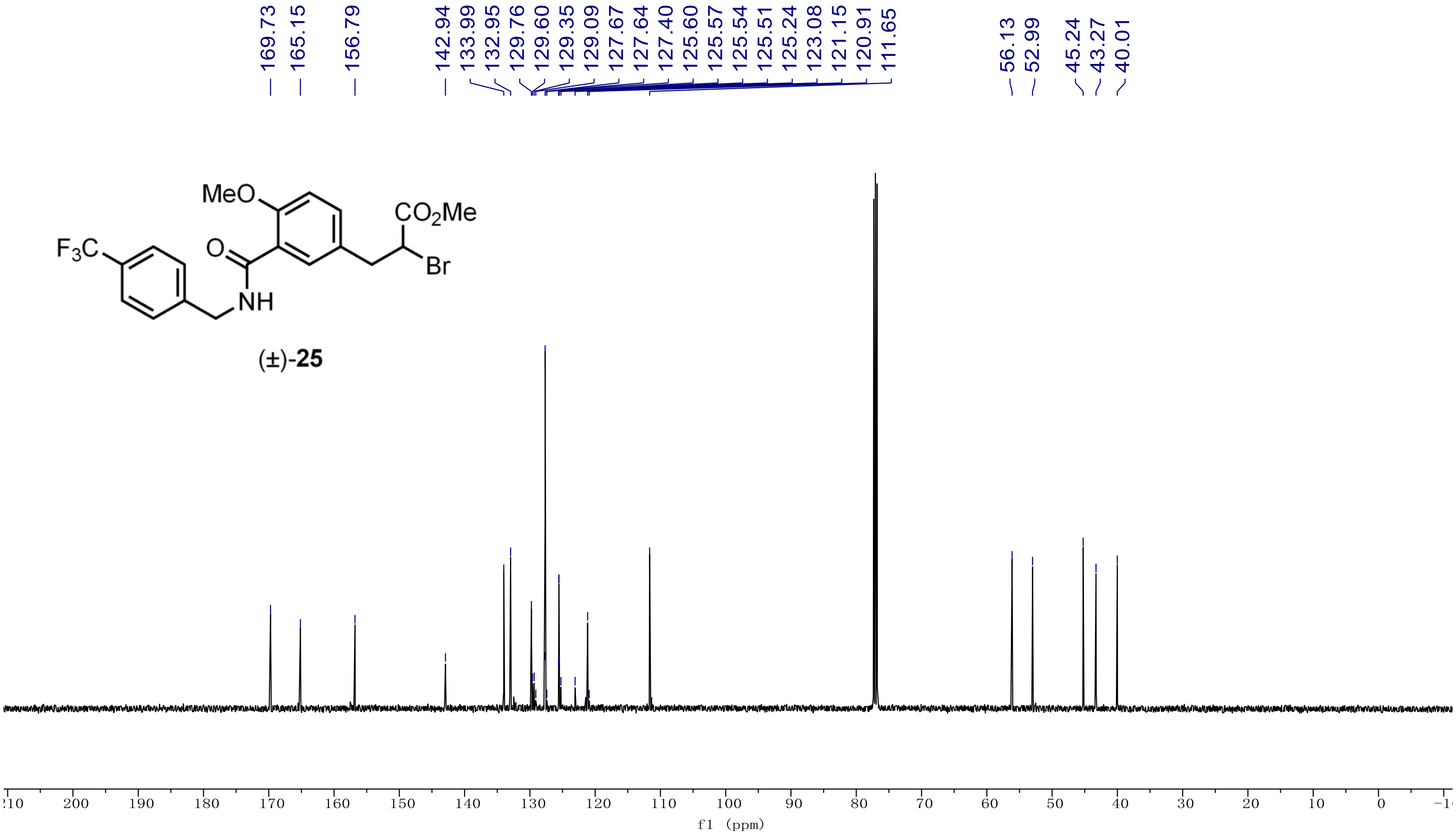

^{19}F NMR of (±)-2-bromo-arylpropanoate 25

$CDCl_3$, 23 ºC

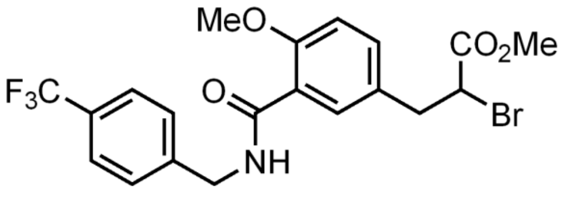

(±)-**25**

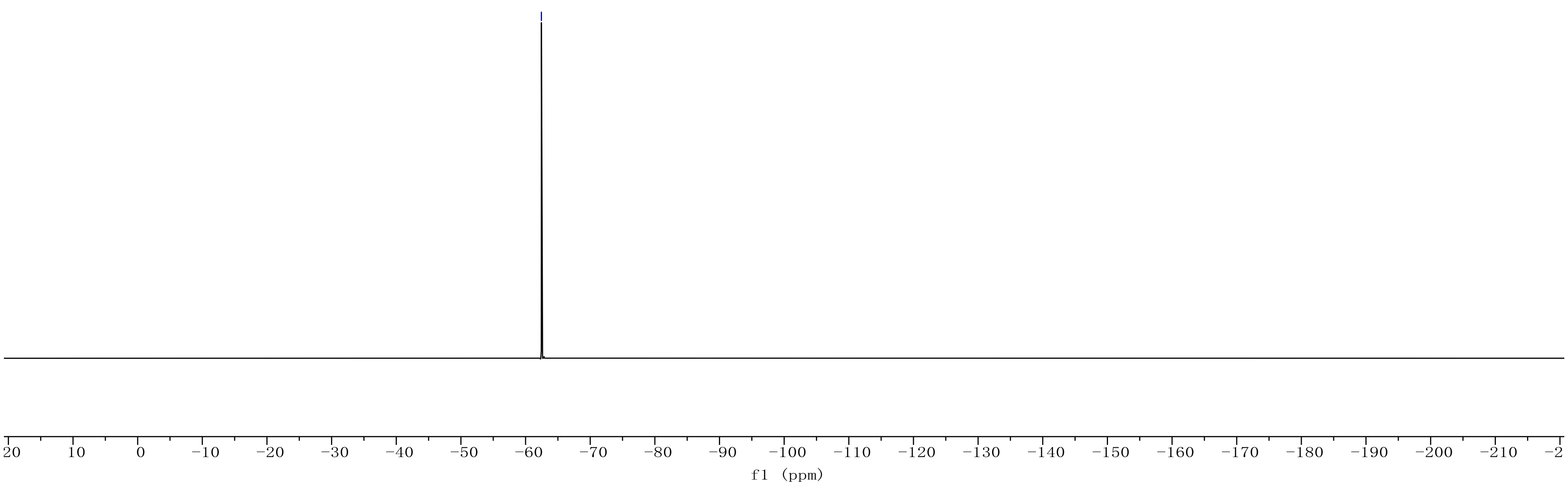

^{1}H NMR of (±)-Indometacin methylester derivative 26

CDCl3, 23 ºC

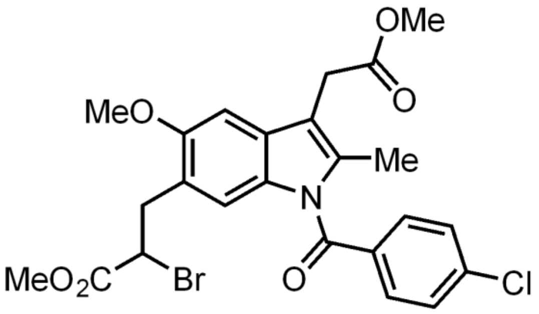

(±)-**26**

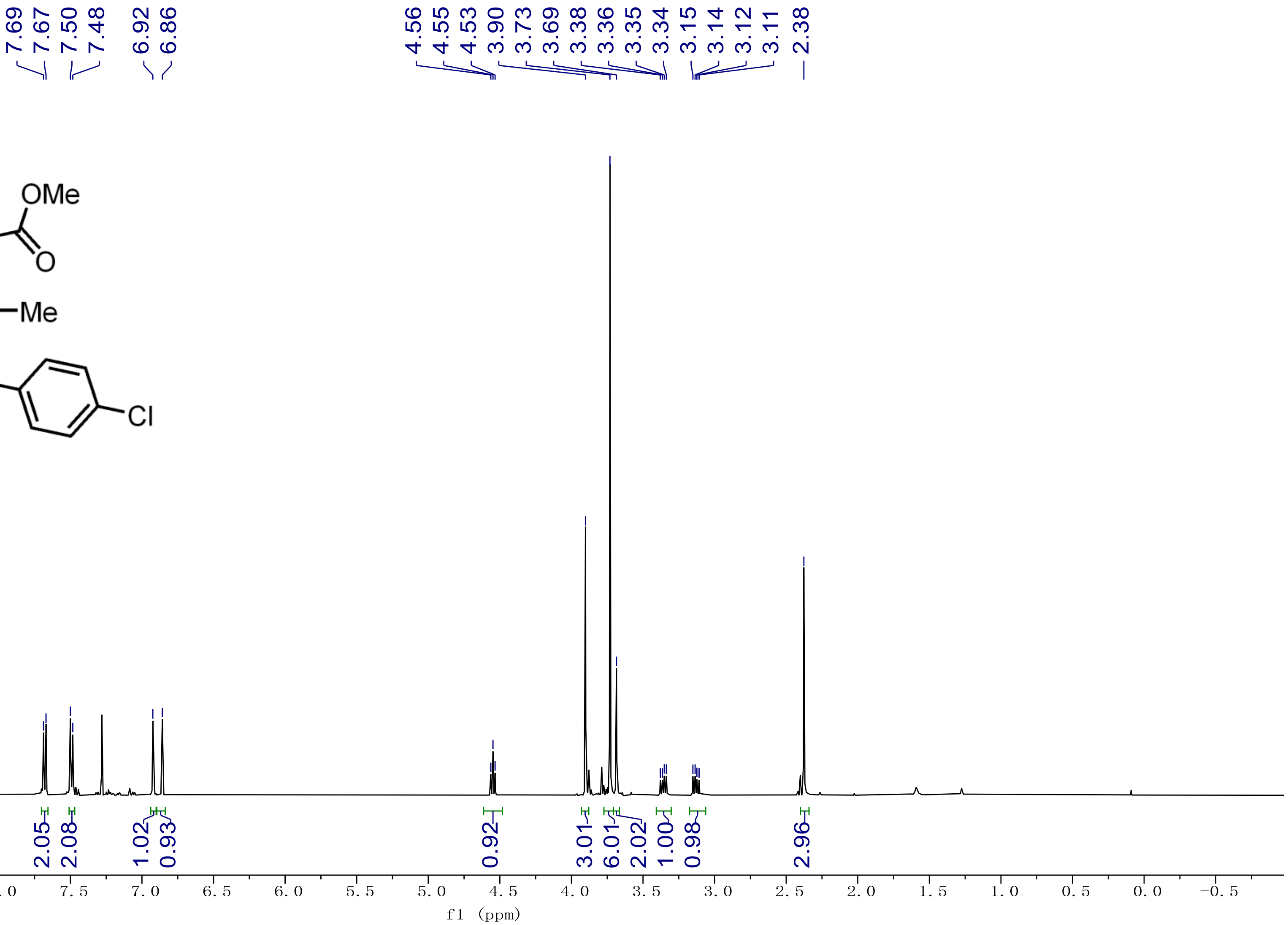

^{13}C NMR of (±)-Indometacin methylester derivative 26

$CDCl_3$, 23 ºC

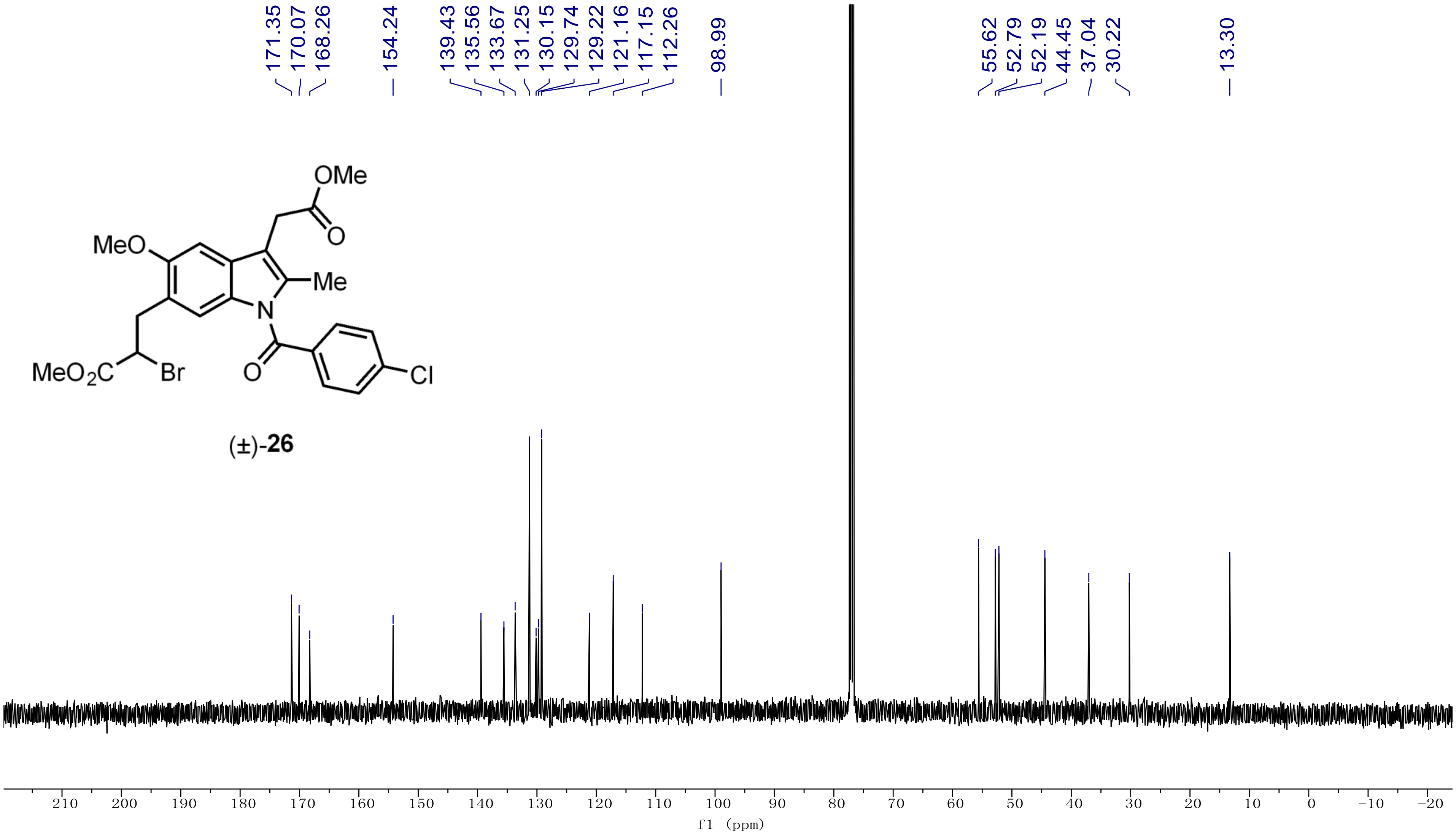

^{1}H NMR of (±)-2-bromo-arylpropanoate 27

$CDCl_3$, 23 ºC

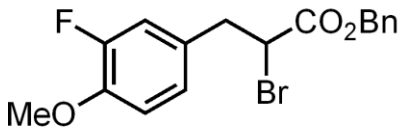

(±)-**27**

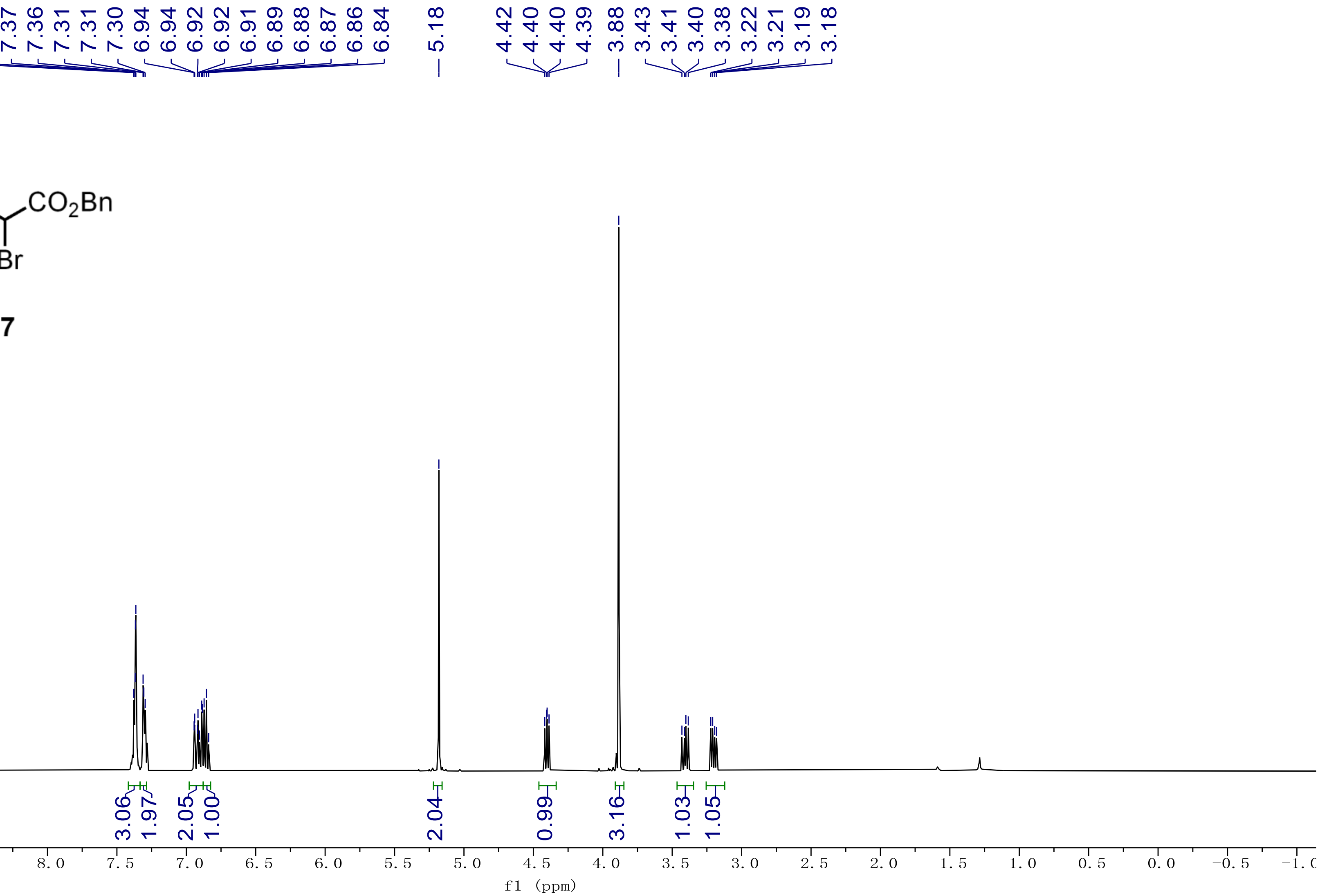

^{13}C NMR of (±)-2-bromo-arylpropanoate 27

CDCl3, 23 ºC

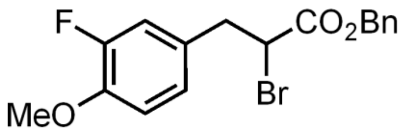

(±)-**27**

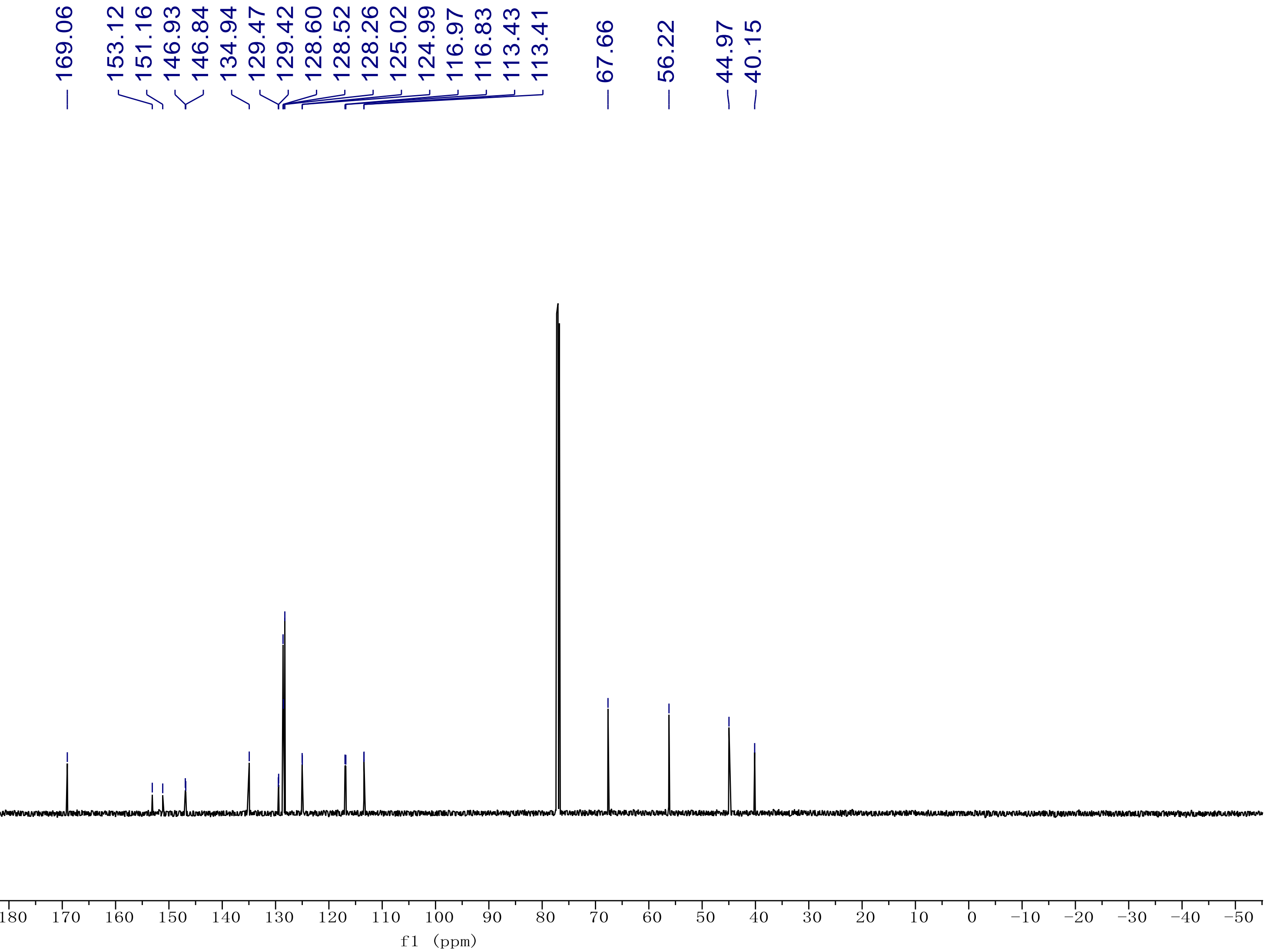

^{19}F NMR of (±)-2-bromo-arylpropanoate 27

$CDCl_3$, 23 ºC

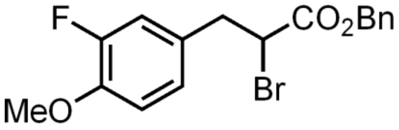

(±)-**27**

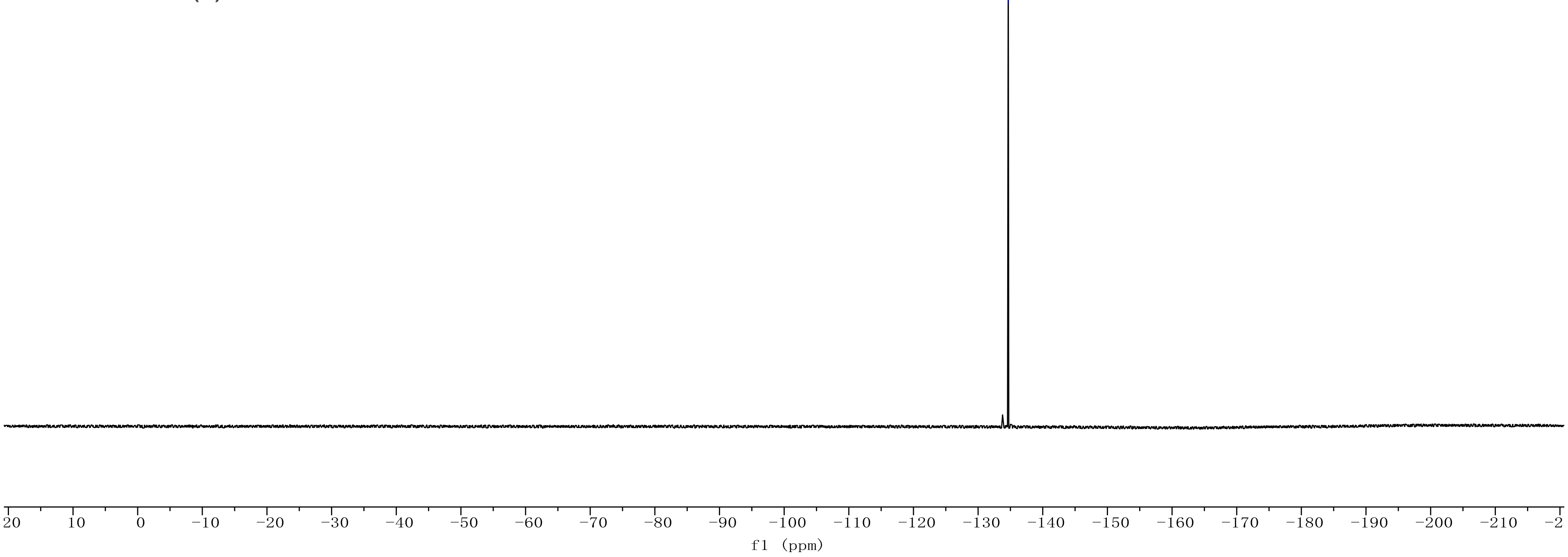

^{1}H NMR of (±)-2-bromo-arylpropanoate 28

$CDCl_3$, 23 ºC

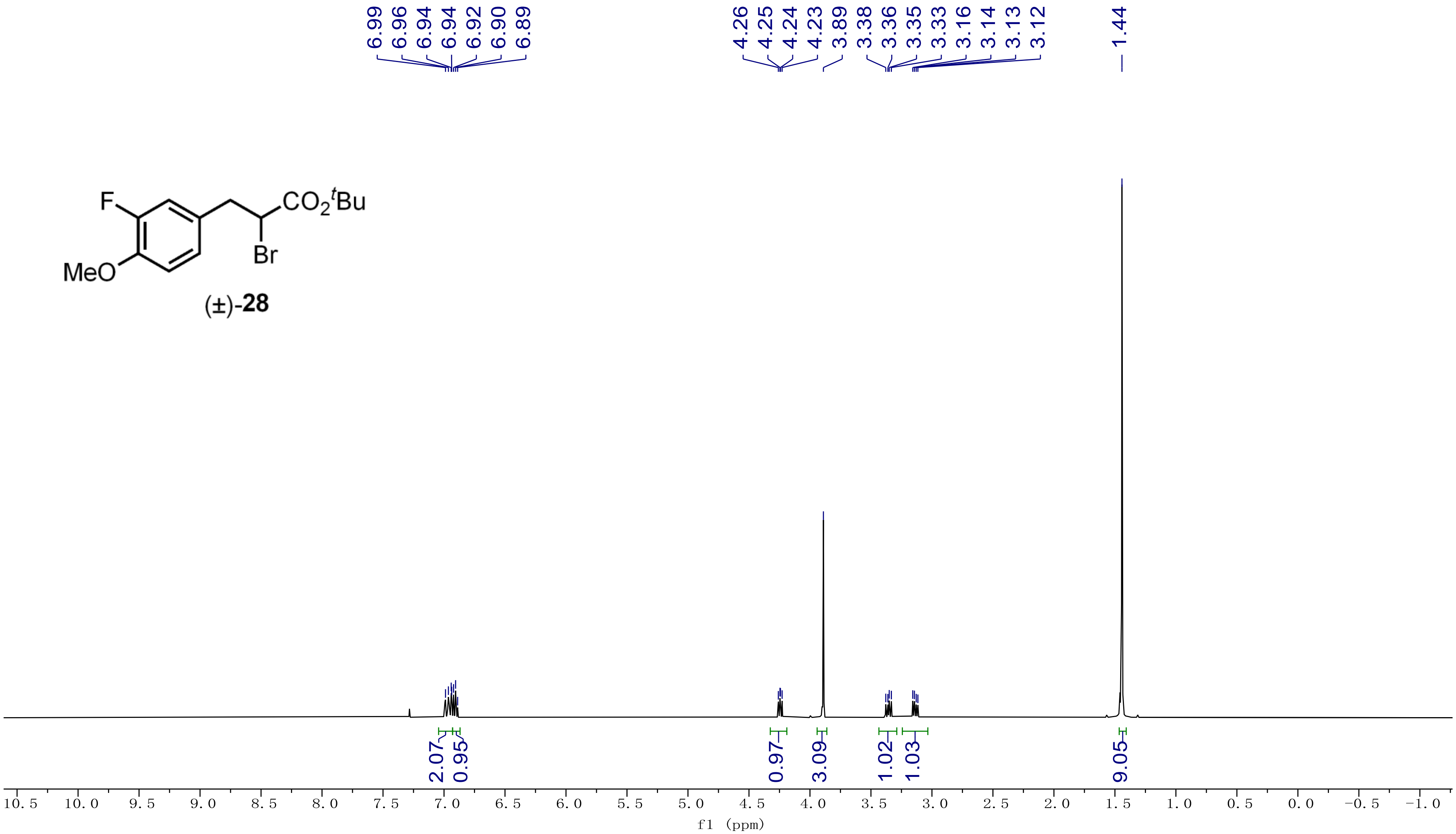

^{13}C NMR of (±)-2-bromo-arylpropanoate 28

$CDCl_3$, 23 ºC

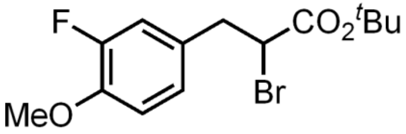

(±)-**28**

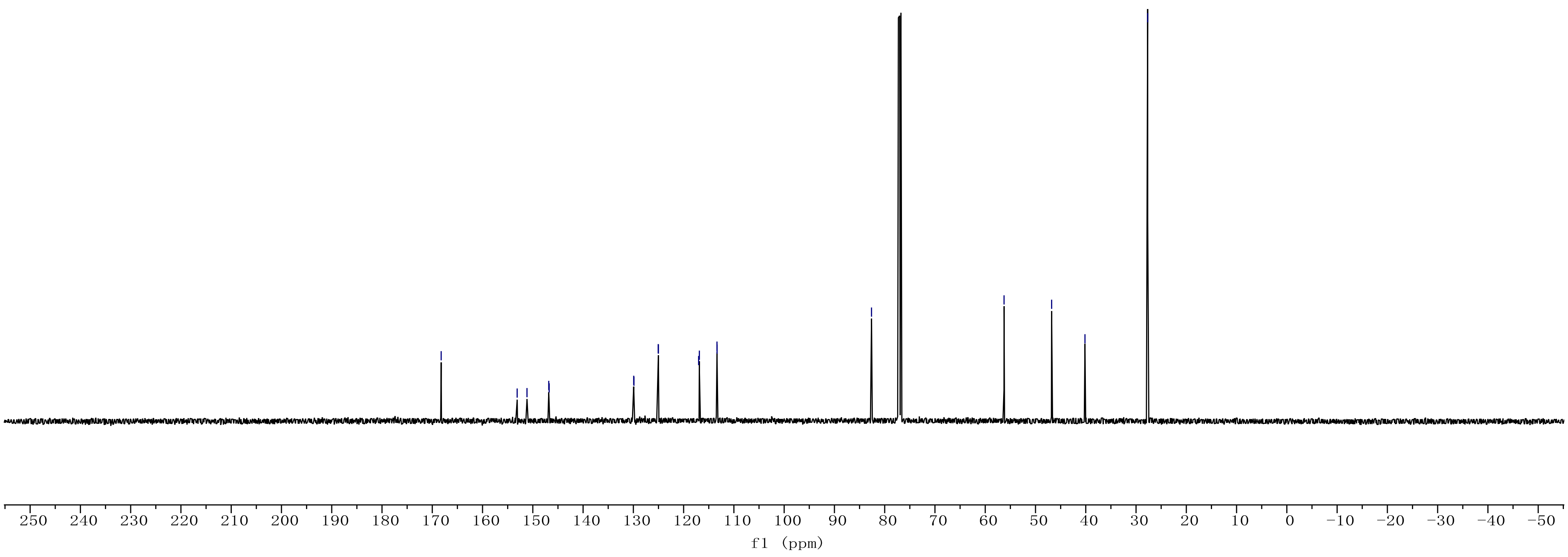

^{19}F NMR of (±)-2-bromo-arylpropanoate 28

$CDCl_3$, 23 ºC

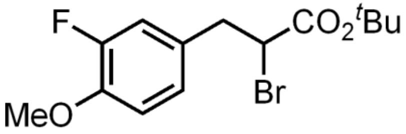

(±)-**28**

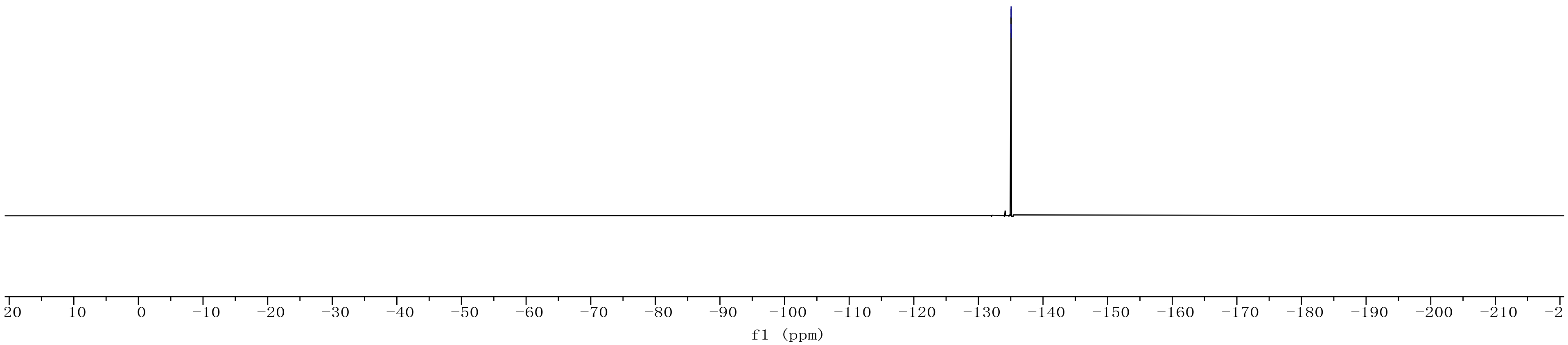

^{1}H NMR of (±)-2-bromo-arylpropanenitrile 29

CDCl$_3$, 23 ºC

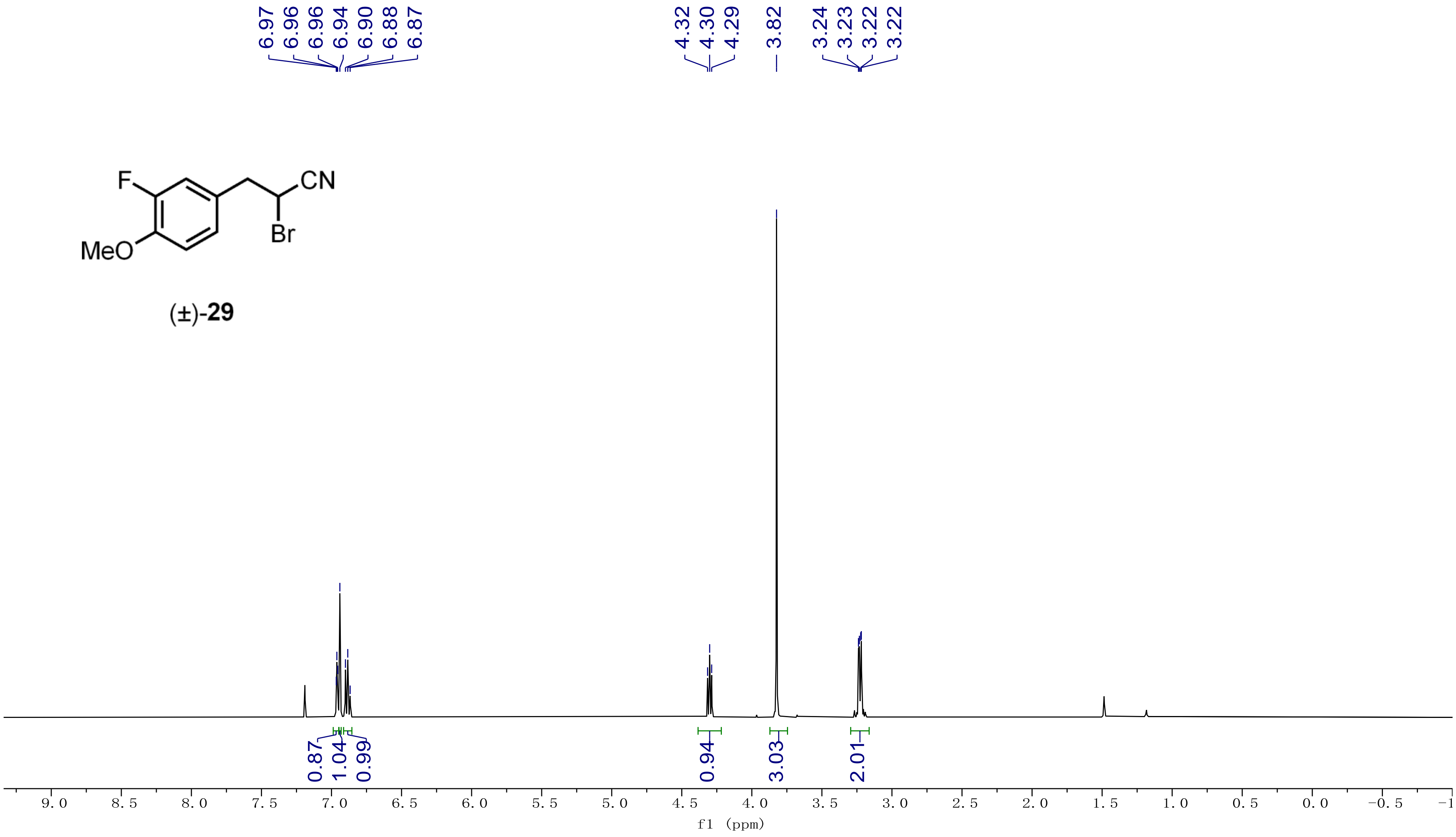

^{13}C NMR of (±)-2-bromo-arylpropanenitrile 29

$CDCl_3$, 23 ºC

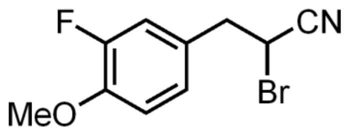

(±)-**29**

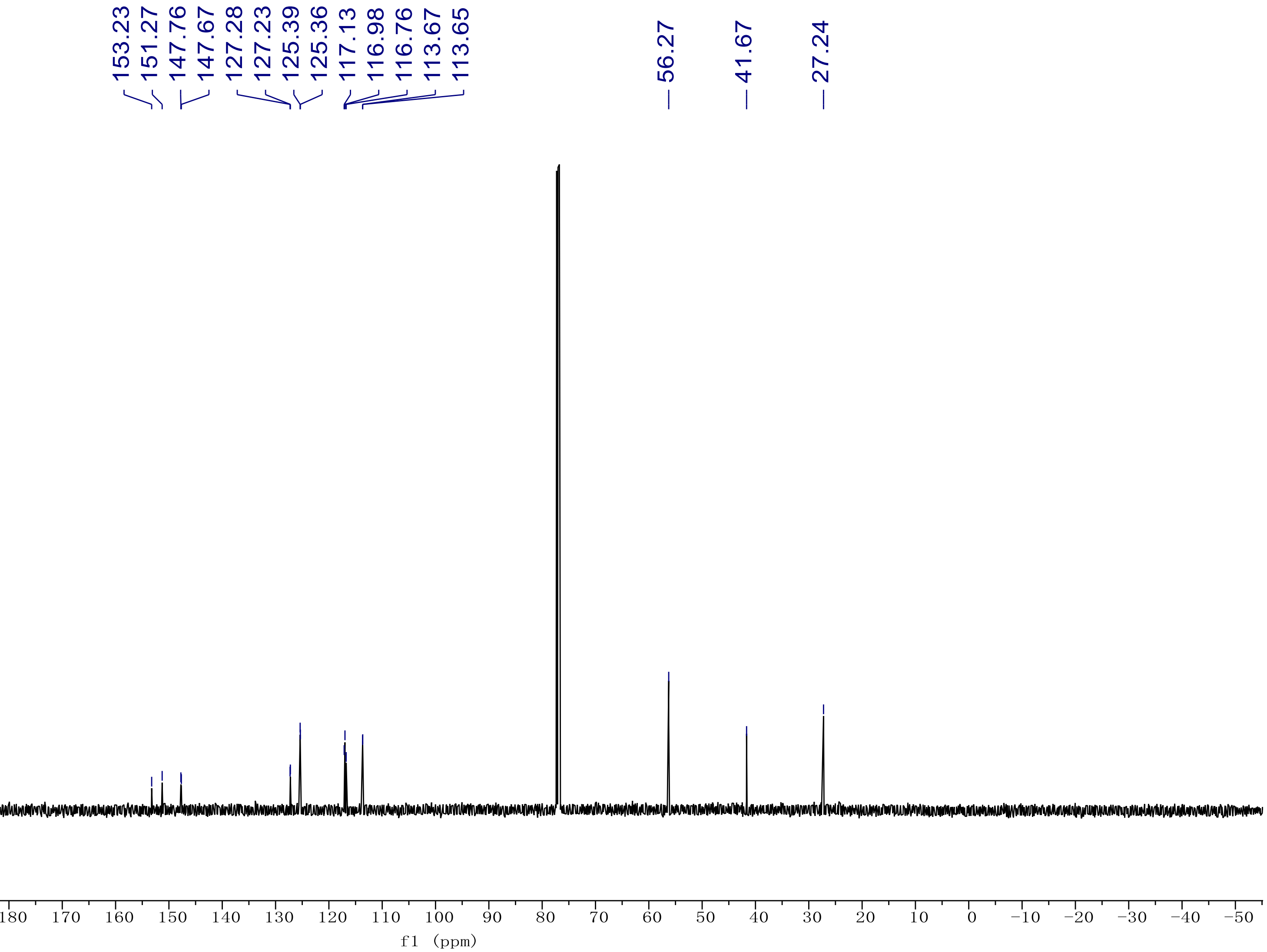

^{19}F NMR of (±)-2-bromo-arylpropanenitrile 29

$CDCl_3$, 23 ºC

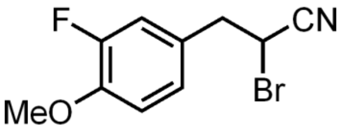

(±)-**29**

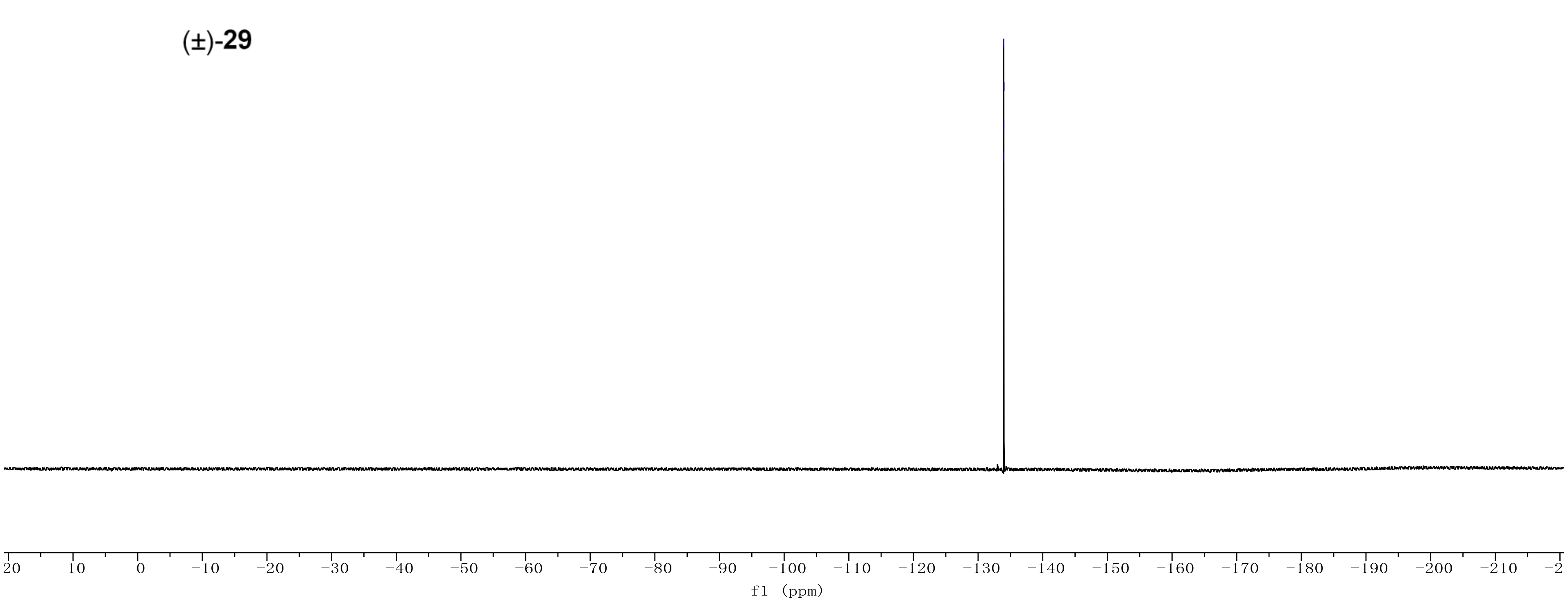

^{1}H NMR of (±)-3-bromo-arylbutan-2-one 30

CDCl3, 23 ºC

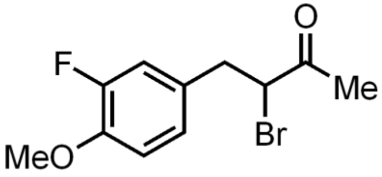

(±)-**30**

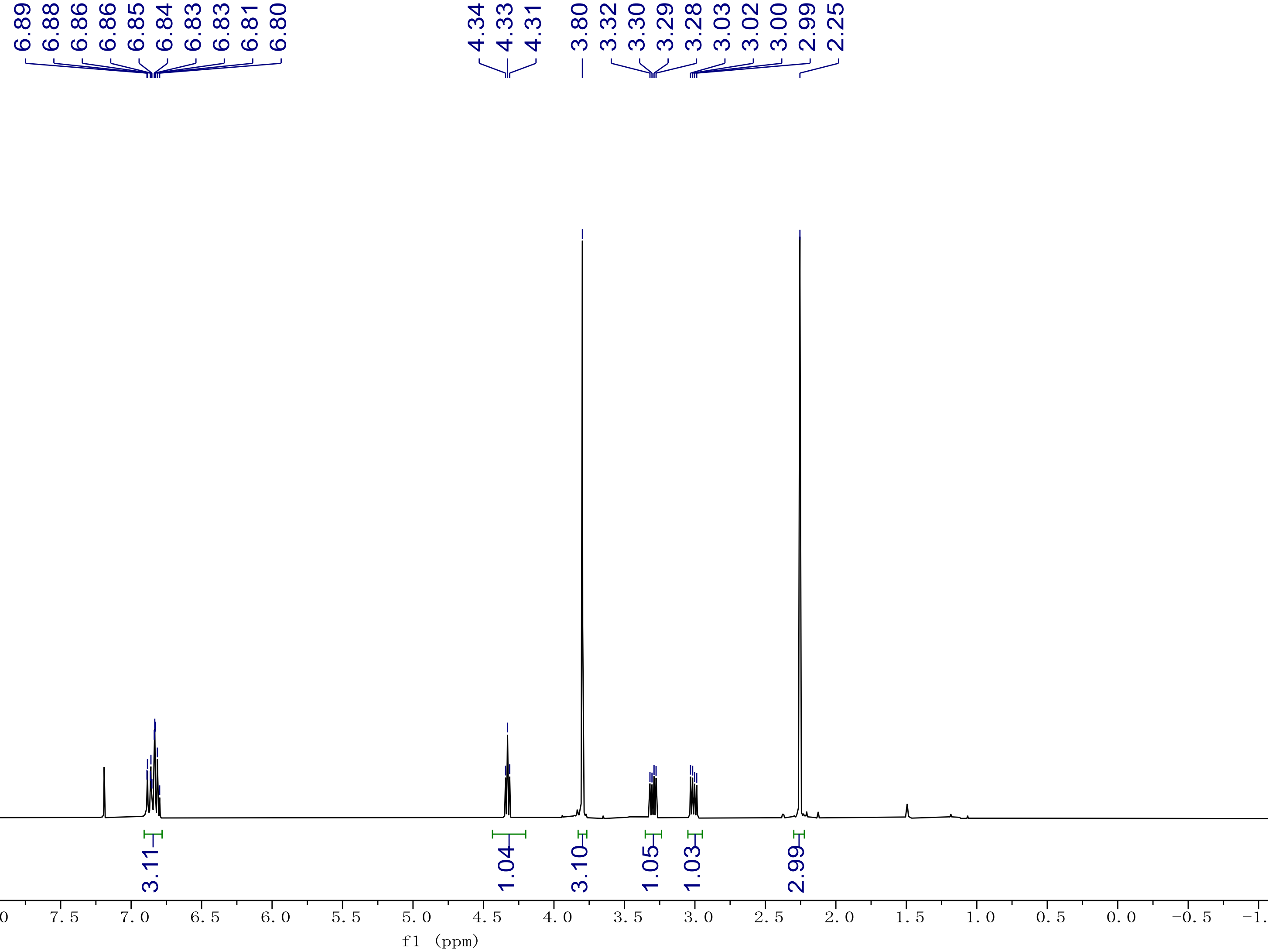

^{13}C NMR of (±)-3-bromo-arylbutan-2-one 30

$CDCl_3$, 23 ºC

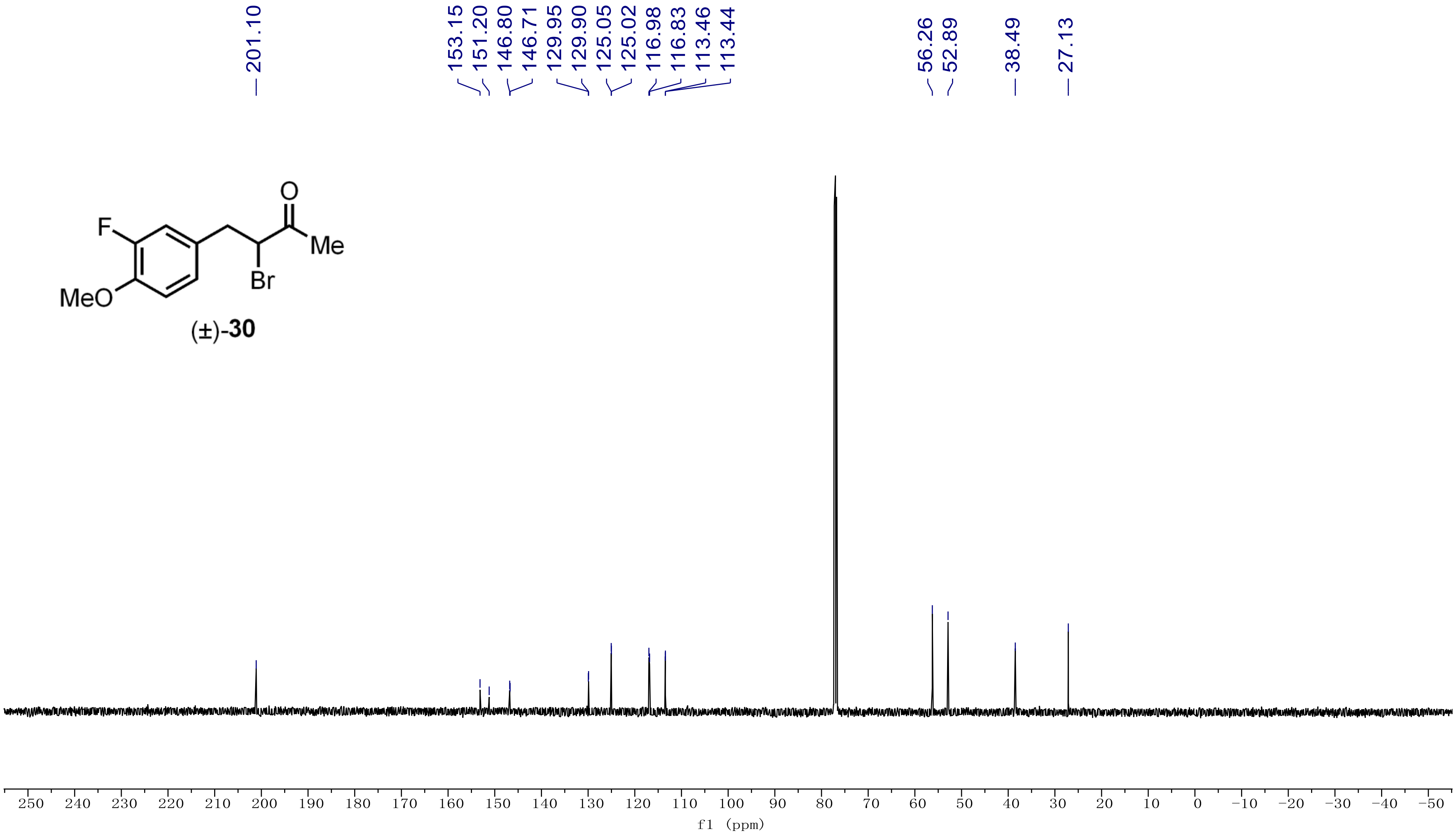

(±)-**30**

^{1}H NMR of (±)-2-bromo-(phenylsulfonyl)ethylarene 31

$CDCl_3$, 23 ºC

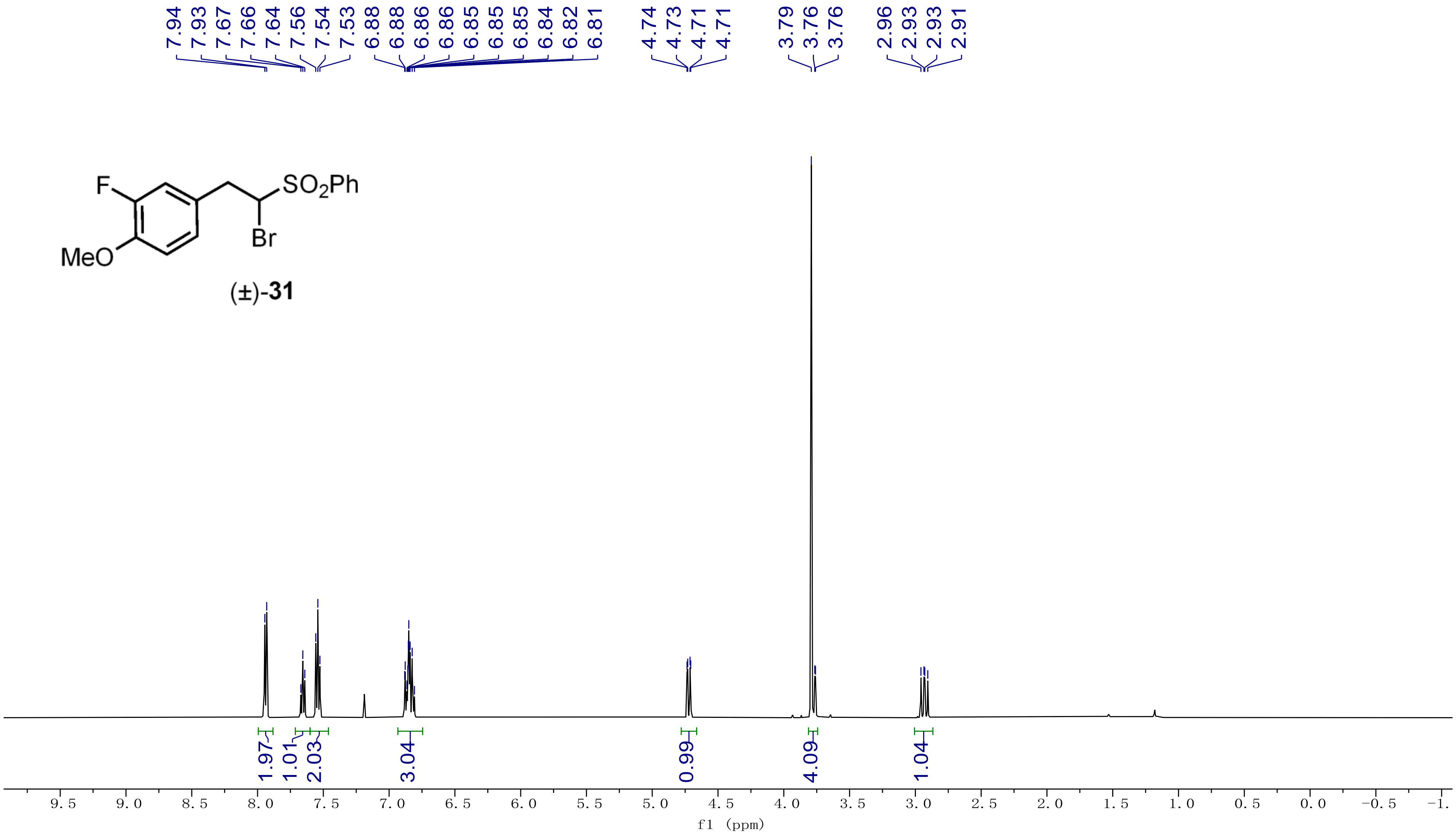

^{13}C NMR of (±)-2-bromo-(phenylsulfonyl)ethylarene 31

CDCl$_3$, 23 ºC

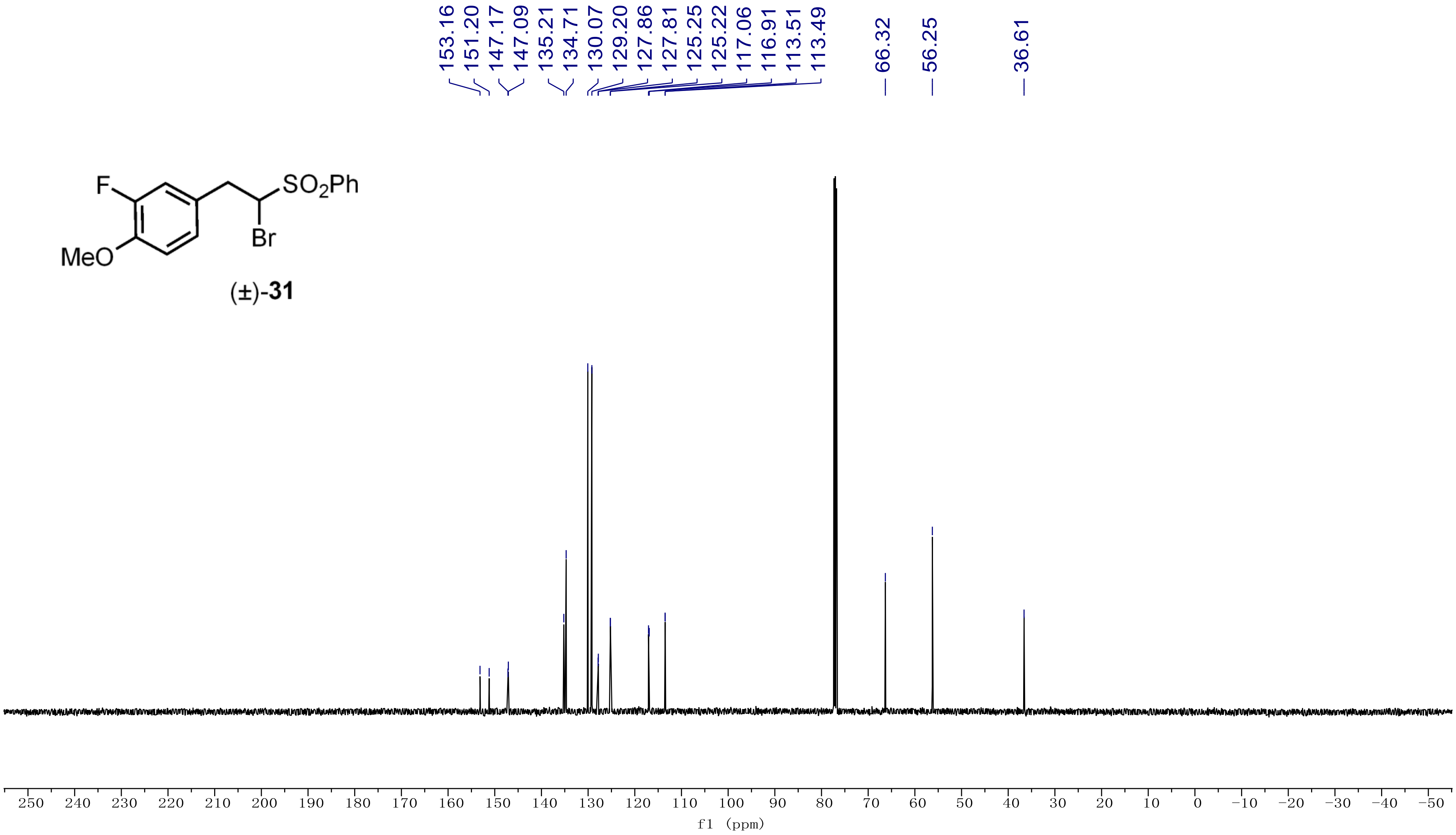

^{19}F NMR of (±)-2-bromo-(phenylsulfonyl)ethylarene 31

$CDCl_3$, 23 ºC

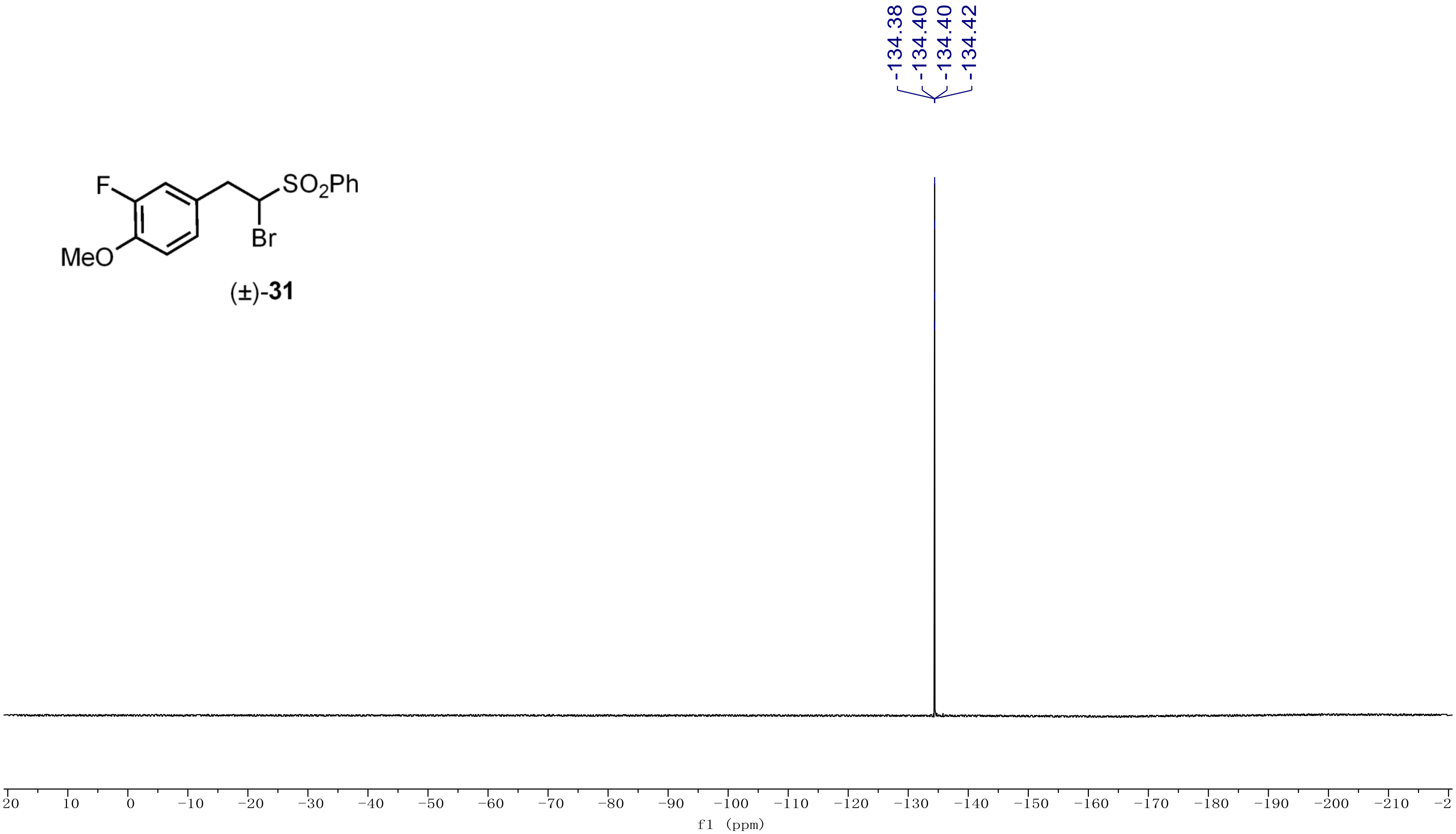

(±)-**31**

^{1}H NMR of (±)-1-bromo-arylethylphosphonate 32

$CDCl_3$, 23 ºC

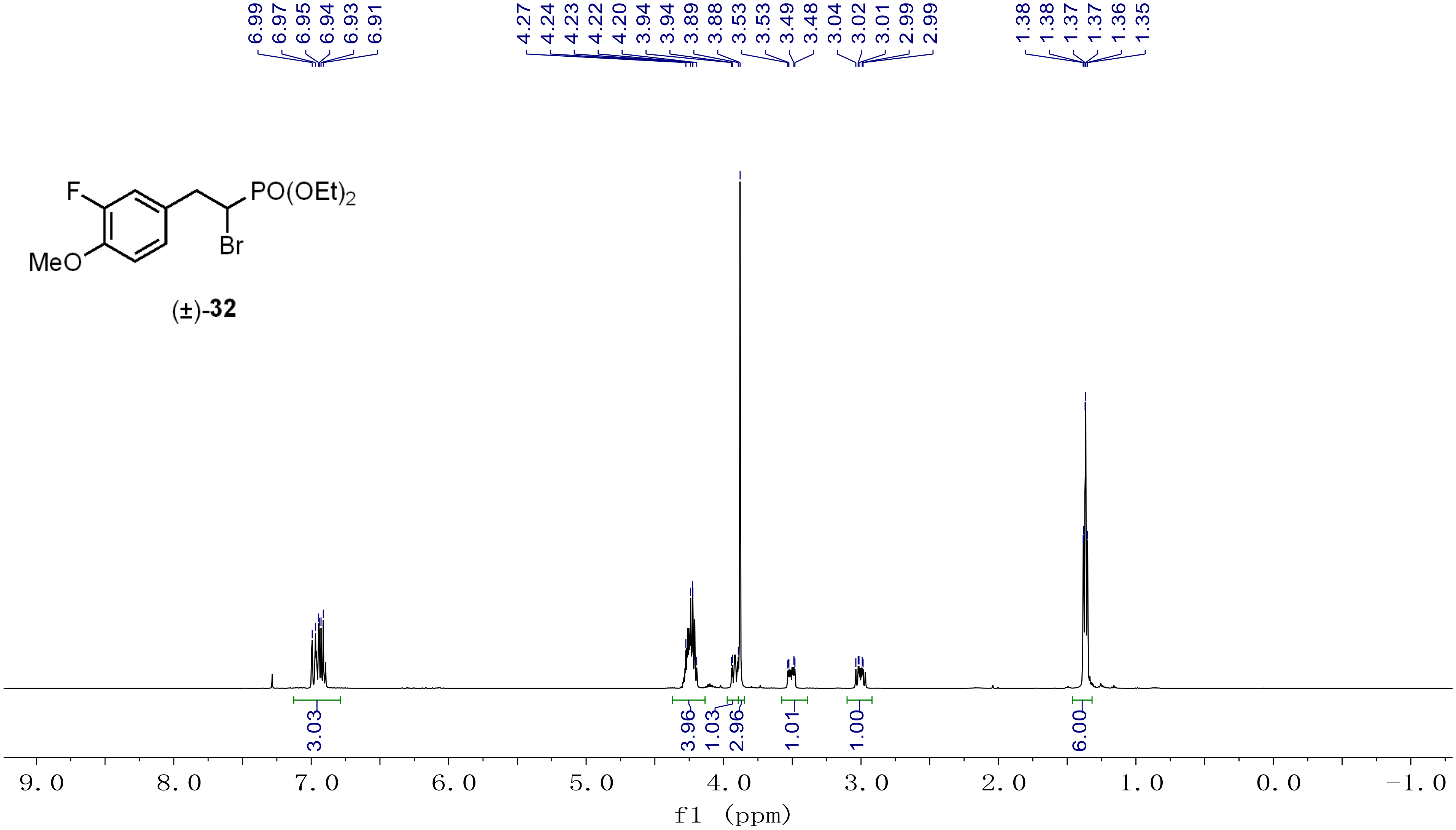

^{13}C NMR of (±)-1-bromo-arylethylphosphonate 32

$CDCl_3$, 23 ºC

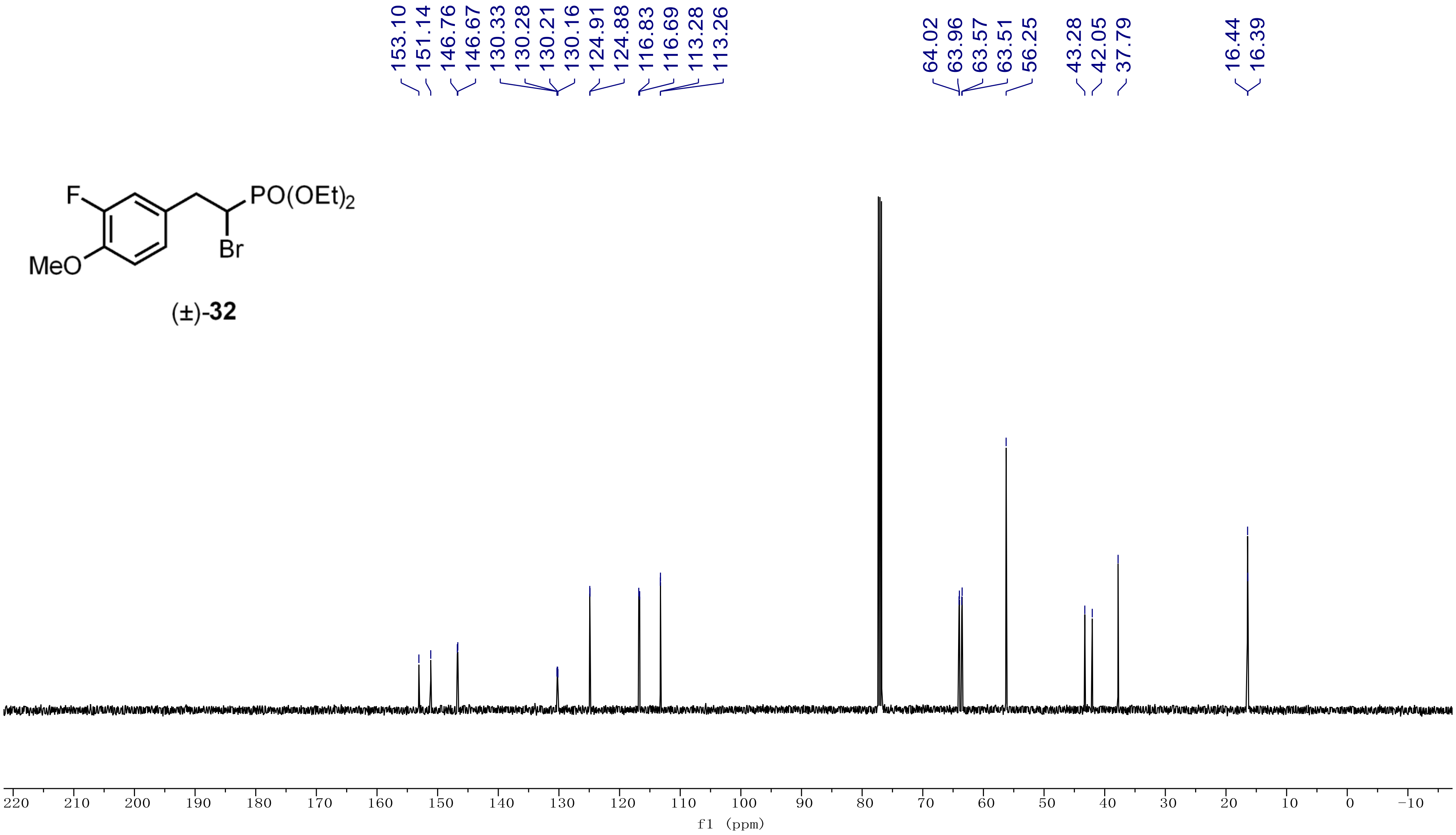

(±)-**32**

^{19}F NMR of (±)-1-bromo-arylethylphosphonate 32

$CDCl_3$, 23 ºC

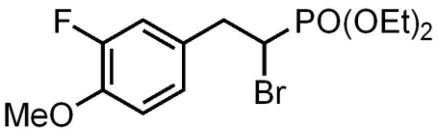

(±)-**32**

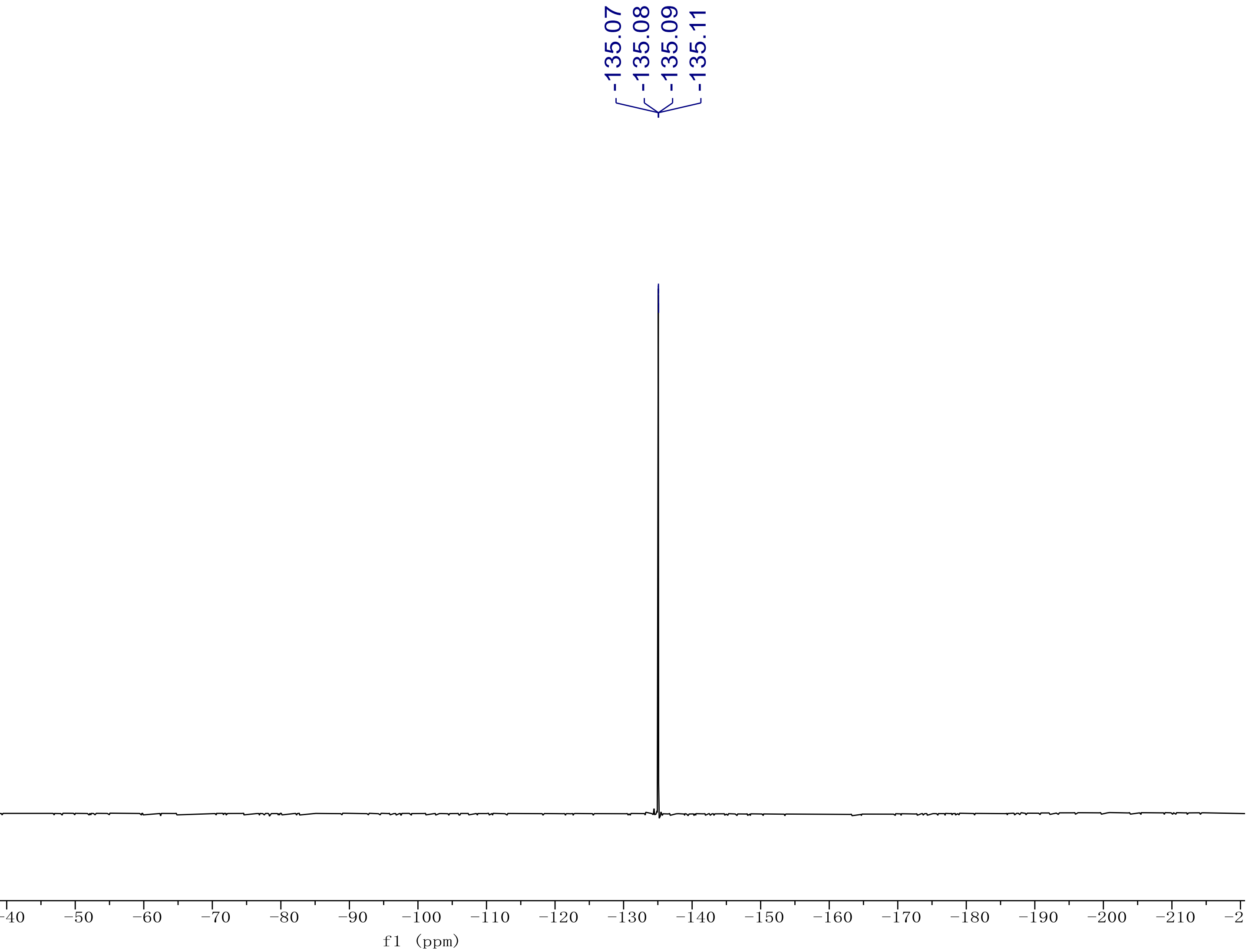

^{31}P NMR of (±)-1-bromo-arylethylphosphonate 32

$CDCl_3$, 23 ºC

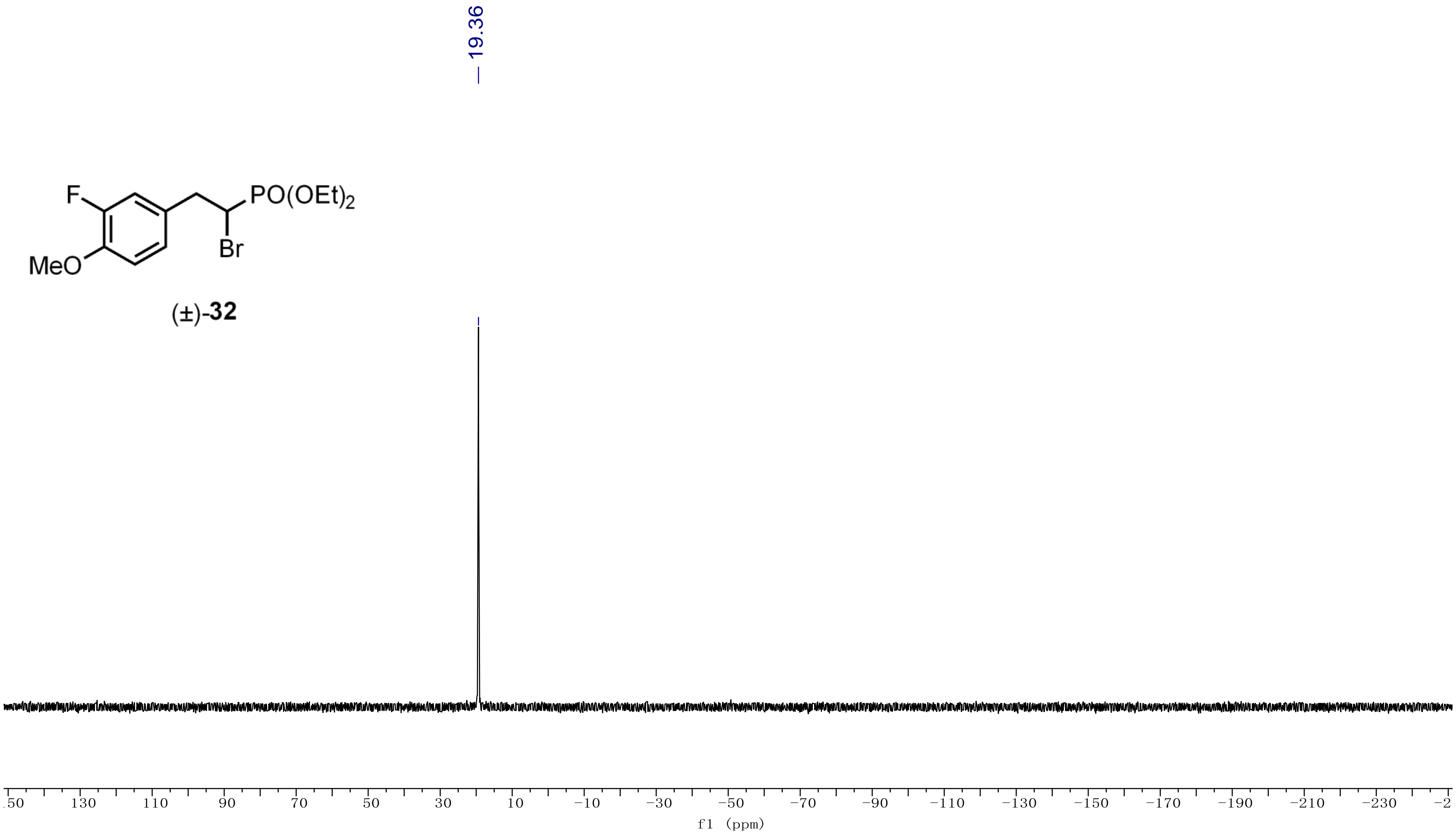

(±)-**32**

^{1}H NMR of (±)-butyrolactone 33

$CDCl_3$, 23 ºC

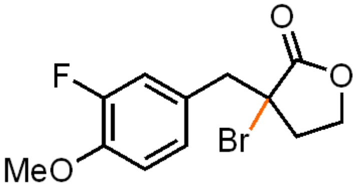

(±)-**33**

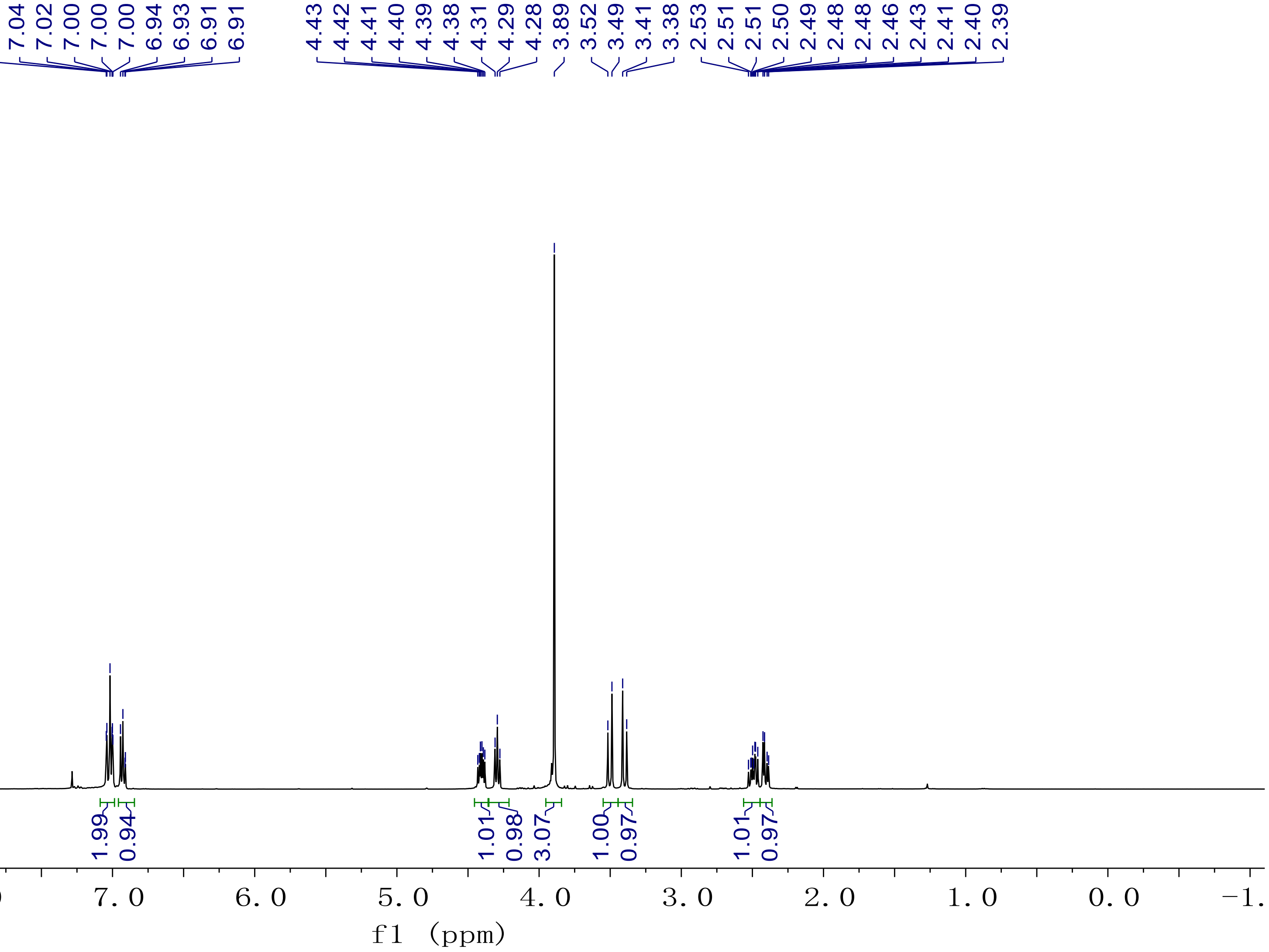

^{13}C NMR of (±)-butyrolactone 33

CDCl$_3$, 23 ºC

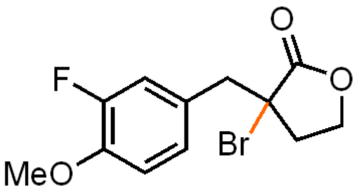

(±)-**33**

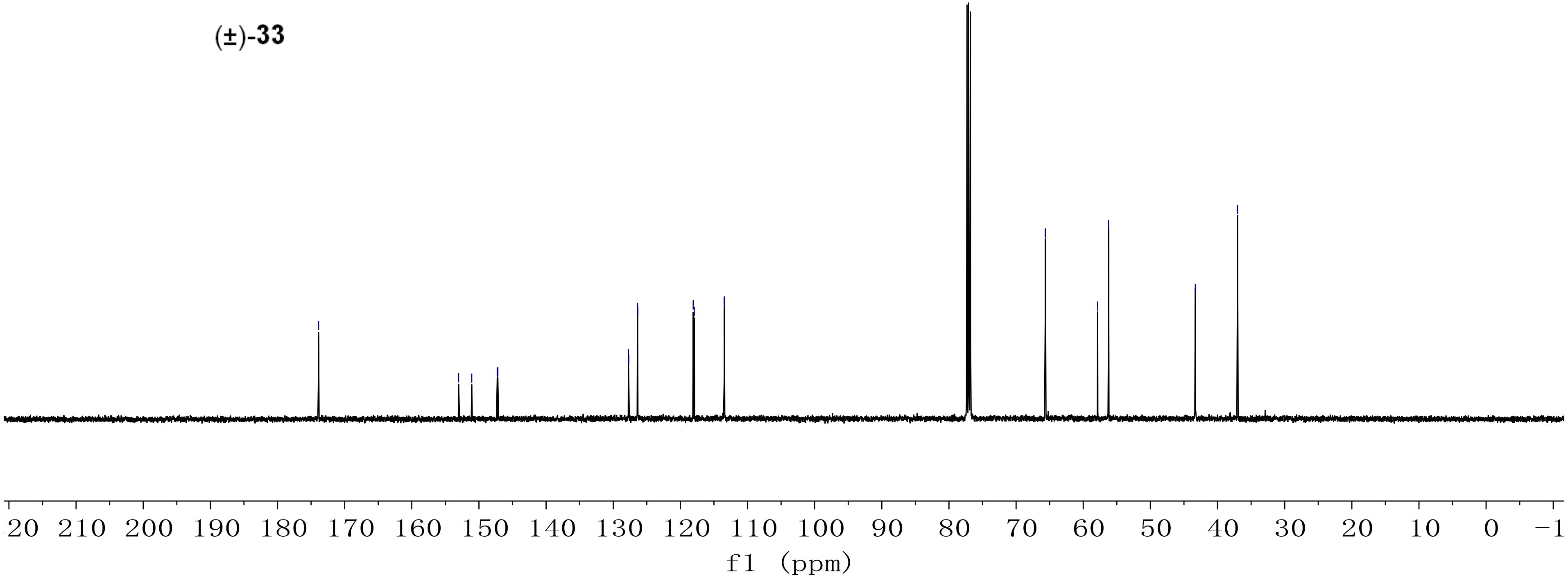

^{19}F NMR of (±)-butyrolactone 33

$CDCl_3$, 23 ºC

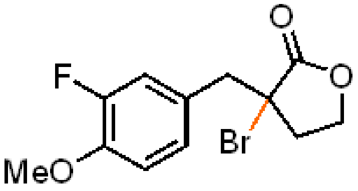

(±)-**33**

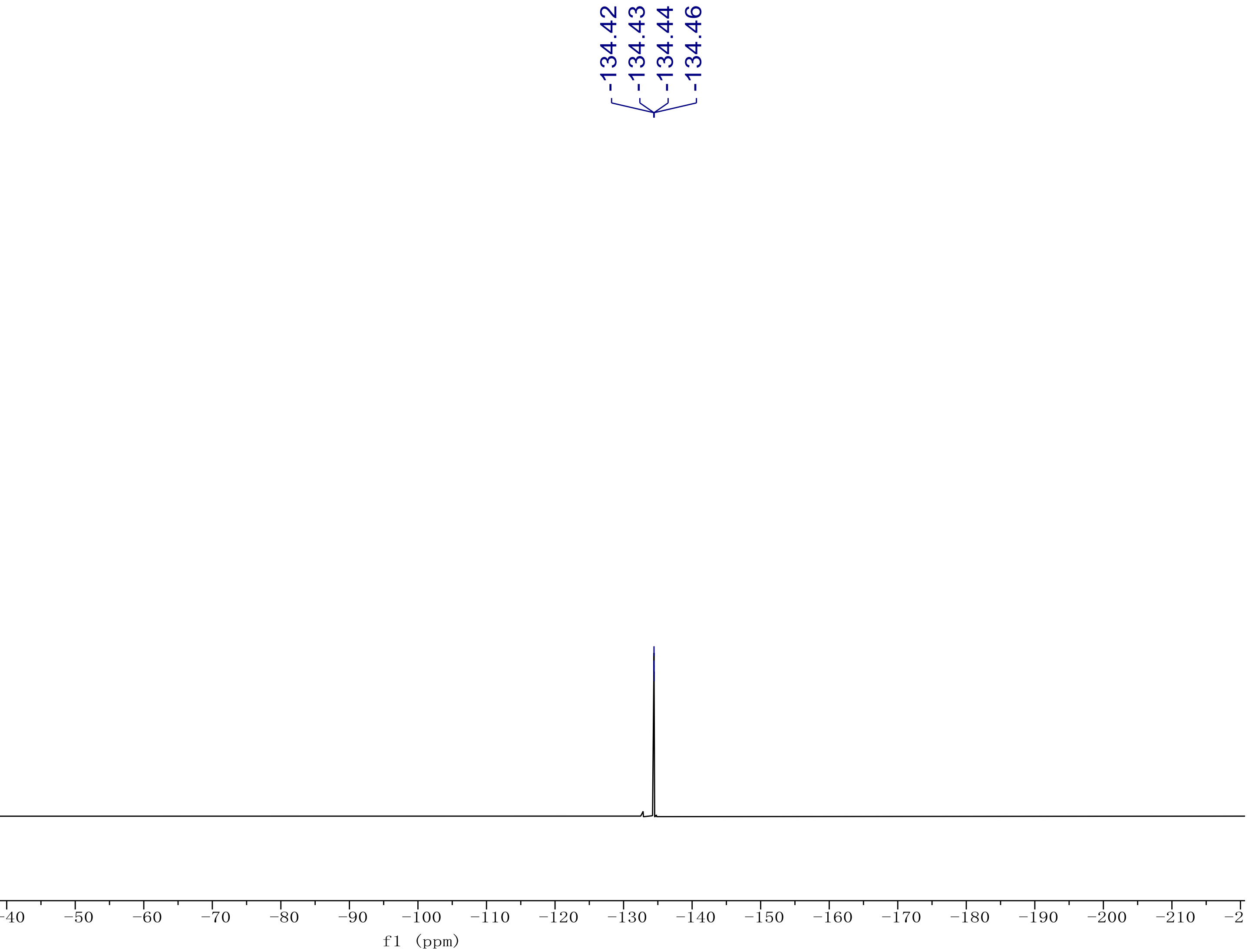

^{1}H NMR of (±)-2-bromo-2-fluoro-arylpropanoate 34

$CDCl_3$, 23 ºC

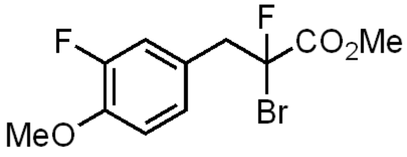

(±)-**34**

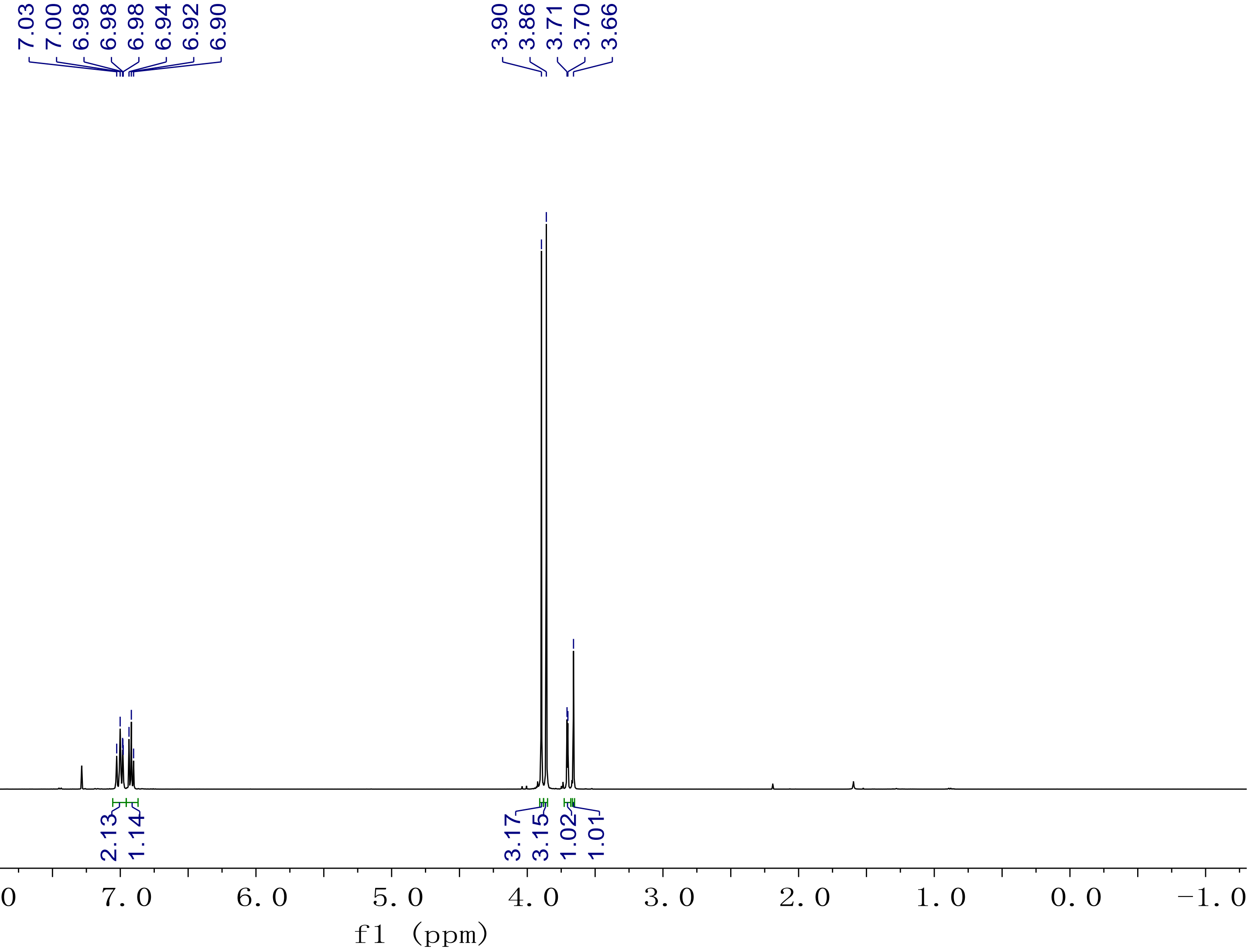

^{13}C NMR of (±)-2-bromo-2-fluoro-arylpropanoate 34

CDCl$_3$, 23 ºC

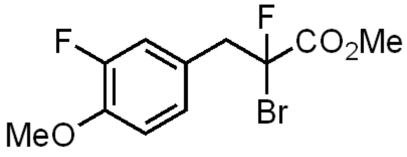
(±)-**34**

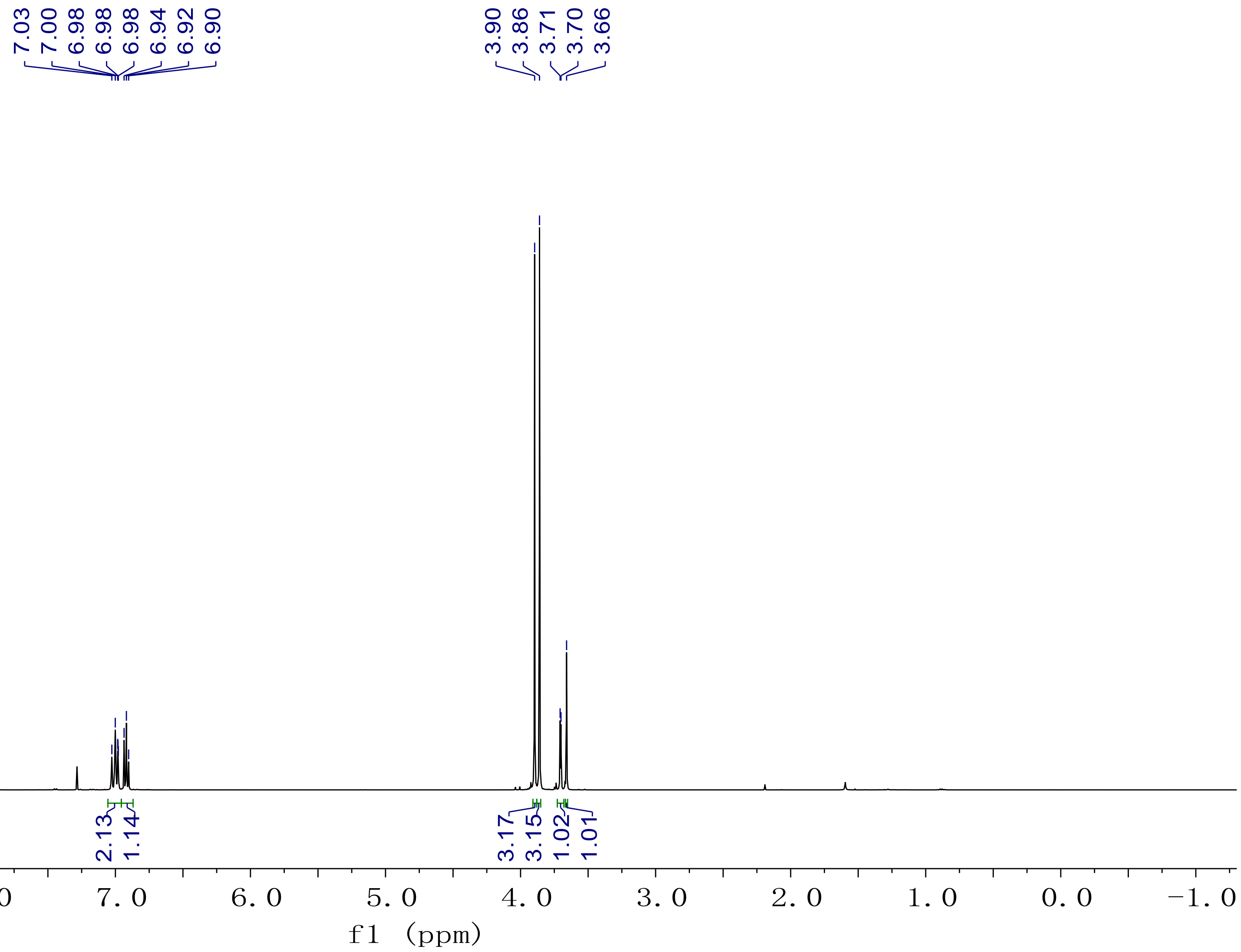

^{19}F NMR of (±)-2-bromo-2-fluoro-arylpropanoate 34

$CDCl_3$, 23 ºC

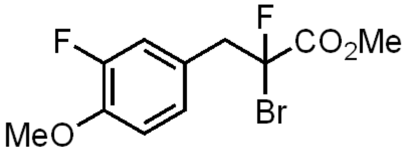

(±)-**34**

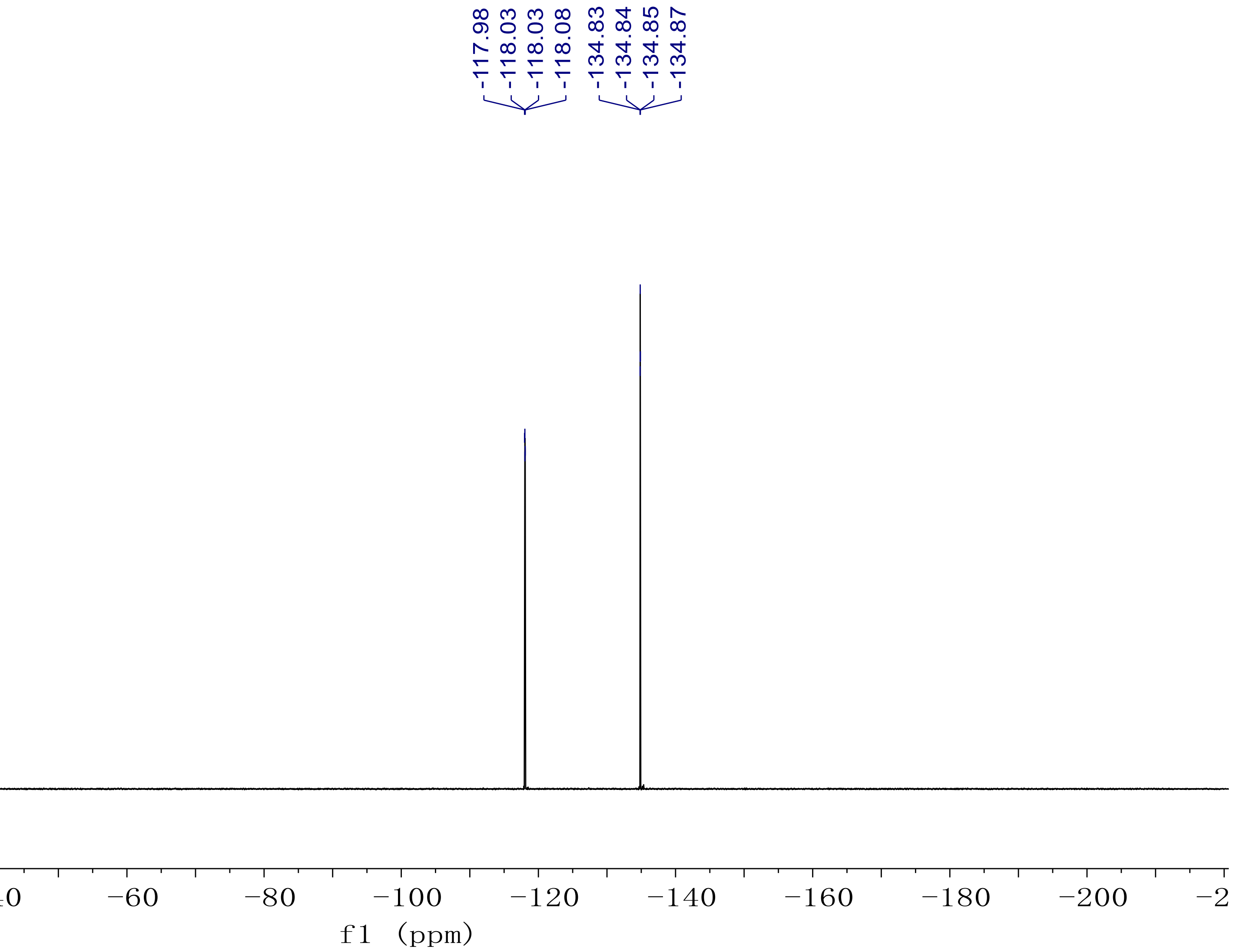

^{1}H NMR of (±)-2-bromo-arylpropanal 35

$CDCl_3$, 23 ºC

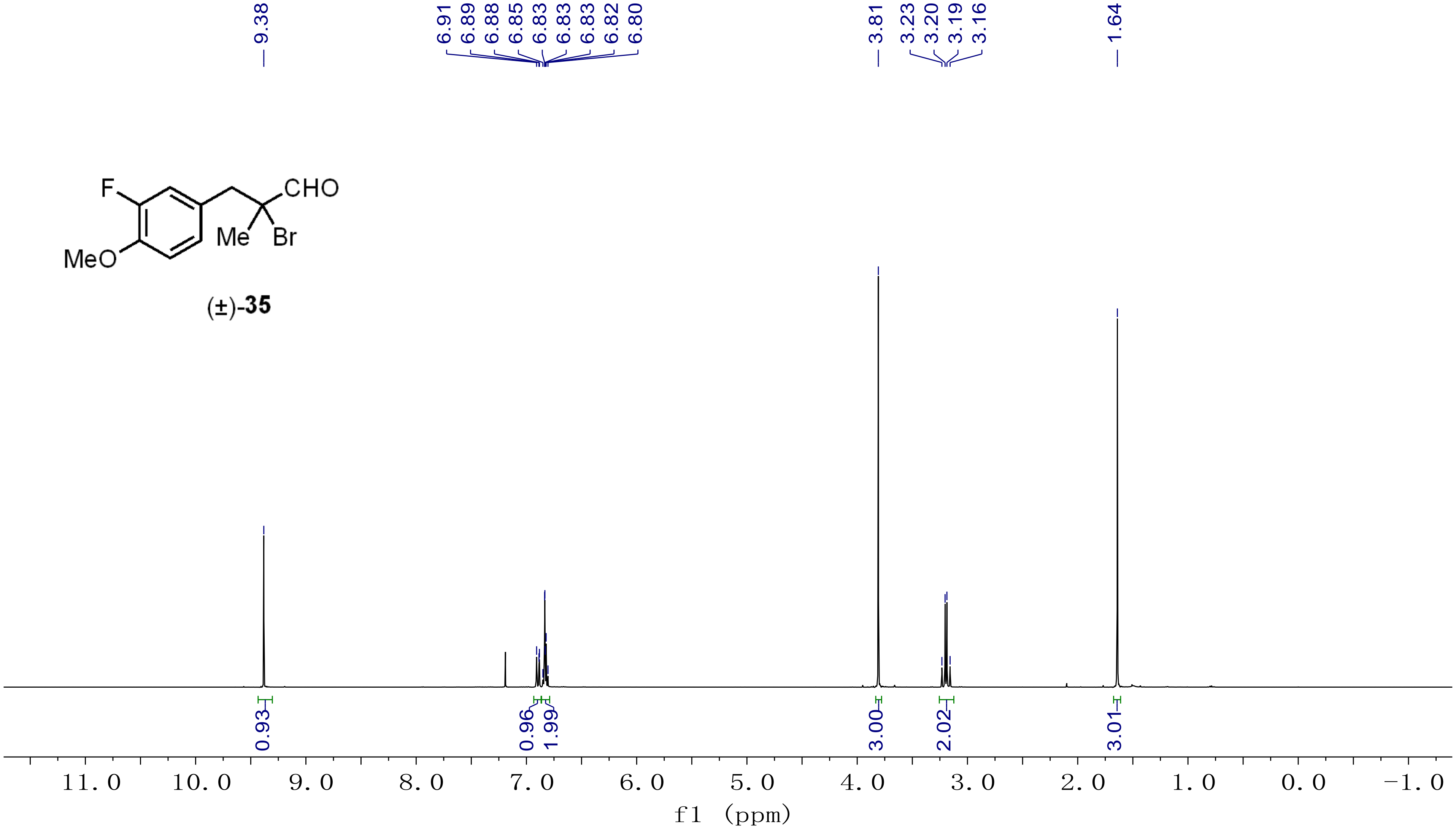

^{13}C NMR of (±)-2-bromo-arylpropanal 35

$CDCl_3$, 23 ºC

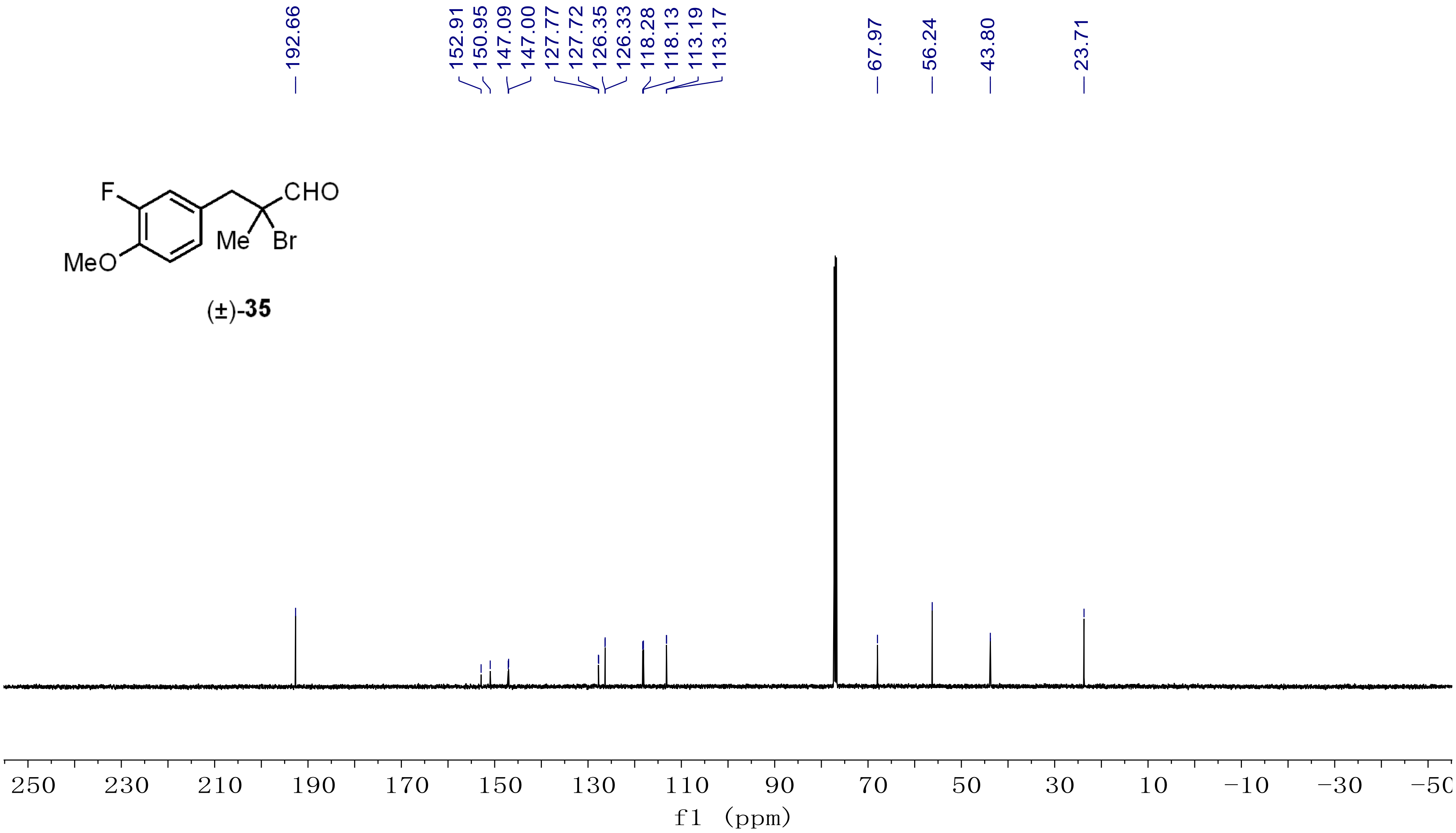

^{19}F NMR of (±)-2-bromo-arylpropanal 35

$CDCl_3$, 23 ºC

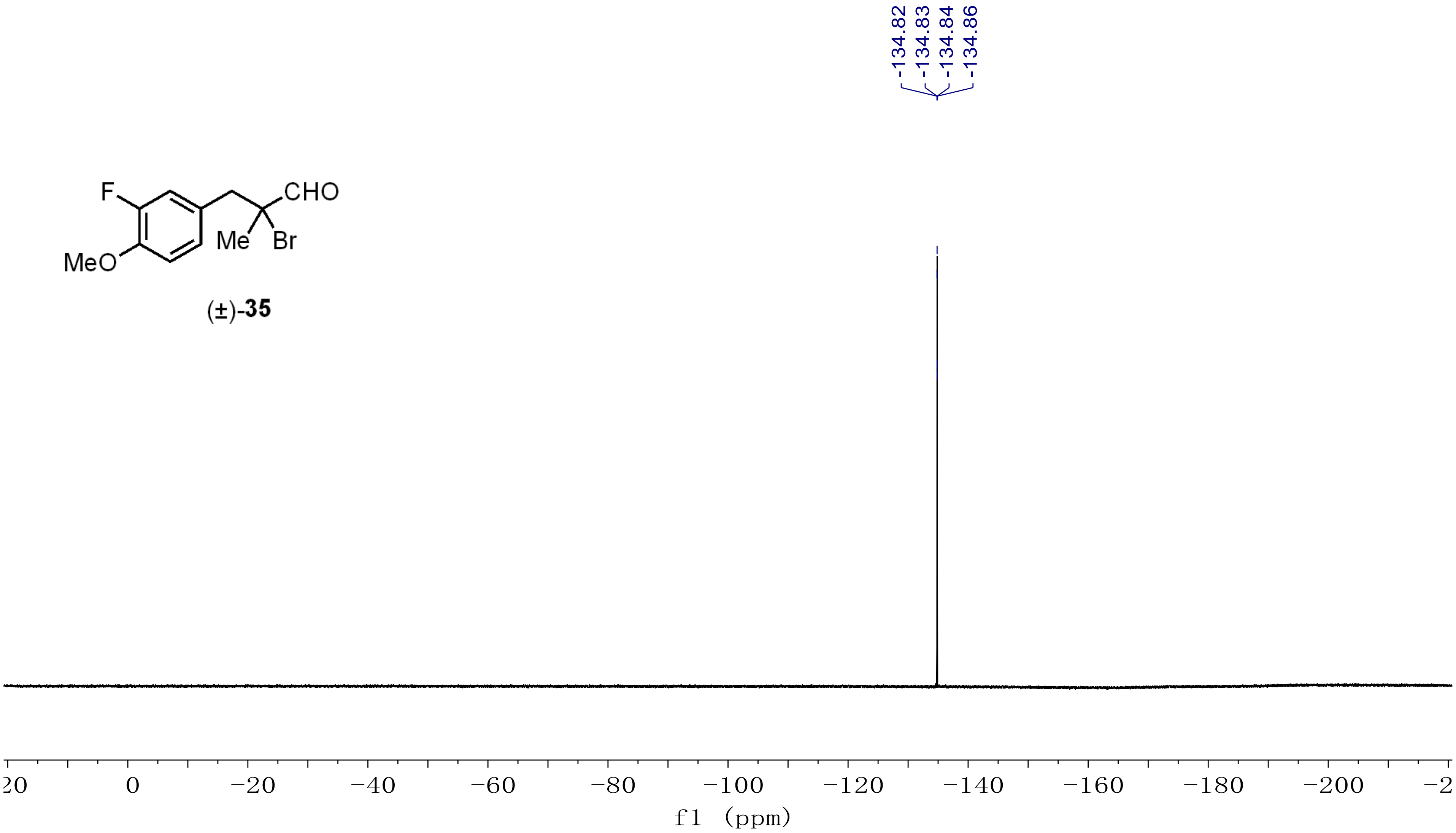

(±)-**35**

^{1}H NMR of (±)-2-bromo-arylpropanamide 36

$CDCl_3$, 23 ºC

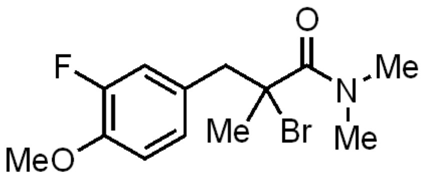

(±)-**36**

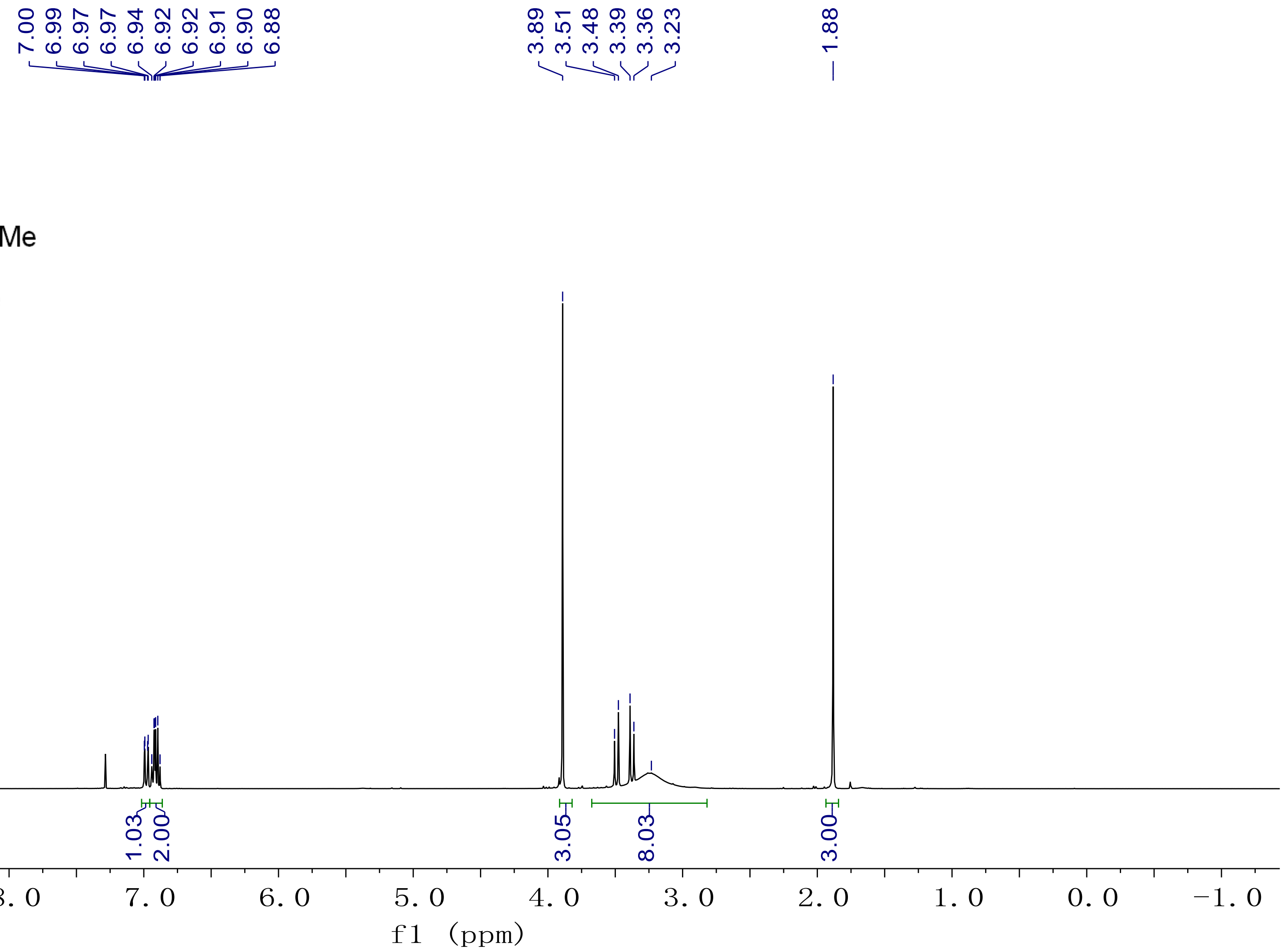

^{13}C NMR of (±)-2-bromo-arylpropanamide 36

CDCl$_3$, 23 ºC

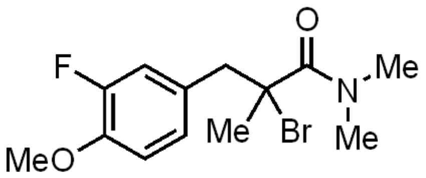

(±)-**36**

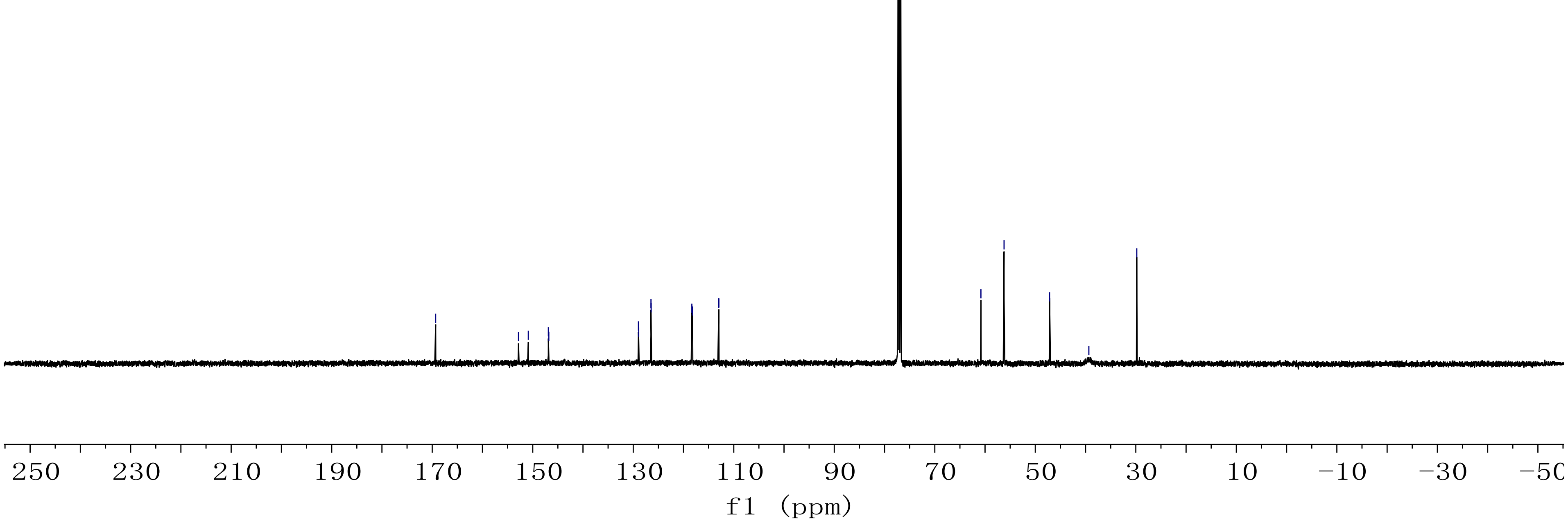

^{19}F NMR of (±)-2-bromo-arylpropanamide 36

$CDCl_3$, 23 ºC

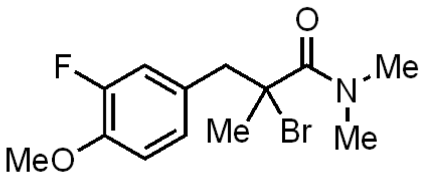

(±)-**36**

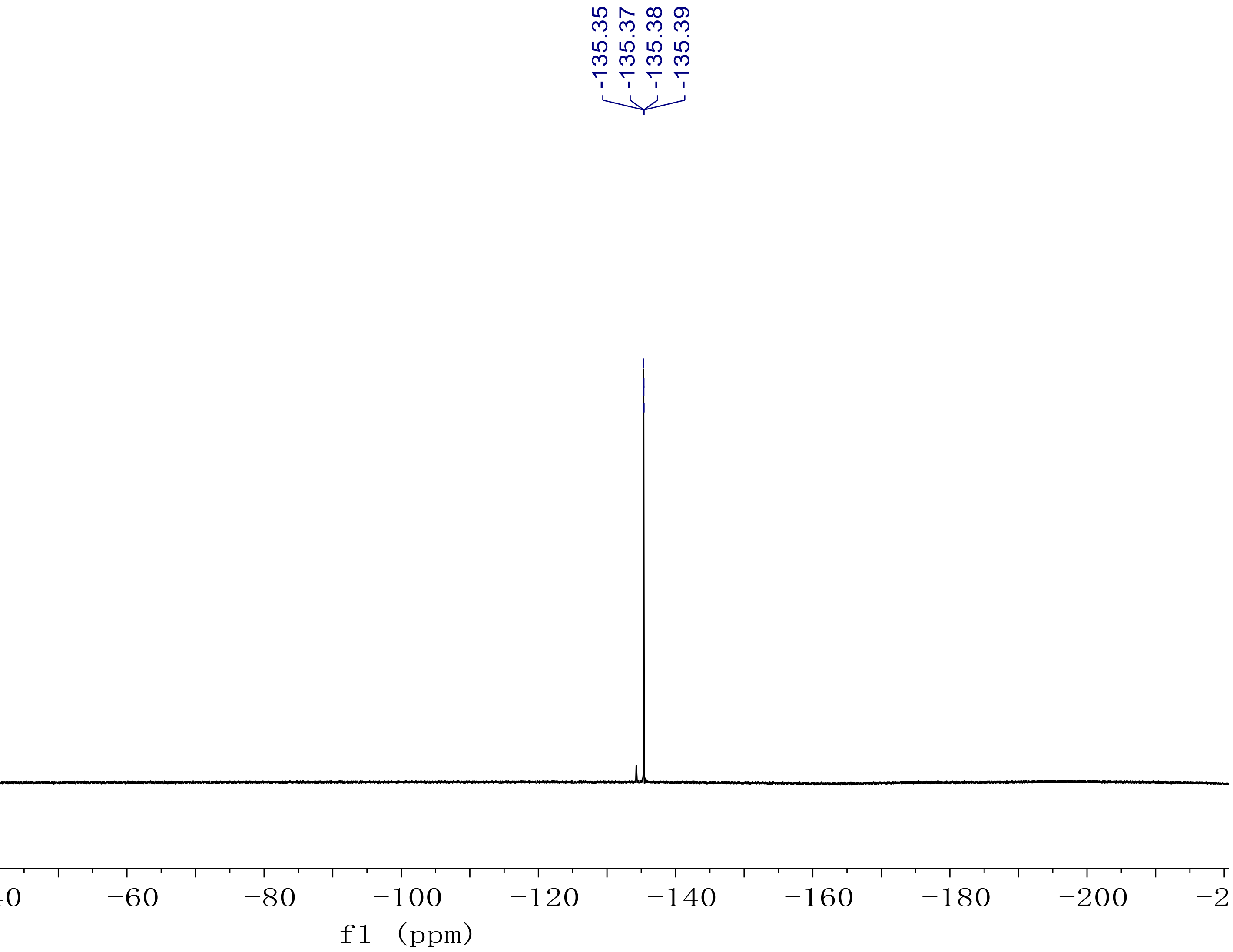

^{1}H NMR of (±)-2-bromo-arylpropanamide 37

$CDCl_3$, 23 ºC

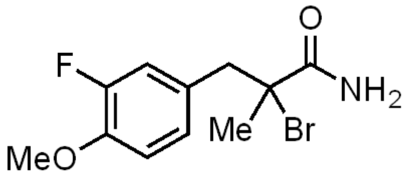

(±)-**37**

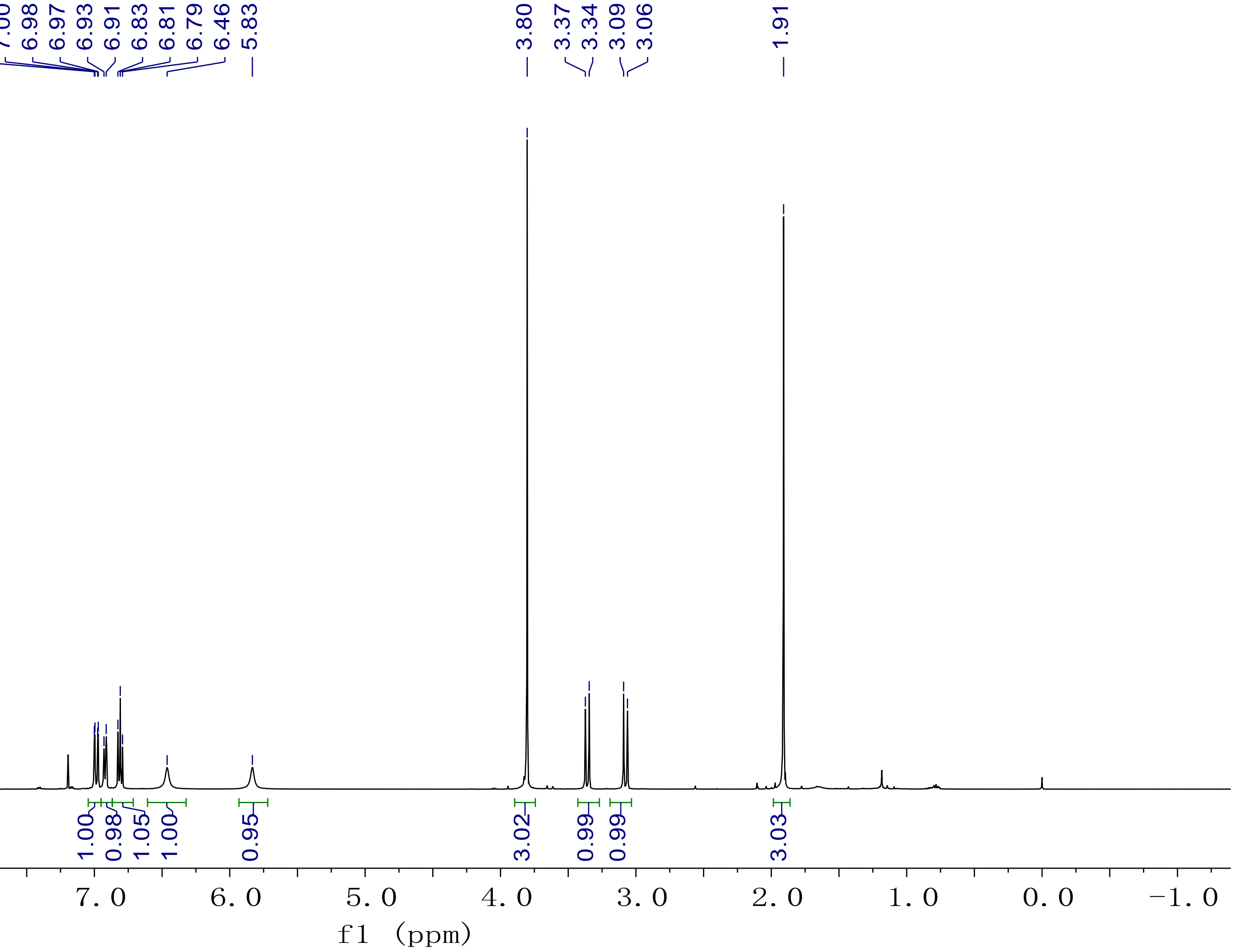

^{13}C NMR of (±)-2-bromo-arylpropanamide 37

$CDCl_3$, 23 ºC

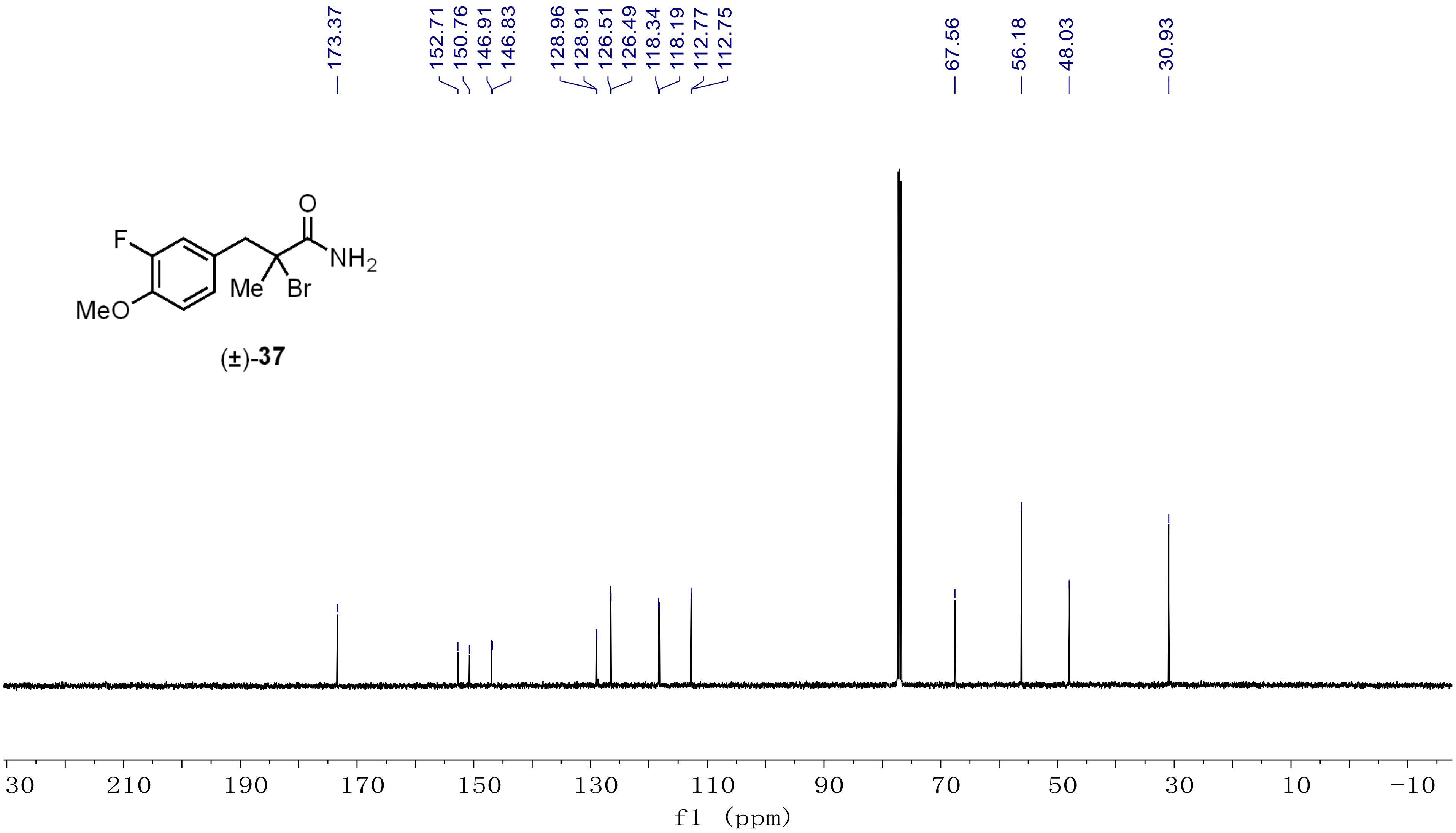

^{19}F NMR of (±)-2-bromo-arylpropanamide 37

$CDCl_3$, 23 ºC

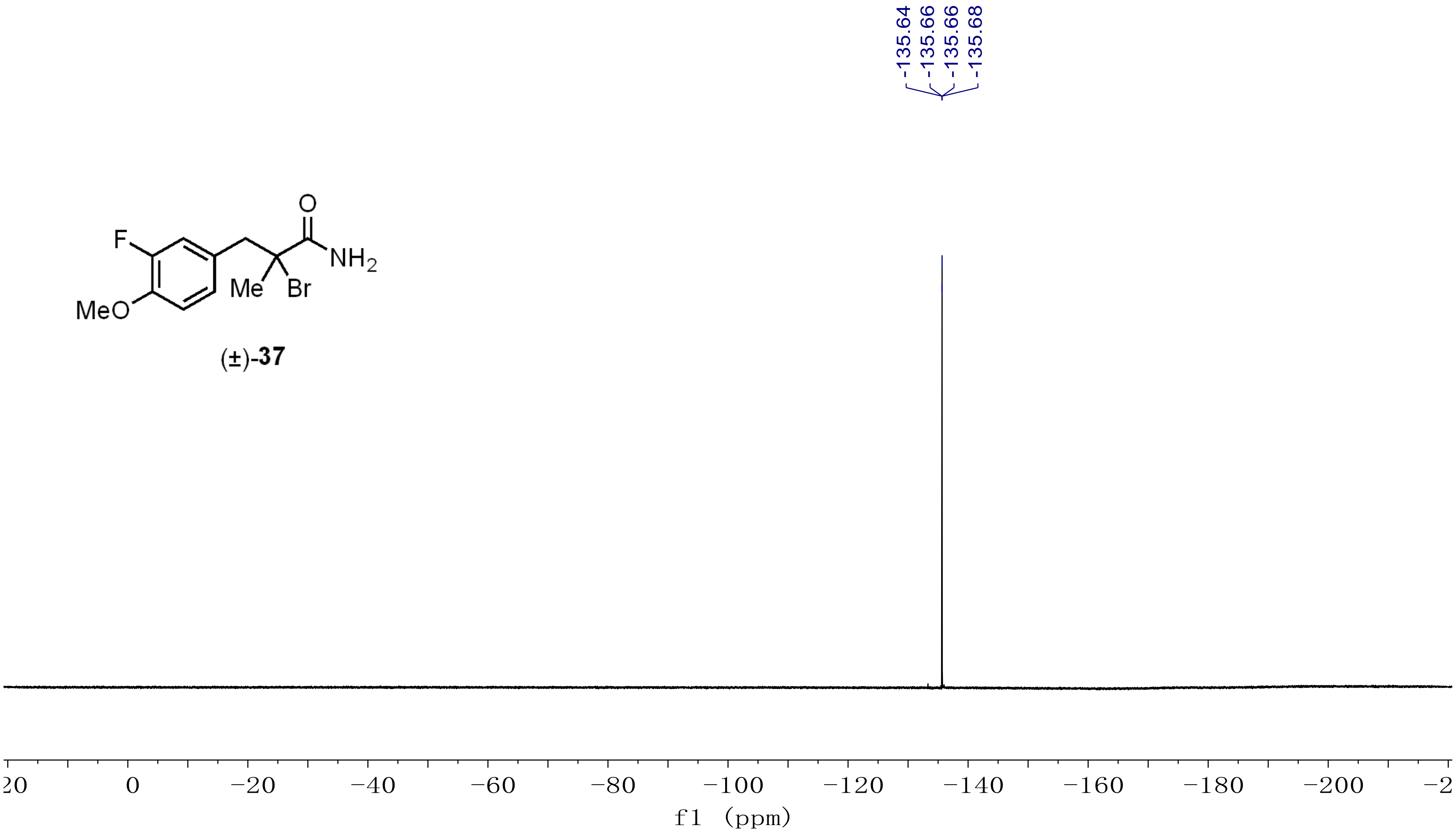

(±)-**37**

^{1}H NMR of (±)-2-bromo-2-phenylethylarene 38

$CDCl_3$, 23 ºC

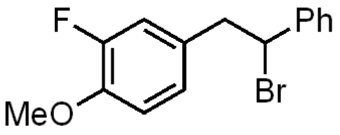

(±)-**38**

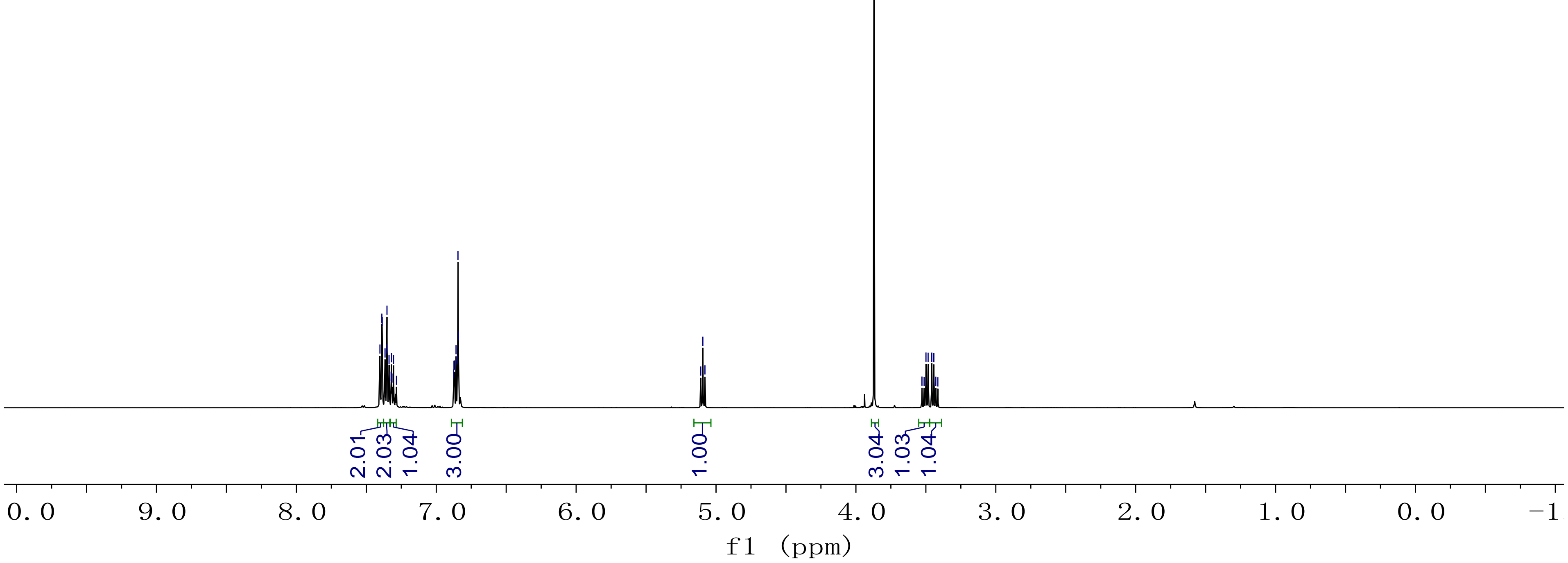

^{13}C NMR of (±)-2-bromo-2-phenylethylarene 38

CDCl$_3$, 23 ºC

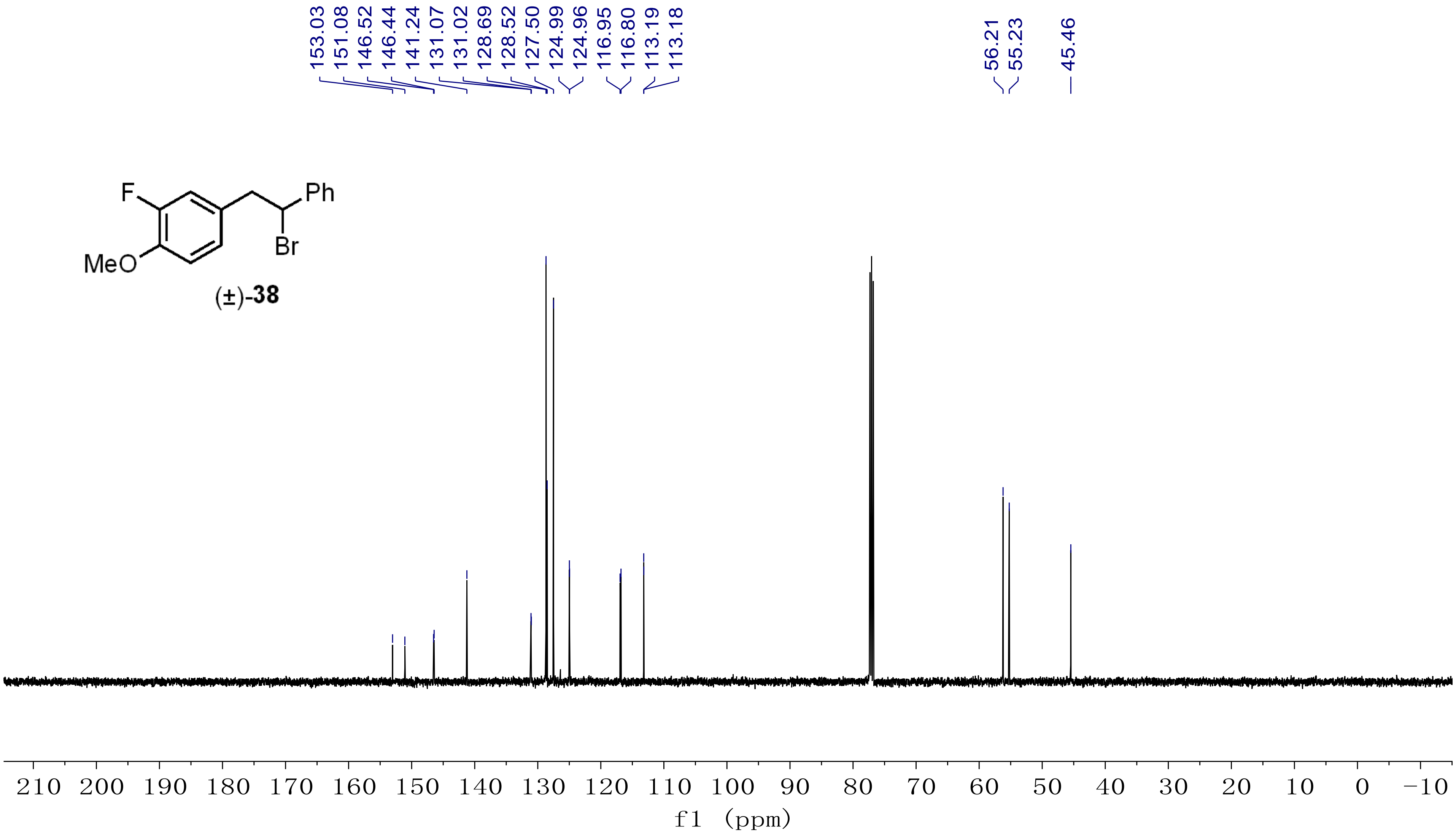

^{1}H NMR of (±)-2-bromo-2-phenylethylarene 39

$CDCl_3$, 23 ºC

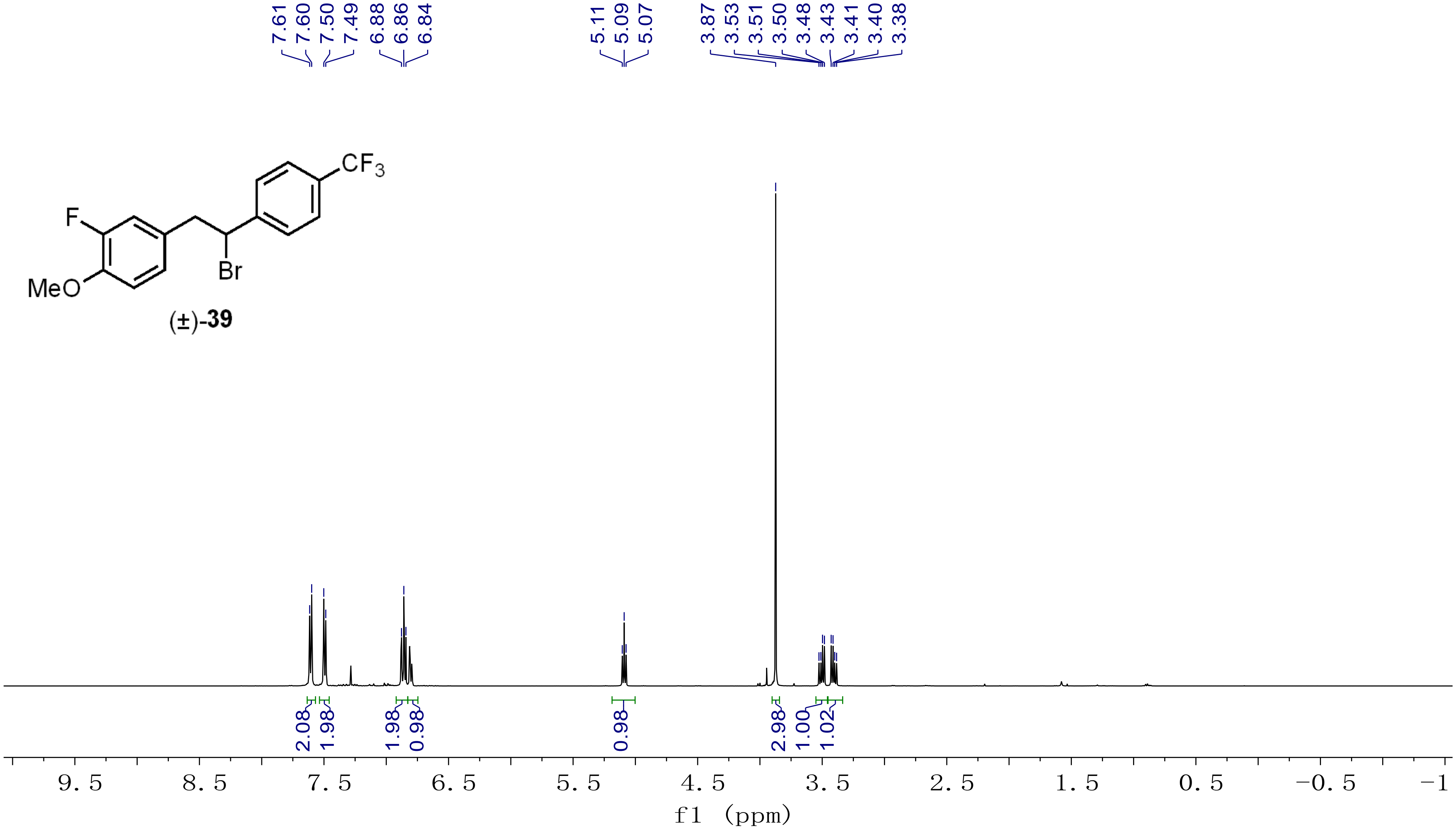

^{13}C NMR of (±)-2-bromo-2-phenylethylarene 39

CDCl3, 23 ºC

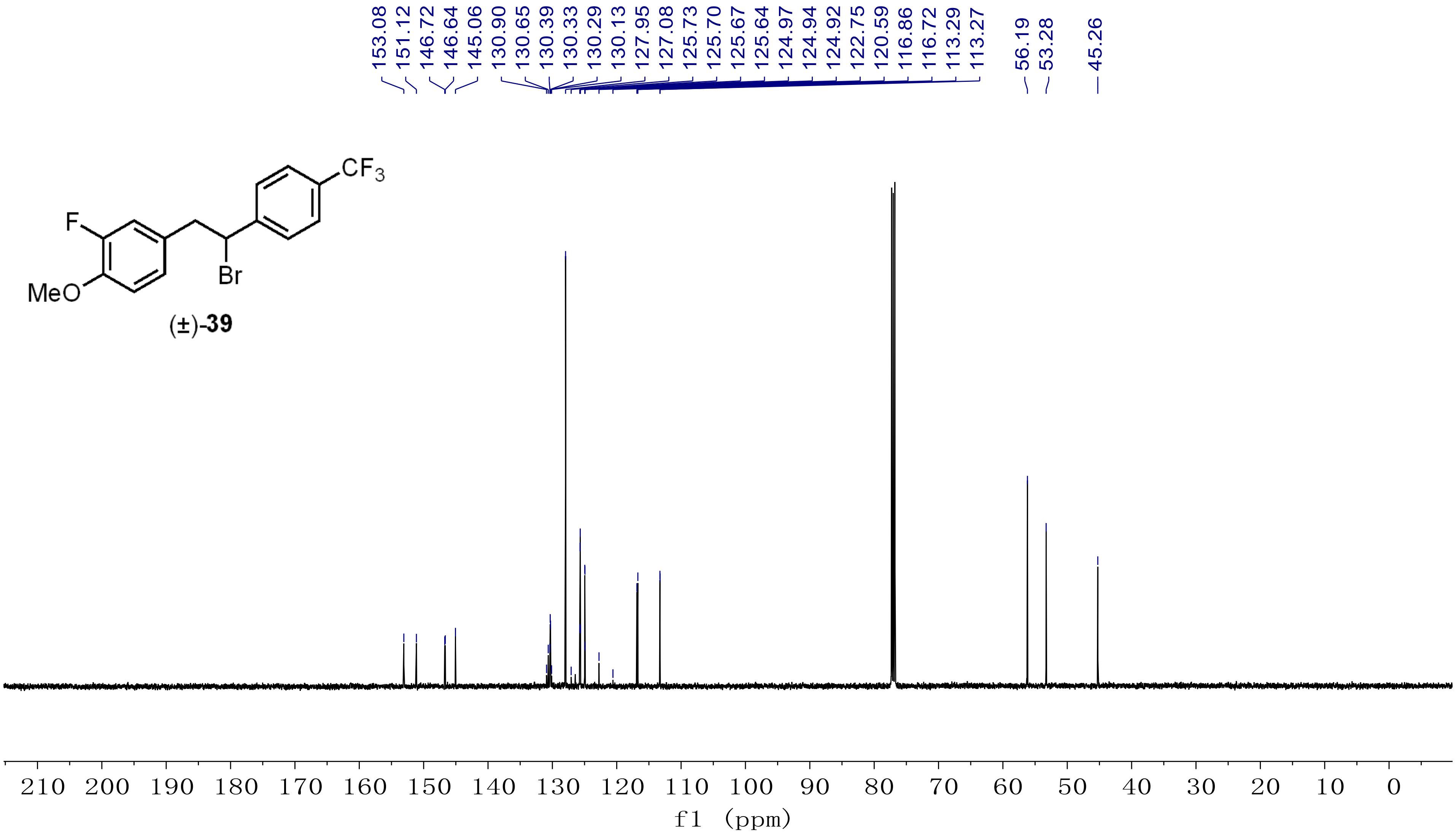

(±)-**39**

^{19}F NMR of (±)-2-bromo-2-phenylethylarene 39

$CDCl_3$, 23 ºC

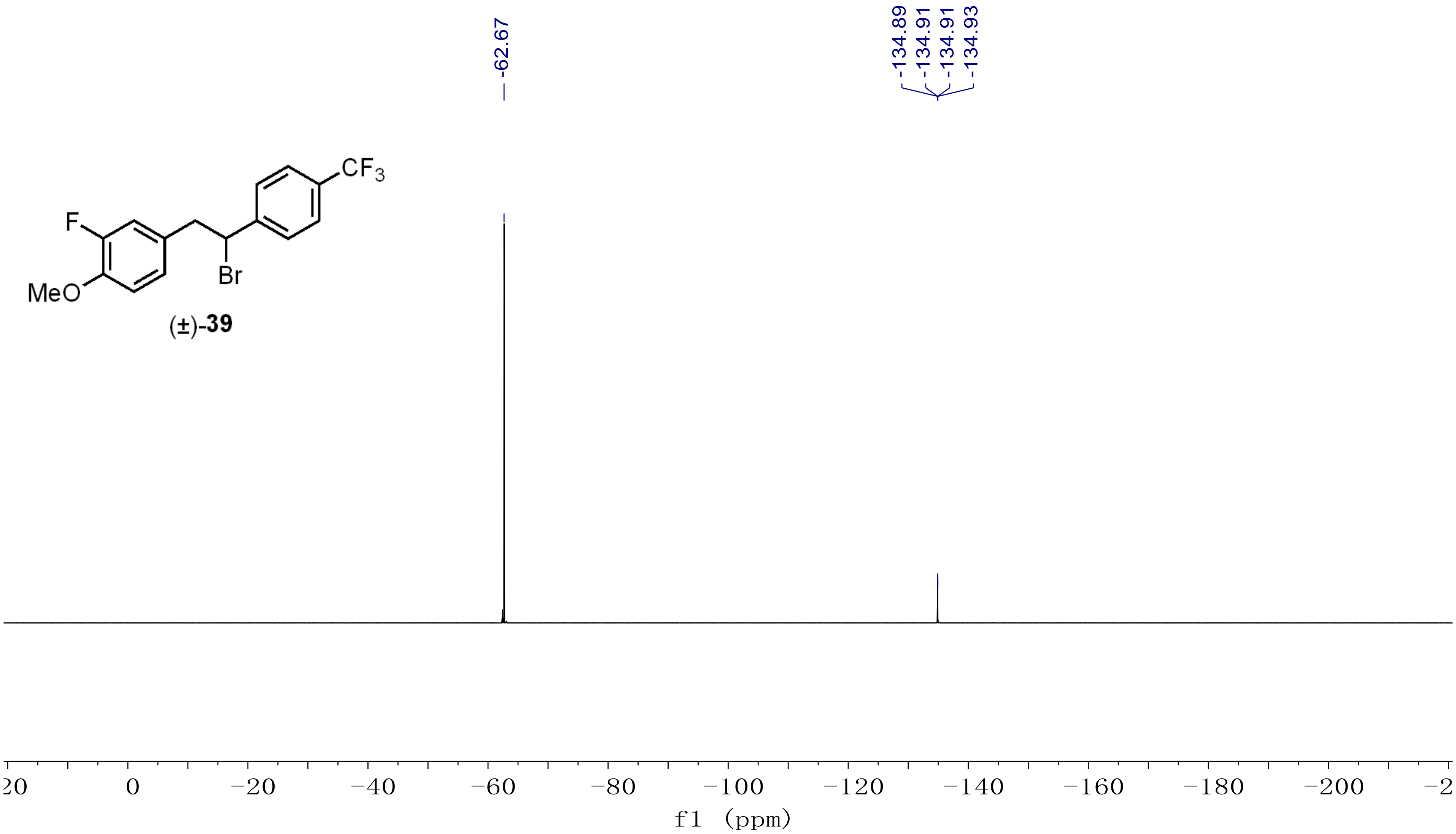

^{1}H NMR of (±)-2,4-dibromobutylarene 40

CDCl3, 23 ºC

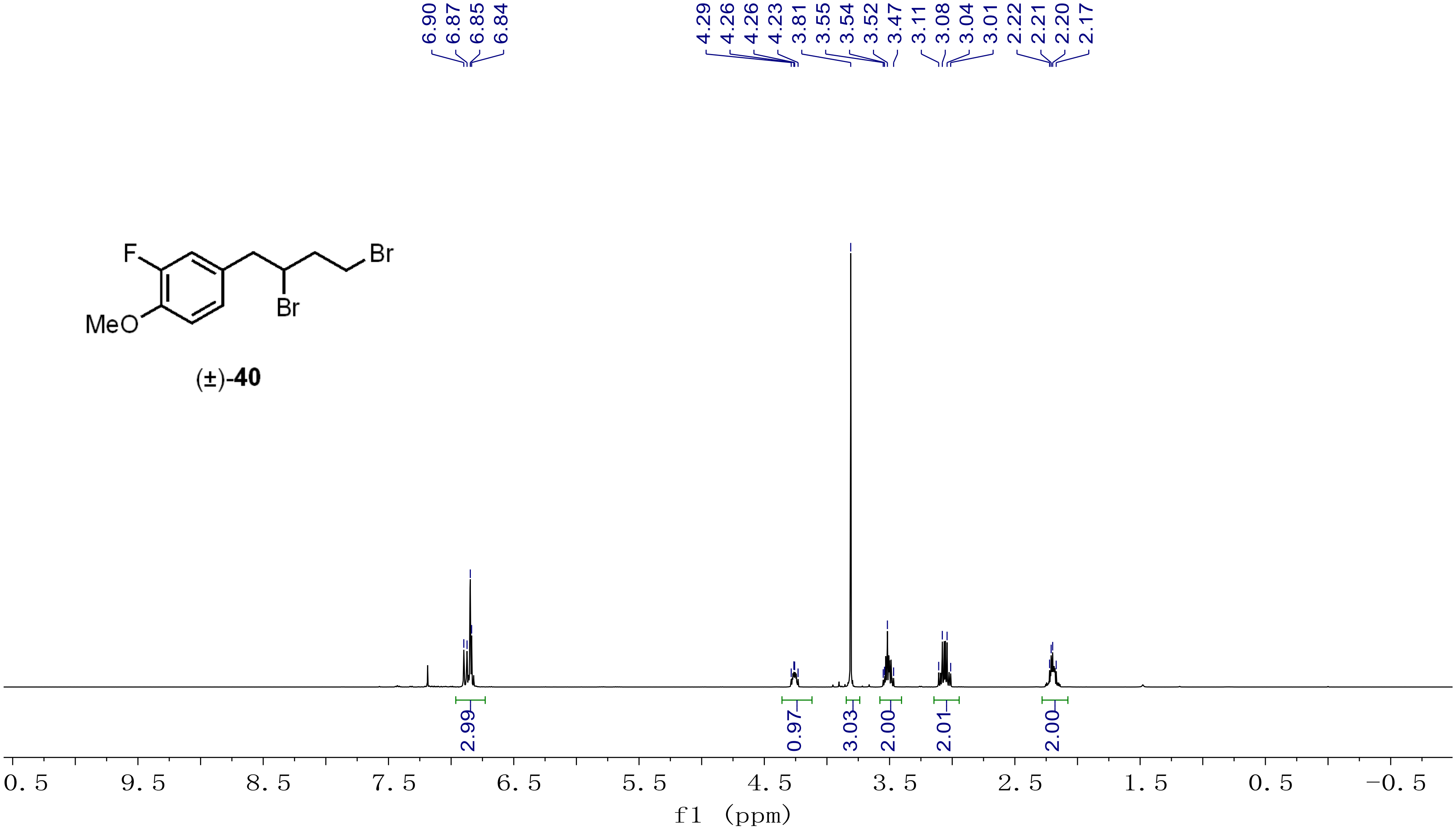

^{13}C NMR of (±)-2,4-dibromobutylarene 40

CDCl$_3$, 23 °C

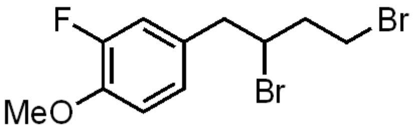

(±)-**40**

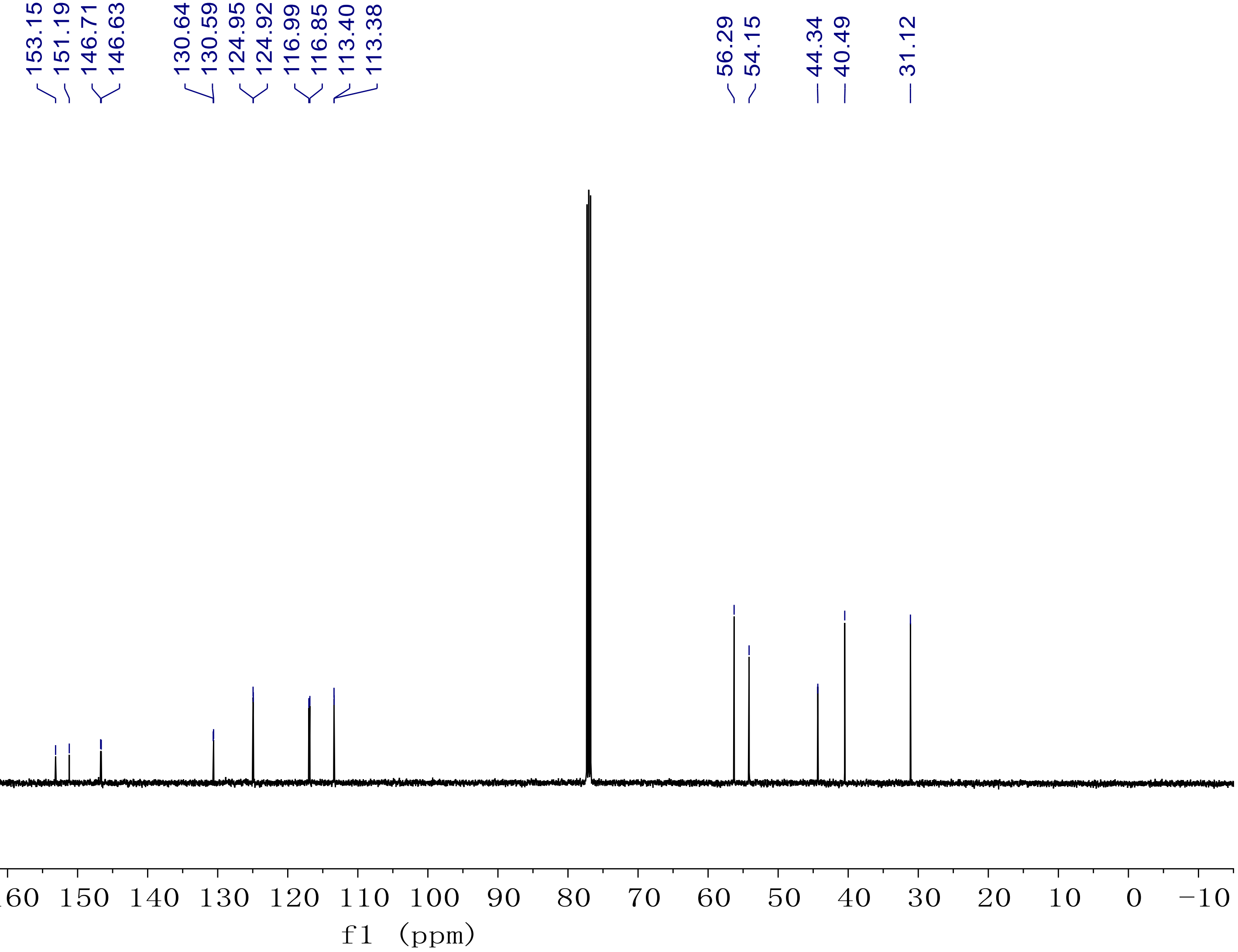

^{19}F NMR of (±)-2,4-dibromobutylarene 40

$CDCl_3$, 23 ºC

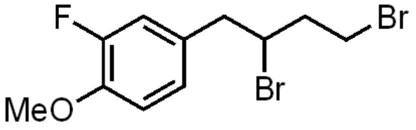

(±)-**40**

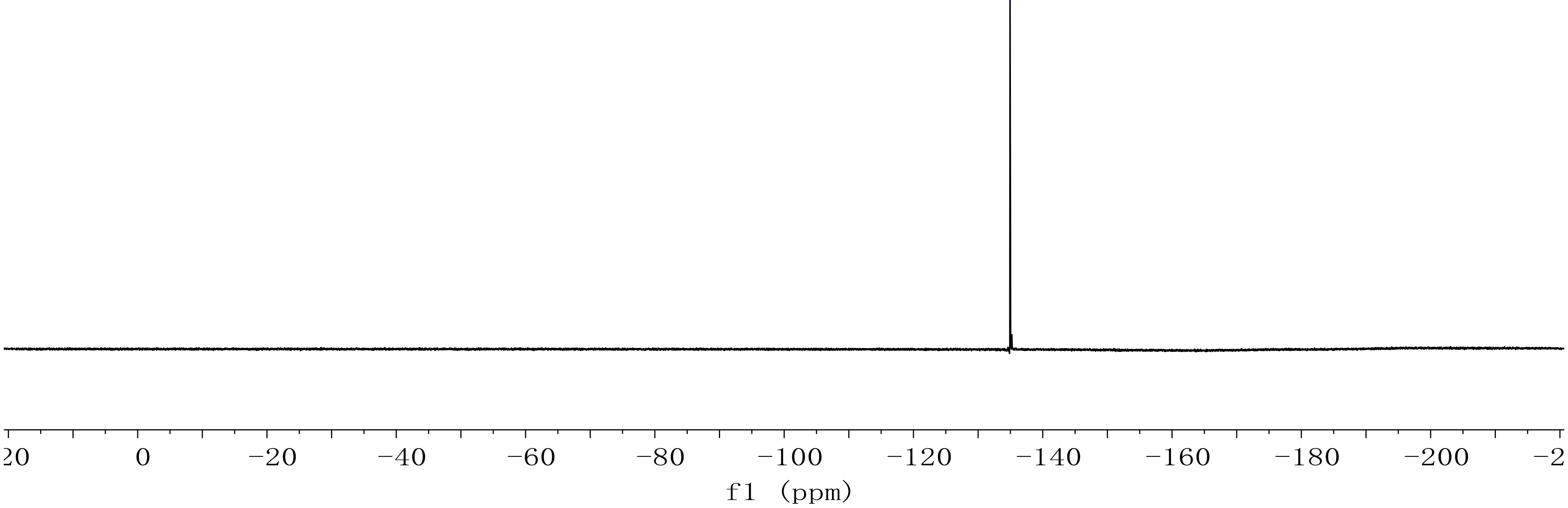

^{1}H NMR of (±)-methyl 2-azidopropanoate 41

$CDCl_3$, 23 ºC

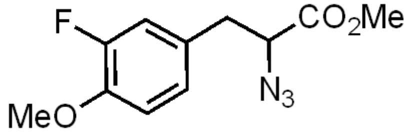

(±)-**41**

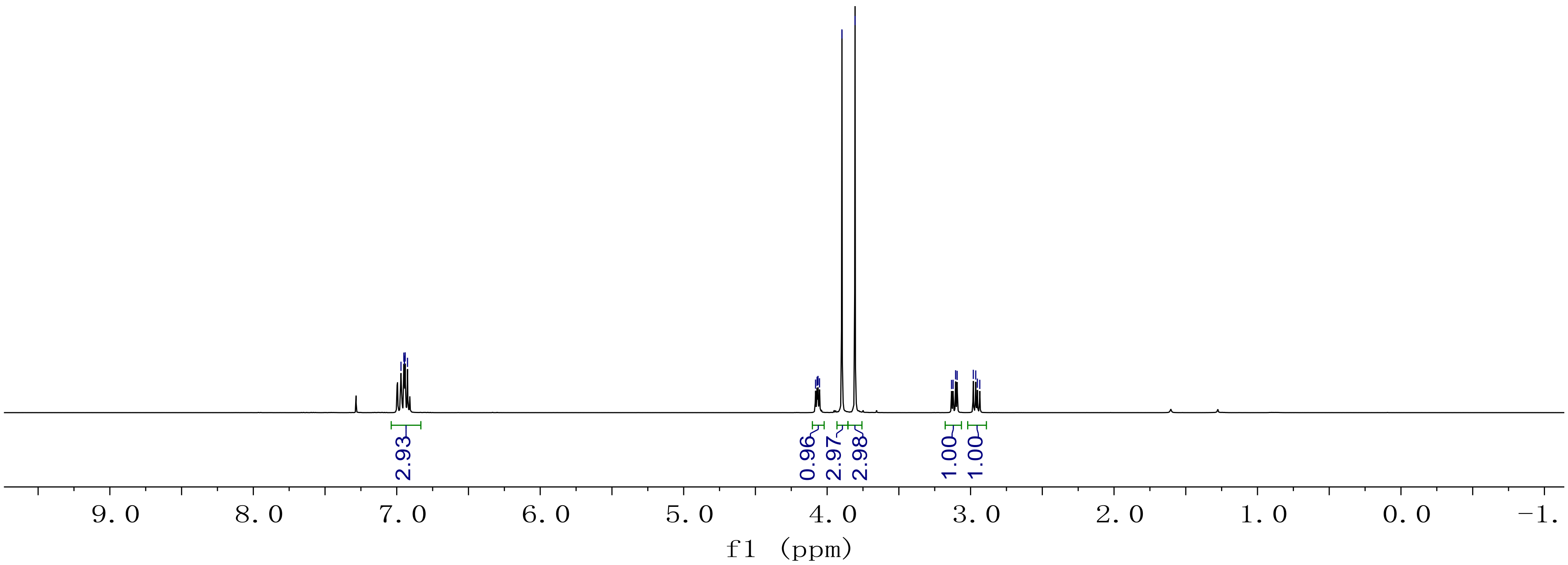

^{13}C NMR of (±)-methyl 2-azidopropanoate 41

CDCl3, 23 ºC

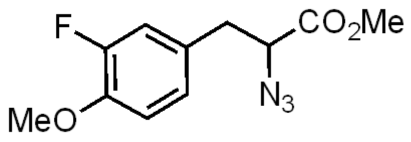

(±)-**41**

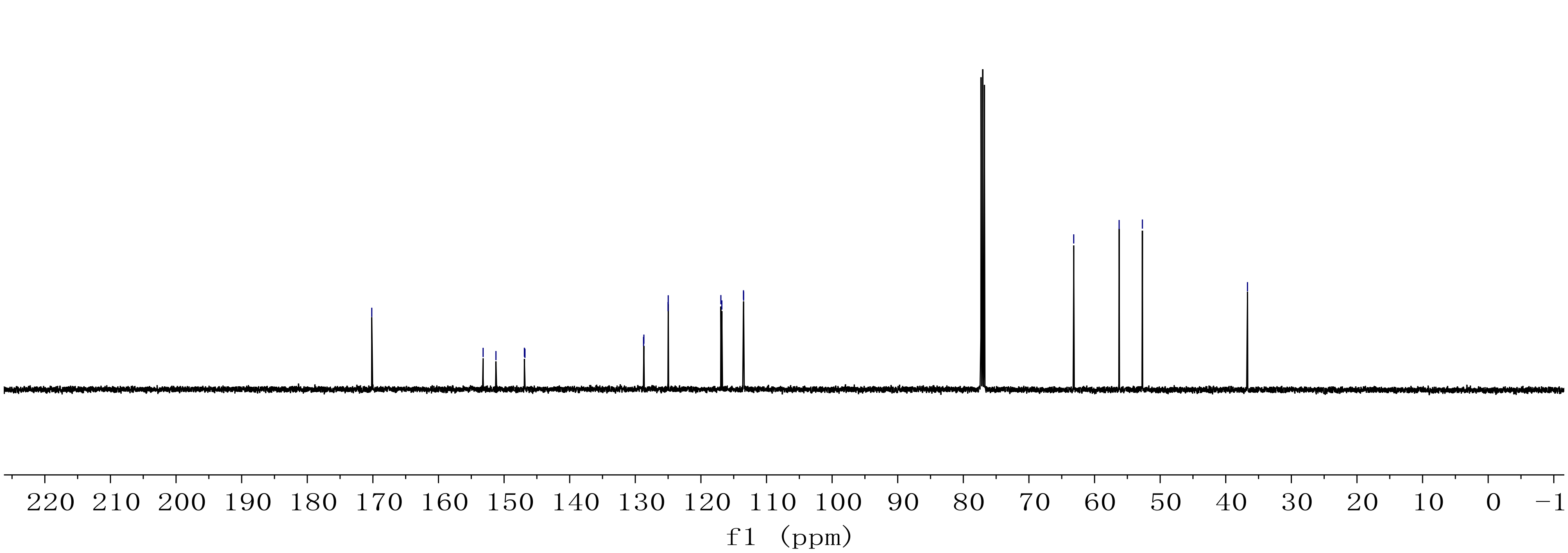

^{19}F NMR of (±)-methyl 2-azidopropanoate 41

$CDCl_3$, 23 ºC

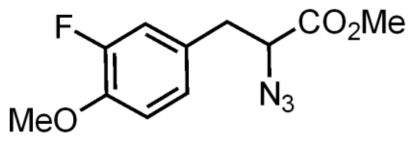

(±)-**41**

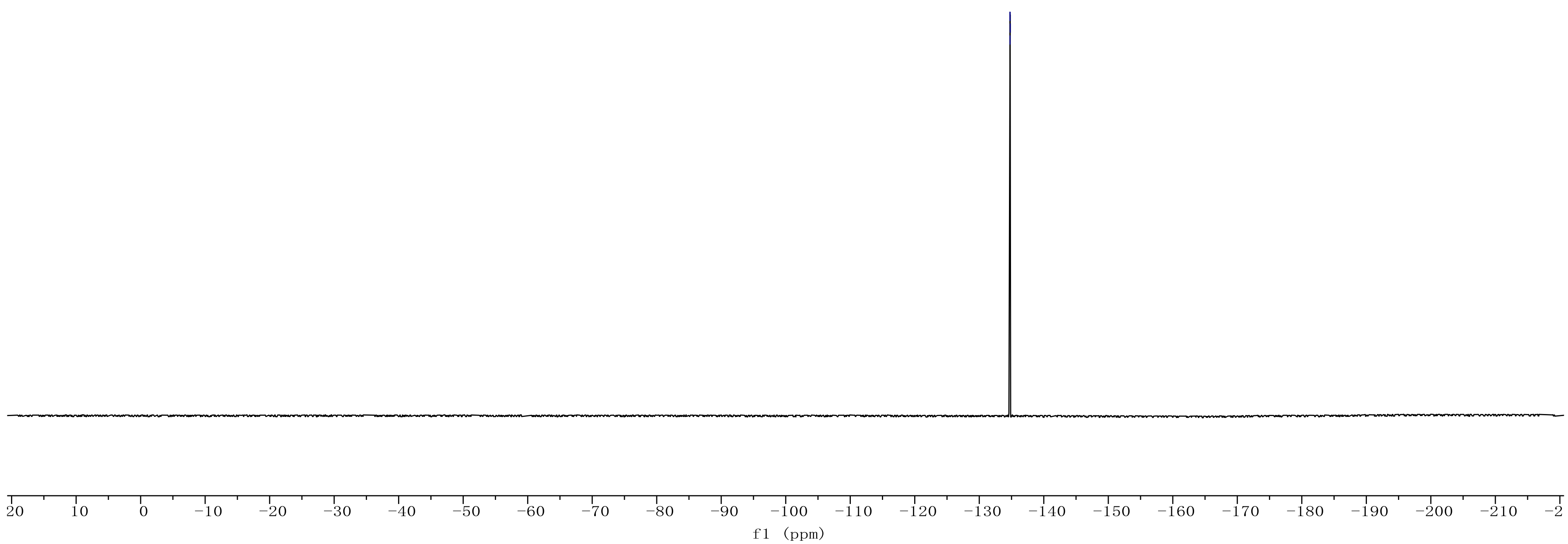

^{1}H NMR of (±)-methyl 2-nitropropanoate 42

$CDCl_3$, 23 ºC

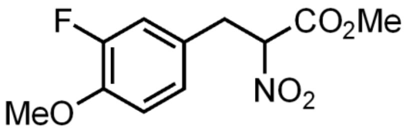

(±)-**42**

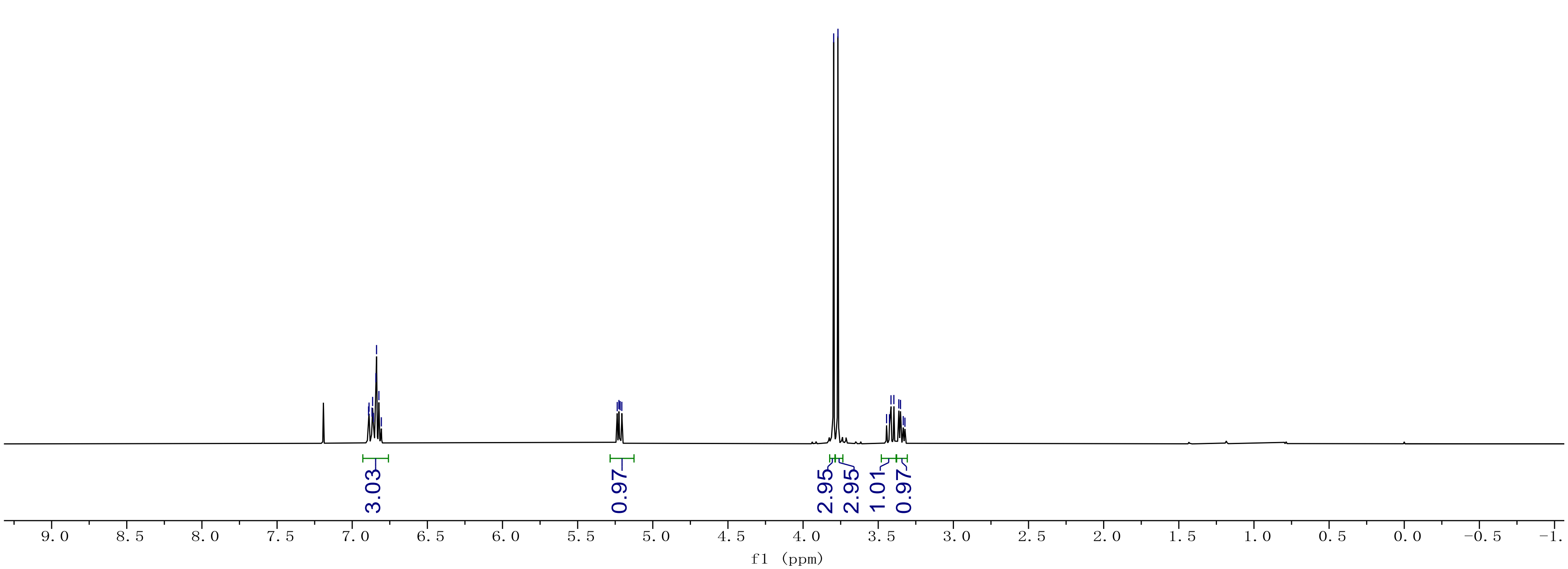

^{13}C NMR of (±)-methyl 2-nitropropanoate 42

$CDCl_3$, 23 ºC

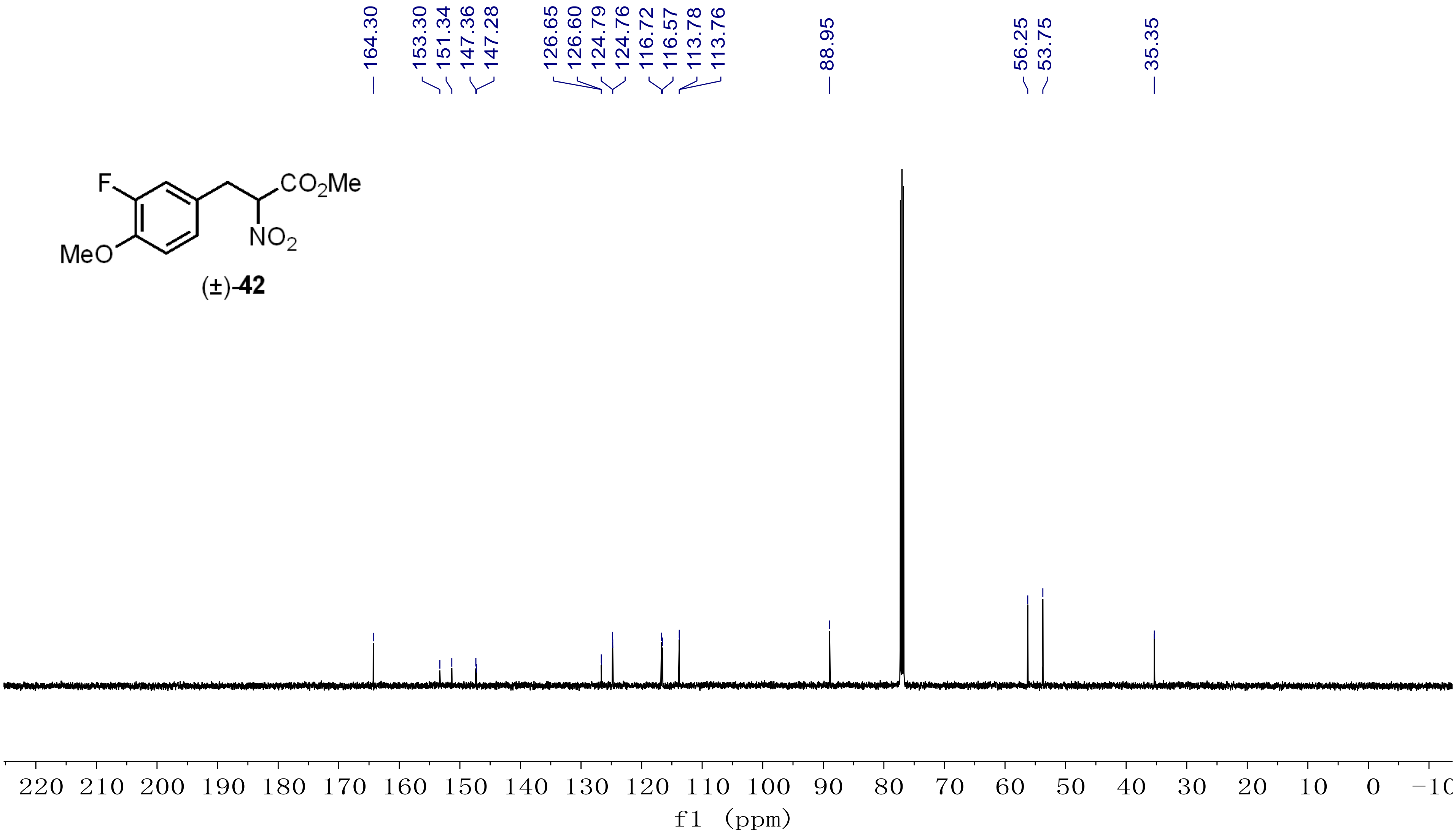

^{1}H NMR of (±)-methyl 2-hydroxy-propanoate 43

$CDCl_3$, 23 ºC

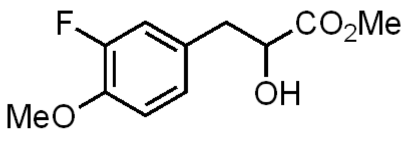

(±)-**43**

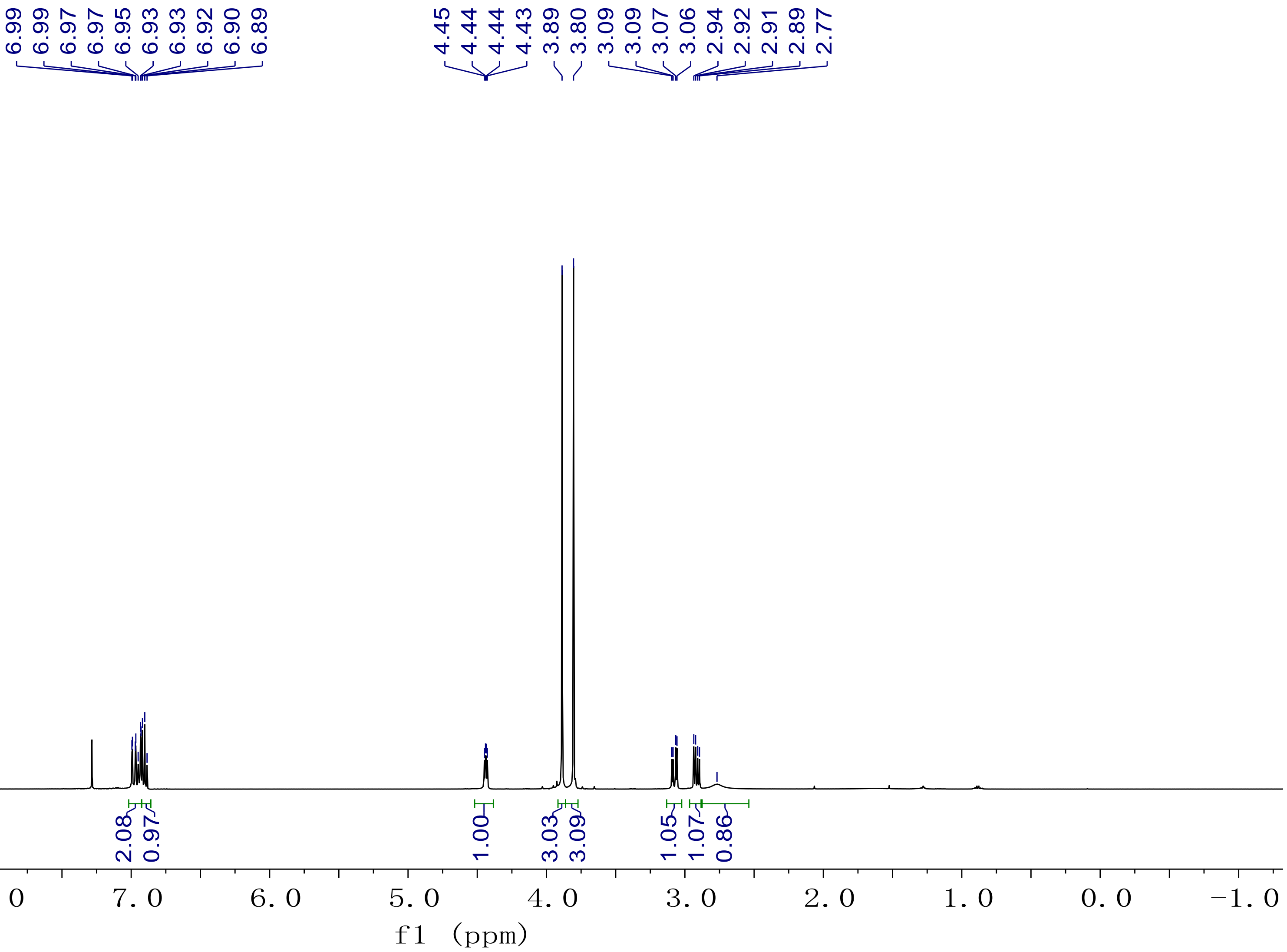

^{13}C NMR of (±)-methyl 2-hydroxy-propanoate 43

$CDCl_3$, 23 ºC

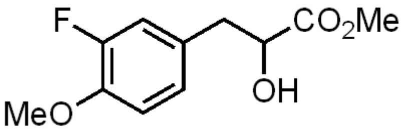

(±)-**43**

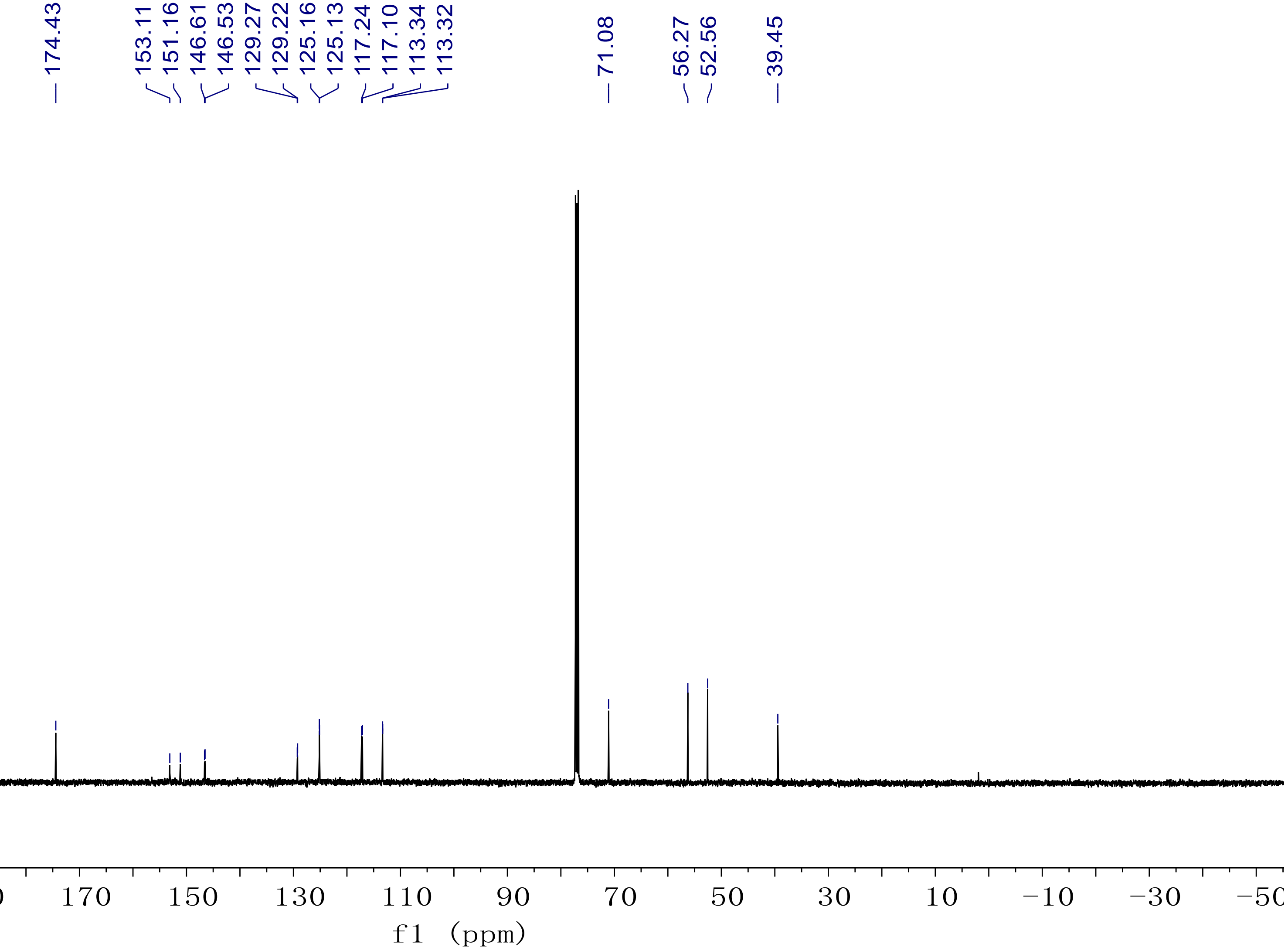

^{19}F NMR of (±)-methyl 2-hydroxy-propanoate 43

$CDCl_3$, 23 ºC

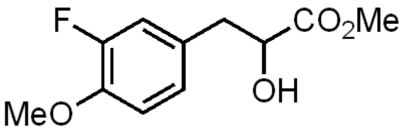

(±)-**43**

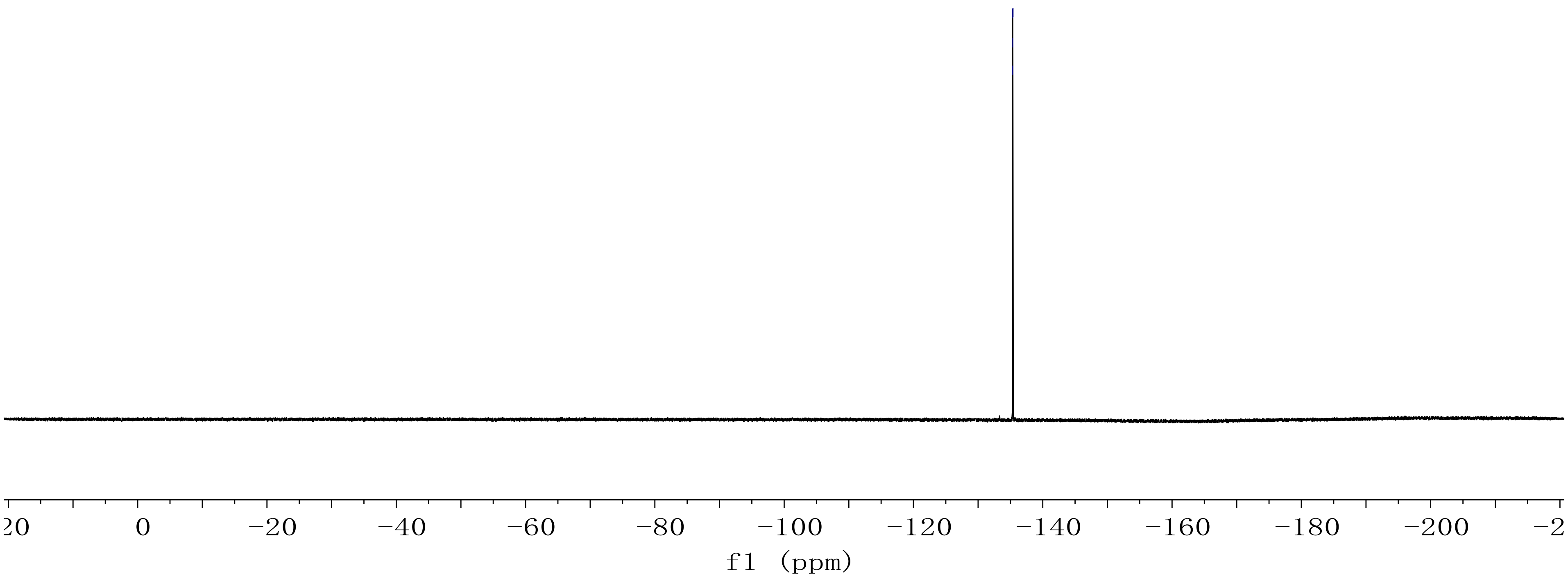

^{1}H NMR of (±)-methyl 2-phenylsulfonylpropanoate 44

$CDCl_3$, 23 ºC

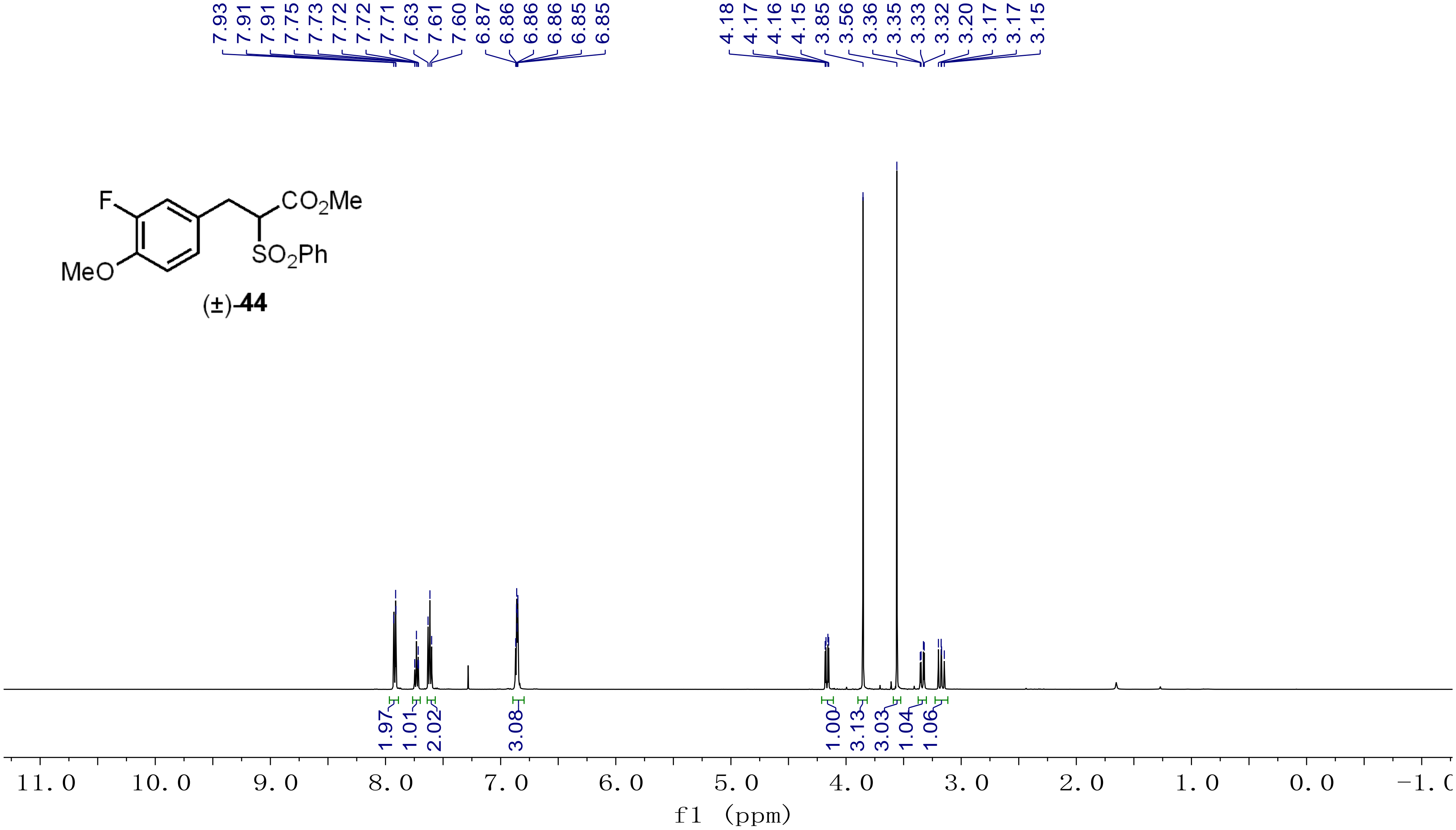

^{13}C NMR of (±)-methyl 2-phenylsulfonylpropanoate 44

$CDCl_3$, 23 ºC

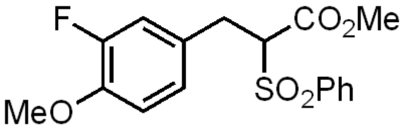

(±)-**44**

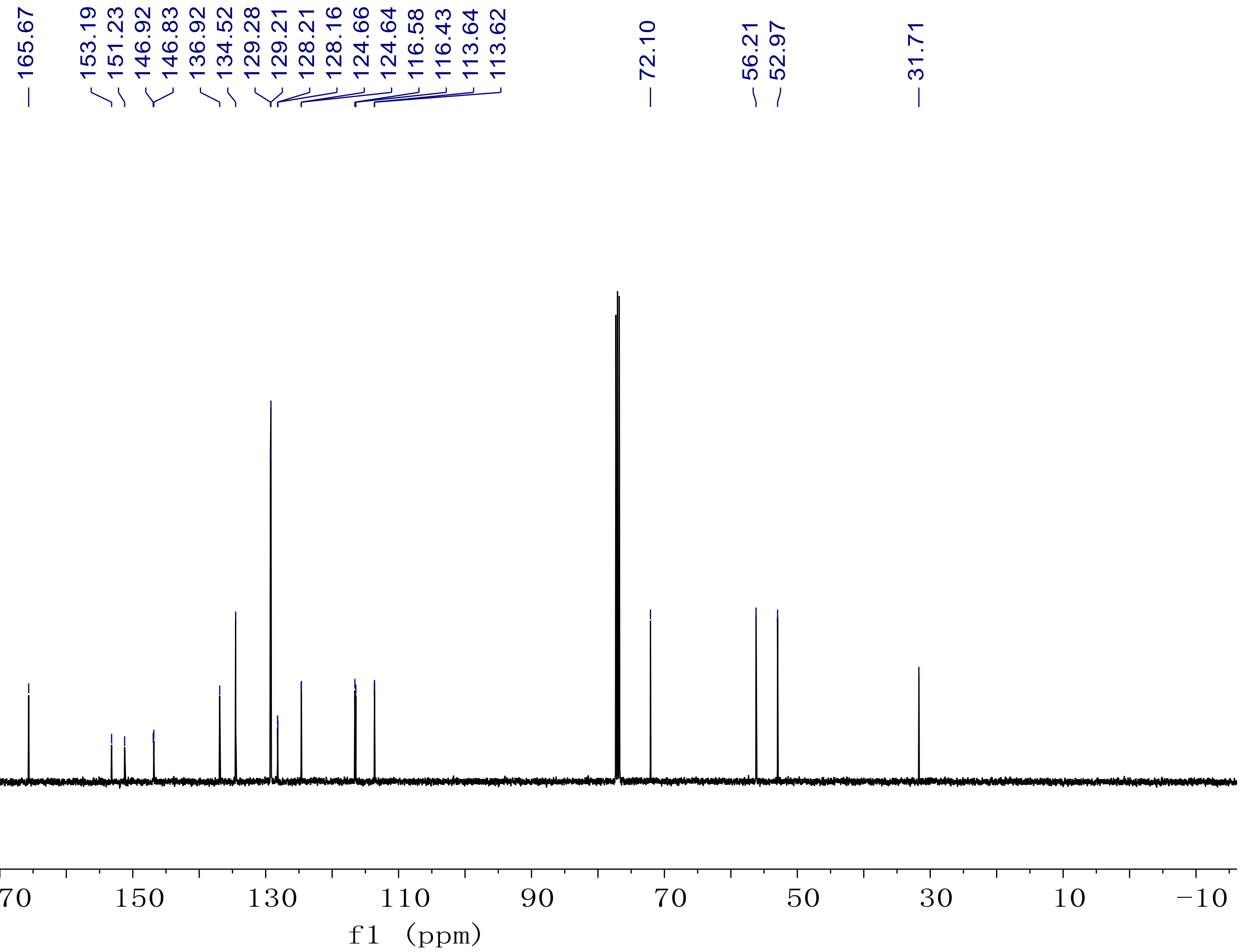

^{19}F NMR of (±)-methyl 2-phenylsulfonylpropanoate 44

$CDCl_3$, 23 ºC

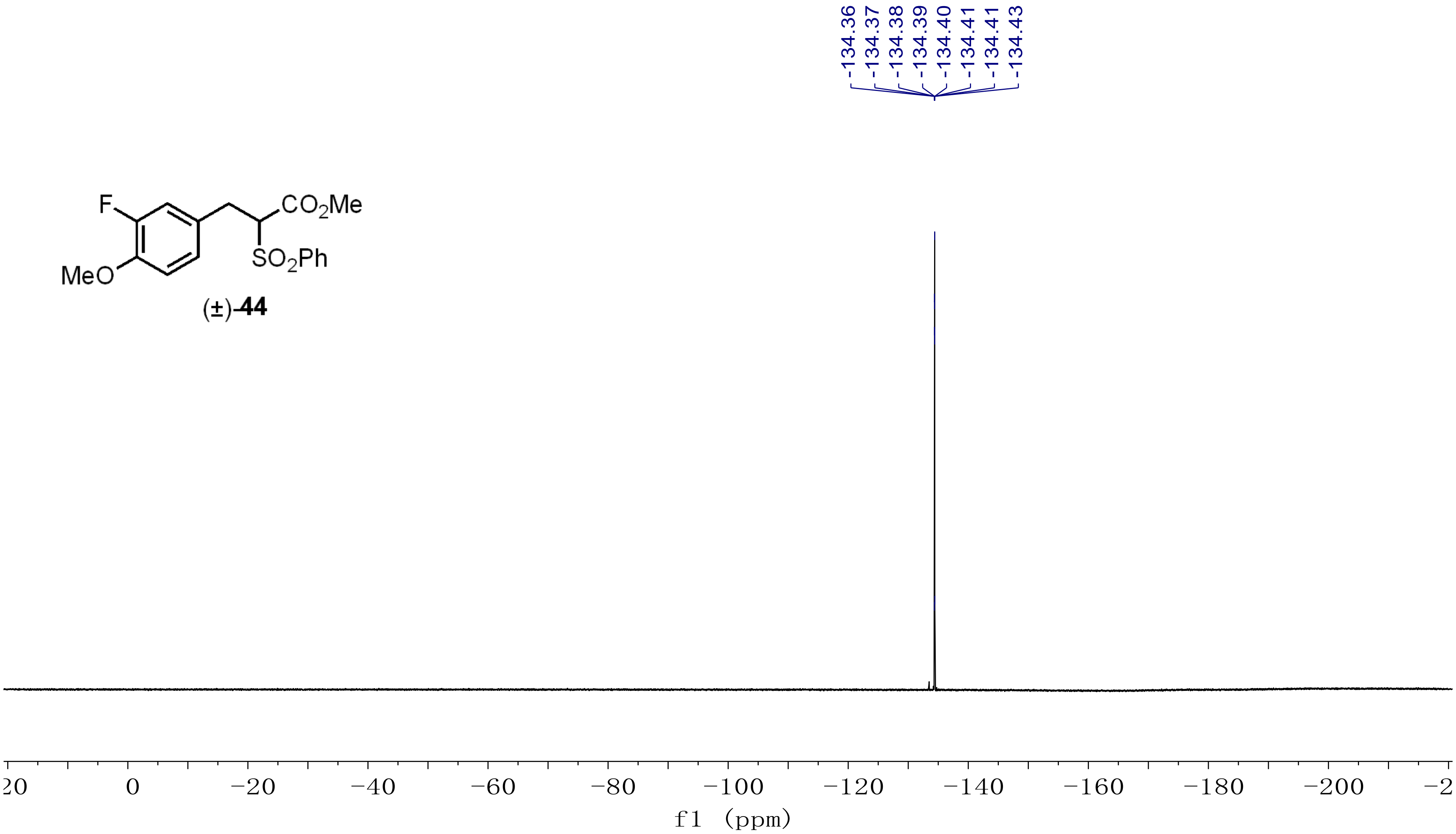

^{1}H NMR of (±)-methyl 2-acetylthiopropanoate 45

$CDCl_3$, 23 ºC

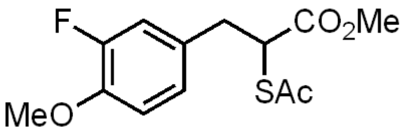

(±)-**45**

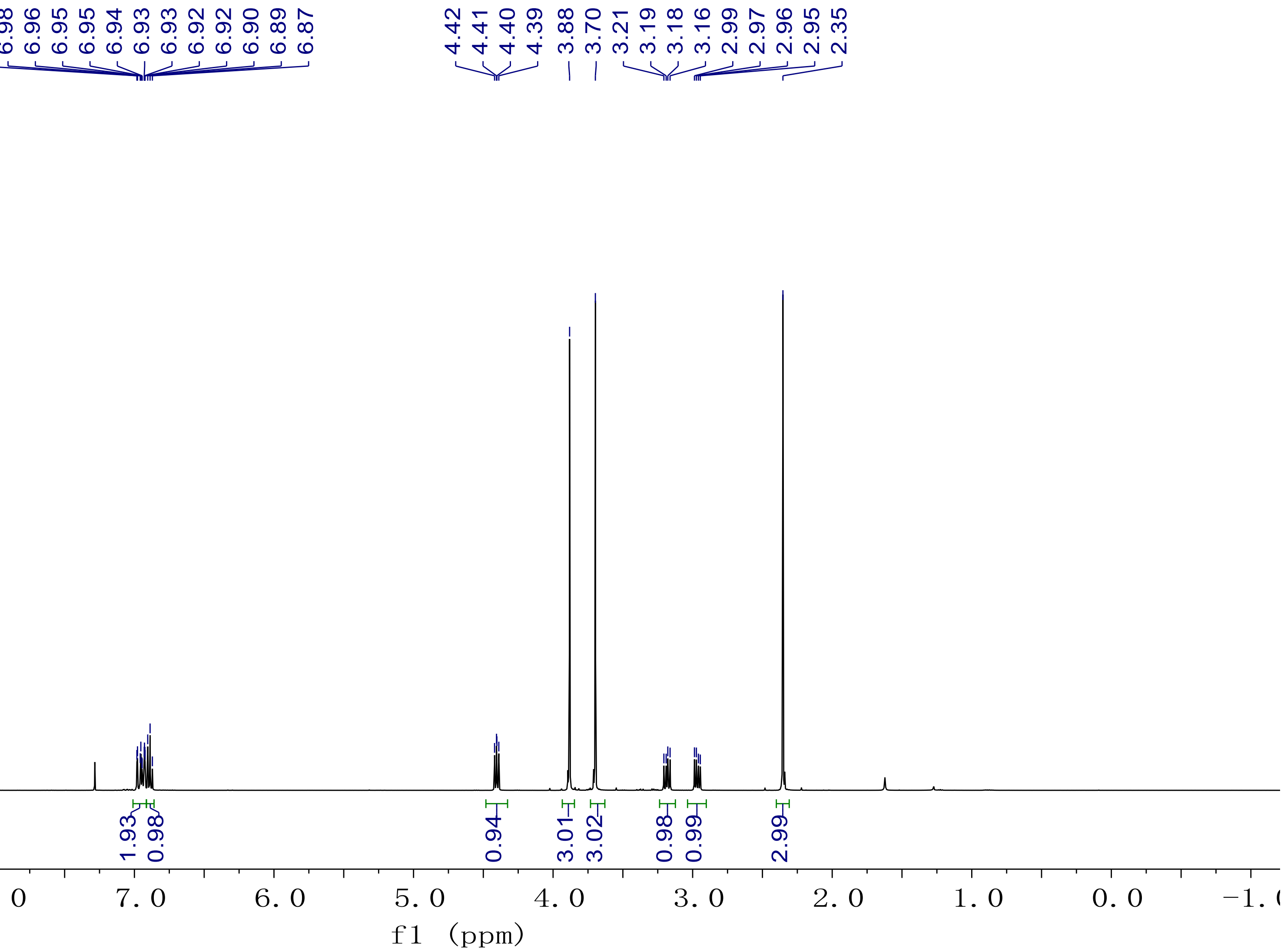

^{13}C NMR of (±)-methyl 2-acetylthiopropanoate 45

CDCl$_3$, 23 ºC

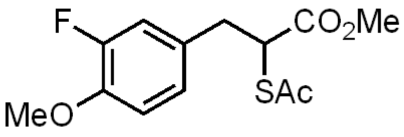

(±)-**45**

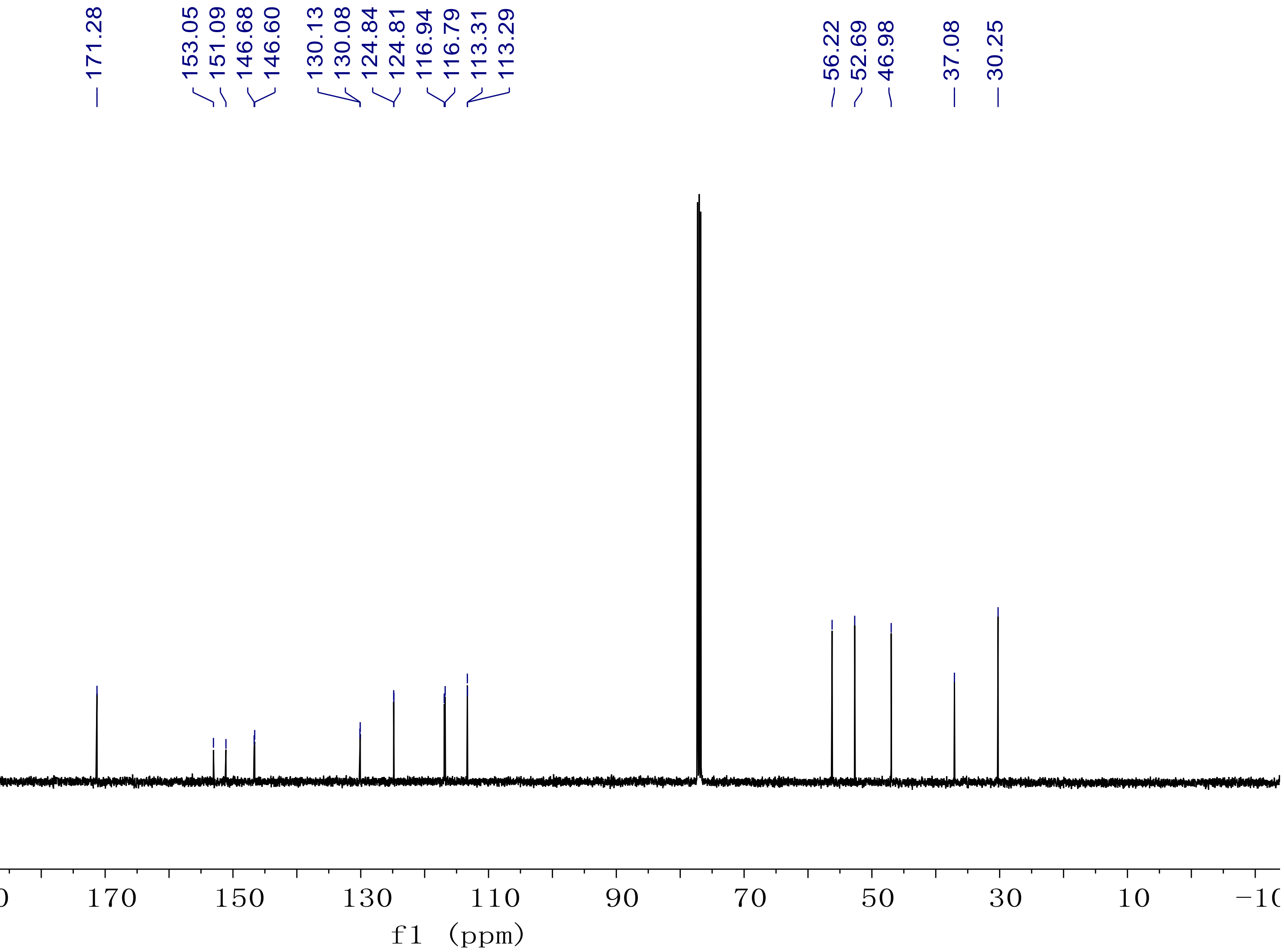

^{19}F NMR of (±)-methyl 2-acetylthiopropanoate 45

CDCl$_3$, 23 ºC

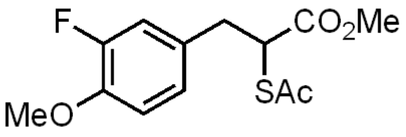

(±)-**45**

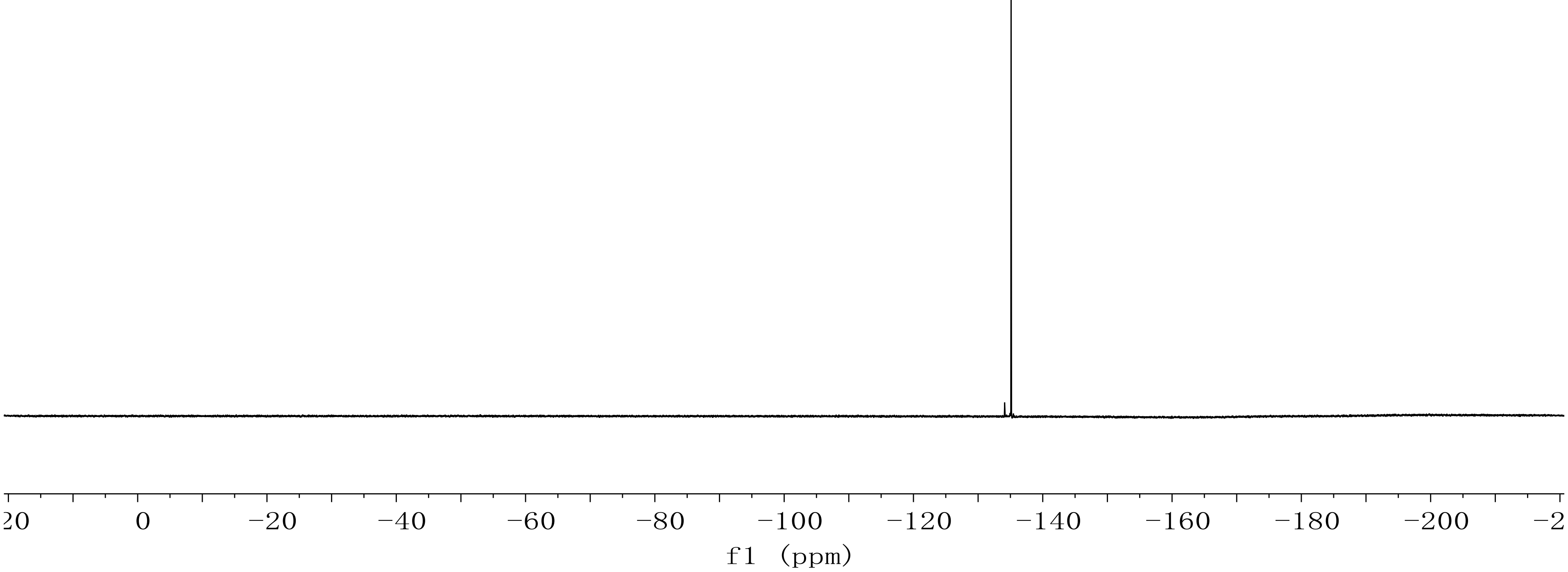

^{1}H NMR of (±)-methyl 2-phenylthiopropanoate 46

$CDCl_3$, 23 ºC

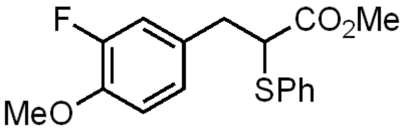

(±)-**46**

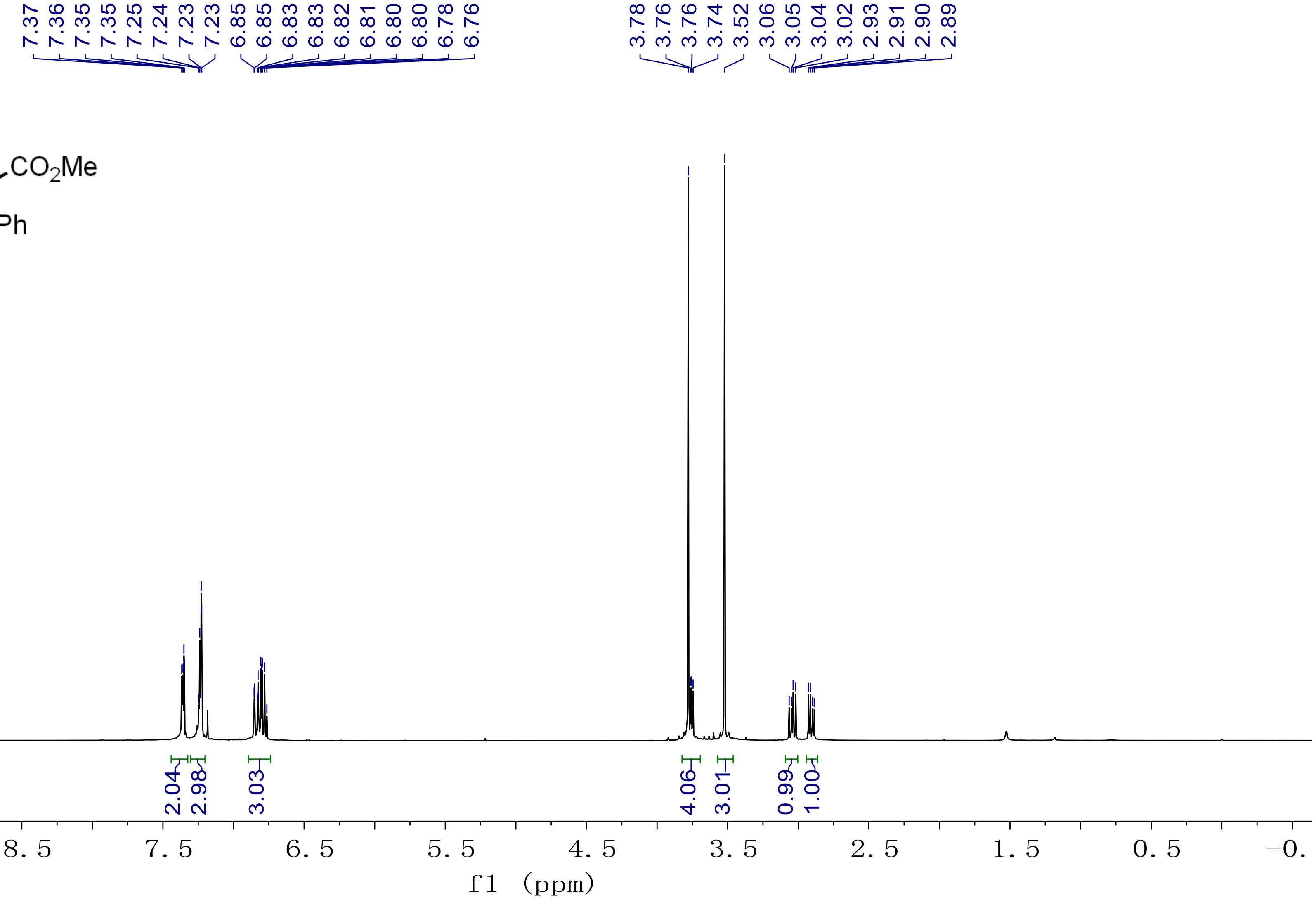

^{13}C NMR of (±)-methyl 2-phenylthiopropanoate 46

$CDCl_3$, 23 ºC

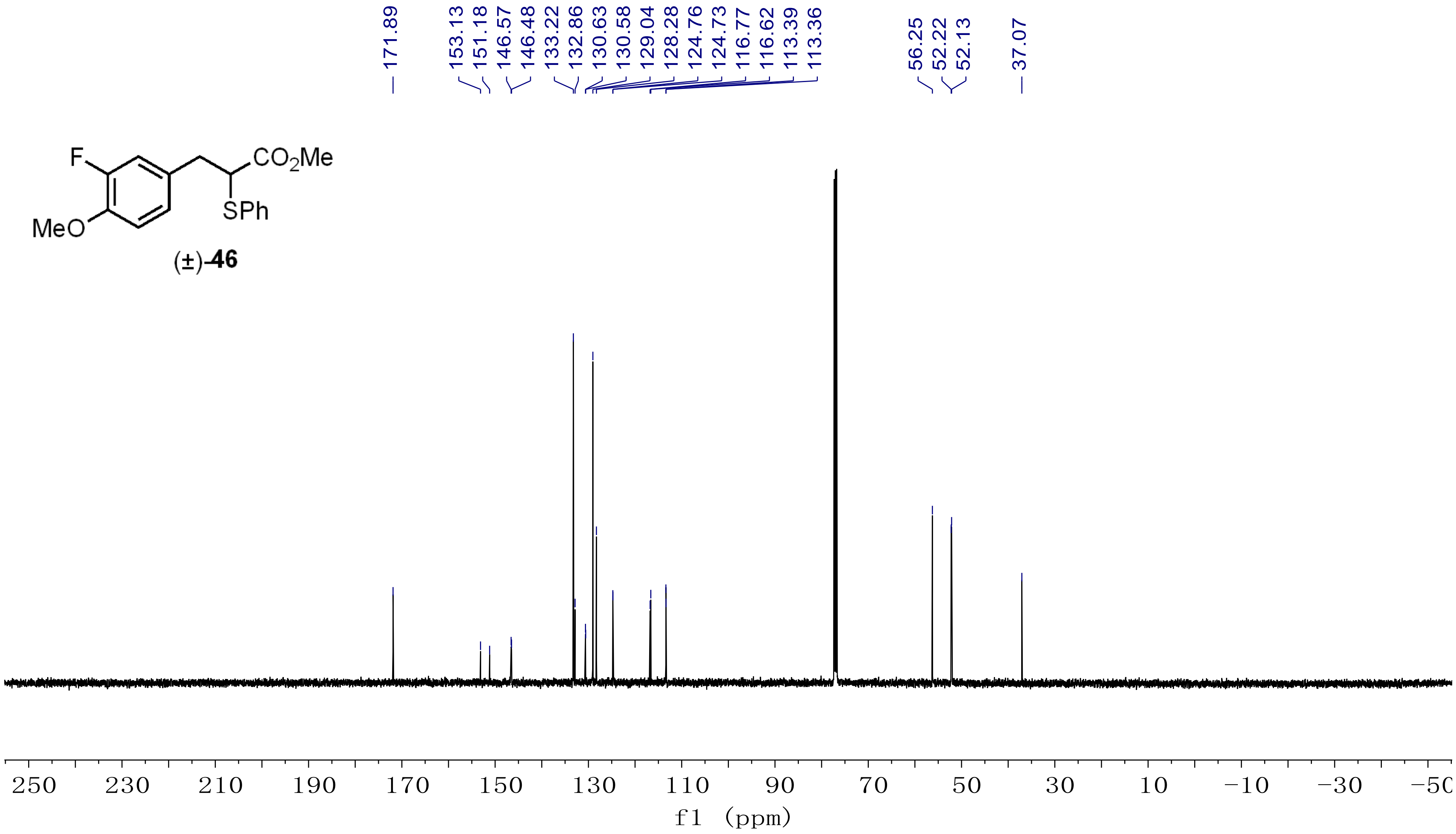

(±)-**46**

^{19}F NMR of (±)-methyl 2-phenylthiopropanoate 46

$CDCl_3$, 23 ºC

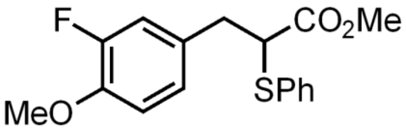

(±)-**46**

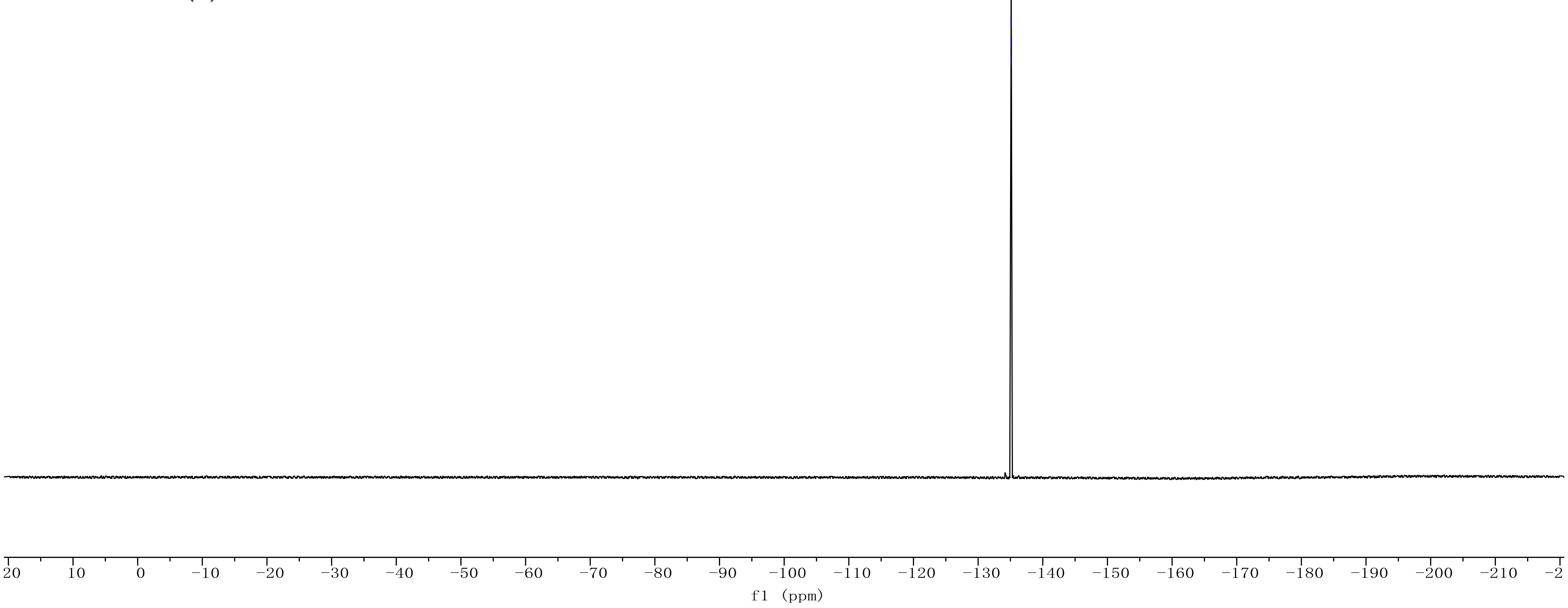

^{1}H NMR of (±)-methyl 2-phenylselanylpropanoate 47

$CDCl_3$, 23 ºC

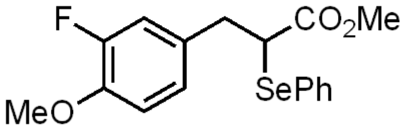

(±)-**47**

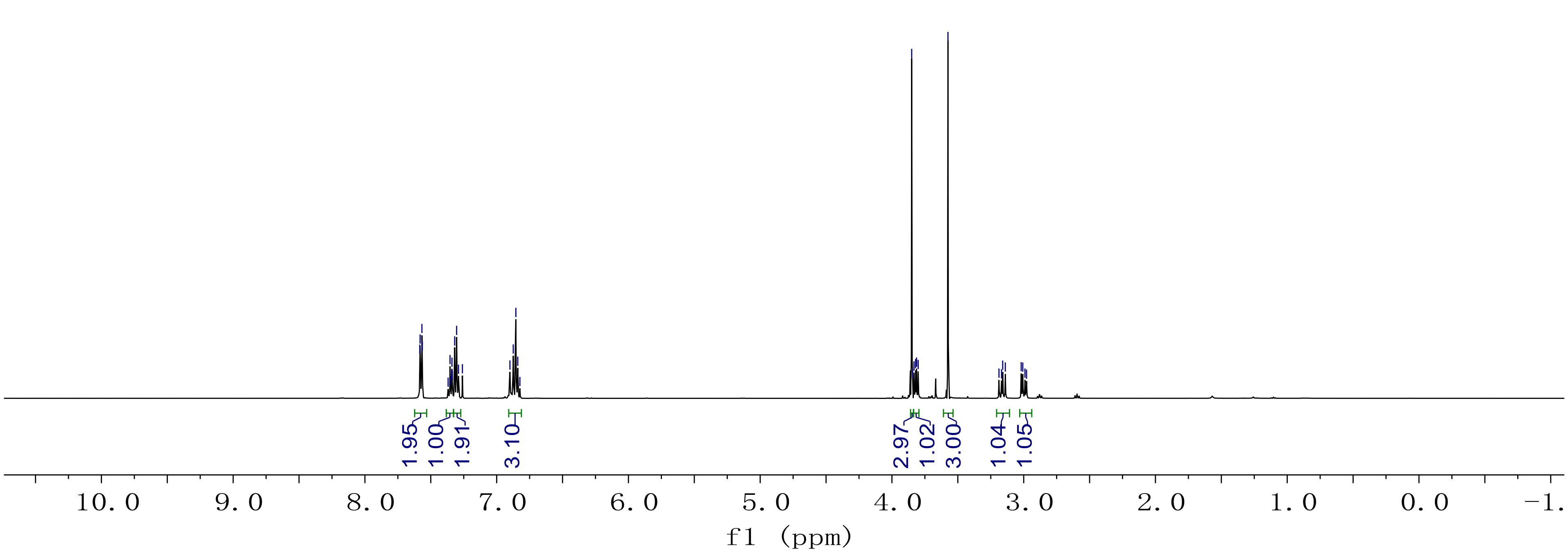

^{13}C NMR of (±)-methyl 2-phenylselanylpropanoate 47

$CDCl_3$, 23 ºC

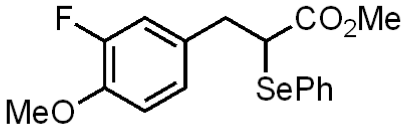

(±)-**47**

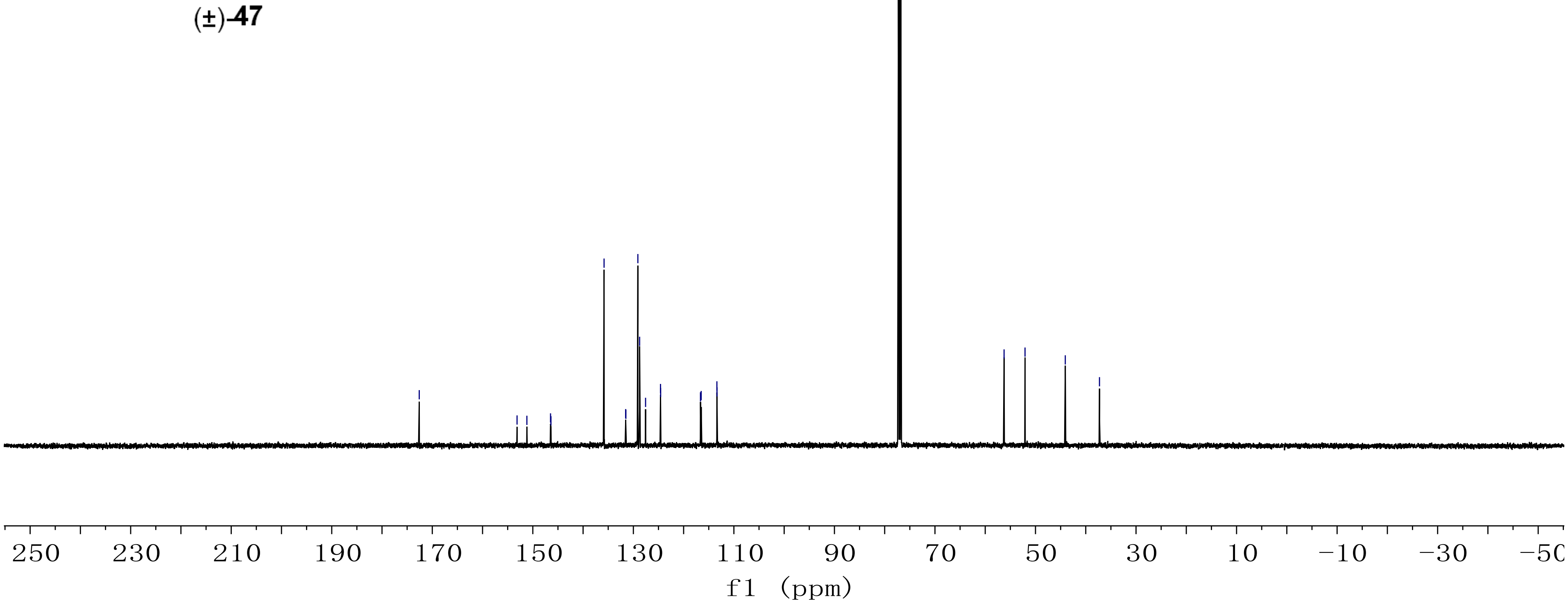

^{19}F NMR of (±)-methyl 2-phenylselanylpropanoate 47

$CDCl_3$, 23 ºC

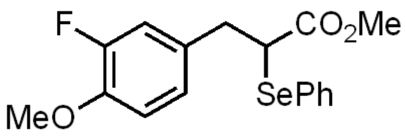

(±)-**47**

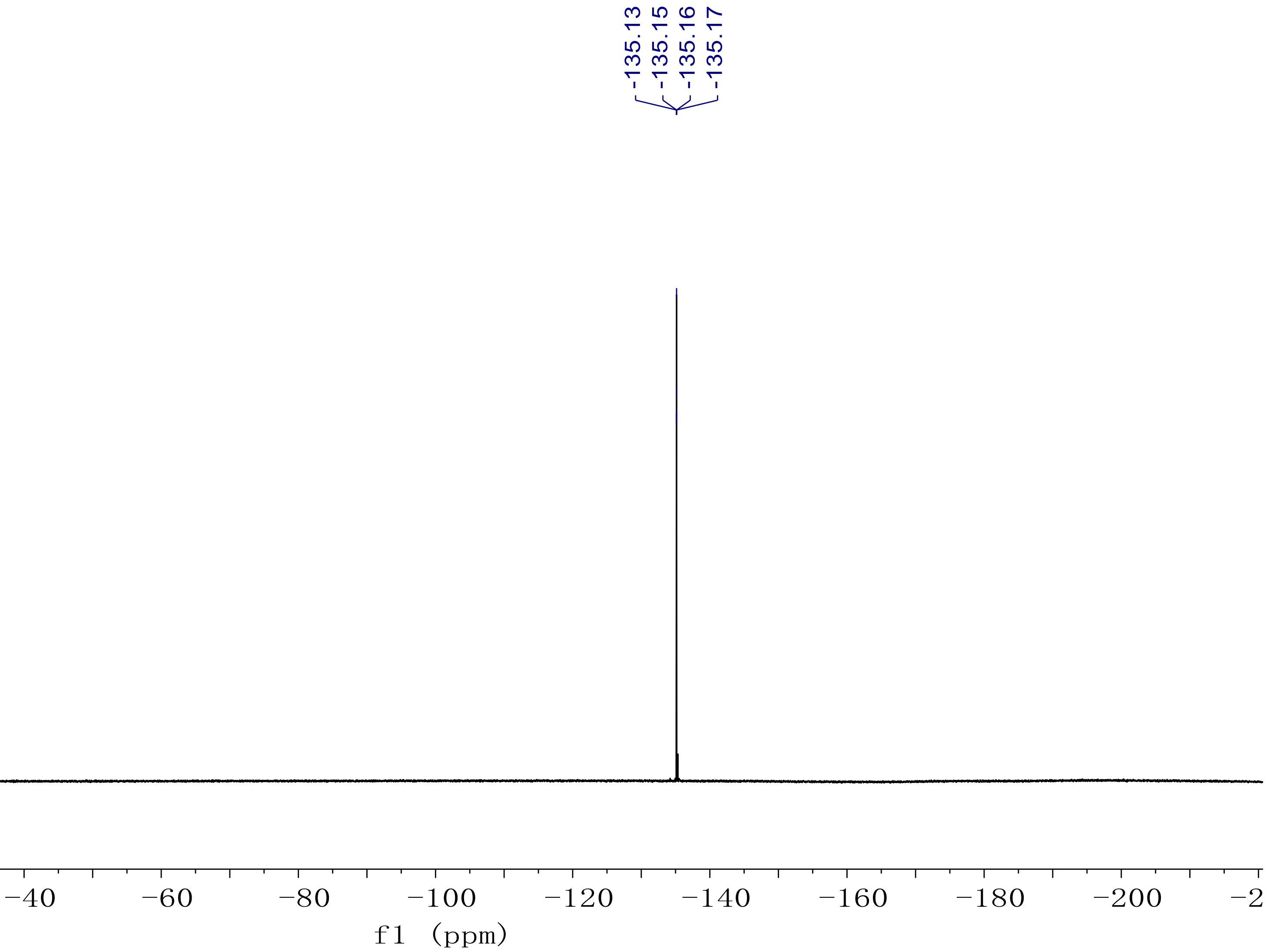

^{1}H NMR of (±)-methyl 2-diethoxyphosphorylpropanoate 48

$CDCl_3$, 23 ºC

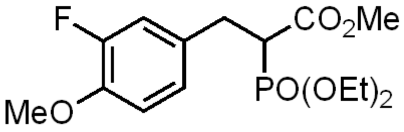

(±)-**48**

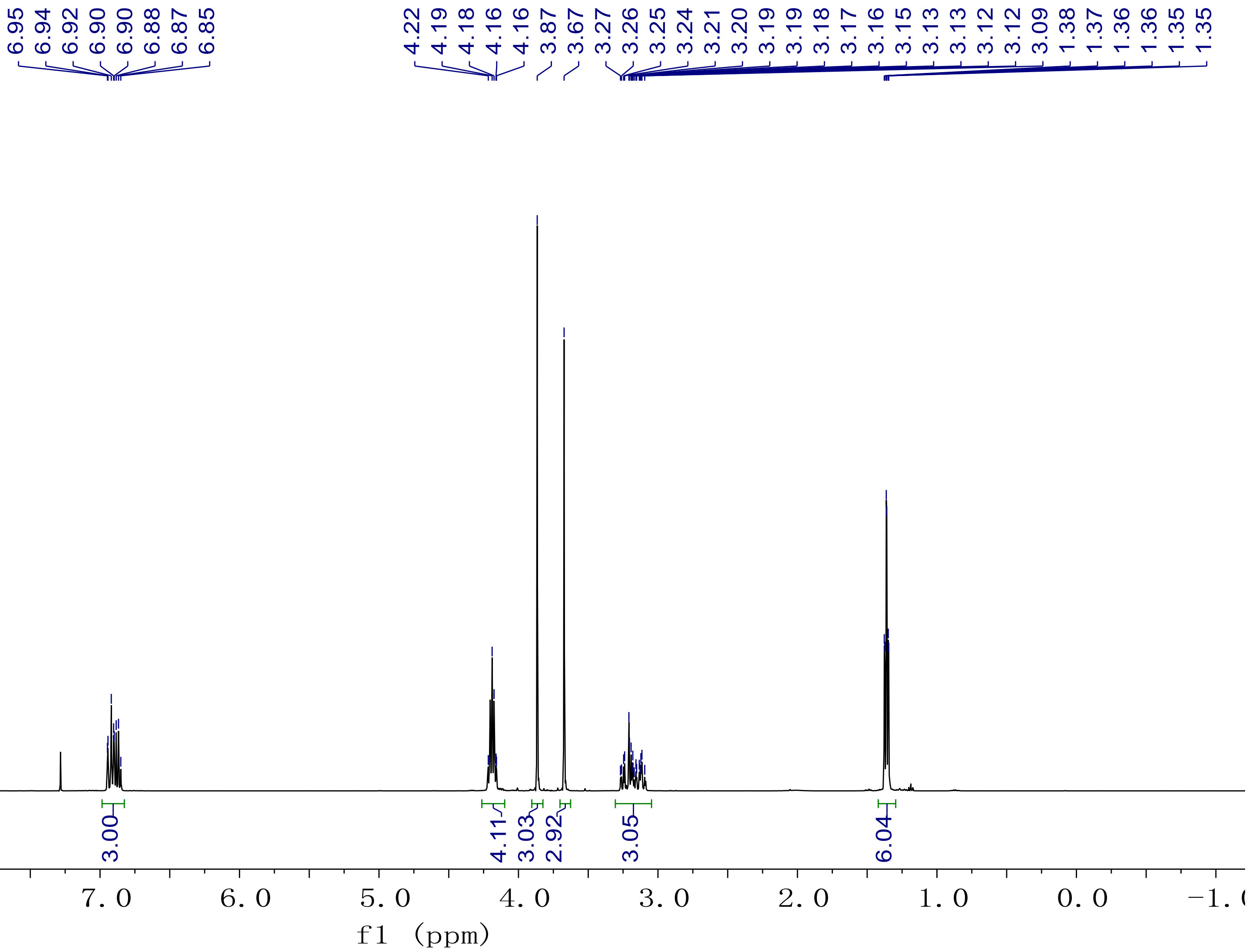

^{13}C NMR of (±)-methyl 2-diethoxyphosphorylpropanoate 48

$CDCl_3$, 23 ºC

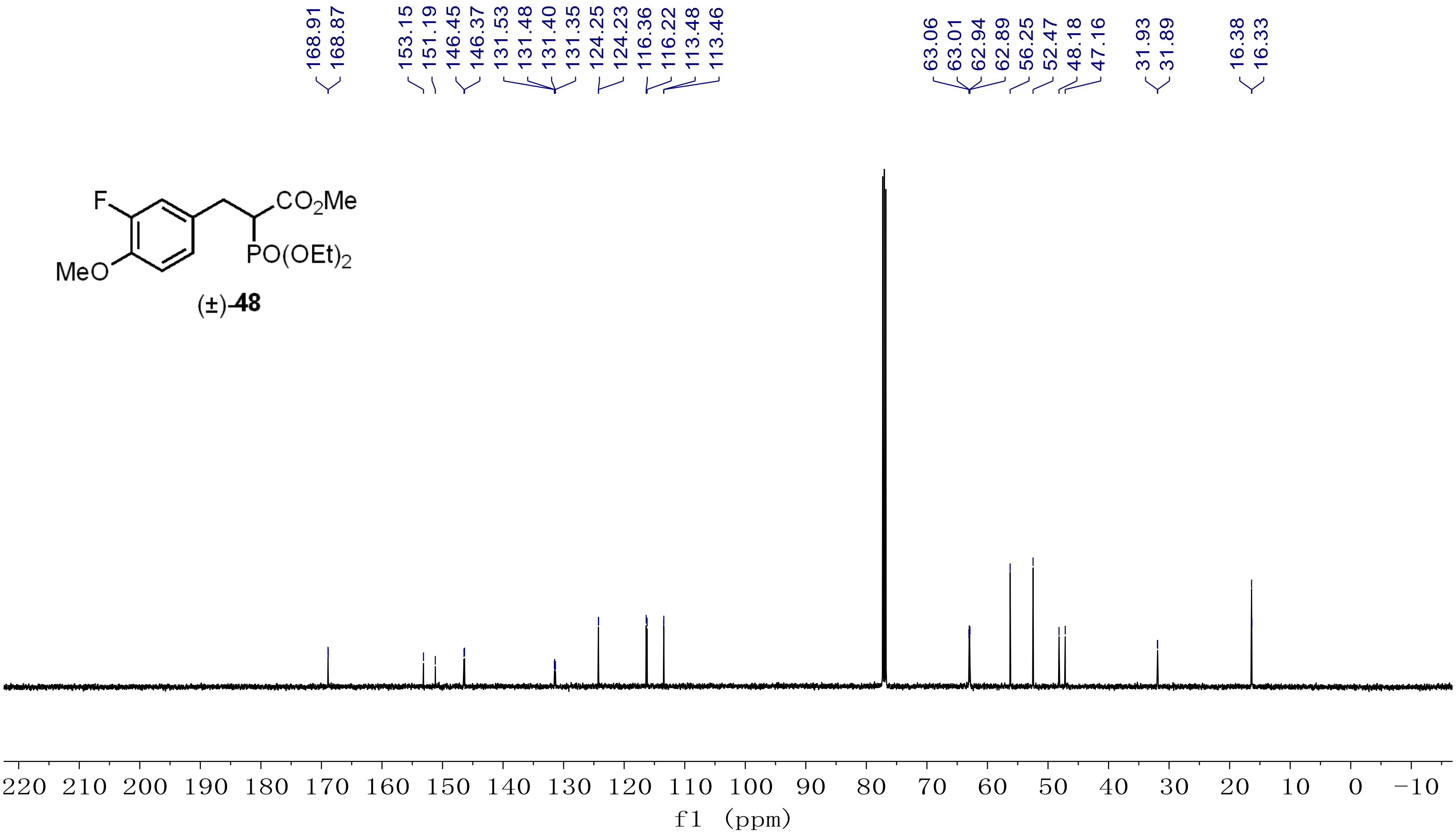

^{31}P NMR of (±)-methyl 2-diethoxyphosphorylpropanoate 48

$CDCl_3$, 23 ºC

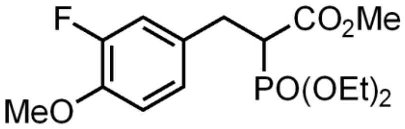

(±)-**48**

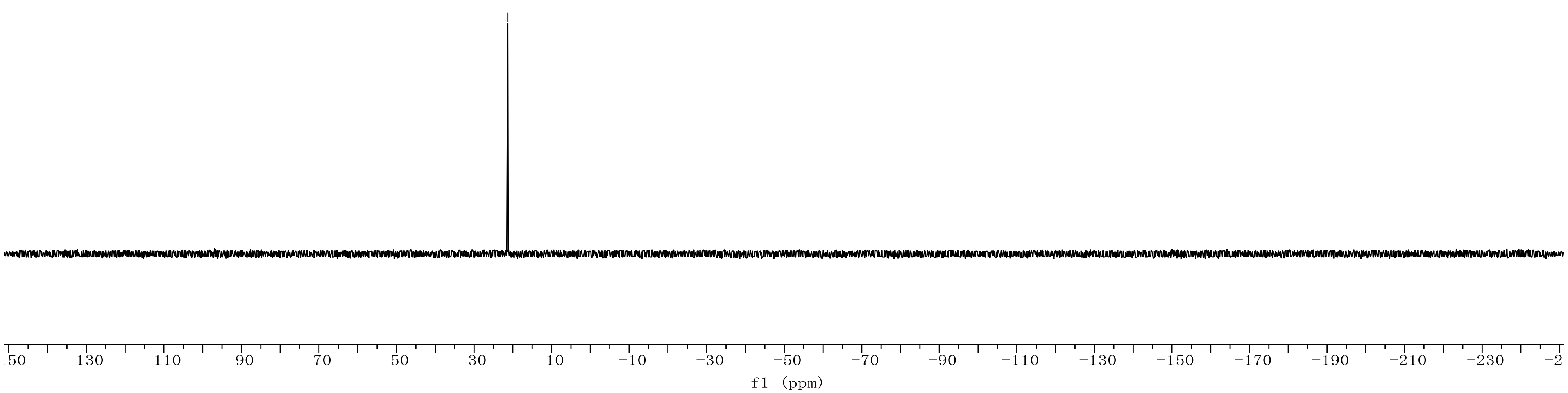

^{1}H NMR of (±)-thiazolidinediones derivative 49

$CDCl_3$, 23 ºC

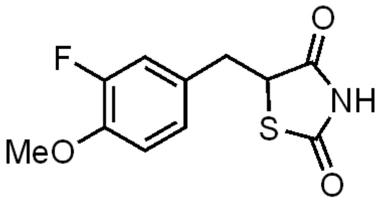

(±)-**49**

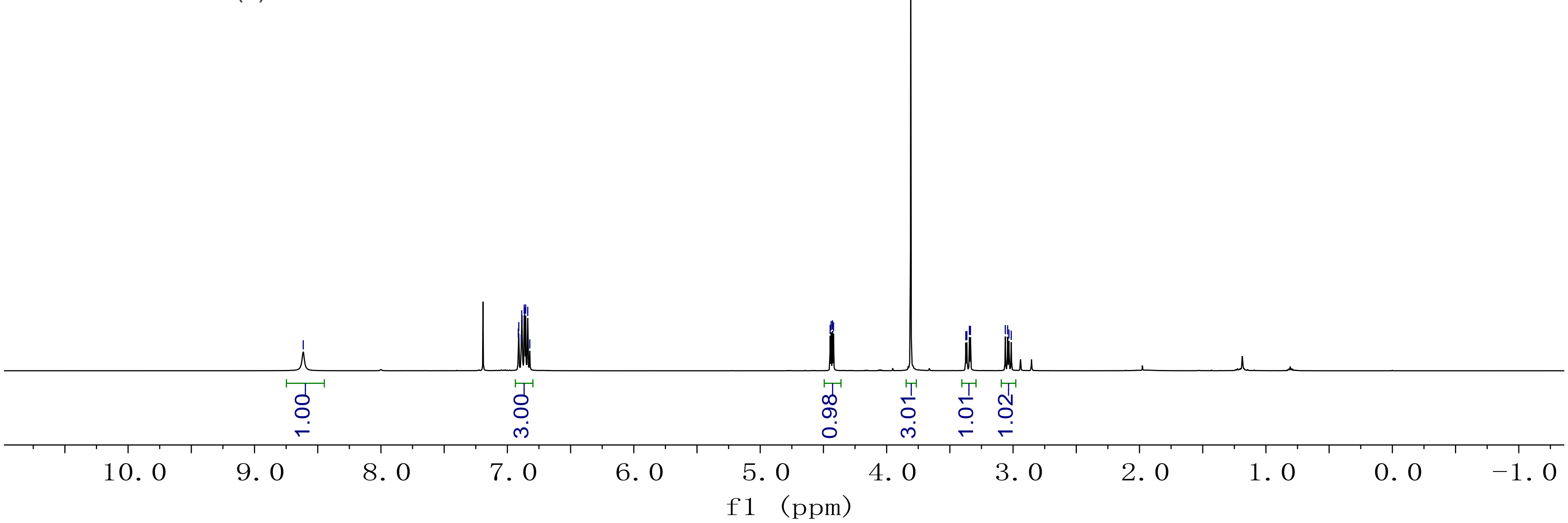

^{13}C NMR of (±)-thiazolidinediones derivative 49

$CDCl_3$, 23 ºC

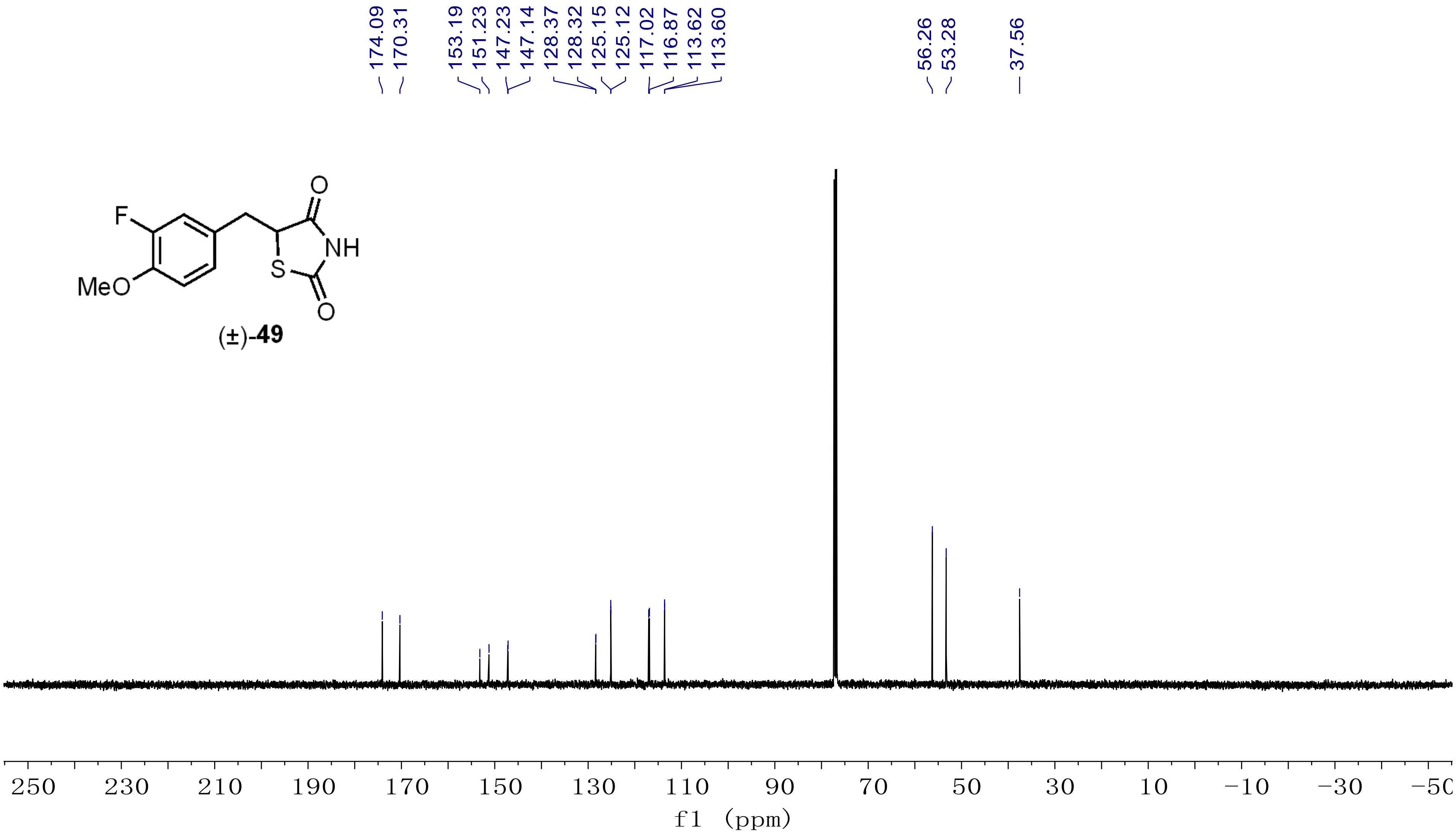

^{19}F NMR of (±)-thiazolidinediones derivative 49

CDCl$_3$, 23 ºC

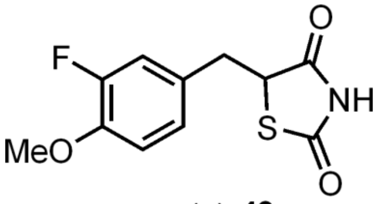

(±)-**49**

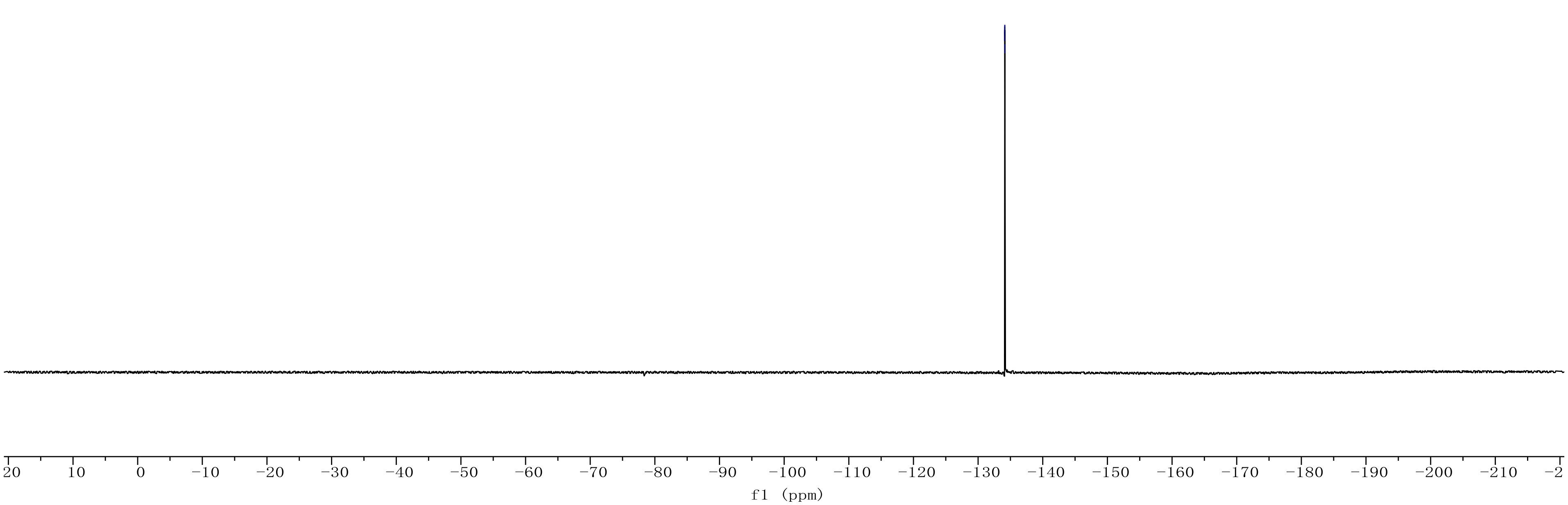

^{1}H NMR of (±)-2*H*-benzo[*b*]-[1,4]thiazin-3(4*H*)-one 50

$CDCl_3$, 23 ºC

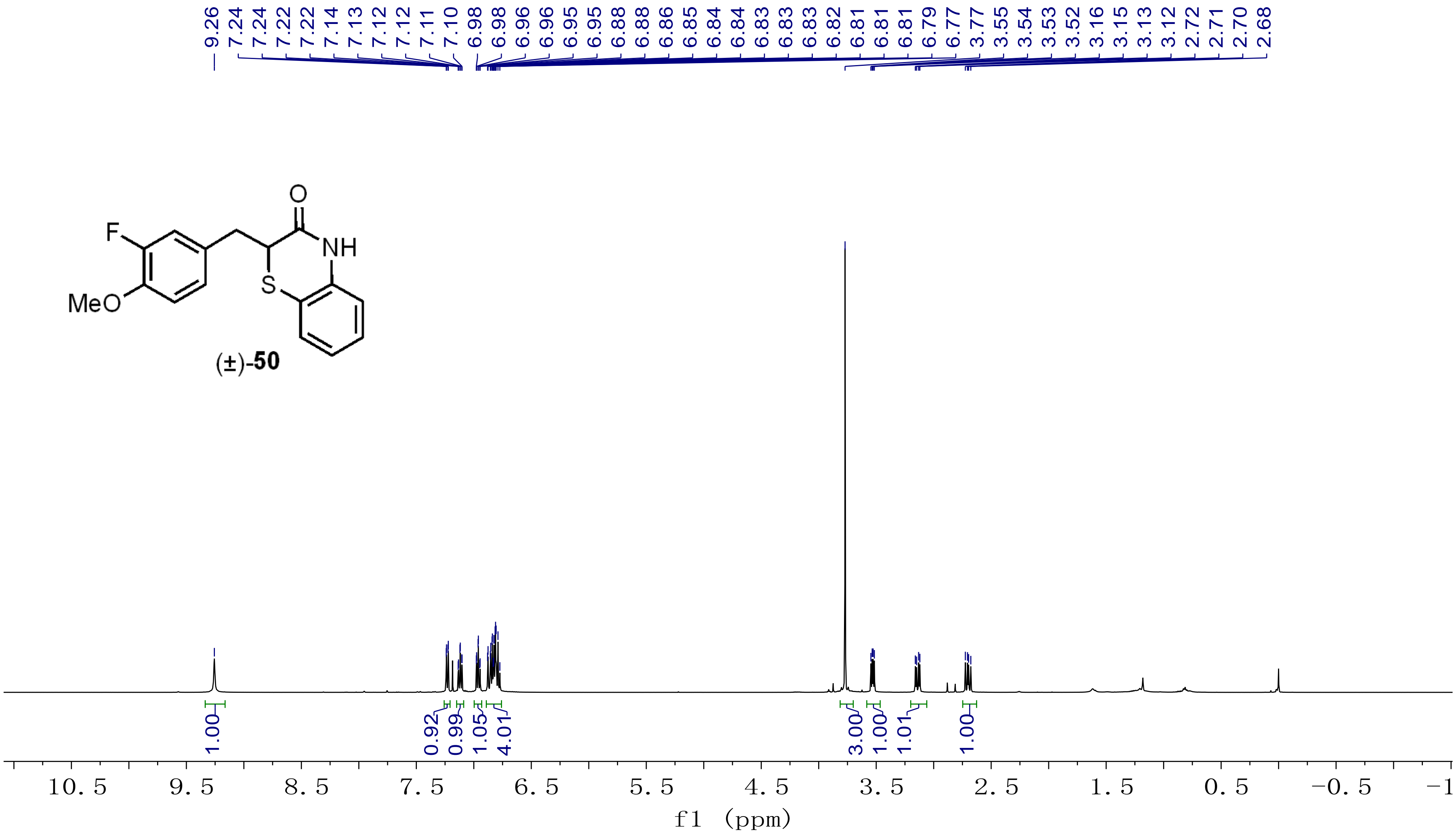

^{13}C NMR of (±)-2*H*-benzo[*b*]-[1,4]thiazin-3(4*H*)-one 50

$CDCl_3$, 23 ºC

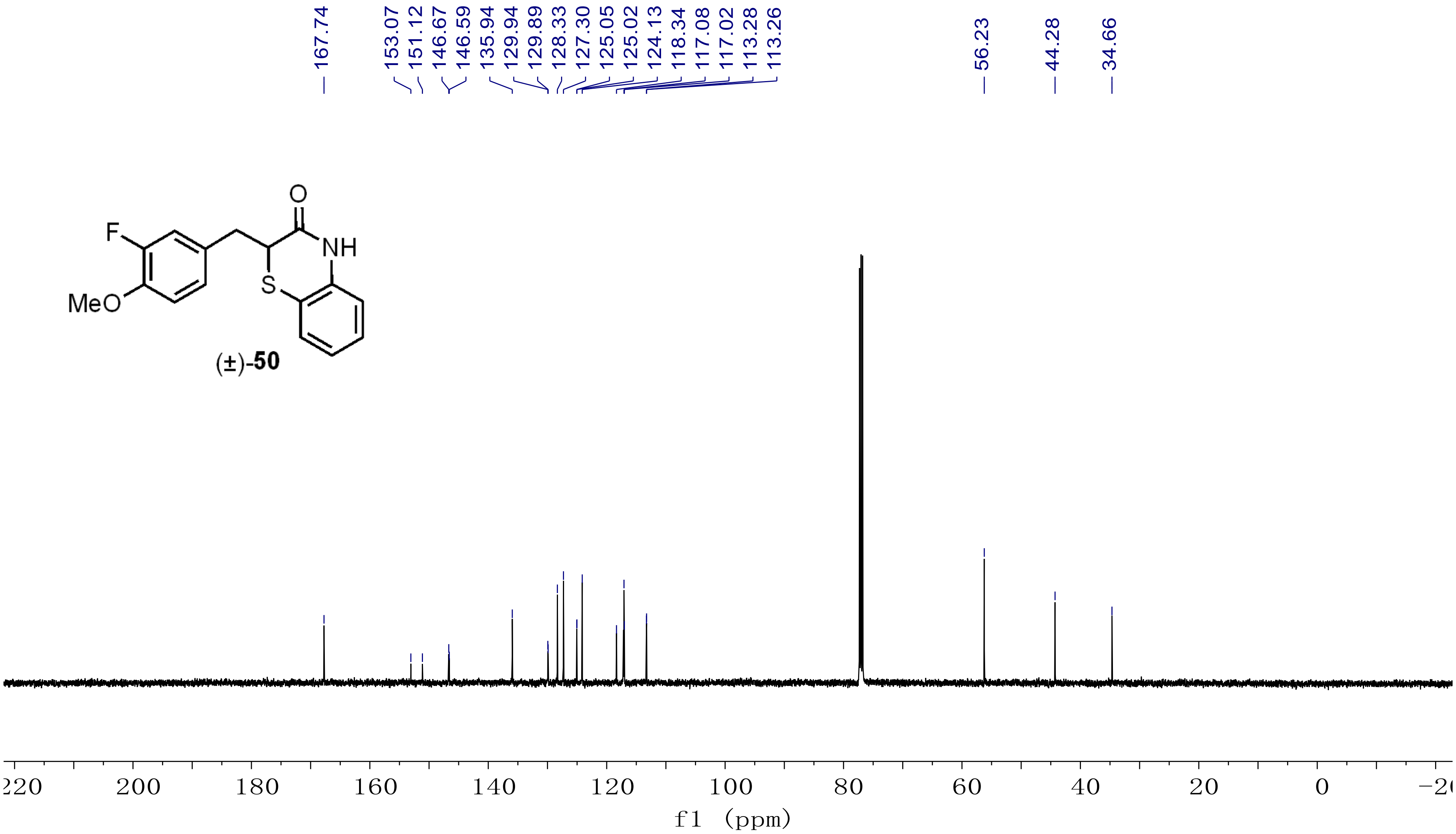

(±)-**50**

^{19}F NMR of (±)-2*H*-benzo[*b*]-[1,4]thiazin-3(4*H*)-one 50

CDCl$_3$, 23 ºC

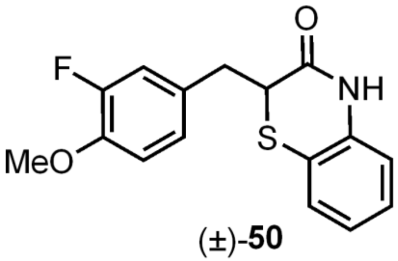

(±)-**50**

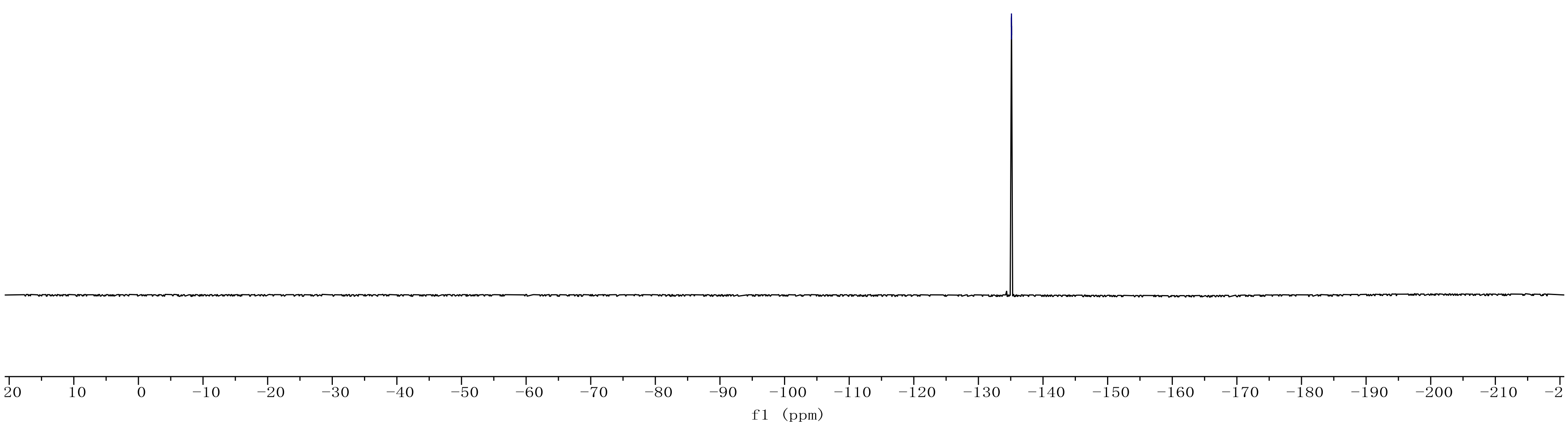

REFERENCES

1. Fulmer, G. R.; Miller, A. J. M.; Sherden, N. H.; Gottlieb, H. E.; Nudelman, A.; Stoltz, B. M.; Bercaw, J. E.; Goldberg, K. I. NMR Chemical Shifts of Trace Impurities: Common Laboratory Solvents, Organics, and Gases in Deuterated Solvents Relevant to the Organometallic Chemist. *Organometallics* **2010**, *29*, 2176–2179.
2. Berger, F.; Plutschack, M. B.; Riegger, J.; Yu, W.; Speicher, S.; Ho, M.; Frank, N.; Ritter, T. Site-selective and versatile aromatic C−H functionalization by thianthrenation. *Nature* **2019**, *567*, 223–228.
3. Pan, X.; Fang, C.; Fantin, M.; Malhotra, N.; So, W. Y.; Peteanu, L. A.; Isse, A. A.; Gennaro, A.; Liu, P.; Matyjaszewski. K. Mechanism of Photoinduced Metal-Free Atom Transfer Radical Polymerization: Experimental and Computational Studies. *J. Am. Chem. Soc.* **2016**, *138*, 2411–2425.
